# The risk of acute kidney injury in colorectal cancer survivors: an english population-based matched cohort study

**DOI:** 10.1186/s12885-023-11329-9

**Published:** 2023-09-07

**Authors:** Kirsty Andresen, Helena Carreira, Helen Strongman, Helen I. McDonald, Sara Benitez-Majano, Kathryn E. Mansfield, Dorothea Nitsch, Laurie A. Tomlinson, Krishnan Bhaskaran

**Affiliations:** 1 Department of Non-Communicable Disease Epidemiology, Faculty of Epidemiology and Population Health, London School of Hygiene & Tropical Medicine, Keppel Street, London, WC1E 7HT UK; 2 Department of Infectious Disease Epidemiology, Faculty of Epidemiology and Population Health, London School of Hygiene & Tropical Medicine, Keppel Street, London, WC1E 7HT UK

**Keywords:** Colorectal neoplasms, Cancer survivors, Acute kidney injury, England

## Abstract

**Background:**

Colorectal cancer survival has improved in recent decades but there are concerns that survivors may develop kidney problems due to adverse effects of cancer treatment or complications of the cancer itself. We quantified the risk of acute kidney injury (AKI) in colorectal cancer survivors compared to people with no prior cancer.

**Methods:**

Retrospective matched cohort study using electronic health record primary care data from the Clinical Practice Research Datalink GOLD linked to hospital data in England (HES-APC). Individuals with colorectal cancer between 1997–2018 were individually matched on age, sex, and GP practice to people with no prior cancer. We used Cox models to estimate hazard ratios for an incident hospital diagnosis of AKI in colorectal cancer survivors compared to individuals without cancer, overall and stratified by time since diagnosis adjusted for other individual-level factors (adj-HR).

**Results:**

Twenty thousand three hundred forty colorectal cancer survivors were matched to 100,058 cancer-free individuals. Colorectal cancer survivors were at increased risk of developing AKI compared to people without cancer (adj-HR = 2.16; 95%CI 2.05–2.27). The HR was highest in the year after diagnosis (adj-HR 7.47, 6.66–8.37), and attenuated over time, but there was still increased AKI risk > 5 years after diagnosis (adj-HR = 1.26, 1.17–1.37). The association between colorectal cancer and AKI was greater for younger people, men, and those with pre-existing chronic kidney disease.

**Conclusions:**

Colorectal cancer survivors were at increased risk of AKI for several years after cancer diagnosis, suggesting a need to prioritise monitoring, prevention, and management of kidney problems in this group of cancer survivors.

**Supplementary Information:**

The online version contains supplementary material available at 10.1186/s12885-023-11329-9.

## Background

Colorectal cancer has a large burden of disease worldwide. In 2018, there were approximately 1.8 million new cases and 900,000 deaths globally [1]. Individuals with colorectal cancer could be at increased risk of acute kidney injury (AKI), for example due to complications of surgery, toxicities of systemic anti-cancer therapies, or complications arising from the cancer itself [2, 3]. Furthermore, shared risk factors could contribute to higher AKI risk in individuals with cancer. Development of AKI is not only associated with high mortality [4], but can also compromise cancer care, forcing a switch to renally safe but less effective treatment, or even discontinuation of therapy [3]. In light of improvements in cancer detection and treatment leading to longer median survival [5] there are concerns about the potential for longer-term adverse consequences of cancer and its treatment, including for renal health [6].

The study of AKI in individuals with cancer has largely been restricted to haematological cancers [7, 8] due to the suspected link with tumour lysis syndrome and malignant infiltration [9, 10]. However, a recent study in China found that half (50.1%) of all cancer-related AKI occurred in individuals with gastrointestinal cancers [11]. Despite this, few studies have quantified the risk of AKI in people diagnosed with gastrointestinal cancers. A study from Denmark found that 1- and 5-year incidence of AKI was higher in colon and rectal cancer than the average for cancer overall [2]. The AKI risks were compared between site-specific cancers, so it is unclear how AKI incidence in people who have had cancer compares to that of the general population. Other studies have focused on the risk of AKI as a short-term postoperative complication of colorectal cancer surgery [11–13], but lacked investigation of the longer-term risks.

AKI is associated with substantial morbidity and mortality, so preventing AKI could improve overall survival rates and quality of life in colorectal cancer patients in both the short and long term. We therefore aimed to quantify both short- and long-term risk of an incident hospital diagnosis of AKI in individuals who have had a prior diagnosis of colorectal cancer, relative to the people who have never had cancer.

## Methods

### Study design and study population

We conducted a retrospective matched cohort study using de-identified electronic health records from the Clinical Practice Research Datalink GOLD (CPRD GOLD) primary care database, linked to hospital admissions data from the Hospital Episode Statistics Admitted Patient Care database (HES-APC). CPRD GOLD is broadly representative of the UK population in terms of age, sex, and ethnicity [14]. It includes data collected as part of routine care on demographics, diagnoses, symptoms, consultations, test data including serum creatinine measurements, general practice-prescribed drugs, and lifestyle factors. Data are recorded by GPs and administration staff using Read codes [14]. CPRD GOLD practices in England that have consented can be linked to hospital inpatient data from HES-APC [14]. HES-APC contains information on diagnoses, procedures, and dates of admission and discharge from all admissions to National Health Service (NHS) hospitals in England. The International Classification of Diseases 10^th^ edition (ICD-10) are used to record diagnoses. Procedures, such as surgery, are classified using Office of Population Censuses and Surveys (OPCS) version 4.6 codes. Additionally, death registration data was obtained via linkage to the Office of National Statistics (ONS) mortality database and patient postcode measures of deprivation were obtained as a proxy measure of socioeconomic status via linkage to 2015 Index of Multiple Deprivation (IMD) data [15].

**Fig. 1:**
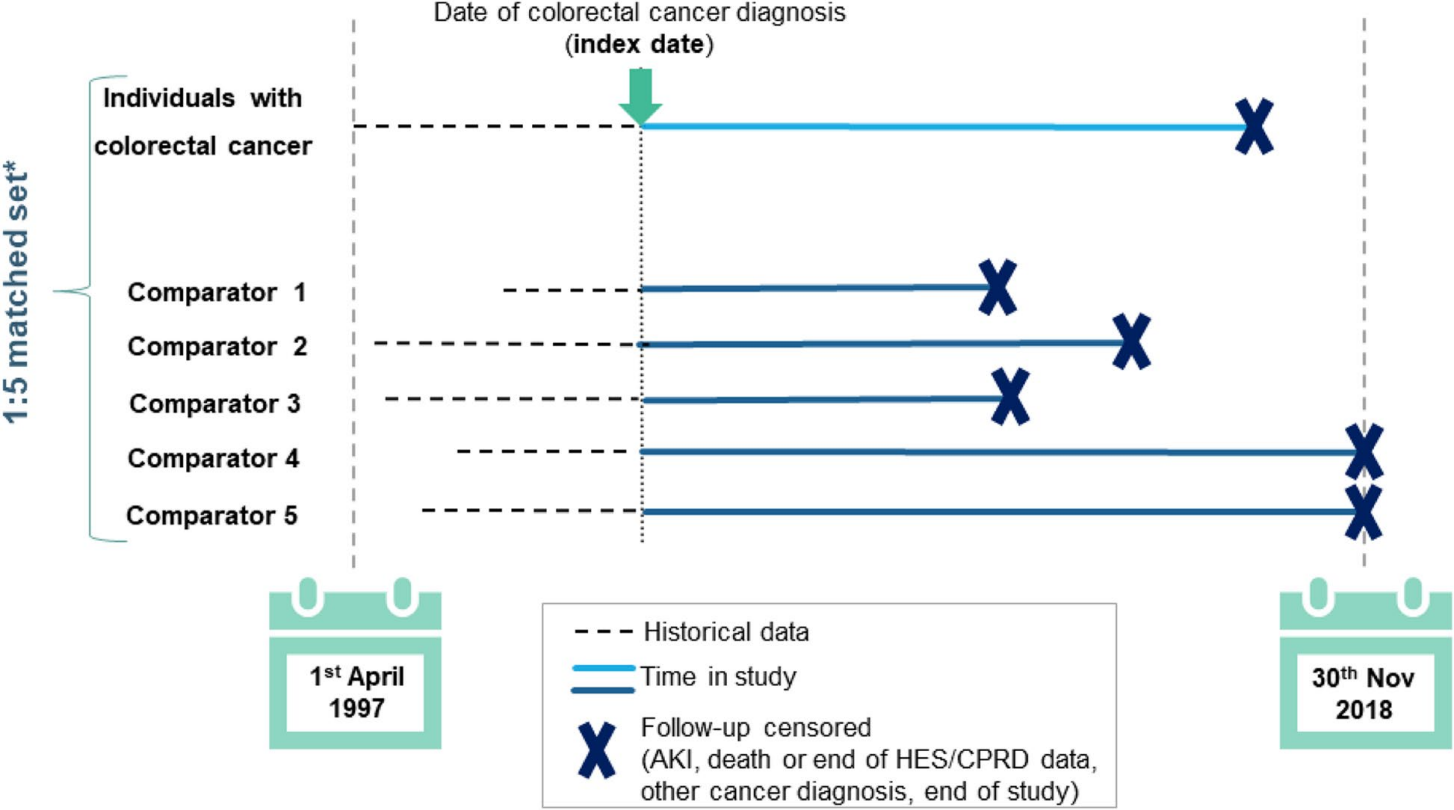
Illustration of study design AKI = acute kidney injury; HES/CPRD GOLD = Hospital Episode Statistics Admitted Patient Care/ Clinical Practice Research Datalink GOLD. *Individuals in the comparison cohort were matched on age (within 3-years), sex and GP practice

The study period was from the 1^st^ of April 1997 to the 30^th^ of November 2018. Individuals ≥ 18-years old eligible for linkage to HES were included in the study if they had a first ever record of colorectal cancer (colorectal cancer morbidity codes recorded in CPRD GOLD or HES) at least 12 months after start of follow-up and no prior record of cancer (other than colorectal), AKI or end-stage renal disease (ESRD). We regarded the date of incident colorectal cancer diagnosis as the date of entry into the study (henceforth index date), to understand the complete picture of survivorship from diagnosis, through the balance of their life [16]. Each colorectal cancer patient was then individually matched by GP practice, sex, and age (within 3-years), to up to 5 people without history of any cancer on the index date, and with at least 12 months of CPRD GOLD follow-up prior to that date (Fig. 1). Patients with a diagnosis of non-melanoma skin cancer were neither excluded nor censored.

We followed individuals from index date (i.e., date of first colorectal cancer diagnosis for those with colorectal cancer, and from the index date of their matched person with cancer for the matched comparator population) until the earliest of: first AKI diagnosis, transfer out of CPRD GOLD, end of study period (30^th^ November 2018), colorectal cancer diagnosis (in the control group), or death.

### Exposure, outcome, and covariates

Our exposure was colorectal cancer, individuals with colorectal cancer were identified if they had at least one Read code for colorectal cancer in CPRD GOLD, or an ICD-10 code in HES-APC. Our outcome, AKI, was defined as a record of at least one ICD-10 code for AKI as part of a hospital admission (previously validated for the identification of AKI in UK hospital data) [17]. Quintiles of individual-level deprivation, smoking (categorised as never, current, ex-smoker), problem drinking, body mass index (BMI), chronic kidney disease (CKD), diabetes mellitus, cardiovascular disease, hypertension, autoimmune disease, rheumatoid arthritis and ethnicity were selected a priori as confounders based on external evidence of associations with both colorectal cancer and AKI [1, 18, 19]. BMI was calculated using height and weight patient measures and categorised according to the World Health Organisation (WHO) definition of obesity [20]. Ethnicity was classified according to the 2001 UK census (white, South Asian, Black African/Caribbean, mixed, and other). Unless otherwise specified, lifestyle factors and comorbidities were classified as yes/no variables based on the presence of Read codes in their medical history at any time prior to the index date (See Additional file 1 for more details). Study code lists for are available for download at https://doi.org/10.17037/DATA.00002792.

### Statistical analyses

#### Primary analysis—AKI risks in colorectal cancer survivors and matched controls

We initially described the characteristics of individuals with and without colorectal cancer. Continuous non-normally distributed variables were described using medians and interquartile ranges (IQR). We estimated the crude incidence rates and hazard ratios (HR)s of first ever AKI in people with colorectal cancer, compared to matched comparators without a history of cancer. HRs were calculated using Cox proportional hazards regression stratified by matched set, with time since index date as the underlying timescale. Our base model (hereafter “minimally adjusted model”) implicitly adjusted for the matching variables as well as the underlying timescale. We then fitted the fully adjusted model, including all the a priori covariates except ethnicity (see sensitivity analyses). We checked for multicollinearity in the full model by comparing the standard errors between the minimally adjusted and fully adjusted models.

We also explored whether age, sex, time since index date (split into 0–1, 1, 2, 3, 4 and 5 + years), diabetes and CKD at baseline modified the effect of colorectal cancer on AKI by conducting Cox regression models stratified by the aforementioned variables. Diabetes and CKD diagnoses were chosen as potential effect modifiers according to previous literature where they were found to be associated with an increased risk of AKI in individuals receiving cancer therapy [21].

Individuals with missing data on a particular variable were omitted from analyses involving that variable (complete case analysis), an approach which is unbiased providing missingness is conditionally independent of the outcome [22]. We considered this assumption more plausible than the missing at random assumption required for multiple imputation, because recording in primary care may be dependent on the underlying value (e.g., smokers more likely than non-smokers to have smoking status recorded).

#### Secondary analysis – AKI risks by receipt of resectional colorectal cancer surgery

Surgery is a common treatment for colorectal cancer and has been highlighted as a potential cause of AKI in colorectal cancer patients [12]. We therefore conducted a secondary analysis where we identified patients who had resectional colorectal surgery recorded in HES data, to investigate how colorectal surgery affects the risk of AKI in colorectal cancer survivors. Resectional colorectal cancer surgery was defined using previously validated methods [23]. For this analysis, we ran the same model recategorizing colorectal cancer exposure as: 1) colorectal cancer with surgery; 2) colorectal cancer without surgery; and 3) general population comparators (no history of cancer). A p-value for heterogeneity was calculated by comparing this model in a likelihood ratio test with a simplified (nested) model combining categories 1 and 2.

#### Sensitivity analyses

Due to important levels of missing ethnicity data (57.7% of the study population (12,701 + 56,812) / (20,340 *100,058) = 0.5773 *100 = 57.73)), we did not adjust for ethnicity in the main analysis; instead, we conducted a sensitivity analysis including ethnicity in the fully adjusted model.

Finally, the potential for differential ascertainment of AKI due to enhanced kidney monitoring in the colorectal cancer group was assessed by comparing the rate of patients undergoing serum creatinine tests between those with colorectal cancer and those without. Statistical analyses were preformed using STATA 16 statistical software (Stata Corps, TX).

### Ethics

The study was approved by the London School of Hygiene and Tropical Medicine (LSHTM) Ethics Committee (ref 16,997) and CPRD’s Independent Scientific Advisory Committee (ISAC) (protocol 19_278).

## Results

### Characteristics of the study population

**Fig. 2:**
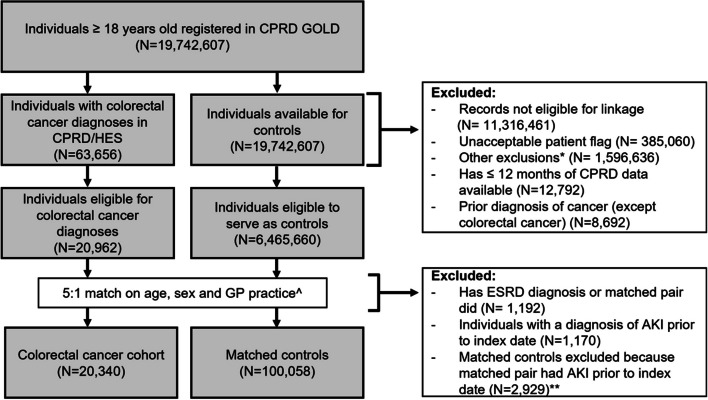
Study flow chart *Other quality issues include index date prior to database entry, indeterminate sex, aged < 18 years-old, end of follow-up prior to study entry. ^ *n* = 4 individuals with incident colorectal cancer excluded as no matches were not found. ** matched controls belonging to matched sets where the exposed individual had a prior diagnosis of AKI. AKI = acute kidney injury; CPRD/HES-APC = Clinical Practice Research Datalink/Hospital Episode Statistics Admitted Patient Care; ESRD = End Stage Renal Disease

The study population included 120,398 individuals (20,340 with colorectal cancer and 100,058 matched comparators) (Fig. 2), contributing a total of 915,470 person-years of follow-up (median follow-up 7.1 years, IQR 3.6–11.1, data not shown). The median age at index date in the colorectal cancer cohort was 73 (IQR 63–80) years (Table 1). The colorectal cancer and matched comparison cohorts were balanced in terms of demographic factors; 11,861/20,340 (58.3%) individuals in the colorectal cancer cohort had at least one of the defined comorbidities compared with 53,803/100,058 (53.8%) of the matched individuals without a history of cancer.

**Table 1:** Study population characteristics at baseline

	**Colorectal cancer cohort** **(*N* = 20,340)**		**Matched controls** **(*N* = 100,058)**	
**Median follow-up (years), IQR**	3.65 (0.93–8.34)		7.60 (4.26–11.49)	
**Age at index date**				
Median	73		72	
IQR	63–80		63–80	
Minimum	18		18	
Maximum	103		102	
**Calendar year of index date, n (%)**				
1997–1999	959	(4.7)	4,775	(4.8)
2000–2004	5,073	(24.9)	25,162	(25.2)
2005–2009	6,729	(33.1)	33,172	(33.2)
2010–2014	5,794	(28.5)	28,347	(28.3)
2015–2018	1,785	(8.8)	8,602	(9.6)
**Sex, n (%)**				
Female	9,253	(45.5)	45,598	(45.6)
**Ethnicity, n (%)**				
White	7,375	(36.3)	41,322	(41.3)
South Asian	112	(0.6)	928	(0.9)
Black	90	(0.4)	559	(0.6)
Other	52	(0.3)	324	(0.3)
Mixed	10	(0.1)	113	(0.1)
Unknown	12,701	(62.4)	56,812	(56.8)
**Index of Multiple Deprivation, n (%)**				
Quintile 1 (most deprived)	4,874	(24.0)	24,297	(24.3)
Quintile 2	4,788	(23.5)	23,559	(23.6)
Quintile 3	4,318	(21.2)	21,213	(21.2)
Quintile 4	3,505	(17.2)	17,067	(17.1)
Quintile 5 (least deprived)	2,833	(14.0)	13,829	(13.8)
Unknown	22	(0.1)	93	(0.1)
**Body Mass Index, Kg/m**^**2**^**, n (%)**				
Underweight (BMI < 18.5)	442	(2.2)	1,763	(1.8)
Normal (BMI 18.5 to < 25)	6,808	(33.5)	32,907	(32.9)
Pre-obesity (BMI 25.0 to < 30)	7,171	(35.3)	36,173	(36.2)
Obesity class I (BMI 30 to < 35)	2,844	(14.0)	14,000	(14.0)
Obesity class II (BMI 35 to < 40)	814	(4.0)	3,973	(4.0)
Obesity class III (BMI ≥ 40)	310	(1.5)	1,492	(1.5)
Unknown	1,951	(9.6)	9,750	(9.7)
**Smoking, n (%)**				
Never smoker	8,385	(41.2)	44,304	(44.3)
Past smoker	8,464	(41.6)	37,500	(37.5)
Current smoker	3,020	(14.9)	15,407	(15.4)
Unknown	471	(2.3)	2,847	(2.9)
**Alcohol use, n (%)**				
Problem drinker	2,401	(11.8)	9,976	(10.0)
**Comorbidities, n (%)**				
At least one comorbidity	11,861	(58.3)	53,803	(53.8)
Diabetes mellitus	2,523	(12.4)	10,043	(10.0)
Cardiovascular disease	6,775	(33.3)	31,048	(31.0)
Hypertension	3,619	(17.8)	10,598	(10.6)
Autoimmune disease	1,291	(6.2)	5,623	(5.6)
Rheumatoid arthritis	320	(1.6)	1,570	(1.6)
Chronic kidney disease (CKD) stage 3-5^a^	3,785	(18.61)	13,719	(13.71)

^a^CKD was determined by the presence of a relevant Read code or from eGFR rates estimated from serum creatinine values

### Primary analysis—AKI risks in colorectal cancer survivors and controls

**Fig. 3:**
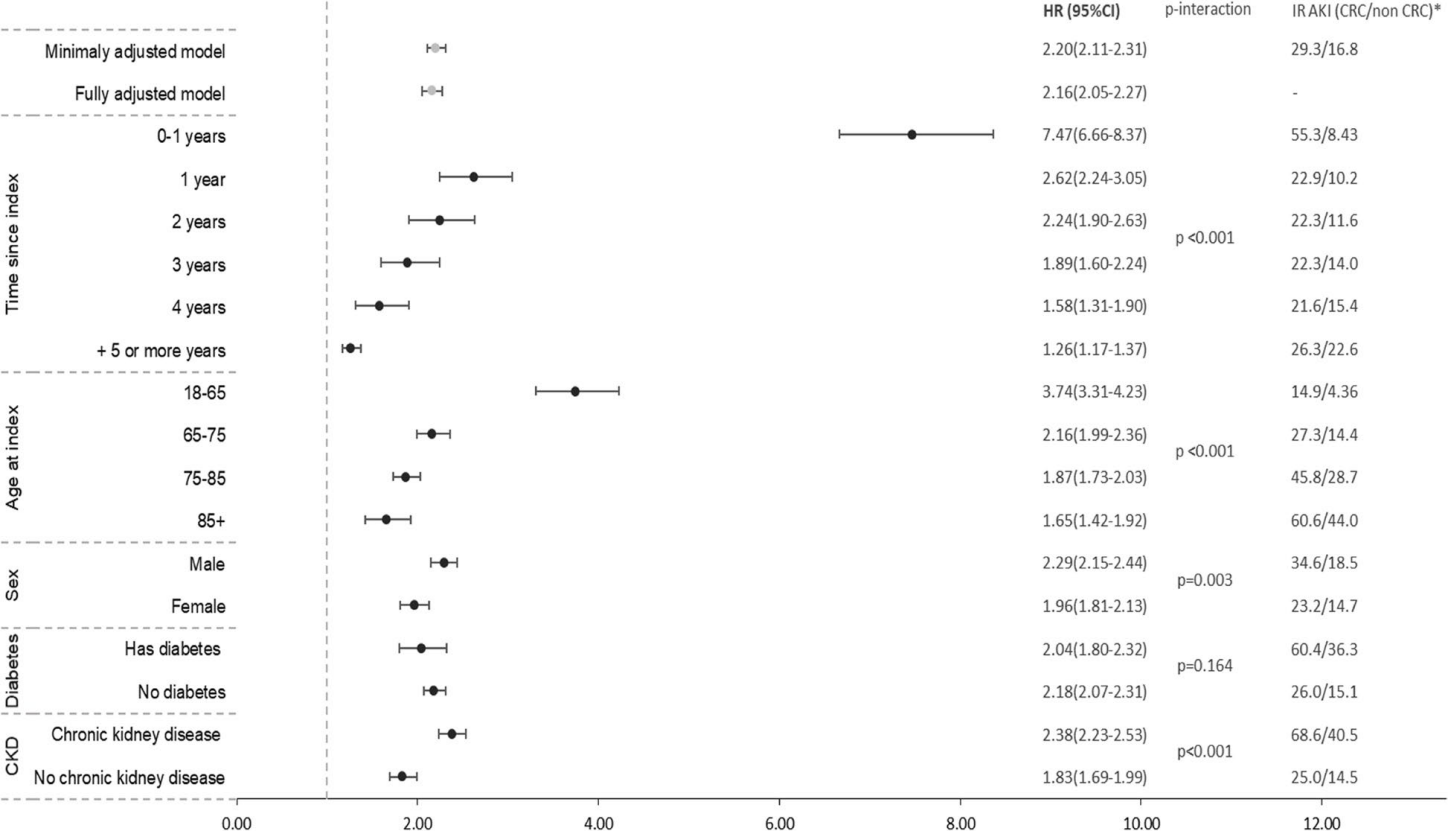
Stratum-specific HRsa for risk of AKI in colorectal cancer survivors compared with matched comparison cohort. Adjusted HRs were calculated using Cox proportional hazards models stratified by matched set with time since index date as the underlying timescale Total *N* = 108,837 and n AKI events = 15,612. ^a^Adjusted for age, sex, GP practice, quintile of relative deprivation, BMI category, smoking status, problem drinking, diabetes, cardiovascular disease, hypertension, autoimmune disease, rheumatoid arthritis, and chronic kidney disease. * Incidence rate of AKI in individuals with CRC per 1,000 patient years/ Incidence rate of AKI in individuals with no prior cancer per 1,000 patient years CKD = Chronic Kidney Disease; HR = Hazard ratio, IR = incidence rate

The incidence rate of AKI during follow-up was 29.3 (95% CI 28.3–30.3) per 1,000 person-years in those with colorectal cancer history, and 16.8 (95% CI 16.5–17.0) per 1,000 person-years in those with no history of cancer (Fig. 3).

The minimally adjusted HR for AKI, comparing people with colorectal cancer to those without was 2.20 (95% CI 2.11–2.31) (Fig. 3). After multivariate adjustment for social deprivation, BMI category, smoking status, problem drinking, diabetes, cardiovascular disease, hypertension, autoimmune disease, rheumatoid arthritis and CKD, the HR between colorectal cancer and AKI was 2.16 95% CI (2.05–2.27).

There was strong evidence that the relative risk of developing AKI in colorectal cancer survivors decreased over time since diagnosis (Likelihood ratio test (LRT) *p*-value for heterogeneity < 0.001), after adjusting for shared risk factors (Fig. 3). Individuals with colorectal cancer were 7.47 (95% CI 6.66–8.37) times more likely to develop AKI in the first year after cancer diagnosis than those without colorectal cancer. The relative risk decreased each successive year after cancer diagnosis, although there was still a 26% increased risk of AKI more than 5 years after diagnosis of colorectal cancer (HR: 1.26; 95% CI 1.17–1.37) compared to those who had never had cancer. There was also strong evidence that age, sex and CKD were effect modifiers of the association between colorectal cancer diagnosis and new onset AKI, with a greater association in younger individuals, men, and those with CKD (see Fig. 3). To note, stratum specific incidence rates were lower in younger individuals, as expected while absolute rates were higher in males and individuals with CKD.

### Secondary analyses – AKI risks by receipt of resectional colorectal cancer surgery

Compared to the matched cohort without a history of cancer, colorectal cancer survivors who did not have colorectal surgery were 2.86 (95% CI 2.60–3.13) times more likely to develop AKI, while those who did have surgery were 1.93 (95% CI 1.82–2.05) times more likely to develop AKI (*p*-value for heterogeneity =  < 0.001) (Table 2). The raised risks were most pronounced in the first year since diagnosis, for both those who did and did not receive surgery and attenuated over time in both groups.

**Table 2:** Adjusted Hazard ratios (HR) and stratum-specific HRs of incident AKI in individuals with a prior diagnosis of colorectal compared to matched individuals with no history of cancer, by exposure to resectional surgery for colorectal cancer and time since index date. HRs were calculated using Cox proportional hazards stratified by matched set with time since index date as the underlying timescale

			**95% CI**	
	**Category**	**Hazard Ratio**^**a**^	**Lower**	**Upper**
**Fully adjusted model**	colorectal cancer surgery	1.93	1.82	2.05
	colorectal cancer no surgery	2.86	2.60	3.13
**Time since index**		**Stratum-specific HR**^**a**^		
**0–1 years**	colorectal cancer surgery	7.87	6.78	9.13
	colorectal cancer no surgery	6.89	5.76	8.25
**1 year**	colorectal cancer surgery	2.16	1.77	2.64
	colorectal cancer no surgery	3.58	2.79	4.60
**2 years**	colorectal cancer surgery	1.86	1.53	2.26
	colorectal cancer no surgery	3.57	2.64	4.82
**3 years**	colorectal cancer surgery	1.69	1.38	2.06
	colorectal cancer no surgery	2.63	1.88	3.66
**4 years**	colorectal cancer surgery	1.47	1.18	1.83
	colorectal cancer no surgery	1.93	1.35	2.75
**5 or more years**	colorectal cancer surgery	1.25	1.15	1.37
	colorectal cancer no surgery	1.31	1.10	1.54

^a^Adjusted for age, sex, GP practice, social deprivation, BMI category, smoking status, problem drinking, diabetes, cardiovascular disease, hypertension, autoimmune disease, rheumatoid arthritis, and chronic kidney disease

### Sensitivity analyses

Including ethnicity in the fully adjusted model, made little difference to the results (HR adjusted model without ethnicity: 2.16 (95% CI 2.05–2.27) vs. HR adjusted model with ethnicity: 2.24 (95% CI 2.07–2.43).

Serum creatinine testing rates were higher in individuals with colorectal cancer than in matched individuals with no history of cancer. The testing rate was 728.3 (95% CI 716.5 – 740.2) per 1,000 person years in the colorectal cancer cohort vs. 430.3 (95% CI 427.3 – 433.3) per 1,000 person years in the matched controls.

## Discussion

### Key findings

Colorectal cancer survivors were more than twice as likely to have a new onset AKI than individuals with no prior history of cancer overall, and more than seven times more likely to experience AKI in the first year after colorectal cancer diagnoses; a smaller raised risk persisted more than five years after colorectal cancer diagnosis. Hazard ratios were larger in younger people, men, and those with a history of chronic kidney disease, though absolute stratum-specific incidence rates were lower in younger individuals.

### Findings in context

To the best of our knowledge, no previous population-based studies have compared the risk of AKI in people with cancer to individuals with no history of cancer, thus our effect estimates could not be compared to studies in other settings. However, there are a few large population-based studies that have quantified the incidence of AKI in colorectal cancer patients [2, 21, 24]. There was substantial variability of results between the studies thought to be mostly due to varying definitions of baseline creatinine measures. Our results are similar to those found in a study of individuals receiving systemic cancer therapy conducted in a health-insurance claims database in Ontario, Canada that also used a morbidity-coded AKI [21]. Our incidence rate estimate was lower than that of a Danish population-based study, where baseline serum creatinine was used to determine AKI, which was defined as the lowest serum creatinine value within a year of cancer diagnoses [2]. Similar to our study, the Danish study demonstrated that in people with cancer, AKI incidence is highest in the first year since cancer diagnoses and reduces over time (although remains increased at 5-years). Conversely, in a study of people hospitalised with cancer in China, the incidence of AKI in people with colorectal cancer was substantially lower than in our findings (5.5% vs 29.3% in our study). Patients were included over a period of two years (2013–2015). This study defined AKI based on baseline serum creatinine and at least two serum creatinine tests within 7 days of hospitalisation [24]. There may be selection bias as individuals who survive longer are more likely to have a second test; thus, the incidence of AKI could have been underestimated.

### Strengths and limitations

Our study included over 120,000 people, which allowed us to estimate the association between colorectal cancer history and AKI with high precision. CPRD GOLD primary care data has high validity across a range of diagnoses [14], and in combination with HES has high sensitivity for capturing cancer diagnoses [25]. We matched on the key variables of age, sex and general practice, and our use of detailed primary care data and linked data sources allowed us to adjust for a range of potential shared risk factors for colorectal cancer and AKI. We used population-based data from England, where healthcare is free at the point of access, so our results are likely to be generalisable to the UK and other comparable settings.

However, a limitation of our study is the lack of information on key cancer characteristics, including cancer stage and systemic anti-cancer treatment, which were not available in CPRD GOLD or HES-APC. Nephrotoxicity and immunosuppression are known complications of colorectal cancer treatment [26], treatment data would have allowed us to explore how these factors drive AKI in people with colorectal cancer, and in understanding the drivers of the increased risk of AKI in individuals with colorectal cancer surgery and those without surgery. People with colorectal cancer who are managed with and without resectional surgery are likely to have a different prognosis, potentially due to differences in cancer stage and treatment; in particular, individuals who do not undergo surgery may have more advanced-stage disease. Furthermore, AKI staging and severity could not be determined as serum creatinine measures are not available from HES-APC. We used ICD-10 codes recorded as part of a hospital admission to define AKI, which have been validated and are considered to have high specificity in identifying AKI [27] but some AKI cases may still have been missed, especially less severe presentations. The journey of a colorectal cancer patient through the healthcare system is likely different to that of an individual with no history of cancer, as evidenced by the difference in follow-up time, a limitation which we acknowledge. However, we endeavoured to minimise differences between groups by using a matched cohort design. Comparison to controls allows for the identification of conditions where cancer survivors may benefit from additional surveillance. Moreover, we explored the assumption of differential ascertainment of AKI due to closer follow-up in cancer patients by describing serum creatinine testing rates in the two groups, the rate of testing in primary care was found to be higher in cancer survivors than controls, meaning that individuals with colorectal cancer may be more likely to have AKI detected. Our focus was on AKI cases severe enough to require hospital admission, which are likely to be picked up regardless of any differential levels of health contacts in cancer survivors. Another limitation may be competing mortality risk. There is a high level of multimorbidity in this patient population, which may mean that AKI incidence may be lower than expected. There were some missing data BMI data for approximately 10% of our study sample, this could potentially introduce selection bias if missingness were related to the outcome risk, however we do not expect this to greatly impact the risk estimate at this proportion of missingness.

Another limitation may be competing mortality risk; this was handled by censoring people who died. This is a competing risks approach that considers the cause-specific hazard, which is appropriate for analyses based on aetiological questions [28]. Finally, incorrectly measured, or unmeasured confounders may have affected the results.

### Implications for clinical practice, public health and future research

Our results suggest that management of AKI risk may be an important factor to consider as part of the treatment and follow-up of colorectal cancer. The first year after diagnosis is a critical period for patients undergoing treatment for colorectal cancer. During this time, patients may undergo surgery to remove the cancerous tissue, as well as chemotherapy to eliminate any remaining cancer cells. Both of these treatments can put a significant strain on the kidneys and increase the risk of AKI thought to be driven by volume depletion and direct toxic effects of chemotherapy drugs [3]. Healthcare practitioners should consider implementing long-term monitoring of renal function markers (i.e., albuminuria and serum creatinine) as part of ongoing care and support. Potential preventative strategies could be initiated such as targeting modifiable risk factors for both diseases or correction of fluid and/or electrolyte imbalances. These measures are currently lacking from colorectal cancer management guidelines [29]. Further research is needed to understand the clinical and cost-effectiveness of these approaches. Furthermore, education and awareness of the risk of AKI may be important and must be phrased such that it is relevant and understood by at-risk populations groups.

Moreover, there is a need to further understand the drivers for the higher risk of AKI in colorectal cancer survivors, including the role of cancer stage and different types of cancer treatments.

## Conclusions

In conclusion, individuals with colorectal cancer are at increased risk of AKI, particularly in the first year after diagnosis although long-term risk of AKI remains in the years after colorectal cancer diagnosis. Our study implies a need for careful monitoring, prevention and management of kidney disease in colorectal cancer patients, especially in the early survivorship period. The implementation of kidney and cardiovascular risk factor management plans could potentially reduce the occurrence of AKI and overall cardiovascular risk in cancer survivors.

### Supplementary Information

**Additional file 1.**

## Data Availability

The data that support the findings of this study but are not publicly available due to CPRD licencing restrictions. Study code lists for are available for download at the on the Electronic Health Records Research Group Data Compass: https://doi.org/10.17037/DATA.00002792.

## Abbreviations

Adj-HR: Adjusted Hazard Ratios
AKI: Acute kidney injury
BMI: Body Mass Index
CKD: Chronic Kidney Disease
CPRD GOLD: Clinical Practice Research Datalink GOLD
ESRD: End-stage renal disease
GP: General Practitioner
HES-APC: Hospital Episode Statistics—Admitted Patient Care
HR: Hazard Ratio
ICD-10: International Classification of Diseases 10^th^ edition
IMD: Index of Multiple Deprivation
IQR: Interquartile ranges
LRT: Likelihood ratio test
NHS: National Health Service
ONS: Office of National Statistics
OPCS: Office of Population Censuses and Surveys
UK: United Kingdom
WHO: World Health Organisation
