# Supplementary material for: The risk of acute kidney injury in colorectal cancer survivors: an english population-based matched cohort study
Source: BMC Cancer. 2023 Sep 7;23:839. doi: 10.1186/s12885-023-11329-9 (PMC10483792; doi:10.1186/s12885-023-11329-9)
Supplement: Supplementary file 1 — Additional file 1. [file 12885_2023_11329_MOESM1_ESM.docx]

# Study variables “The risk of acute kidney injury in colorectal cancer survivors: An English population-based matched cohort study”

| **Covariate** | **Definition and use** | **Derivation** |
| --- | --- | --- |
| **Demographic** |  |  |
| Age | Matching variable (within 3 years) and descriptive variable as continuous variable and categorical interaction term (18-65, 65-75, 75-85, 85+) | 01/07/birthyear (Exact date of birth not collected by CPRD GOLD to maintain the de-identified nature of the data) |
| Calendar year of cancer diagnosis | Date of first ever diagnosis of colorectal cancer or corresponding date in matched individual categorised (1997-1999, 2000-2004, 2005-2009, 2010-2014, 2015-2018) |  |
| Sex | Matching variable, descriptive variable and interaction term. Binary (male, female); | People of unknown sex were dropped |
| Ethnicity | Descriptive variable. Excluded from main model due to high proportion of missingness. Categorised (White, South Asian, Black, Other, Mixed or unknown) | Previously defined algorithm using Read codes.  *Rohini Mathur, Krishnan Bhaskaran, Nish Chaturvedi, David A. Leon, Tjeerd vanStaa, Emily Grundy, Liam Smeeth, Completeness and usability of ethnicity data in UK-based primary care and hospital databases, Journal of Public Health, Volume 36, Issue 4, December 2014, Pages 684–692, https://doi.org/10.1093/pubmed/fdt116* |
| **Risk factors** |  |  |
| Index of multiple deprivation | Ordered categorical covariate used as a proxy for socio-economic status (1 least deprived, 2, 3, 4, 5 most deprived). Individuals with missing data excluded from all models | Index of multiple deprivation (IMD) quintile identified by CPRD through third party linkage to patient postcode. This small-area deprivation scale was applied at patient postcode level |
| Body Mass Index | Categorical variable categorised as Underweight (BMI <18.5), Normal (BMI 18.5 to <25), Pre-obesity (BMI 25.0 to <30), Obesity class I (BMI 30 to <35), Obesity class II (BMI 35 to <40), Obesity class III (BMI ≥40)) based on WHO categories. Individuals with missing data excluded from all models | Calculated from nearest structured record of weight and height. Records prior and nearer the index date were prioritised. Implausible measurements excluded.  *Bhaskaran K, Forbes HJ, Douglas I, Leon DA, Smeeth L. Representativeness and optimal use of body mass index (BMI) in the UK Clinical Practice Research Datalink (CPRD). BMJ Open. 2013 Sep 13;3(9):e003389. doi: 10.1136/bmjopen-2013-003389. PMID: 24038008; PMCID: PMC3773634.* |
| Smoking | Categorical variable  (non smoker, past smoker, current smoker). | Nearest structured or Read coded record of smoking status to the index date. Records of non-smokers were re-categorised as ex-smokers if there was a previous record of smoking. |
| Alcohol use | Binary variable (Problem drinker, non-problem drinker). | Read-coded record of problem drinking (indicating heavy drinking, alcoholism) prior to index date. Individuals without a record for alcohol consumption were considered non-problem drinkers. |
| Diabetes mellitus | Descriptive variable and interaction term. Binary shared risk factor (yes/no) and interaction term | Presence of Read codes in their medical history at any time prior to the index date or up to 30 days after index date (assumed prevalent disease). |
| Cardiovascular disease | Binary variable (yes/no) | Record of cardiovascular disease prior to baseline (Read codes in CPRD GOLD or ICD-10 codes in HES) at any time prior to index date or up to 30 days after index date. Includes fatal or non-fatal coronary artery disease (angina, myocardial infarction, revascularisation procedures, and sudden cardiac arrest), stroke (haemorrhagic and ischaemic stroke), arrhythmia, venous thromboembolism (deep vein thrombosis and pulmonary embolism), heart failure and cardiomyopathy combined, pericarditis, valvular heart disease, and peripheral vascular disease. |
| Hypertension | Binary variable (yes/no). Individuals without as least two blood pressure measures, including one in the 365 days prior to baseline, are classified as “no hypertension” | Read code or ICD-10 code for hypertension in the clinical record prior to the index date or up to 30 days after index date. |
| Autoimmune disease | Binary variable (yes/no) | Read-coded record of an autoimmune disease at any time prior or 30 days after index date that are considered to have a positive association with colorectal cancer. These include ulcerative colitis, Crohn’s disease, coeliac disease, lupus, primary biliary cirrhosis and sarcoidosis. |
| Rheumatoid arthritis | Binary variable (yes/no) | Read-coded record of rheumatoid arthritis at any time prior or up to 30 days after index date. Separated from other autoimmune diseases as it is considered to have a negative association with colorectal cancer. |
| Ever had a serum creatinine test | Binary variable (yes/no). The result was stored and used to calculate eGFR and categorise Chronic Kidney Disease stage | Read-coded instance of serum creatinine test. |
| Chronic Kidney Disease | Descriptive variable and interaction term. Binary variable (yes/no). | Read coded record of CKD or eGFR estimated from serum creatinine measures indicating stage 3-5 disease. |
| Chronic Kidney Disease staging | Categorisation of individuals with a record of chronic kidney disease and serum creatinine test with which an estimated glomerular filtration rate could be calculated. Stage 3 (eGFR 30-59), stage 4 (eGFR 15-29), stage 5 (eGFR <15). | Most recent cleaned* serum creatine value recorded prior to index date used to calculate eGFR. Categories: no CKD >=60 mL/min, stage 3 30-<60 mL/min, stage 4 15-<30, stage 5 <15 mL/min. Stage 5 includes patients in dialysis. |
| Cancer surgery | Used to recategorise the exposure variable into three categories (colorectal cancer with surgery, colorectal cancer without surgery, no history of colorectal cancer) | Classification of Intervention and Procedures of the Office of Population Censuses and Surveys (OPCS), version 4 code for major colorectal surgery record  in the -30 days to +180 days of the index date. |

# Codelists

# Exposure

## Colorectal cancer (medcode, Read and ICD-10)

| medcode | readcode | icdcode | description |
| --- | --- | --- | --- |
| 108905 | 68W2400 | C18 | Bowel scope (flexible sigmoidoscopy) screen: cancer detected |
| 22163 | B134.11 | C18 | Carcinoma of caecum |
| 9118 | B13z.11 | C18 | Colonic cancer |
| 101700 | B139.00 | C18 | Hereditary nonpolyposis colon cancer |
| 18632 | B135.00 | C18 | Malignant neoplasm of appendix |
| 10946 | B136.00 | C18 | Malignant neoplasm of ascending colon |
| 3811 | B134.00 | C18 | Malignant neoplasm of caecum |
| 1220 | B13..00 | C18 | Malignant neoplasm of colon |
| 28163 | B13z.00 | C18 | Malignant neoplasm of colon nos |
| 10864 | B132.00 | C18 | Malignant neoplasm of descending colon |
| 9088 | B130.00 | C18 | Malignant neoplasm of hepatic flexure of colon |
| 48231 | B13y.00 | C18 | Malignant neoplasm of other specified sites of colon |
| 2815 | B133.00 | C18 | Malignant neoplasm of sigmoid colon |
| 18619 | B137.00 | C18 | Malignant neoplasm of splenic flexure of colon |
| 6935 | B131.00 | C18 | Malignant neoplasm of transverse colon |
| 93478 | B138.00 | C18 | Malignant neoplasm, overlapping lesion of colon |
| 73275 | BB5N100 | C18 | [M]adenocarcinoma in adenomatous polposis coli |
| 55468 | BB5R600 | C18 | [M]mucocarcinoid tumour, malignant |
| 27855 | B140.00 | C19 | Malignant neoplasm of rectosigmoid junction |
| 7219 | B141.11 | C20 | Carcinoma of rectum |
| 1800 | B141.00 | C20 | Malignant neoplasm of rectum |
| 5901 | B141.12 | C20 | Rectal carcinoma |

## Colorectal surgery (OPCS)

| opcs | opcs_desc |
| --- | --- |
| H04 | Total excision of colon and rectum (Clean-Contaminated) |
| H041 | Panproctocolectomy and ileostomy |
| H042 | Panproctocolectomy and anastomosis of ileum to anus and creation of pouch however further qualified |
| H043 | Panproctocolectomy and anastomosis of ileum to anus not elsewhere classified |
| H048 | Other specified |
| H049 | Unspecified |
| H05 | Total excision of colon (Clean-Contaminated) |
| H051 | Total colectomy and anastomosis of ileum to rectum |
| H052 | Total colectomy and ileostomy and creation of rectal fistula however further qualified |
| H053 | Total colectomy and ileostomy not elsewhere classified |
| H058 | Other specified |
| H059 | Unspecified |
| H06 | Extended excision of right hemicolon (Clean-Contaminated) |
| H061 | Extended right hemicolectomy and end to end anastomosis |
| H062 | Extended right hemicolectomy and anastomosis of ileum to colon |
| H063 | Extended right hemicolectomy and anastomosis not elsewhere classified |
| H064 | Extended right hemicolectomy and ileostomy however further qualified |
| H065 | Extended right hemicolectomy and end to side anastomosis |
| H068 | Other specified |
| H069 | Unspecified |
| H07 | Other excision of right hemicolon (Clean-Contaminated) |
| H071 | Right hemicolectomy and end to end anastomosis of ileum to colon |
| H072 | Right hemicolectomy and side to side anastomosis of ileum to transverse colon |
| H073 | Right hemicolectomy and anastomosis not elsewhere classified |
| H074 | Right hemicolectomy and ileostomy however further qualified |
| H075 | Right hemicolectomy and end to side anastomosis |
| H078 | Other specified |
| H079 | Unspecified |
| H08 | Excision of transverse colon (Clean-Contaminated) |
| H081 | Transverse colectomy and end to end anastomosis |
| H082 | Transverse colectomy and anastomosis of ileum to colon |
| H083 | Transverse colectomy and anastomosis not elsewhere classified |
| H084 | Transverse colectomy and ileostomy however further qualified |
| H085 | Transverse colectomy and exteriorisation of bowel not elsewhere classified* |
| H086 | Transverse colectomy and end to side anastomosis |
| H088 | Other specified |
| H089 | Unspecified |
| H09 | Excision of left hemicolon (Clean-Contaminated) |
| H091 | Left hemicolectomy and end to end anastomosis of colon to rectum |
| H092 | Left hemicolectomy and end to end anastomosis of colon to colon |
| H093 | Left hemicolectomy and anastomosis not elsewhere classified |
| H094 | Left hemicolectomy and ileostomy however further qualified |
| H095 | Left hemicolectomy and exteriorisation of bowel not elsewhere classified* |
| H096 | Left hemicolectomy and end to side anastomosis |
| H098 | Other specified |
| H099 | Unspecified |
| H10 | Excision of sigmoid colon (Clean-Contaminated) |
| H101 | Sigmoid colectomy and end to end anastomosis of ileum to rectum |
| H102 | Sigmoid colectomy and anastomosis of colon to rectum |
| H103 | Sigmoid colectomy and anastomosis not elsewhere classified |
| H104 | Sigmoid colectomy and ileostomy however further qualified |
| H105 | Sigmoid colectomy and exteriorisation of bowel not elsewhere classified* |
| H106 | Sigmoid colectomy and end to side anastomosis |
| H108 | Other specified |
| H109 | Unspecified |
| H11 | Other excision of colon (Clean-Contaminated) |
| H111 | Colectomy and end to end anastomosis of colon to colon not elsewhere classified |
| H112 | Colectomy and side to side anastomosis of ileum to colon not elsewhere classified |
| H113 | Colectomy and anastomosis not elsewhere classified |
| H114 | Colectomy and ileostomy not elsewhere classified |
| H115 | Colectomy and exteriorisation of bowel not elsewhere classified* |
| H116 | Colectomy and end to side anastomosis NEC |
| H118 | Other specified |
| H119 | Unspecified |
| H12 | Extirpation of lesion of colon |
| H121 | Excision of diverticulum of colon (Dirty) |
| H122 | Excision of lesion of colon to not elsewhere classified |
| H123 | Destruction of lesion of colon not elsewhere classified |
| H128 | Other specified |
| H129 | Unspecified |
| H29 | Subtotal excision of colon and rectum (Clean contaminated) |
| H291 | Subtotal excision of colon and rectum and creation of colonic pouch and anastomosis of colon to anus |
| H292 | Subtotal excision of colon and rectum and creation of colonic pouch NEC |
| H293 | Subtotal excision of colon and creation of colonic pouch and anastomosis of colon to rectum. |
| H294 | Subtotal excision of colon and creation of colonic pouch NEC |
| H298 | Other specified subtotal excision of colon |
| H299 | Unspecified subtotal excision of colon |
| H33 | Excision of rectum (Clean contaminated) |
| H331 | Abdominoperineal excision of rectum and end colostomy |
| H332 | Proctectomy and anastomosis of colon to anus |
| H333 | Anterior resection of rectum and anastomosis of colon to rectum using staples |
| H334 | Anterior resection of rectum and anastomosis not elsewhere classified |
| H335 | Rectosigmoidectomy and closure of rectal stump and exteriorisation of bowel* |
| H336 | Anterior resection of rectum and exteriorisation of bowel* |
| H337 | Perineal resection of rectum HFQ |
| H338 | Other specified |
| H339 | Unspecified |
| H341 | Open excision of lesion of rectum |
| H342 | Open cauterisation of lesion of rectum |
| H343 | Open cryotherapy to lesion of rectum |
| H344 | Open laser destruction of lesion of rectum |
| H345 | Open destruction of lesion of rectum NEC |
| H348 | Other specified open extirpation of lesion of rectum |
| H349 | Unspecified open extirpation of lesion of rectum |
| H401 | Trans-sphincteric excision of mucosa of rectum |
| H402 | Trans-sphincteric excision of lesion of rectum |
| H403 | Trans-sphincteric destruction of lesion of rectum |
| H404 | Trans-sphincteric anastomosis of colon to anus |
| H408 | Other specified operations on rectum through anal sphincter |
| H409 | Unspecified operations on rectum through anal sphincter |
| H411 | Rectosigmoidectomy and peranal anastomosis |
| H412 | Peranal excision of lesion of rectum |
| H413 | Peranal destruction of lesion of rectum |
| H414 | Peranal mucosal proctectomy and endoanal anastomosis |
| H415 | Peranal resection of rectum using staples |
| H418 | Other specified other operations on rectum through anus |
| H419 | Unspecified other operations on rectum through anus |
| X141 | Total exenteration of pelvis |
| X142 | Anterior exenteration of pelvis |
| X143 | Posterior exenteration of pelvis |
| X148 | Other specified clearance of pelvis |
| X149 | Unspecified clearance of pelvis |

# Exclusion criteria

## All cancers except CRC (medcode, Read)*

| medcode | readcode | Description |
| --- | --- | --- |
| 9984 | B00..11 | Carcinoma of lip |
| 96783 | B005.00 | Malignant neoplasm of commissure of lip |
| 14712 | B00..00 | Malignant neoplasm of lip |
| 112660 | B004100 | Malignant neoplasm of lip unspecified, frenulum |
| 61692 | B004.00 | Malignant neoplasm of lip unspecified, inner aspect |
| 68399 | B004200 | Malignant neoplasm of lip unspecified, mucosa |
| 100144 | B004300 | Malignant neoplasm of lip, oral aspect |
| 37553 | B007.00 | Malignant neoplasm of lip, unspecified |
| 100906 | B00z000 | Malignant neoplasm of lip, unspecified, external |
| 94251 | B00z100 | Malignant neoplasm of lip, unspecified, lipstick area |
| 69761 | B00zz00 | Malignant neoplasm of lip, vermilion border nos |
| 67504 | B003000 | Malignant neoplasm of lower lip, buccal aspect |
| 66384 | B001000 | Malignant neoplasm of lower lip, external |
| 91843 | B003100 | Malignant neoplasm of lower lip, frenulum |
| 71147 | B003.00 | Malignant neoplasm of lower lip, inner aspect |
| 96782 | B003z00 | Malignant neoplasm of lower lip, inner aspect nos |
| 95480 | B001100 | Malignant neoplasm of lower lip, lipstick area |
| 89909 | B003200 | Malignant neoplasm of lower lip, mucosa |
| 94441 | B003300 | Malignant neoplasm of lower lip, oral aspect |
| 67446 | B001.00 | Malignant neoplasm of lower lip, vermilion border |
| 101707 | B001z00 | Malignant neoplasm of lower lip, vermilion border nos |
| 18882 | B006.00 | Malignant neoplasm of overlapping lesion of lip |
| 111289 | B002000 | Malignant neoplasm of upper lip, buccal aspect |
| 66270 | B000000 | Malignant neoplasm of upper lip, external |
| 99001 | B002100 | Malignant neoplasm of upper lip, frenulum |
| 99493 | B002.00 | Malignant neoplasm of upper lip, inner aspect |
| 100721 | B002z00 | Malignant neoplasm of upper lip, inner aspect nos |
| 50296 | B000100 | Malignant neoplasm of upper lip, lipstick area |
| 98500 | B002200 | Malignant neoplasm of upper lip, mucosa |
| 90610 | B002300 | Malignant neoplasm of upper lip, oral aspect |
| 73962 | B000.00 | Malignant neoplasm of upper lip, vermilion border |
| 98740 | B000z00 | Malignant neoplasm of upper lip, vermilion border nos |
| 58121 | B014.00 | Malignant neoplasm of anterior 2/3 of tongue unspecified |
| 43431 | B010.00 | Malignant neoplasm of base of tongue |
| 34409 | B010000 | Malignant neoplasm of base of tongue dorsal surface |
| 69671 | B010.11 | Malignant neoplasm of posterior third of tongue |
| 40557 | B01z.00 | Malignant neoplasm of tongue nos |
| 37096 | B015.00 | Malignant neoplasm of tongue, junctional zone |
| 36161 | B012.00 | Malignant neoplasm of tongue, tip and lateral border |
| 113897 | B011000 | Malignant neoplasm of anterior 2/3 of tongue dorsal surface |
| 102142 | B013000 | Malignant neoplasm of anterior 2/3 of tongue ventral surface |
| 43642 | B011.00 | Malignant neoplasm of dorsal surface of tongue |
| 43781 | B011z00 | Malignant neoplasm of dorsum of tongue nos |
| 91035 | B010z00 | Malignant neoplasm of fixed part of tongue nos |
| 63979 | B013100 | Malignant neoplasm of frenulum linguae |
| 24852 | B016.00 | Malignant neoplasm of lingual tonsil |
| 107258 | B011100 | Malignant neoplasm of midline of tongue |
| 41530 | B01y.00 | Malignant neoplasm of other sites of tongue |
| 10283 | B01..00 | Malignant neoplasm of tongue |
| 62840 | B013.00 | Malignant neoplasm of ventral surface of tongue |
| 38488 | B013z00 | Malignant neoplasm of ventral tongue surface nos |
| 47205 | B017.00 | Malignant overlapping lesion of tongue |
| 43400 | B03..00 | Malignant neoplasm of gum |
| 93218 | B03z.00 | Malignant neoplasm of gum nos |
| 49360 | B031.00 | Malignant neoplasm of lower gum |
| 101753 | B03y.00 | Malignant neoplasm of other sites of gum |
| 32024 | B030.00 | Malignant neoplasm of upper gum |
| 45408 | B040.00 | Malignant neoplasm of anterior portion of floor of mouth |
| 20092 | B04..00 | Malignant neoplasm of floor of mouth |
| 36716 | B04z.00 | Malignant neoplasm of floor of mouth nos |
| 45986 | B041.00 | Malignant neoplasm of lateral portion of floor of mouth |
| 56709 | B04y.00 | Malignant neoplasm of other sites of floor of mouth |
| 37916 | B05y.00 | Malignant neoplasm of other specified mouth parts |
| 17912 | B042.00 | Malignant neoplasm, overlapping lesion of floor of mouth |
| 99185 | B062100 | Malignant neoplasm of glossopalatine fold |
| 37590 | B052.00 | Malignant neoplasm of hard palate |
| 96003 | B055000 | Malignant neoplasm of junction of hard and soft palate |
| 28559 | B055z00 | Malignant neoplasm of palate nos |
| 70819 | B055.00 | Malignant neoplasm of palate unspecified |
| 61510 | B062200 | Malignant neoplasm of palatoglossal arch |
| 93842 | B062300 | Malignant neoplasm of palatopharyngeal arch |
| 69951 | B055100 | Malignant neoplasm of roof of mouth |
| 40292 | B053.00 | Malignant neoplasm of soft palate |
| 37516 | B054.00 | Malignant neoplasm of uvula |
| 30402 | B050.11 | Malignant neoplasm of buccal mucosa |
| 31364 | B050.00 | Malignant neoplasm of cheek mucosa |
| 41931 | B550100 | Malignant neoplasm of cheek nos |
| 97530 | B051100 | Malignant neoplasm of lower buccal sulcus |
| 55015 | B05z.00 | Malignant neoplasm of mouth nos |
| 14792 | B05..00 | Malignant neoplasm of other and unspecified parts of mouth |
| 37724 | B056.00 | Malignant neoplasm of retromolar area |
| 95772 | B051000 | Malignant neoplasm of upper buccal sulcus |
| 103796 | B051.00 | Malignant neoplasm of vestibule of mouth |
| 112528 | B051z00 | Malignant neoplasm of vestibule of mouth nos |
| 62182 | B200300 | Malignant neoplasm of vestibule of nose |
| 4388 | B020.00 | Malignant neoplasm of parotid gland |
| 43761 | B451.00 | Malignant neoplasm of labia majora |
| 59362 | B451z00 | Malignant neoplasm of labia majora nos |
| 50475 | B02z.00 | Malignant neoplasm of major salivary gland nos |
| 20292 | B02..00 | Malignant neoplasm of major salivary glands |
| 70696 | B02y.00 | Malignant neoplasm of other major salivary glands |
| 70928 | B022.00 | Malignant neoplasm of sublingual gland |
| 51786 | B021.00 | Malignant neoplasm of submandibular gland |
| 16902 | BB5y000 | [M]basal cell adenocarcinoma |
| 37510 | BBLG.00 | [M]carcinoma in pleomorphic adenoma |
| 51926 | B062000 | Malignant neoplasm of faucial pillar |
| 26448 | B060000 | Malignant neoplasm of faucial tonsil |
| 102151 | B060200 | Malignant neoplasm of overlapping lesion of tonsil |
| 101988 | B060100 | Malignant neoplasm of palatine tonsil |
| 46548 | B071100 | Malignant neoplasm of pharyngeal tonsil |
| 16241 | B060.00 | Malignant neoplasm of tonsil |
| 24397 | B061.00 | Malignant neoplasm of tonsillar fossa |
| 100002 | B062z00 | Malignant neoplasm of tonsillar fossa nos |
| 55066 | B062.00 | Malignant neoplasm of tonsillar pillar |
| 53884 | B060z00 | Malignant neoplasm tonsil nos |
| 73439 | B064z00 | Malignant neoplasm of anterior epiglottis nos |
| 113398 | B06y000 | Malignant neoplasm of branchial cleft |
| 55374 | B215.00 | Malignant neoplasm of epiglottis nos |
| 26134 | B064000 | Malignant neoplasm of epiglottis, free border |
| 48519 | B065.00 | Malignant neoplasm of junctional region of epiglottis |
| 56355 | B066.00 | Malignant neoplasm of lateral wall of oropharynx |
| 22893 | B06..00 | Malignant neoplasm of oropharynx |
| 43200 | B06z.00 | Malignant neoplasm of oropharynx nos |
| 67323 | B06y.00 | Malignant neoplasm of oropharynx, other specified sites |
| 91037 | B06yz00 | Malignant neoplasm of other specified site of oropharynx nos |
| 90124 | B067.00 | Malignant neoplasm of posterior wall of oropharynx |
| 39554 | B063.00 | Malignant neoplasm of vallecula |
| 33388 | B071000 | Malignant neoplasm of adenoid |
| 44139 | B073.00 | Malignant neoplasm of anterior wall of nasopharynx |
| 100918 | B073z00 | Malignant neoplasm of anterior wall of nasopharynx nos |
| 59004 | B072.00 | Malignant neoplasm of lateral wall of nasopharynx |
| 102205 | B072z00 | Malignant neoplasm of lateral wall of nasopharynx nos |
| 106915 | B073100 | Malignant neoplasm of nasopharyngeal soft palate surface |
| 24675 | B07..00 | Malignant neoplasm of nasopharynx |
| 28665 | B07z.00 | Malignant neoplasm of nasopharynx nos |
| 55630 | B07y.00 | Malignant neoplasm of other specified site of nasopharynx |
| 95429 | B071.00 | Malignant neoplasm of posterior wall of nasopharynx |
| 96869 | B071z00 | Malignant neoplasm of posterior wall of nasopharynx nos |
| 94390 | B070.00 | Malignant neoplasm of roof of nasopharynx |
| 66422 | B074.00 | Malignant neoplasm, overlapping lesion of nasopharynx |
| 39897 | B081.00 | Malignant neoplasm of pyriform sinus |
| 57248 | B082.00 | Malignant neoplasm aryepiglottic fold, hypopharyngeal aspect |
| 91895 | B064100 | Malignant neoplasm of glossoepiglottic fold |
| 34012 | B08..00 | Malignant neoplasm of hypopharynx |
| 28451 | B08z.00 | Malignant neoplasm of hypopharynx nos |
| 39084 | B0z2.00 | Malignant neoplasm of laryngopharynx |
| 88362 | B08y.00 | Malignant neoplasm of other specified hypopharyngeal site |
| 43548 | B080.00 | Malignant neoplasm of postcricoid region |
| 89916 | B553100 | Malignant neoplasm of presacral region |
| 24374 | B0...11 | Carcinoma of lip, oral cavity and pharynx |
| 46114 | B0z..00 | Malig neop other/ill-defined sites lip, oral cavity, pharynx |
| 19415 | B0...00 | Malignant neoplasm of lip, oral cavity and pharynx |
| 39430 | B0zz.00 | Malignant neoplasm of lip, oral cavity and pharynx nos |
| 49758 | B0zy.00 | Malignant neoplasm of other sites lip, oral cavity, pharynx |
| 37940 | B072000 | Malignant neoplasm of pharyngeal recess |
| 16297 | B0z0.00 | Malignant neoplasm of pharynx unspecified |
| 64462 | B083.00 | Malignant neoplasm of posterior pharynx |
| 98537 | B201100 | Malignant neoplasm of tympanic cavity |
| 95016 | B0z1.00 | Malignant neoplasm of waldeyer's ring |
| 58973 | Byu0.00 | [X]malignant neoplasm of lip, oral cavity and pharynx |
| 63470 | B102.00 | Malignant neoplasm of abdominal oesophagus |
| 61695 | B100.00 | Malignant neoplasm of cervical oesophagus |
| 42416 | B105.00 | Malignant neoplasm of lower third of oesophagus |
| 54171 | B104.00 | Malignant neoplasm of middle third of oesophagus |
| 1062 | B10..00 | Malignant neoplasm of oesophagus |
| 30700 | B10z.00 | Malignant neoplasm of oesophagus nos |
| 53591 | B10y.00 | Malignant neoplasm of other specified part of oesophagus |
| 41362 | B101.00 | Malignant neoplasm of thoracic oesophagus |
| 50789 | B103.00 | Malignant neoplasm of upper third of oesophagus |
| 67497 | B106.00 | Malignant neoplasm, overlapping lesion of oesophagus |
| 4865 | B10z.11 | Oesophageal cancer |
| 98142 | B107.00 | Siewert type i adenocarcinoma |
| 65312 | B11y000 | Malignant neoplasm of anterior wall of stomach nec |
| 43572 | B114.00 | Malignant neoplasm of body of stomach |
| 32022 | B110.00 | Malignant neoplasm of cardia of stomach |
| 37859 | B110z00 | Malignant neoplasm of cardia of stomach nos |
| 100584 | B110000 | Malignant neoplasm of cardiac orifice of stomach |
| 22894 | B110100 | Malignant neoplasm of cardio-oesophageal junction of stomach |
| 32362 | B113.00 | Malignant neoplasm of fundus of stomach |
| 94278 | B110111 | Malignant neoplasm of gastro-oesophageal junction |
| 55434 | B116.00 | Malignant neoplasm of greater curve of stomach unspecified |
| 42193 | B115.00 | Malignant neoplasm of lesser curve of stomach unspecified |
| 55019 | B11y.00 | Malignant neoplasm of other specified site of stomach |
| 65372 | B11yz00 | Malignant neoplasm of other specified site of stomach nos |
| 96802 | B11y100 | Malignant neoplasm of posterior wall of stomach nec |
| 48237 | B111000 | Malignant neoplasm of prepylorus of stomach |
| 19318 | B112.00 | Malignant neoplasm of pyloric antrum of stomach |
| 41215 | B111100 | Malignant neoplasm of pyloric canal of stomach |
| 21620 | B111.00 | Malignant neoplasm of pylorus of stomach |
| 59092 | B111z00 | Malignant neoplasm of pylorus of stomach nos |
| 8386 | B11..00 | Malignant neoplasm of stomach |
| 14800 | B11z.00 | Malignant neoplasm of stomach nos |
| 51690 | B117.00 | Malignant neoplasm, overlapping lesion of stomach |
| 97499 | B118.00 | Siewert type ii adenocarcinoma |
| 96094 | B119.00 | Siewert type iii adenocarcinoma |
| 28272 | BB57.00 | [M]adenocarcinoma, intestinal type |
| 59240 | BB58.00 | [M]carcinoma, diffuse type |
| 27440 | BB55.00 | [M]linitis plastica |
| 18613 | B120.00 | Malignant neoplasm of duodenum |
| 33871 | B122.00 | Malignant neoplasm of ileum |
| 43479 | B121.00 | Malignant neoplasm of jejunum |
| 63995 | B123.00 | Malignant neoplasm of meckel's diverticulum |
| 99896 | B12y.00 | Malignant neoplasm of other specified site small intestine |
| 6806 | B12..00 | Malignant neoplasm of small intestine and duodenum |
| 43390 | B12z.00 | Malignant neoplasm of small intestine nos |
| 66166 | B124.00 | Malignant neoplasm, overlapping lesion of small intestine |
| 5069 | PB0..00 | Meckel's diverticulum |
| 33781 | PB0z.00 | Meckel's diverticulum nos |
| 9491 | B142.11 | Anal carcinoma |
| 55659 | B14y.00 | Malig neop other site rectum, rectosigmoid junction and anus |
| 24370 | B142.00 | Malignant neoplasm of anal canal |
| 27897 | B143.00 | Malignant neoplasm of anus unspecified |
| 46159 | B142000 | Malignant neoplasm of cloacogenic zone |
| 35357 | B14..00 | Malignant neoplasm of rectum, rectosigmoid junction and anus |
| 50974 | B14z.00 | Malignant neoplasm rectum,rectosigmoid junction and anus nos |
| 38454 | BB48.00 | [M]basaloid carcinoma |
| 65216 | BB49.00 | [M]cloacogenic carcinoma |
| 31210 | B150100 | Hepatoblastoma of liver |
| 22187 | B150300 | Hepatocellular carcinoma |
| 65124 | B151000 | Malignant neoplasm of interlobular bile ducts |
| 110775 | B151100 | Malignant neoplasm of interlobular biliary canals |
| 16915 | B151.00 | Malignant neoplasm of intrahepatic bile ducts |
| 61643 | B151z00 | Malignant neoplasm of intrahepatic bile ducts nos |
| 89593 | B151200 | Malignant neoplasm of intrahepatic biliary passages |
| 58088 | B151400 | Malignant neoplasm of intrahepatic gall duct |
| 8918 | B15..00 | Malignant neoplasm of liver and intrahepatic bile ducts |
| 38978 | B15z.00 | Malignant neoplasm of liver and intrahepatic bile ducts nos |
| 26393 | B152.00 | Malignant neoplasm of liver unspecified |
| 68410 | B150200 | Primary angiosarcoma of liver |
| 16126 | B150000 | Primary carcinoma of liver |
| 25535 | B150.00 | Primary malignant neoplasm of liver |
| 44399 | B150z00 | Primary malignant neoplasm of liver nos |
| 41313 | BB5D300 | [M]bile duct cystadenocarcinoma |
| 8711 | BB5D100 | [M]cholangiocarcinoma |
| 107299 | BB5D700 | [M]combined hepatocellular carcinoma and cholangiocarcinoma |
| 106889 | BBL8.11 | [M]embryonal hepatoma |
| 57677 | BBL8.00 | [M]hepatoblastoma |
| 40240 | BB5D500 | [M]hepatocellular carcinoma nos |
| 46771 | BB5D800 | [M]hepatocellular carcinoma, fibrolamellar |
| 110147 | BB5D711 | [M]hepatocholangiocarcinoma |
| 26814 | BB5D512 | [M]hepatoma, malignant |
| 49900 | BB5y200 | [M]klatskin's tumour |
| 113013 | BBT5.00 | [M]kupffer cell sarcoma |
| 25641 | BB5D513 | [M]liver cell carcinoma |
| 113084 | Byu1000 | [X]other sarcomas of the liver |
| 43490 | Byu1100 | [X]other specified carcinomas of liver |
| 31393 | B160.11 | Carcinoma gallbladder |
| 16105 | B160.00 | Malignant neoplasm of gallbladder |
| 36495 | B161211 | Carcinoma common bile duct |
| 54103 | B16..00 | Malignant neoplasm gallbladder and extrahepatic bile ducts |
| 15907 | B16z.00 | Malignant neoplasm gallbladder/extrahepatic bile ducts nos |
| 10949 | B162.00 | Malignant neoplasm of ampulla of vater |
| 7982 | B161200 | Malignant neoplasm of common bile duct |
| 72445 | B161000 | Malignant neoplasm of cystic duct |
| 23433 | B161.00 | Malignant neoplasm of extrahepatic bile ducts |
| 74896 | B161z00 | Malignant neoplasm of extrahepatic bile ducts nos |
| 105613 | B161300 | Malignant neoplasm of sphincter of oddi |
| 60312 | B16y.00 | Malignant neoplasm other gallbladder/extrahepatic bile duct |
| 35039 | B163.00 | Malignant neoplasm, overlapping lesion of biliary tract |
| 40438 | BB5D111 | [M]bile duct carcinoma |
| 70516 | BB5D.11 | [M]biliary tract adenomas and adenocarcinomas |
| 40810 | B171.00 | Malignant neoplasm of body of pancreas |
| 96635 | B17y000 | Malignant neoplasm of ectopic pancreatic tissue |
| 8771 | B170.00 | Malignant neoplasm of head of pancreas |
| 52537 | B161100 | Malignant neoplasm of hepatic duct |
| 35795 | B174.00 | Malignant neoplasm of islets of langerhans |
| 48537 | B17y.00 | Malignant neoplasm of other specified sites of pancreas |
| 8166 | B17..00 | Malignant neoplasm of pancreas |
| 34388 | B17z.00 | Malignant neoplasm of pancreas nos |
| 35535 | B173.00 | Malignant neoplasm of pancreatic duct |
| 95783 | B17yz00 | Malignant neoplasm of specified site of pancreas nos |
| 39870 | B172.00 | Malignant neoplasm of tail of pancreas |
| 97875 | B175.00 | Malignant neoplasm, overlapping lesion of pancreas |
| 49629 | BB5C100 | [M]gastrinoma, malignant |
| 32294 | BB5B500 | [M]glucagonoma, malignant |
| 95609 | BB5B300 | [M]insulinoma, malignant |
| 63102 | BB5B100 | [M]islet cell carcinoma |
| 98825 | BB5B600 | [M]mixed islet cell and exocrine adenocarcinoma |
| 112383 | BBLK.00 | [M]pancreatoblastoma |
| 108667 | B1z1000 | Angiosarcoma of spleen |
| 94000 | 9Ow1.00 | Bowel cancer detected by national screening programme |
| 11628 | B1z0.11 | Cancer of bowel |
| 3357 | B1...11 | Carcinoma of digestive organs and peritoneum |
| 72224 | B1z1100 | Fibrosarcoma of spleen |
| 11009 | B1z..00 | Malig neop oth/ill-defined sites digestive tract/peritoneum |
| 15709 | B1...00 | Malignant neoplasm of digestive organs and peritoneum |
| 51255 | B1zz.00 | Malignant neoplasm of digestive tract and peritoneum nos |
| 17559 | B1z0.00 | Malignant neoplasm of intestinal tract, part unspecified |
| 65460 | B1z1.00 | Malignant neoplasm of spleen nec |
| 93778 | B1z1z00 | Malignant neoplasm of spleen nos |
| 56918 | B1zy.00 | Malignant neoplasm other spec digestive tract and peritoneum |
| 94776 | B1z2.00 | Malignant neoplasm, overlapping lesion of digestive system |
| 111172 | BB5R300 | [M]carcinoid tumour, argentaffin, malignant |
| 35180 | Byu1.00 | [X]malignant neoplasm of digestive organs |
| 45766 | Byu1200 | [X]malignant neoplasm of intestinal tract, part unspecified |
| 49292 | Byu1300 | [X]malignant neoplsm/ill-defin sites within digestive system |
| 24456 | B201.00 | Malig neop auditory tube, middle ear and mastoid air cells |
| 73537 | B201z00 | Malig neop auditory tube, middle ear, mastoid air cells nos |
| 107916 | B201000 | Malignant neoplasm of auditory (eustachian) tube |
| 71204 | B200000 | Malignant neoplasm of cartilage of nose |
| 71946 | B201300 | Malignant neoplasm of mastoid air cells |
| 95458 | B300300 | Malignant neoplasm of nasal bone |
| 23389 | B200.00 | Malignant neoplasm of nasal cavities |
| 42856 | B200z00 | Malignant neoplasm of nasal cavities nos |
| 98911 | B200100 | Malignant neoplasm of nasal conchae |
| 62761 | B200200 | Malignant neoplasm of septum of nose |
| 54613 | B201200 | Malignant neoplasm of tympanic antrum |
| 99386 | B073200 | Malignant neoplasm posterior margin nasal septum and choanae |
| 51878 | BBcC.00 | [M]aesthesioneuroblastoma |
| 39388 | BBcC.11 | [M]olfactory neuroblastoma |
| 106131 | BBcD.11 | [M]olfactory neuroepithelioma |
| 100111 | BB46.00 | [M]schneiderian carcinoma |
| 26652 | B20..00 | Malig neop nasal cavities, middle ear and accessory sinuses |
| 96971 | B20y.00 | Malig neop other site nasal cavity, middle ear and sinuses |
| 55246 | B20z.00 | Malignant neoplasm of accessory sinus nos |
| 54636 | B203.00 | Malignant neoplasm of ethmoid sinus |
| 15684 | B204.00 | Malignant neoplasm of frontal sinus |
| 17475 | B300A00 | Malignant neoplasm of maxilla |
| 32174 | B202.00 | Malignant neoplasm of maxillary sinus |
| 65215 | B205.00 | Malignant neoplasm of sphenoidal sinus |
| 39590 | B206.00 | Malignant neoplasm, overlapping lesion of accessory sinuses |
| 46728 | B064.00 | Malignant neoplasm of anterior epiglottis |
| 37805 | B213100 | Malignant neoplasm of cricoid cartilage |
| 107878 | B213200 | Malignant neoplasm of cuneiform cartilage |
| 318 | B210.00 | Malignant neoplasm of glottis |
| 43111 | B213.00 | Malignant neoplasm of laryngeal cartilage |
| 97332 | B213z00 | Malignant neoplasm of laryngeal cartilage nos |
| 319 | B21..00 | Malignant neoplasm of larynx |
| 9237 | B21z.00 | Malignant neoplasm of larynx nos |
| 26813 | B21y.00 | Malignant neoplasm of larynx, other specified site |
| 22441 | B212.00 | Malignant neoplasm of subglottis |
| 26165 | B211.00 | Malignant neoplasm of supraglottis |
| 47862 | B213300 | Malignant neoplasm of thyroid cartilage |
| 50579 | B214.00 | Malignant neoplasm, overlapping lesion of larynx |
| 103946 | B220100 | Malignant neoplasm of mucosa of trachea |
| 15221 | B220.00 | Malignant neoplasm of trachea |
| 37810 | B220z00 | Malignant neoplasm of trachea nos |
| 2587 | B22z.11 | Lung cancer |
| 3903 | B22z.00 | Malignant neoplasm of bronchus or lung nos |
| 17391 | B221000 | Malignant neoplasm of carina of bronchus |
| 33444 | B221100 | Malignant neoplasm of hilus of lung |
| 18678 | B224000 | Malignant neoplasm of lower lobe bronchus |
| 12582 | B224100 | Malignant neoplasm of lower lobe of lung |
| 31188 | B224.00 | Malignant neoplasm of lower lobe, bronchus or lung |
| 42566 | B224z00 | Malignant neoplasm of lower lobe, bronchus or lung nos |
| 12870 | B221.00 | Malignant neoplasm of main bronchus |
| 21698 | B221z00 | Malignant neoplasm of main bronchus nos |
| 41523 | B223000 | Malignant neoplasm of middle lobe bronchus |
| 39923 | B223100 | Malignant neoplasm of middle lobe of lung |
| 31268 | B223.00 | Malignant neoplasm of middle lobe, bronchus or lung |
| 54134 | B223z00 | Malignant neoplasm of middle lobe, bronchus or lung nos |
| 38961 | B22y.00 | Malignant neoplasm of other sites of bronchus or lung |
| 36371 | B225.00 | Malignant neoplasm of overlapping lesion of bronchus & lung |
| 13243 | B22..00 | Malignant neoplasm of trachea, bronchus and lung |
| 31700 | B222000 | Malignant neoplasm of upper lobe bronchus |
| 25886 | B222100 | Malignant neoplasm of upper lobe of lung |
| 10358 | B222.00 | Malignant neoplasm of upper lobe, bronchus or lung |
| 44169 | B222z00 | Malignant neoplasm of upper lobe, bronchus or lung nos |
| 20170 | B222.11 | Pancoast's syndrome |
| 57802 | BB5S400 | [M]alveolar adenocarcinoma |
| 36530 | BB5S211 | [M]alveolar cell carcinoma |
| 16723 | BB5S212 | [M]bronchiolar carcinoma |
| 34015 | BB5S200 | [M]bronchiolo-alveolar adenocarcinoma |
| 23081 | BB5R111 | [M]carcinoid bronchial adenoma |
| 60756 | BB5J.12 | [M]cylindroid bronchial adenoma |
| 106519 | BB1P.00 | [M]non-small cell carcinoma |
| 9156 | BB1K.00 | [M]oat cell carcinoma |
| 61082 | BBLA.11 | [M]pneumoblastoma |
| 48348 | BBLM.00 | [M]pulmonary blastoma |
| 67970 | BB1L.00 | [M]small cell carcinoma, fusiform cell type |
| 30988 | BB1M.00 | [M]small cell carcinoma, intermediate cell |
| 21217 | BB1N.00 | [M]small cell-large cell carcinoma |
| 40595 | Byu2000 | [X]malignant neoplasm of bronchus or lung, unspecified |
| 27483 | B240.00 | Malignant neoplasm of thymus |
| 49186 | BBbR.00 | [M]oligodendroglioma, anaplastic type |
| 59415 | BBB6100 | [M]thymoma, malignant |
| 39531 | B25..00 | Malig neo, overlapping lesion of heart, mediastinum & pleura |
| 27715 | B242.00 | Malignant neoplasm of anterior mediastinum |
| 63430 | B241000 | Malignant neoplasm of endocardium |
| 95644 | B241.00 | Malignant neoplasm of heart |
| 50289 | B241z00 | Malignant neoplasm of heart nos |
| 34878 | B308300 | Malignant neoplasm of medial cuneiform |
| 61064 | B24X.00 | Malignant neoplasm of mediastinum, part unspecified |
| 65605 | B241200 | Malignant neoplasm of myocardium |
| 98104 | B23y.00 | Malignant neoplasm of other specified pleura |
| 67107 | B230.00 | Malignant neoplasm of parietal pleura |
| 31573 | B23..00 | Malignant neoplasm of pleura |
| 34742 | B23z.00 | Malignant neoplasm of pleura nos |
| 92720 | B243.00 | Malignant neoplasm of posterior mediastinum |
| 106194 | B231.00 | Malignant neoplasm of visceral pleura |
| 50292 | Byu2500 | [X]malignant neoplasm of mediastinum, part unspecified |
| 66444 | Byu2100 | [X]malignant neoplasm/overlap lesion/heart,mediastinm+pleura |
| 45307 | B2...11 | Carcinoma of respiratory tract and intrathoracic organs |
| 100232 | B24y.00 | Malig neop of other site of heart, thymus and mediastinum |
| 34075 | B2...00 | Malig neop of respiratory tract and intrathoracic organs |
| 65793 | B2z0.00 | Malig neop of upper respiratory tract, part unspecified |
| 44356 | B2z..00 | Malig neop other/ill-defined sites resp/intrathoracic organs |
| 66750 | B24z.00 | Malignant neoplasm of heart, thymus and mediastinum nos |
| 97547 | B551200 | Malignant neoplasm of intrathoracic site nos |
| 29283 | B2zy.00 | Malignant neoplasm of other site of respiratory tract |
| 42569 | B2zz.00 | Malignant neoplasm of respiratory tract nos |
| 62556 | B24..00 | Malignant neoplasm of thymus, heart and mediastinum |
| 66646 | B26..00 | Malignant neoplasm, overlap lesion of resp & intrathor orgs |
| 99096 | Byu2300 | [X]malignant neopl/overlapping les/resp+intrathoracic organs |
| 35325 | Byu2.00 | [X]malignant neoplasm of respiratory and intrathoracic orga |
| 111904 | Byu2200 | [X]malignant neoplasm/upper resp tract, part unspecified |
| 65880 | B304z00 | Malig neop of scapula and long bones of upper arm nos |
| 105797 | B304100 | Malignant neoplasm of acromion |
| 72212 | B308200 | Malignant neoplasm of calcaneum |
| 69104 | B305100 | Malignant neoplasm of carpal bone - lunate |
| 57988 | B305000 | Malignant neoplasm of carpal bone - scaphoid |
| 106069 | B305.11 | Malignant neoplasm of carpal bones |
| 56513 | B307000 | Malignant neoplasm of femur |
| 50402 | B307100 | Malignant neoplasm of fibula |
| 94427 | B305C00 | Malignant neoplasm of fifth metacarpal bone |
| 110993 | B305800 | Malignant neoplasm of first metacarpal bone |
| 69927 | B308800 | Malignant neoplasm of first metatarsal bone |
| 92382 | B308B00 | Malignant neoplasm of fourth metatarsal bone |
| 73530 | B305.00 | Malignant neoplasm of hand bones |
| 73556 | B305z00 | Malignant neoplasm of hand bones nos |
| 61741 | B304200 | Malignant neoplasm of humerus |
| 68055 | B307.00 | Malignant neoplasm of long bones of leg |
| 62630 | B307z00 | Malignant neoplasm of long bones of leg nos |
| 72464 | B305.12 | Malignant neoplasm of metacarpal bones |
| 113608 | B308.11 | Malignant neoplasm of metatarsal bones of foot |
| 111779 | B308000 | Malignant neoplasm of patella |
| 58949 | B308D00 | Malignant neoplasm of phalanges of foot |
| 86812 | B305D00 | Malignant neoplasm of phalanges of hand |
| 92371 | B304300 | Malignant neoplasm of radius |
| 49054 | B304000 | Malignant neoplasm of scapula |
| 71810 | B304.00 | Malignant neoplasm of scapula and long bones of upper arm |
| 105475 | B308.00 | Malignant neoplasm of short bones of leg |
| 103354 | B308z00 | Malignant neoplasm of short bones of leg nos |
| 95182 | B308100 | Malignant neoplasm of talus |
| 108638 | B305A00 | Malignant neoplasm of third metacarpal bone |
| 40814 | B307200 | Malignant neoplasm of tibia |
| 64848 | B304400 | Malignant neoplasm of ulna |
| 43614 | B30X.00 | Malignant neoplasm/bones+articular cartilage/limb,unspfd |
| 38593 | BBY1.00 | [M]adamantinoma of long bones |
| 67430 | BBY1.11 | [M]tibial adamantinoma |
| 16075 | B30z.00 | Malignant neoplasm of bone and articular cartilage nos |
| 19437 | B30z000 | Osteosarcoma |
| 29337 | BBVA.00 | [M] small cell osteosarcoma |
| 100267 | BBZG.11 | [M]adamantinoma, malignant |
| 24539 | BBV2.00 | [M]chondroblastic osteosarcoma |
| 98559 | BBW8.00 | [M]chondroblastoma, malignant |
| 7941 | BBW4.00 | [M]chondrosarcoma nos |
| 49023 | BBY0.11 | [M]endothelial bone sarcoma |
| 4473 | BBY0.00 | [M]ewing's sarcoma |
| 21447 | BBV3.00 | [M]fibroblastic osteosarcoma |
| 68220 | BBW4.11 | [M]fibrochondrosarcoma |
| 50859 | BBX1.11 | [M]giant cell bone sarcoma |
| 68956 | BBX1.00 | [M]giant cell tumour of bone, malignant |
| 93175 | BBZ2.11 | [M]intraosseous carcinoma |
| 63659 | BBW6.00 | [M]juxtacortical chondrosarcoma |
| 99665 | BBV..11 | [M]juxtacortical osteogenic sarcoma |
| 49862 | BBV1.11 | [M]osteoblastic sarcoma |
| 59310 | BBV1.12 | [M]osteochondrosarcoma |
| 31673 | BBX1.12 | [M]osteoclastoma, malignant |
| 5052 | BBV1.13 | [M]osteogenic sarcoma nos |
| 60631 | BBV5.00 | [M]osteosarcoma in paget's disease of bone |
| 8660 | BBV1.00 | [M]osteosarcoma nos |
| 63571 | BBV..12 | [M]parosteal osteosarcoma |
| 22561 | BBV4.00 | [M]telangiectatic osteosarcoma |
| 40749 | Byu3.00 | [X]malignant neoplasm of bone and articular cartilage |
| 63460 | B213000 | Malignant neoplasm of arytenoid cartilage |
| 18314 | B30..00 | Malignant neoplasm of bone and articular cartilage |
| 59036 | B300.00 | Malignant neoplasm of bones of skull and face |
| 69146 | B300z00 | Malignant neoplasm of bones of skull and face nos |
| 46939 | B302000 | Malignant neoplasm of cervical vertebra |
| 66639 | B303200 | Malignant neoplasm of clavicle |
| 46905 | B545200 | Malignant neoplasm of coccygeal body |
| 66908 | B306400 | Malignant neoplasm of coccygeal vertebra |
| 60403 | B303300 | Malignant neoplasm of costal cartilage |
| 67763 | B303400 | Malignant neoplasm of costo-vertebral joint |
| 53594 | B300000 | Malignant neoplasm of ethmoid bone |
| 53599 | B300100 | Malignant neoplasm of frontal bone |
| 44609 | B306000 | Malignant neoplasm of ilium |
| 59223 | B306100 | Malignant neoplasm of ischium |
| 54691 | B302200 | Malignant neoplasm of lumbar vertebra |
| 59520 | B300200 | Malignant neoplasm of malar bone |
| 33833 | B301.00 | Malignant neoplasm of mandible |
| 55953 | B300400 | Malignant neoplasm of occipital bone |
| 50298 | B300500 | Malignant neoplasm of orbital bone |
| 54747 | B300600 | Malignant neoplasm of parietal bone |
| 54631 | B306.00 | Malignant neoplasm of pelvic bones, sacrum and coccyx |
| 38938 | B306z00 | Malignant neoplasm of pelvis, sacrum or coccyx nos |
| 51921 | B306200 | Malignant neoplasm of pubis |
| 37842 | B303000 | Malignant neoplasm of rib |
| 51237 | B303z00 | Malignant neoplasm of rib, sternum and clavicle nos |
| 27528 | B303.00 | Malignant neoplasm of ribs, sternum and clavicle |
| 40966 | B306300 | Malignant neoplasm of sacral vertebra |
| 111426 | B308900 | Malignant neoplasm of second metatarsal bone |
| 55595 | B300700 | Malignant neoplasm of sphenoid bone |
| 49491 | B303100 | Malignant neoplasm of sternum |
| 73510 | B550500 | Malignant neoplasm of supraclavicular fossa nos |
| 62104 | B300800 | Malignant neoplasm of temporal bone |
| 32372 | B302100 | Malignant neoplasm of thoracic vertebra |
| 96445 | B300B00 | Malignant neoplasm of turbinate |
| 16704 | B302.00 | Malignant neoplasm of vertebral column |
| 49701 | B302z00 | Malignant neoplasm of vertebral column nos |
| 44452 | B300C00 | Malignant neoplasm of vomer |
| 54493 | B303500 | Malignant neoplasm of xiphoid process |
| 50299 | B300900 | Malignant neoplasm of zygomatic bone |
| 108389 | B310500 | Malignant neoplasm soft tissues of cervical spine |
| 67451 | B30W.00 | Malignant neoplasm/overlap lesion/bone+articulr cartilage |
| 50152 | B306500 | Malignant sacral teratoma |
| 68730 | BBZN.00 | [M]ameloblastic fibrosarcoma |
| 46741 | BBZC.00 | [M]ameloblastic odontosarcoma |
| 97593 | BBZG.00 | [M]ameloblastoma, malignant |
| 4118 | BBV9.00 | [M]myxoid chondrosarcoma |
| 98483 | BBZN.11 | [M]odontogenic fibrosarcoma |
| 72443 | BBZ2.00 | [M]odontogenic tumour, malignant |
| 107884 | BBba000 | [M]peripheral neuroectodermal tumour |
| 43151 | Byu3300 | [X]malignant neoplasm/bone+articular cartilage, unspecified |
| 73296 | Byu3100 | [X]malignant neoplasm/bones+articular cartilage/limb,unspfd |
| 63300 | Byu3200 | [X]malignant neoplasm/overlap lesion/bone+articulr cartilage |
| 104609 | 4M71.00 | Clark melanoma level 2 |
| 96280 | 4M72.00 | Clark melanoma level 3 |
| 102116 | 4M73.00 | Clark melanoma level 4 |
| 108866 | 4M74.00 | Clark melanoma level 5 |
| 42714 | B327500 | Malignant melanoma of ankle |
| 59061 | B322000 | Malignant melanoma of auricle (ear) |
| 49814 | B325000 | Malignant melanoma of axilla |
| 43463 | B325700 | Malignant melanoma of back |
| 32768 | B325100 | Malignant melanoma of breast |
| 53629 | B325200 | Malignant melanoma of buttock |
| 51209 | B325800 | Malignant melanoma of chest wall |
| 71136 | B323100 | Malignant melanoma of chin |
| 57260 | B322.00 | Malignant melanoma of ear and external auricular canal |
| 73744 | B322z00 | Malignant melanoma of ear and external auricular canal nos |
| 102145 | B322100 | Malignant melanoma of external auditory meatus |
| 41278 | B323000 | Malignant melanoma of external surface of cheek |
| 45139 | B323400 | Malignant melanoma of external surface of nose |
| 108363 | B509.00 | Malignant melanoma of eye |
| 47094 | B323200 | Malignant melanoma of eyebrow |
| 54632 | B321.00 | Malignant melanoma of eyelid including canthus |
| 67806 | B323z00 | Malignant melanoma of face nos |
| 25602 | B326400 | Malignant melanoma of finger |
| 41490 | B327700 | Malignant melanoma of foot |
| 45755 | B326200 | Malignant melanoma of fore-arm |
| 68133 | B323300 | Malignant melanoma of forehead |
| 53369 | B327900 | Malignant melanoma of great toe |
| 34259 | B325300 | Malignant melanoma of groin |
| 62475 | B326300 | Malignant melanoma of hand |
| 61246 | B327600 | Malignant melanoma of heel |
| 73536 | B327000 | Malignant melanoma of hip |
| 54305 | B327200 | Malignant melanoma of knee |
| 70637 | B320.00 | Malignant melanoma of lip |
| 37872 | B327400 | Malignant melanoma of lower leg |
| 46255 | B327.00 | Malignant melanoma of lower limb and hip |
| 64327 | B327z00 | Malignant melanoma of lower limb or hip nos |
| 45306 | B324100 | Malignant melanoma of neck |
| 47252 | B323.00 | Malignant melanoma of other and unspecified parts of face |
| 42153 | B32y.00 | Malignant melanoma of other specified skin site |
| 109002 | B325400 | Malignant melanoma of perianal skin |
| 95629 | B325500 | Malignant melanoma of perineum |
| 39878 | B327300 | Malignant melanoma of popliteal fossa area |
| 55881 | B324000 | Malignant melanoma of scalp |
| 65625 | B324.00 | Malignant melanoma of scalp and neck |
| 99257 | B324z00 | Malignant melanoma of scalp and neck nos |
| 50505 | B326000 | Malignant melanoma of shoulder |
| 865 | B32..00 | Malignant melanoma of skin |
| 28556 | B32z.00 | Malignant melanoma of skin nos |
| 58958 | B323500 | Malignant melanoma of temple |
| 51873 | B327100 | Malignant melanoma of thigh |
| 63997 | B326500 | Malignant melanoma of thumb |
| 36899 | B327800 | Malignant melanoma of toe |
| 38689 | B325.00 | Malignant melanoma of trunk (excluding scrotum) |
| 45760 | B325z00 | Malignant melanoma of trunk, excluding scrotum, nos |
| 43715 | B325600 | Malignant melanoma of umbilicus |
| 54685 | B326100 | Malignant melanoma of upper arm |
| 65164 | B326.00 | Malignant melanoma of upper limb and shoulder |
| 55292 | B326z00 | Malignant melanoma of upper limb or shoulder nos |
| 111079 | B328.00 | Malignant melanoma stage ia |
| 109827 | B329.00 | Malignant melanoma stage ib |
| 110139 | B32A.00 | Malignant melanoma stage iia |
| 110180 | B32B.00 | Malignant melanoma stage iib |
| 111413 | B32C.00 | Malignant melanoma stage iic |
| 111672 | B32D.00 | Malignant melanoma stage iiia |
| 111943 | B32E.00 | Malignant melanoma stage iiib |
| 110961 | B32F.00 | Malignant melanoma stage iiic |
| 109745 | B32G.00 | Malignant melanoma stage iv m1a |
| 110483 | B32H.00 | Malignant melanoma stage iv m1b |
| 111162 | B32J.00 | Malignant melanoma stage iv m1c |
| 96585 | B32y000 | Overlapping malignant melanoma of skin |
| 22692 | BBEG000 | [M]acral lentiginous melanoma, malignant |
| 17232 | BBEA.00 | [M]amelanotic melanoma |
| 68889 | BBE4.00 | [M]balloon cell melanoma |
| 58835 | BBE1100 | [M]desmoplastic melanoma, malignant |
| 23085 | BBEP.00 | [M]epithelioid cell melanoma |
| 11922 | BBEG.11 | [M]lentigo maligna melanoma |
| 73251 | BBEM.00 | [M]malignant melanoma in giant pigmented naevus |
| 62088 | BBEG.00 | [M]malignant melanoma in hutchinson's melanotic freckle |
| 63574 | BBEC.00 | [M]malignant melanoma in junctional naevus |
| 113738 | BBEE.00 | [M]malignant melanoma in precancerous melanosis |
| 579 | BBE1.00 | [M]malignant melanoma nos |
| 51353 | BBE1000 | [M]malignant melanoma, regressing |
| 24551 | BBE1.11 | [M]melanocarcinoma |
| 44157 | BBE1.13 | [M]melanosarcoma nos |
| 67966 | BBE1.14 | [M]naevocarcinoma |
| 20982 | BBE2.00 | [M]nodular melanoma |
| 44061 | BBEQ.00 | [M]spindle cell melanoma nos |
| 24208 | BBEH.00 | [M]superficial spreading melanoma |
| 56925 | Byu4000 | [X]malignant melanoma of other+unspecified parts of face |
| 19444 | Byu4100 | [X]malignant melanoma of skin, unspecified |
| 104025 | B337600 | Malignant neoplasm of skin of heel |
| 19144 | Byu4.00 | [X]melanoma and other malignant neoplasms of skin |
| 94975 | B241300 | Malignant neoplasm of pericardium |
| 7484 | B226.00 | Mesothelioma |
| 101885 | B241400 | Mesothelioma of pericardium |
| 17874 | B181.00 | Mesothelioma of peritoneum |
| 9600 | B232.00 | Mesothelioma of pleura |
| 47734 | BBP5.00 | [M]epithelioid mesothelioma, malignant |
| 113440 | BBP3.00 | [M]fibrous mesothelioma, malignant |
| 86820 | BBP7.00 | [M]mesothelioma, biphasic type, malignant |
| 27509 | BBP1.00 | [M]mesothelioma, malignant |
| 21770 | BBPX.00 | [M]mesothelioma, unspecified |
| 104720 | BBP3.11 | [M]sarcomatoid mesothelioma |
| 21715 | Byu5011 | [X]mesothelioma of lung |
| 67034 | Byu5000 | [X]mesothelioma of other sites |
| 30526 | Byu5100 | [X]mesothelioma, unspecified |
| 27853 | A789500 | Hiv disease resulting in kaposi's sarcoma |
| 50290 | B6z0.00 | Kaposi's sarcoma of lymph nodes |
| 65466 | B592X00 | Kaposi's sarcoma of multiple organs |
| 37549 | B05z000 | Kaposi's sarcoma of palate |
| 27931 | B33z000 | Kaposi's sarcoma of skin |
| 104128 | B31z000 | Kaposi's sarcoma of soft tissue |
| 49525 | B59zX00 | Kaposi's sarcoma, unspecified |
| 27439 | BBTA.00 | [M]kaposi's sarcoma |
| 98361 | Byu5B00 | [X]kaposi's sarcoma of other sites |
| 93665 | Byu5300 | [X]kaposi's sarcoma, unspecified |
| 106654 | B524W00 | Mal neoplasm/periph nerves+autonomic nervous system,unspc |
| 24235 | B524.00 | Malig neopl peripheral nerves and autonomic nervous system |
| 86046 | B524400 | Malignant neoplasm of peripheral nerve of abdomen |
| 89258 | B524200 | Malignant neoplasm of peripheral nerve of low limb, incl hip |
| 73988 | B524500 | Malignant neoplasm of peripheral nerve of pelvis |
| 63695 | B524300 | Malignant neoplasm of peripheral nerve of thorax |
| 61716 | B524100 | Malignant neoplasm of peripheral nerve,upp limb,incl should |
| 63568 | B524000 | Malignant neoplasm of peripheral nerves of head, face & neck |
| 50777 | B524600 | Malignant neoplasm,overlap lesion periph nerve & auton ns |
| 105072 | Byu5500 | [X]mal neoplasm/overlap les/periph nerv+autonomic nerv systm |
| 86997 | Byu2400 | [X]malignant neoplasm/ill-defined sites within resp system |
| 101668 | Byu5400 | [X]malignant neoplasm/peripheral nerves of trunk,unspecified |
| 90290 | B18y700 | Malignant neoplasm of mesentery |
| 59388 | B18y100 | Malignant neoplasm of mesocaecum |
| 30165 | B18y200 | Malignant neoplasm of mesorectum |
| 50898 | B18y300 | Malignant neoplasm of omentum |
| 64516 | B18y400 | Malignant neoplasm of parietal peritoneum |
| 39413 | B18y500 | Malignant neoplasm of pelvic peritoneum |
| 65159 | B180100 | Malignant neoplasm of perinephric tissue |
| 24048 | B180200 | Malignant neoplasm of retrocaecal tissue |
| 21330 | B180.00 | Malignant neoplasm of retroperitoneum |
| 44108 | B18..00 | Malignant neoplasm of retroperitoneum and peritoneum |
| 16298 | B18z.00 | Malignant neoplasm of retroperitoneum and peritoneum nos |
| 61555 | B180z00 | Malignant neoplasm of retroperitoneum nos |
| 46613 | B18y.00 | Malignant neoplasm of specified parts of peritoneum |
| 64106 | B18yz00 | Malignant neoplasm of specified parts of peritoneum nos |
| 69821 | B18y600 | Malignant neoplasm of the pouch of douglas |
| 101907 | B182.00 | Overlapping malign lesion of retroperitoneum and peritoneum |
| 95671 | Byu5700 | [X]malignant neoplasm of peritoneum, unspecified |
| 73718 | B310z00 | Malig neop connective and soft tissue head, face, neck nos |
| 90546 | B312z00 | Malig neop connective and soft tissue hip and leg nos |
| 54965 | B312200 | Malig neop connective and soft tissue of popliteal space |
| 65233 | B31y.00 | Malig neop connective and soft tissue other specified site |
| 53989 | B311.00 | Malig neop connective and soft tissue upper limb/shoulder |
| 104913 | B311z00 | Malig neop connective soft tissue upper limb/shoulder nos |
| 43475 | B310.00 | Malig neop of connective and soft tissue head, face and neck |
| 60247 | B314z00 | Malig neop of connective and soft tissue of abdomen nos |
| 66488 | B314000 | Malig neop of connective and soft tissue of abdominal wall |
| 66088 | B312.00 | Malig neop of connective and soft tissue of hip and leg |
| 67324 | B315100 | Malig neop of connective and soft tissue of inguinal region |
| 30542 | B312300 | Malig neop of connective and soft tissue of lower leg |
| 58836 | B315z00 | Malig neop of connective and soft tissue of pelvis nos |
| 98408 | B313z00 | Malig neop of connective and soft tissue of thorax nos |
| 44805 | B312100 | Malig neop of connective and soft tissue thigh and upper leg |
| 57471 | B316.00 | Malig neop of connective and soft tissue trunk unspecified |
| 110192 | B315300 | Malig neopl of connective and soft tissue - sacrum or coccyx |
| 94272 | B314100 | Malig neoplasm of connective and soft tissues of lumb spine |
| 104139 | B313300 | Malig neoplasm of connective and soft tissues of thor spine |
| 34451 | B31..00 | Malignant neoplasm of connective and other soft tissue |
| 45071 | B314.00 | Malignant neoplasm of connective and soft tissue of abdomen |
| 29160 | B313000 | Malignant neoplasm of connective and soft tissue of axilla |
| 70463 | B315000 | Malignant neoplasm of connective and soft tissue of buttock |
| 91586 | B311400 | Malignant neoplasm of connective and soft tissue of finger |
| 54222 | B312400 | Malignant neoplasm of connective and soft tissue of foot |
| 57482 | B311200 | Malignant neoplasm of connective and soft tissue of fore-arm |
| 19321 | B311300 | Malignant neoplasm of connective and soft tissue of hand |
| 102949 | B312000 | Malignant neoplasm of connective and soft tissue of hip |
| 51965 | B315.00 | Malignant neoplasm of connective and soft tissue of pelvis |
| 59152 | B315200 | Malignant neoplasm of connective and soft tissue of perineum |
| 50222 | B311000 | Malignant neoplasm of connective and soft tissue of shoulder |
| 22290 | B313.00 | Malignant neoplasm of connective and soft tissue of thorax |
| 63988 | B311500 | Malignant neoplasm of connective and soft tissue of thumb |
| 99572 | B312500 | Malignant neoplasm of connective and soft tissue of toe |
| 15182 | B31z.00 | Malignant neoplasm of connective and soft tissue, site nos |
| 64345 | B311100 | Malignant neoplasm of connective and soft tissue, upper arm |
| 86996 | B501000 | Malignant neoplasm of connective tissue of orbit |
| 54186 | B313100 | Malignant neoplasm of diaphragm |
| 72522 | B313200 | Malignant neoplasm of great vessels |
| 55098 | B550000 | Malignant neoplasm of head nos |
| 40014 | B310100 | Malignant neoplasm of soft tissue of face |
| 59382 | B310000 | Malignant neoplasm of soft tissue of head |
| 48517 | B310200 | Malignant neoplasm of soft tissue of neck |
| 49463 | B310400 | Malignant neoplasm of tarsus of eyelid |
| 67217 | B55y100 | Malignant neoplasm of trunk nos |
| 111311 | B317.00 | Malignant neoplasm, overlap lesion connective & soft tissue |
| 42082 | BBK3700 | [M]alveolar rhabdomyosarcoma |
| 71869 | BBf2.00 | [M]alveolar soft part sarcoma |
| 63286 | BBN5.00 | [M]clear cell sarcoma of tendons and aponeuroses |
| 7856 | BBJH.00 | [M]dedifferentiated liposarcoma |
| 48275 | BBK3600 | [M]embryonal rhabdomyosarcoma |
| 98797 | BBLD.00 | [M]embryonal sarcoma |
| 105166 | BBDB.11 | [M]glomoid sarcoma |
| 95024 | BBG8.00 | [M]infantile fibrosarcoma |
| 28599 | BBJ1.00 | [M]liposarcoma nos |
| 105944 | BBK3300 | [M]mixed cell rhabdomyosarcoma |
| 56676 | BBJ5.00 | [M]myxoid liposarcoma |
| 55947 | BBJ7.00 | [M]pleomorphic liposarcoma |
| 103708 | BBJ6.00 | [M]round cell liposarcoma |
| 69844 | BBF5.11 | [M]round cell sarcoma |
| 58837 | BBF5.00 | [M]small cell sarcoma |
| 31026 | BBF3.00 | [M]spindle cell sarcoma |
| 50379 | BBN1.00 | [M]synovial sarcoma nos |
| 57796 | BBN4.00 | [M]synovial sarcoma, biphasic type |
| 105073 | BBN2.00 | [M]synovial sarcoma, spindle cell type |
| 91896 | Byu5800 | [X]mal neoplasm/connective+soft tissue of trunk,unspecified |
| 40592 | Byu5.00 | [X]malignant neoplasm of mesothelial and soft tissue |
| 91457 | Byu5900 | [X]malignant neoplasm/connective + soft tissue,unspecified |
| 348 | B34..11 | Ca female breast |
| 105488 | B36..00 | Local recurrence of malignant tumour of breast |
| 64686 | B340100 | Malignant neoplasm of areola of female breast |
| 67884 | B350100 | Malignant neoplasm of areola of male breast |
| 20685 | B346.00 | Malignant neoplasm of axillary tail of female breast |
| 31546 | B341.00 | Malignant neoplasm of central part of female breast |
| 95057 | B34y000 | Malignant neoplasm of ectopic site of female breast |
| 95323 | B35z000 | Malignant neoplasm of ectopic site of male breast |
| 3968 | B34..00 | Malignant neoplasm of female breast |
| 9470 | B34z.00 | Malignant neoplasm of female breast nos |
| 45222 | B343.00 | Malignant neoplasm of lower-inner quadrant of female breast |
| 42070 | B345.00 | Malignant neoplasm of lower-outer quadrant of female breast |
| 19423 | B35..00 | Malignant neoplasm of male breast |
| 48809 | B35zz00 | Malignant neoplasm of male breast nos |
| 26853 | B340.00 | Malignant neoplasm of nipple and areola of female breast |
| 54494 | B350.00 | Malignant neoplasm of nipple and areola of male breast |
| 23380 | B340000 | Malignant neoplasm of nipple of female breast |
| 68480 | B350000 | Malignant neoplasm of nipple of male breast |
| 59831 | B340z00 | Malignant neoplasm of nipple or areola of female breast nos |
| 56715 | B34y.00 | Malignant neoplasm of other site of female breast |
| 38475 | B34yz00 | Malignant neoplasm of other site of female breast nos |
| 54202 | B35z.00 | Malignant neoplasm of other site of male breast |
| 29826 | B342.00 | Malignant neoplasm of upper-inner quadrant of female breast |
| 23399 | B344.00 | Malignant neoplasm of upper-outer quadrant of female breast |
| 49148 | B347.00 | Malignant neoplasm, overlapping lesion of breast |
| 58131 | BB93.00 | [M]comedocarcinoma nos |
| 59251 | BBM9.00 | [M]cystosarcoma phyllodes, malignant |
| 39760 | BB91100 | [M]infiltrating duct and lobular carcinoma |
| 8351 | BB91.00 | [M]infiltrating duct carcinoma |
| 7319 | BB9G.00 | [M]infiltrating ductular carcinoma |
| 32472 | BB9H.00 | [M]inflammatory carcinoma |
| 3969 | BB9M.00 | [M]intracystic carcinoma nos |
| 30189 | BB91000 | [M]intraductal papillary adenocarcinoma with invasion |
| 40359 | BB94.00 | [M]juvenile breast carcinoma |
| 12427 | BB9F.00 | [M]lobular carcinoma nos |
| 98883 | BB9D.00 | [M]medullary carcinoma with lymphoid stroma |
| 42542 | BB9K.00 | [M]paget's disease and infiltrating breast duct carcinoma |
| 12480 | BB9K000 | [M]paget's disease and intraductal carcinoma of breast |
| 60803 | BB9J.11 | [M]paget's disease, breast |
| 12300 | BB9J.00 | [M]paget's disease, mammary |
| 67701 | BB94.11 | [M]secretory breast carcinoma |
| 12499 | Byu6.00 | [X]malignant neoplasm of breast |
| 53910 | B453.00 | Malignant neoplasm of clitoris |
| 47899 | B451000 | Malignant neoplasm of greater vestibular (bartholin's) gland |
| 58061 | B452.00 | Malignant neoplasm of labia minora |
| 27617 | B45y000 | Malignant neoplasm of overlapping lesion of vulva |
| 4554 | B454.00 | Malignant neoplasm of vulva unspecified |
| 11991 | B454.11 | Primary vulval cancer |
| 37328 | B450.00 | Malignant neoplasm of vagina |
| 60772 | B450z00 | Malignant neoplasm of vagina nos |
| 10698 | B450100 | Malignant neoplasm of vaginal vault |
| 3230 | B41..11 | Cervical carcinoma (uterus) |
| 107456 | 4K2M.00 | Crv smr - hi grade dyskaryosis? Invasive squamous carcinoma |
| 95505 | B41y000 | Malignant neoplasm of cervical stump |
| 2747 | B41..00 | Malignant neoplasm of cervix uteri |
| 28311 | B41z.00 | Malignant neoplasm of cervix uteri nos |
| 57235 | B410000 | Malignant neoplasm of endocervical canal |
| 53103 | B410100 | Malignant neoplasm of endocervical gland |
| 48820 | B410.00 | Malignant neoplasm of endocervix |
| 50285 | B410z00 | Malignant neoplasm of endocervix nos |
| 50297 | B411.00 | Malignant neoplasm of exocervix |
| 32955 | B41y.00 | Malignant neoplasm of other site of cervix |
| 43435 | B41yz00 | Malignant neoplasm of other site of cervix nos |
| 57719 | B41y100 | Malignant neoplasm of squamocolumnar junction of cervix |
| 58094 | B412.00 | Malignant neoplasm, overlapping lesion of cervix uteri |
| 33497 | BB2J.00 | [M]squamous cell carcinoma, microinvasive |
| 7046 | B43..00 | Malignant neoplasm of body of uterus |
| 33617 | B43z.00 | Malignant neoplasm of body of uterus nos |
| 72723 | B430000 | Malignant neoplasm of cornu of corpus uteri |
| 45490 | B430z00 | Malignant neoplasm of corpus uteri nos |
| 3213 | B430.00 | Malignant neoplasm of corpus uteri, excluding isthmus |
| 49400 | B430211 | Malignant neoplasm of endometrium |
| 2890 | B430200 | Malignant neoplasm of endometrium of corpus uteri |
| 68155 | B430100 | Malignant neoplasm of fundus of corpus uteri |
| 43940 | B431.00 | Malignant neoplasm of isthmus of uterine body |
| 70729 | B431z00 | Malignant neoplasm of isthmus of uterine body nos |
| 59097 | B431000 | Malignant neoplasm of lower uterine segment |
| 45793 | B430300 | Malignant neoplasm of myometrium of corpus uteri |
| 31608 | B43y.00 | Malignant neoplasm of other site of uterine body |
| 16967 | B432.00 | Malignant neoplasm of overlapping lesion of corpus uteri |
| 34030 | BBL0.00 | [M]endometrial stromal sarcoma |
| 21173 | BBL5.00 | [M]mullerian mixed tumour |
| 2744 | B40..00 | Malignant neoplasm of uterus, part unspecified |
| 48145 | D212000 | Anaemia in ovarian carcinoma |
| 1986 | B440.11 | Cancer of ovary |
| 7805 | B440.00 | Malignant neoplasm of ovary |
| 69978 | BB80200 | [M]borderline mucinous cystadenoma of the ovary |
| 70383 | BBM0100 | [M]brenner tumour, malignant |
| 103034 | BB5j500 | [M]endometrioid adenofibroma, malignant |
| 9447 | BB5j200 | [M]endometrioid carcinoma |
| 31609 | BBC4.00 | [M]granulosa cell tumour, malignant |
| 51656 | BB81E00 | [M]mucinous cystadenocarcinoma nos |
| 28396 | BB81D00 | [M]mucinous cystadenoma, borderline malignancy |
| 65051 | BB81500 | [M]papillary cystadenocarcinoma, nos |
| 98696 | BB81400 | [M]papillary cystadenoma, borderline malignancy |
| 46113 | BB81K00 | [M]papillary cystadenoma, borderline malignancy |
| 54749 | BB81H00 | [M]papillary mucinous cystadenocarcinoma |
| 44930 | BB81800 | [M]papillary serous cystadenocarcinoma |
| 6203 | BB81M00 | [M]papillary serous cystadenoma, borderline malignancy |
| 66876 | BB81E11 | [M]pseudomucinous adenocarcinoma |
| 38442 | BB81200 | [M]serous cystadenocarcinoma, nos |
| 52263 | BB81100 | [M]serous cystadenoma, borderline malignancy |
| 21131 | BB81J00 | [M]serous cystadenoma, borderline malignancy |
| 95150 | BB81B00 | [M]serous surface papillary carcinoma |
| 71301 | BBQA100 | [M]struma ovarii, malignant |
| 32191 | BBQ0.00 | [M]dysgerminoma |
| 37621 | BBQ4.00 | [M]endodermal sinus tumour |
| 37793 | 4M4..00 | Figo staging of gynaecological malignancy |
| 4555 | B45..00 | Malig neop of other and unspecified female genital organs |
| 101778 | B442.00 | Malignant neoplasm of broad ligament |
| 49828 | B441.00 | Malignant neoplasm of fallopian tube |
| 20166 | B45z.00 | Malignant neoplasm of female genital organ nos |
| 97996 | B44y.00 | Malignant neoplasm of other site of uterine adnexa |
| 95421 | B45y.00 | Malignant neoplasm of other specified female genital organ |
| 19141 | B44..00 | Malignant neoplasm of ovary and other uterine adnexa |
| 46153 | B443.00 | Malignant neoplasm of parametrium |
| 113479 | B444.00 | Malignant neoplasm of round ligament |
| 65106 | B44z.00 | Malignant neoplasm of uterine adnexa nos |
| 26454 | B45X.00 | Malignant neoplasm/overlapping lesion/feml genital organs |
| 55588 | Byu7300 | [X]malignant neoplasm of female genital organ, unspecified |
| 40598 | Byu7.00 | [X]malignant neoplasm of female genital organs |
| 64497 | Byu7000 | [X]malignant neoplasm of uterine adnexa, unspecified |
| 57756 | Byu7100 | [X]malignant neoplasm/other specified female genital organs |
| 16874 | B4...11 | Carcinoma of genitourinary organ |
| 20350 | BBQ4.14 | [M]yolk sac tumour |
| 28003 | B420.00 | Choriocarcinoma |
| 5136 | B911013 | Choriocarcinoma |
| 93762 | B42..00 | Malignant neoplasm of placenta |
| 67712 | BBR2.00 | [M]choriocarcinoma |
| 48743 | B482.00 | Malignant neoplasm of body of penis |
| 17841 | B481.00 | Malignant neoplasm of glans penis |
| 43392 | B483.00 | Malignant neoplasm of penis, part unspecified |
| 50681 | B480.00 | Malignant neoplasm of prepuce (foreskin) |
| 52570 | B487.00 | Malignant neoplasm, overlapping lesion of penis |
| 110384 | 8AD0.00 | Active surveillance of prostate cancer |
| 10178 | 4M0..00 | Gleason grading of prostate cancer |
| 18503 | 4M00.00 | Gleason prostate grade 2-4 (low) |
| 18612 | 4M01.00 | Gleason prostate grade 5-7 (medium) |
| 26081 | 4M02.00 | Gleason prostate grade 8-10 (high) |
| 780 | B46..00 | Malignant neoplasm of prostate |
| 19475 | B471.00 | Malignant neoplasm of descended testis |
| 91509 | B471z00 | Malignant neoplasm of descended testis nos |
| 15148 | B47..00 | Malignant neoplasm of testis |
| 38510 | B47z.00 | Malignant neoplasm of testis nos |
| 64602 | B470.00 | Malignant neoplasm of undescended testis |
| 96429 | B470z00 | Malignant neoplasm of undescended testis nos |
| 21786 | B471000 | Seminoma of descended testis |
| 2961 | B47z.11 | Seminoma of testis |
| 7740 | B470200 | Seminoma of undescended testis |
| 112307 | BBCC111 | [M]interstitial cell tumour, malignant |
| 95373 | BBCC100 | [M]leydig cell tumour, malignant |
| 29945 | BBR4.00 | [M]malignant teratoma, trophoblastic |
| 55658 | BBQ4.12 | [M]orchioblastoma |
| 9859 | BBQ1z00 | [M]seminoma nos |
| 57084 | BBQ1000 | [M]seminoma, anaplastic type |
| 7476 | BBQ1.00 | [M]seminomas |
| 29580 | BBCA.00 | [M]sertoli cell carcinoma |
| 35223 | BBQ1100 | [M]spermatocytic seminoma |
| 72127 | B484.00 | Malignant neoplasm of epididymis |
| 67949 | B48y.00 | Malignant neoplasm of other male genital organ |
| 92329 | B48yz00 | Malignant neoplasm of other male genital organ nos |
| 63224 | B48z.00 | Malignant neoplasm of penis and other male genital organ nos |
| 3541 | B48..00 | Malignant neoplasm of penis and other male genital organs |
| 47767 | B486.00 | Malignant neoplasm of scrotum |
| 68161 | B48y000 | Malignant neoplasm of seminal vesicle |
| 63331 | B485.00 | Malignant neoplasm of spermatic cord |
| 47668 | B48y100 | Malignant neoplasm of tunica vaginalis |
| 68824 | B48y200 | Malignant neoplasm, overlapping lesion male genital orgs |
| 45262 | Byu8200 | [X]malignant neoplasm of male genital organ, unspecified |
| 40671 | Byu8.00 | [X]malignant neoplasm of male genital organs |
| 57191 | Byu8000 | [X]malignant neoplasm/other specified male genital organs |
| 7978 | B4A0000 | Hypernephroma |
| 1599 | B4A0.00 | Malignant neoplasm of kidney parenchyma |
| 18712 | B4A..11 | Renal malignant neoplasm |
| 108922 | K01w112 | Wilms' tumour + nephrotic syndrome + pseudohermaphroditism |
| 112850 | BBJB100 | [M]angiomyoliposarcoma |
| 18771 | BBLJ.00 | [M]clear cell sarcoma of kidney |
| 52266 | BB5a011 | [M]grawitz tumour |
| 15419 | BB5a012 | [M]hypernephroma |
| 105862 | BBL7300 | [M]mesenchymal nephroblastoma |
| 21681 | BBL7100 | [M]nephroblastoma nos |
| 10668 | BB5a000 | [M]renal cell carcinoma |
| 17314 | BBL7112 | [M]wilms' tumour |
| 113076 | B180000 | Malignant neoplasm of periadrenal tissue |
| 27540 | B4A1000 | Malignant neoplasm of renal calyces |
| 12389 | B4A1.00 | Malignant neoplasm of renal pelvis |
| 54184 | B4A1z00 | Malignant neoplasm of renal pelvis nos |
| 101608 | B4A1100 | Malignant neoplasm of ureteropelvic junction |
| 15223 | B4A2.00 | Malignant neoplasm of ureter |
| 105388 | B498.00 | Local recurrence of malignant tumour of urinary bladder |
| 19162 | B493.00 | Malignant neoplasm of anterior wall of urinary bladder |
| 41571 | B495.00 | Malignant neoplasm of bladder neck |
| 44996 | B491.00 | Malignant neoplasm of dome of urinary bladder |
| 35963 | B492.00 | Malignant neoplasm of lateral wall of urinary bladder |
| 36949 | B49y.00 | Malignant neoplasm of other site of urinary bladder |
| 42012 | B494.00 | Malignant neoplasm of posterior wall of urinary bladder |
| 38862 | B490.00 | Malignant neoplasm of trigone of urinary bladder |
| 42023 | B497.00 | Malignant neoplasm of urachus |
| 28241 | B496.00 | Malignant neoplasm of ureteric orifice |
| 779 | B49..00 | Malignant neoplasm of urinary bladder |
| 31102 | B49z.00 | Malignant neoplasm of urinary bladder nos |
| 47801 | B49y000 | Malignant neoplasm, overlapping lesion of bladder |
| 13559 | B4A..00 | Malig neop of kidney and other unspecified urinary organs |
| 13252 | B4...00 | Malignant neoplasm of genitourinary organ |
| 52594 | B4z..00 | Malignant neoplasm of genitourinary organ nos |
| 38931 | B4y..00 | Malignant neoplasm of genitourinary organ os |
| 29462 | B4Az.00 | Malignant neoplasm of kidney or urinary organs nos |
| 44884 | B4Ay.00 | Malignant neoplasm of other urinary organs |
| 59286 | B4Ay000 | Malignant neoplasm of overlapping lesion of urinary organs |
| 72174 | B4A4.00 | Malignant neoplasm of paraurethral glands |
| 15644 | B4A3.00 | Malignant neoplasm of urethra |
| 101095 | BB4B.00 | [M]grade 1 (stage pta) papillary urothelial/transit cell ca |
| 102244 | BB4C.00 | [M]grade 2 (stage pta) papillary urothelial/transit cell ca |
| 101978 | BB4D.00 | [M]grade 3 (stage pta) papillary urothelial/transit cell ca |
| 45260 | Byu9000 | [X]malignant neoplasm of urinary organ, unspecified |
| 35113 | Byu9.00 | [X]malignant neoplasm of urinary tract |
| 98813 | B500.00 | Malig neop eyeball excl conjunctiva, cornea, retina, choroid |
| 15991 | B506.00 | Malignant neoplasm of choroid |
| 46789 | B515000 | Malignant neoplasm of choroid plexus |
| 59041 | B500000 | Malignant neoplasm of ciliary body |
| 63657 | B503.00 | Malignant neoplasm of conjunctiva |
| 73992 | B504.00 | Malignant neoplasm of cornea |
| 106569 | B500200 | Malignant neoplasm of crystalline lens |
| 112379 | B501100 | Malignant neoplasm of extraocular muscle of orbit |
| 20160 | B50..00 | Malignant neoplasm of eye |
| 54956 | B50z.00 | Malignant neoplasm of eye nos |
| 56718 | B500z00 | Malignant neoplasm of eyeball nos |
| 59381 | B500100 | Malignant neoplasm of iris |
| 71584 | B507.00 | Malignant neoplasm of lacrimal duct |
| 64817 | B502.00 | Malignant neoplasm of lacrimal gland |
| 101805 | B507000 | Malignant neoplasm of lacrimal sac |
| 65357 | B507100 | Malignant neoplasm of nasolacrimal duct |
| 45667 | B501.00 | Malignant neoplasm of orbit |
| 63104 | B501z00 | Malignant neoplasm of orbit nos |
| 40437 | B50y.00 | Malignant neoplasm of other specified site of eye |
| 28069 | B505.00 | Malignant neoplasm of retina |
| 45922 | B508.00 | Malignant neoplasm, overlapping lesion of eye and adnexa |
| 48952 | BBc9z00 | [M]retinoblastoma nos |
| 112174 | BBc9000 | [M]retinoblastoma, differentiated type |
| 103883 | BBc9100 | [M]retinoblastoma, undifferentiated type |
| 28836 | BBc9.00 | [M]retinoblastomas |
| 92293 | BBES.00 | [M]spindle cell melanoma, type b |
| 110766 | B521000 | Malignant neoplasm of cerebral dura mater |
| 28919 | B521.00 | Malignant neoplasm of cerebral meninges |
| 70104 | B521z00 | Malignant neoplasm of cerebral meninges nos |
| 109473 | B521200 | Malignant neoplasm of cerebral pia mater |
| 49875 | B52X.00 | Malignant neoplasm of meninges, unspecified |
| 49714 | B523.00 | Malignant neoplasm of spinal meninges |
| 67211 | B523z00 | Malignant neoplasm of spinal meninges nos |
| 60347 | BBd2.11 | [M]leptomeningeal sarcoma |
| 106134 | BBdB.00 | [M]meningeal sarcomatosis |
| 27363 | BBd2.00 | [M]meningioma, malignant |
| 96798 | BBd2.12 | [M]meningothelial sarcoma |
| 63925 | ByuA200 | [X]malignant neoplasm of meninges, unspecified |
| 10851 | B51..11 | Cerebral tumour - malignant |
| 53504 | B52W.00 | Malig neopl, overlap lesion brain & other part of cns |
| 15711 | B510.00 | Malignant neoplasm cerebrum (excluding lobes and ventricles) |
| 48073 | B510000 | Malignant neoplasm of basal ganglia |
| 18617 | B51..00 | Malignant neoplasm of brain |
| 41520 | B51z.00 | Malignant neoplasm of brain nos |
| 44089 | B517.00 | Malignant neoplasm of brain stem |
| 68641 | B517z00 | Malignant neoplasm of brain stem nos |
| 45154 | B516.00 | Malignant neoplasm of cerebellum |
| 61399 | B510100 | Malignant neoplasm of cerebral cortex |
| 64557 | B517000 | Malignant neoplasm of cerebral peduncle |
| 112831 | B515z00 | Malignant neoplasm of cerebral ventricle nos |
| 52511 | B515.00 | Malignant neoplasm of cerebral ventricles |
| 54133 | B510z00 | Malignant neoplasm of cerebrum nos |
| 59170 | B51y000 | Malignant neoplasm of corpus callosum |
| 42426 | B511.00 | Malignant neoplasm of frontal lobe |
| 99913 | B510300 | Malignant neoplasm of globus pallidus |
| 70942 | B510400 | Malignant neoplasm of hypothalamus |
| 93537 | B517200 | Malignant neoplasm of midbrain |
| 39088 | B514.00 | Malignant neoplasm of occipital lobe |
| 100733 | B51yz00 | Malignant neoplasm of other part of brain nos |
| 71139 | B51y.00 | Malignant neoplasm of other parts of brain |
| 19226 | B513.00 | Malignant neoplasm of parietal lobe |
| 91240 | B517300 | Malignant neoplasm of pons |
| 46792 | B512.00 | Malignant neoplasm of temporal lobe |
| 47556 | B512z00 | Malignant neoplasm of temporal lobe nos |
| 62126 | B510500 | Malignant neoplasm of thalamus |
| 65241 | B51y200 | Malignant neoplasm, overlapping lesion of brain |
| 50235 | BBbK.00 | [M]astroblastoma |
| 27748 | BBbB.11 | [M]astrocytic glioma |
| 8547 | BBbB.00 | [M]astrocytoma nos |
| 8328 | BBbC.00 | [M]astrocytoma, anaplastic type |
| 37473 | BBbW.00 | [M]cerebellar sarcoma nos |
| 65952 | BBbU.00 | [M]desmoplastic medulloblastoma |
| 46769 | BBb8.11 | [M]ependymoblastoma |
| 20084 | BBb7.00 | [M]ependymoma nos |
| 52751 | BBb8.00 | [M]ependymoma, anaplastic type |
| 27846 | BBbF.00 | [M]fibrillary astrocytoma |
| 45531 | BBbE.00 | [M]gemistocytic astrocytoma |
| 66064 | BBbM.00 | [M]giant cell glioblastoma |
| 9575 | BBbL.11 | [M]glioblastoma multiforme |
| 23083 | BBbL.00 | [M]glioblastoma nos |
| 8523 | BBb0.11 | [M]glioma nos |
| 27653 | BBbz.00 | [M]glioma nos |
| 31574 | BBb0.00 | [M]glioma, malignant |
| 12309 | BBb..00 | [M]gliomas |
| 38551 | BBb1.00 | [M]gliomatosis cerebri |
| 34252 | BBb0.12 | [M]gliosarcoma |
| 61783 | BBbG.11 | [M]juvenile astrocytoma |
| 34763 | BBbT.00 | [M]medulloblastoma nos |
| 31767 | BBbV.00 | [M]medullomyoblastoma |
| 39386 | BBb2.11 | [M]mixed glioma |
| 68808 | BBb2.00 | [M]mixed glioma |
| 46404 | BBbS.00 | [M]oligodendroblastoma |
| 27744 | BBbQ.00 | [M]oligodendroglioma nos |
| 30273 | BBbG.00 | [M]pilocytic astrocytoma |
| 98800 | BBbG.12 | [M]piloid astrocytoma |
| 67587 | BBbZ.00 | [M]pleomorphic xanthoastrocytoma |
| 41695 | BBba.00 | [M]primitive neuroectodermal tumour |
| 113678 | BBbD.00 | [M]protoplasmic astrocytoma |
| 103047 | BBbH.00 | [M]spongioblastoma nos |
| 113660 | BBbJ.00 | [M]spongioblastoma polare |
| 65458 | B52..00 | Malig neop of other and unspecified parts of nervous system |
| 65599 | B520200 | Malignant neoplasm of acoustic nerve |
| 9622 | B525.00 | Malignant neoplasm of cauda equina |
| 99621 | B520.00 | Malignant neoplasm of cranial nerves |
| 101086 | B520z00 | Malignant neoplasm of cranial nerves nos |
| 56490 | B52z.00 | Malignant neoplasm of nervous system nos |
| 64971 | B520000 | Malignant neoplasm of olfactory bulb |
| 70126 | B520100 | Malignant neoplasm of optic nerve |
| 88144 | B52y.00 | Malignant neoplasm of other specified part of nervous system |
| 51115 | B522.00 | Malignant neoplasm of spinal cord |
| 113721 | B523000 | Malignant neoplasm of spinal dura mater |
| 69981 | BBe7.00 | [M]neurilemmoma, malignant |
| 47633 | ByuA300 | [X]malig neopl, overlap lesion brain & other part of cns |
| 35285 | ByuA.00 | [X]malignant neoplasm of eye, brain and other parts of cent |
| 41515 | ByuA100 | [X]malignant neoplasm/central nervous system, unspecified |
| 68027 | ByuA000 | [X]malignant neoplasm/other and unspecified cranial nerves |
| 5637 | B53..00 | Malignant neoplasm of thyroid gland |
| 47920 | BB9B.11 | [M]c cell carcinoma |
| 21741 | BB5f100 | [M]follicular adenocarcinoma nos |
| 61467 | BB5f300 | [M]follicular adenocarcinoma, trabecular type |
| 59918 | BB5f200 | [M]follicular adenocarcinoma, well differentiated type |
| 21847 | BB5f111 | [M]follicular carcinoma |
| 50946 | BB9C.00 | [M]medullary carcinoma with amyloid stroma |
| 68757 | BB5f700 | [M]nonencapsulated sclerosing carcinoma |
| 46761 | BB5f600 | [M]papillary and follicular adenocarcinoma |
| 113642 | BB9B.12 | [M]parafollicular cell carcinoma |
| 112200 | BB9C.11 | [M]solid carcinoma with amyloid stroma |
| 61390 | B540000 | Malignant neoplasm of adrenal cortex |
| 28148 | B540.00 | Malignant neoplasm of adrenal gland |
| 70824 | B540z00 | Malignant neoplasm of adrenal gland nos |
| 94220 | B540100 | Malignant neoplasm of adrenal medulla |
| 49132 | B517100 | Malignant neoplasm of medulla oblongata |
| 60775 | BB5h100 | [M]adrenal cortical carcinoma |
| 2123 | BBc1.00 | [M]neuroblastoma nos |
| 106613 | BBDA.11 | [M]phaeochromoblastoma |
| 65047 | BBDA.00 | [M]phaeochromocytoma, malignant |
| 64195 | B54z.00 | Malig neop of endocrine gland or related structure nos |
| 30511 | B54..00 | Malig neop of other endocrine glands and related structures |
| 59718 | B542z00 | Malig neop pituitary gland or craniopharyngeal duct nos |
| 47840 | B545100 | Malignant neoplasm of aortic body |
| 50035 | B545.00 | Malignant neoplasm of aortic body and other paraganglia |
| 103995 | B545z00 | Malignant neoplasm of aortic body or paraganglia nos |
| 57047 | B544.00 | Malignant neoplasm of carotid body |
| 39899 | B542100 | Malignant neoplasm of craniopharyngeal duct |
| 51795 | B545000 | Malignant neoplasm of glomus jugulare |
| 90659 | B54y.00 | Malignant neoplasm of other specified endocrine gland |
| 4218 | B541.00 | Malignant neoplasm of parathyroid gland |
| 42460 | B543.00 | Malignant neoplasm of pineal gland |
| 8550 | B542000 | Malignant neoplasm of pituitary gland |
| 59823 | B542.00 | Malignant neoplasm pituitary gland and craniopharyngeal duct |
| 87113 | B54X.00 | Malignant neoplasm-pluriglandular involvement,unspecified |
| 72277 | BB5V700 | [M]basophil carcinoma |
| 68456 | BB5V100 | [M]chromophobe carcinoma |
| 36876 | BB5V311 | [M]eosinophil carcinoma |
| 40622 | BB5V711 | [M]mucoid cell carcinoma |
| 50151 | BBa3.00 | [M]pineoblastoma |
| 112235 | BB5c200 | [M]water-clear cell adenocarcinoma |
| 64309 | ByuB100 | [X]malignant neoplasm of endocrine gland, unspecified |
| 40608 | ByuB.00 | [X]malignant neoplasm of thyroid and other endocrine glands |
| 9902 | B3...11 | Carcinoma of bone, connective tissue, skin and breast |
| 18608 | B3...00 | Malig neop of bone, connective tissue, skin and breast |
| 41011 | B3z..00 | Malig neop of bone, connective tissue, skin and breast nos |
| 19389 | B3y..00 | Malig neop of bone, connective tissue, skin and breast os |
| 15976 | B552.00 | Malignant neoplasm of abdomen |
| 68787 | B55y000 | Malignant neoplasm of back nos |
| 23861 | B551100 | Malignant neoplasm of chest wall nos |
| 94355 | B55y200 | Malignant neoplasm of flank nos |
| 68236 | B550.00 | Malignant neoplasm of head, neck and face |
| 58903 | B550z00 | Malignant neoplasm of head, neck and face nos |
| 51818 | B550300 | Malignant neoplasm of jaw nos |
| 31399 | B555.00 | Malignant neoplasm of lower limb nos |
| 16280 | B550400 | Malignant neoplasm of neck nos |
| 12490 | B550200 | Malignant neoplasm of nose nos |
| 45267 | B55z.00 | Malignant neoplasm of other and ill defined site nos |
| 9030 | B55..00 | Malignant neoplasm of other and ill-defined sites |
| 52316 | B553.00 | Malignant neoplasm of pelvis |
| 55101 | B553z00 | Malignant neoplasm of pelvis nos |
| 107126 | B553200 | Malignant neoplasm of sacrococcygeal region |
| 47286 | B551.00 | Malignant neoplasm of thorax |
| 64810 | B551z00 | Malignant neoplasm of thorax nos |
| 27449 | B554.00 | Malignant neoplasm of upper limb nos |
| 12539 | B3...12 | Sarcoma of bone and connective tissue |
| 96226 | ByuC100 | [X]malignant neoplasm/overlap lesion/other+ill-defined sites |
| 26197 | 8B3p.00 | Administration of cancer treatment |
| 10335 | 1O0..00 | Cancer confirmed |
| 10292 | 8CL0.00 | Cancer diagnosis discussed |
| 22382 | 8CL2.00 | Cancer diagnosis discussed with patient |
| 11075 | 8CL1.00 | Cancer diagnosis discussed with significant other |
| 54336 | 8BCF.00 | Cancer hospital treatment completed |
| 26076 | 9Ok5.00 | Cancer pain and symptom management |
| 59054 | 9Ok7.00 | Cancer rehabilitation and readaption |
| 32411 | 8BC6.00 | Cancer treatment started |
| 8693 | B5...11 | Carcinoma of other and unspecified sites |
| 6170 | B590.11 | Carcinomatosis |
| 44952 | 9Ok3.00 | Date cancer diagnosis received in primary care |
| 105324 | A789800 | Hiv disease resulting in multiple malignant neoplasms |
| 67575 | A788W00 | Hiv disease resulting in unspecified malignant neoplasm |
| 101836 | A788600 | Human immunodeficiency virus with secondary cancers |
| 30283 | 7B2C700 | Intravesical install chemotherapeutic agent for malignancy |
| 37618 | B551000 | Malignant neoplasm of axilla nos |
| 57854 | B553000 | Malignant neoplasm of inguinal region nos |
| 1056 | B5z..00 | Malignant neoplasm of other and unspecified site nos |
| 38736 | B5y..00 | Malignant neoplasm of other and unspecified site os |
| 10995 | B5...00 | Malignant neoplasm of other and unspecified sites |
| 42218 | B55y.00 | Malignant neoplasm of other specified sites |
| 60052 | B55yz00 | Malignant neoplasm of specified site nos |
| 47810 | B59..00 | Malignant neoplasm of unspecified site |
| 54267 | B59z.00 | Malignant neoplasm of unspecified site nos |
| 104324 | B595.00 | Malignant tumour of unknown origin |
| 104684 | B76D.00 | Multiple self-healing epithelioma of ferguson-smith |
| 57551 | F381100 | Myasthenic syndrome due to other malignancy |
| 49482 | F396200 | Myopathy due to malignant disease |
| 100083 | B546.00 | Neuroblastoma |
| 26034 | B591.00 | Other malignant neoplasm nos |
| 30537 | F373.00 | Polyneuropathy in malignant disease |
| 11035 | B593.00 | Primary malignant neoplasm of unknown site |
| 32351 | 44a4.00 | Squamous cell carcinoma antigen level |
| 37688 | BBA2.00 | [M]acinar cell carcinoma |
| 8524 | BBB2.11 | [M]adenoacanthoma |
| 56794 | BB5R800 | [M]adenocarcinoid tumour |
| 52326 | BB5L100 | [M]adenocarcinoma in adenomatous polyp |
| 73434 | BB5L300 | [M]adenocarcinoma in multiple adenomatous polyps |
| 44778 | BB52000 | [M]adenocarcinoma in tubulovillous adenoma |
| 67342 | BB5U100 | [M]adenocarcinoma in villous adenoma |
| 8930 | BB52.00 | [M]adenocarcinoma nos |
| 66000 | BBB5.00 | [M]adenocarcinoma with apocrine metaplasia |
| 42553 | BBB3.00 | [M]adenocarcinoma with cartilaginous and osseous metaplasia |
| 94810 | BBB4.00 | [M]adenocarcinoma with spindle cell metaplasia |
| 16146 | BBB2.00 | [M]adenocarcinoma with squamous metaplasia |
| 2272 | BB5..11 | [M]adenocarcinomas |
| 33775 | BB5J.00 | [M]adenoid cystic carcinoma |
| 31004 | BB2G.00 | [M]adenoid squamous cell carcinoma |
| 63518 | BBLE.00 | [M]adenosarcoma |
| 36870 | BBL7111 | [M]adenosarcoma |
| 12580 | BBB0.00 | [M]adenosquamous carcinoma |
| 67019 | BBK1100 | [M]angiomyosarcoma |
| 22650 | BBT1.11 | [M]angiosarcoma |
| 34110 | BB5R100 | [M]carcinoid tumour, malignant |
| 100625 | BB5R500 | [M]carcinoid tumour, nonargentaffin, malignant |
| 8695 | BB12.00 | [M]carcinoma nos |
| 12609 | BB19.00 | [M]carcinoma, anaplastic type, nos |
| 21609 | BB18.00 | [M]carcinoma, undifferentiated type, nos |
| 16692 | BB14.00 | [M]carcinomatosis |
| 19334 | BBL9.00 | [M]carcinosarcoma nos |
| 67934 | BBLA.00 | [M]carcinosarcoma, embryonal type |
| 21758 | BBa5.00 | [M]chordoma |
| 54627 | BBR3.00 | [M]choriocarcinoma combined with teratoma |
| 37354 | BB5X100 | [M]clear cell adenocarcinoma nos |
| 30416 | BB82111 | [M]colloid adenocarcinoma |
| 94286 | BBG8.11 | [M]congenital fibrosarcoma |
| 50140 | BB5K.00 | [M]cribriform carcinoma |
| 34879 | BB5J.11 | [M]cylindroid adenocarcinoma |
| 34000 | BB80100 | [M]cystadenocarcinoma nos |
| 65861 | BBQ9.00 | [M]dermoid cyst with malignant transformation |
| 21833 | BB91.11 | [M]duct carcinoma nos |
| 28941 | BBQ3.00 | [M]embryonal carcinoma nos |
| 57087 | BBQ7211 | [M]embryonal teratoma |
| 56600 | BB2A.11 | [M]epidermoid carcinoma nos |
| 57513 | BB2C.11 | [M]epidermoid carcinoma, keratinising type |
| 38770 | BBB7.00 | [M]epithelial-myoepithelial carcinoma |
| 62396 | BBF6.00 | [M]epithelioid cell sarcoma |
| 38481 | BBTK.00 | [M]epithelioid haemangioendothelioma, malignant |
| 73916 | BBK0400 | [M]epithelioid leiomyosarcoma |
| 57336 | BB16.00 | [M]epithelioma, malignant |
| 101923 | BBJ1.11 | [M]fibroliposarcoma |
| 8088 | BBG3.00 | [M]fibromyxosarcoma |
| 31323 | BBG1.00 | [M]fibrosarcoma nos |
| 37680 | BBGF.00 | [M]fibrous histiocytoma, malignant |
| 96231 | BBGJ.00 | [M]fibroxanthoma, malignant |
| 35034 | BBGJ.11 | [M]fibroxanthosarcoma |
| 113899 | BB5C111 | [M]g cell tumour, malignant |
| 39121 | BBc0100 | [M]ganglioneuroblastoma |
| 95008 | BB82112 | [M]gelatinous adenocarcinoma |
| 27971 | BBQ2.00 | [M]germinoma |
| 48048 | BB1B.00 | [M]giant cell and spindle cell carcinoma |
| 35474 | BB1C.00 | [M]giant cell carcinoma |
| 97463 | BBF4.00 | [M]giant cell sarcoma (except of bone) |
| 50605 | BBDB.00 | [M]glomangiosarcoma |
| 69210 | BB5R611 | [M]goblet cell tumour |
| 34096 | BB5b.00 | [M]granular cell carcinoma |
| 98322 | BBT7100 | [M]haemangioendothelioma, malignant |
| 113118 | BBTD.00 | [M]haemangiopericytic neoplasms |
| 105296 | BBTD200 | [M]haemangiopericytoma, malignant |
| 62348 | BBT1.00 | [M]haemangiosarcoma |
| 29008 | BB5W111 | [M]hurthle cell adenocarcinoma |
| 43865 | BBQ7212 | [M]immature teratoma |
| 25961 | BB17.00 | [M]large cell carcinoma nos |
| 10588 | BBK0200 | [M]leiomyosarcoma nos |
| 28628 | BBJ3.00 | [M]liposarcoma, well differentiated type |
| 57729 | BBU1.00 | [M]lymphangiosarcoma |
| 45510 | BB2M.00 | [M]lymphoepithelial carcinoma |
| 99797 | BBX3.00 | [M]malignant giant cell tumour of soft parts |
| 21682 | BBQ7500 | [M]malignant teratoma, intermediate type |
| 61542 | BBQ7400 | [M]malignant teratoma, undifferentiated type |
| 32213 | BB0A.00 | [M]malignant tumour, fusiform cell type |
| 24511 | BB09.00 | [M]malignant tumour, giant cell type |
| 22156 | BB08.00 | [M]malignant tumour, small cell type |
| 16677 | BB9B.00 | [M]medullary carcinoma nos |
| 67288 | BBc2.00 | [M]medulloepithelioma nos |
| 52684 | BBW9.00 | [M]mesenchymal chondrosarcoma |
| 87003 | BBLC100 | [M]mesenchymoma, malignant |
| 49811 | BBL6.00 | [M]mesodermal mixed tumour |
| 40303 | BBET.00 | [M]mixed epithelioid and spindle melanoma |
| 35071 | BBQB.00 | [M]mixed germ cell tumour |
| 66607 | BBL4.00 | [M]mixed tumour, malignant, nos |
| 59651 | BBJ8.00 | [M]mixed type liposarcoma |
| 44074 | BB84.00 | [M]mucin-producing adenocarcinoma |
| 12497 | BB82100 | [M]mucinous adenocarcinoma |
| 28625 | BB71.00 | [M]mucoepidermoid carcinoma |
| 55429 | BB82113 | [M]mucoid adenocarcionoma |
| 59284 | BB82114 | [M]mucous adenocarcinoma |
| 55268 | BBK2100 | [M]myosarcoma |
| 64596 | BBK0700 | [M]myxoid leiomyosarcoma |
| 60127 | BBJ5.12 | [M]myxoliposarcoma |
| 21732 | BBH1.00 | [M]myxosarcoma |
| 22267 | BB04.00 | [M]neoplasm, malig, uncertain whether primary or metastatic |
| 21868 | BB02.00 | [M]neoplasm, malignant |
| 26253 | BB5R900 | [M]neuroendocrine carcinoma |
| 107281 | BBz0.00 | [M]neuroendocrine neoplasm |
| 97961 | BBc4.00 | [M]neuroepithelioma nos |
| 99491 | BBcz.00 | [M]neuroepitheliomatous neoplasm nos |
| 54284 | BBc..00 | [M]neuroepitheliomatous neoplasms |
| 62941 | BBe2.00 | [M]neurofibrosarcoma |
| 58902 | BBcA.00 | [M]olfactory neurogenic tumour |
| 53129 | BB5W112 | [M]oncytic adenocarcinoma |
| 71497 | BB5W100 | [M]oxyphilic adenocarcinoma |
| 24523 | BB9L.00 | [M]paget's disease, extramammary, exc paget's disease bone |
| 35348 | BB5T100 | [M]papillary adenocarcinoma nos |
| 10541 | BB22.00 | [M]papillary carcinoma nos |
| 67912 | BB26.11 | [M]papillary epidermoid carcinoma |
| 20807 | BB26.00 | [M]papillary squamous cell carcinoma |
| 9712 | BB4A.00 | [M]papillary transitional cell carcinoma |
| 95818 | BBD1.00 | [M]paraganglioma, malignant |
| 105275 | BBV..13 | [M]periosteal osteogenic sarcoma |
| 31090 | BBGP.00 | [M]pigmented dermatofibrosarcoma protuberans |
| 26413 | BB1A.00 | [M]pleomorphic carcinoma |
| 46581 | BBF4.11 | [M]pleomorphic cell sarcoma |
| 57505 | BBK3200 | [M]pleomorphic rhabdomyosarcoma |
| 102356 | BBQ5.00 | [M]polyembryoma |
| 69300 | BB1F.00 | [M]polygonal cell carcinoma |
| 54276 | BB1E.00 | [M]pseudosarcomatous carcinoma |
| 17212 | BBLH.00 | [M]rhabdoid sarcoma |
| 31421 | BBK3100 | [M]rhabdomyosarcoma nos |
| 66541 | BB1J.12 | [M]round cell carcinoma |
| 63247 | BBK3611 | [M]sarcoma botryoides |
| 8085 | BBF1.00 | [M]sarcoma nos |
| 37477 | BBe7.11 | [M]schwannoma, malignant |
| 48223 | BB54.00 | [M]scirrhous adenocarcinoma |
| 39038 | BB85.00 | [M]signet ring carcinoma |
| 94438 | BB85z00 | [M]signet ring carcinoma nos |
| 61588 | BB85000 | [M]signet ring cell carcinoma |
| 9291 | BB1J.00 | [M]small cell carcinoma nos |
| 94083 | BB5P.00 | [M]solid carcinoma nos |
| 61984 | BB1G.00 | [M]spheroidal cell carcinoma |
| 6966 | BB1D.00 | [M]spindle cell carcinoma |
| 57680 | BB2A.12 | [M]spinous cell carcinoma |
| 1624 | BB2A.00 | [M]squamous cell carcinoma nos |
| 29787 | BB2C.00 | [M]squamous cell carcinoma, keratinising type nos |
| 59143 | BB2D.00 | [M]squamous cell carcinoma, large cell, non-keratinising |
| 41816 | BB2E.00 | [M]squamous cell carcinoma, small cell, non-keratinising |
| 45458 | BB2F.00 | [M]squamous cell carcinoma, spindle cell type |
| 71895 | BB56.00 | [M]superficial spreading adenocarcinoma |
| 52493 | BBQ7213 | [M]teratoblastoma, malignant |
| 37542 | BBQ7300 | [M]teratocarcinoma |
| 107681 | BBc3.00 | [M]teratoid medulloepithelioma |
| 33636 | BBQ7200 | [M]teratoma, malignant, nos |
| 98781 | BB5F.00 | [M]trabecular adenocarcinoma |
| 6436 | BB43.00 | [M]transitional cell carcinoma nos |
| 58798 | BB47.00 | [M]transitional cell carcinoma, spindle cell type |
| 40492 | BBe9.00 | [M]triton tumour, malignant |
| 60045 | BB5M100 | [M]tubular adenocarcinoma |
| 8627 | BB07.00 | [M]tumour cells, malignant |
| 12388 | BB43.11 | [M]urothelial carcinoma |
| 34395 | BB24.00 | [M]verrucous carcinoma nos |
| 43717 | BB24.11 | [M]verrucous epidermoid carcinoma |
| 4852 | BB24.12 | [M]verrucous squamous cell carcinoma |
| 27849 | BB5U200 | [M]villous adenocarcinoma |
| 61764 | BB5y100 | [M]vipoma |
| 42509 | ZV67900 | [V]follow-up exam aft combined treatment for malig neoplasm |
| 31561 | ZV67600 | [V]follow-up examination aft surgery for malignant neoplasm |
| 44421 | ZV67A00 | [V]folow-up exam aft other treatment for malignant neoplasm |
| 36321 | ZV67B00 | [V]folow-up exam aft unspec treatment for malignant neoplasm |
| 113162 | ZVu0900 | [X]follow-up examinat after other treatment for malig neopl |
| 112035 | AyuC800 | [X]hiv disease resulting in other malignant neoplasms |
| 112036 | AyuC900 | [X]hiv disease resulting in unspecified malignant neoplasm |
| 35186 | ByuC.00 | [X]malignant neoplasm of ill-defined, secondary and unspeci |
| 39027 | ByuC000 | [X]malignant neoplasm of other specified sites |
| 52029 | ByuC800 | [X]malignant neoplasm without specification of site |
| 104291 | B61..11 | Hodgkin lymphoma |
| 106349 | B61z.11 | Hodgkin lymphoma nos |
| 2462 | B61..00 | Hodgkin's disease |
| 53397 | B61z.00 | Hodgkin's disease nos |
| 42461 | B61zz00 | Hodgkin's disease nos |
| 107804 | B61z300 | Hodgkin's disease nos of intra-abdominal lymph nodes |
| 59755 | B61z200 | Hodgkin's disease nos of intrathoracic lymph nodes |
| 99012 | B61z500 | Hodgkin's disease nos of lymph nodes inguinal region and leg |
| 91900 | B61z400 | Hodgkin's disease nos of lymph nodes of axilla and arm |
| 59778 | B61z100 | Hodgkin's disease nos of lymph nodes of head, face and neck |
| 97746 | B61z800 | Hodgkin's disease nos of lymph nodes of multiple sites |
| 94279 | B61z700 | Hodgkin's disease nos of spleen |
| 61662 | B61z000 | Hodgkin's disease nos, unspecified site |
| 67703 | B616.00 | Hodgkin's disease, lymphocytic depletion |
| 101530 | B616z00 | Hodgkin's disease, lymphocytic depletion nos |
| 101715 | B616700 | Hodgkin's disease, lymphocytic depletion of spleen |
| 38939 | B613.00 | Hodgkin's disease, lymphocytic-histiocytic predominance |
| 49605 | B615.00 | Hodgkin's disease, mixed cellularity |
| 94005 | B615z00 | Hodgkin's disease, mixed cellularity nos |
| 97863 | B615000 | Hodgkin's disease, mixed cellularity of unspecified site |
| 29178 | B614.00 | Hodgkin's disease, nodular sclerosis |
| 63054 | B614z00 | Hodgkin's disease, nodular sclerosis nos |
| 105472 | B614700 | Hodgkin's disease, nodular sclerosis of spleen |
| 57225 | B614000 | Hodgkin's disease, nodular sclerosis of unspecified site |
| 44196 | B611.00 | Hodgkin's granuloma |
| 98909 | B611100 | Hodgkin's granuloma of lymph nodes of head, face and neck |
| 63625 | B616400 | Hodgkin's lymphocytic depletion lymph nodes axilla and arm |
| 110563 | B616500 | Hodgkin's lymphocytic depletion lymph nodes inguinal and leg |
| 107032 | B616800 | Hodgkin's lymphocytic depletion lymph nodes multiple sites |
| 111942 | B616100 | Hodgkin's lymphocytic depletion of head, face and neck |
| 95049 | B616000 | Hodgkin's lymphocytic depletion of unspecified site |
| 58684 | B615200 | Hodgkin's mixed cellularity of intrathoracic lymph nodes |
| 94407 | B615100 | Hodgkin's mixed cellularity of lymph nodes head, face, neck |
| 108886 | B615500 | Hodgkin's mixed cellularity of lymph nodes inguinal and leg |
| 55303 | B614100 | Hodgkin's nodular sclerosis of head, face and neck |
| 61149 | B614300 | Hodgkin's nodular sclerosis of intra-abdominal lymph nodes |
| 67506 | B614200 | Hodgkin's nodular sclerosis of intrathoracic lymph nodes |
| 65483 | B614400 | Hodgkin's nodular sclerosis of lymph nodes of axilla and arm |
| 19140 | B614800 | Hodgkin's nodular sclerosis of lymph nodes of multiple sites |
| 65489 | B610.00 | Hodgkin's paragranuloma |
| 113111 | B610z00 | Hodgkin's paragranuloma nos |
| 98840 | B610300 | Hodgkin's paragranuloma of intra-abdominal lymph nodes |
| 64036 | B612.00 | Hodgkin's sarcoma |
| 112982 | B612300 | Hodgkin's sarcoma of intra-abdominal lymph nodes |
| 68039 | B612400 | Hodgkin's sarcoma of lymph nodes of axilla and upper limb |
| 113613 | B613400 | Hodgkin's, lymphocytic-histiocytic pred axilla and arm |
| 93951 | B613500 | Hodgkin's, lymphocytic-histiocytic pred inguinal and leg |
| 73532 | B613300 | Hodgkin's, lymphocytic-histiocytic pred intra-abdominal node |
| 95338 | B613600 | Hodgkin's, lymphocytic-histiocytic pred intrapelvic nodes |
| 92245 | B613200 | Hodgkin's, lymphocytic-histiocytic pred intrathoracic nodes |
| 68330 | B613100 | Hodgkin's, lymphocytic-histiocytic pred of head, face, neck |
| 104743 | B613800 | Hodgkin's, lymphocytic-histiocytic pred of multiple sites |
| 29876 | B613z00 | Hodgkin's, lymphocytic-histiocytic predominance nos |
| 106911 | B613700 | Hodgkin's, lymphocytic-histiocytic predominance of spleen |
| 71142 | B613000 | Hodgkin's, lymphocytic-histiocytic predominance unspec site |
| 106597 | B61B.00 | Lymphocyte-rich classical hodgkin lymphoma |
| 108775 | B619.00 | Mixed cellularity classical hodgkin lymphoma |
| 104895 | B617.00 | Nodular lymphocyte predominant hodgkin lymphoma |
| 105841 | B618.00 | Nodular sclerosis classical hodgkin lymphoma |
| 104484 | B61C.00 | Other classical hodgkin lymphoma |
| 20710 | BBj..00 | [M]hodgkin's disease |
| 42769 | BBjz.00 | [M]hodgkin's disease nos |
| 61997 | BBj0.00 | [M]hodgkin's disease nos |
| 111113 | BBj3.00 | [M]hodgkin's disease, lymphocytic depletion nos |
| 56041 | BBj1.00 | [M]hodgkin's disease, lymphocytic predominance |
| 51285 | BBj2.00 | [M]hodgkin's disease, mixed cellularity |
| 42198 | BBj6.00 | [M]hodgkin's disease, nodular sclerosis nos |
| 99200 | BBj7.00 | [M]hodgkin's disease, nodular sclerosis, cellular phase |
| 96183 | BBj4.00 | [M]hodgkin's disease,lymphocytic depletion,diffuse fibrosis |
| 89230 | BBj9.00 | [M]hodgkin's granuloma |
| 65584 | BBj1000 | [M]hodgkin,s disease, lymphocytic predominance, diffuse |
| 31537 | BBj1100 | [M]hodgkin,s disease, lymphocytic predominance, nodular |
| 31741 | BBj6200 | [M]hodgkin,s disease, nodular sclerosis, lymphocytic deplet |
| 40508 | BBj6000 | [M]hodgkin,s disease, nodular sclerosis, lymphocytic predom |
| 64343 | BBj6100 | [M]hodgkin,s disease, nodular sclerosis, mixed cellularity |
| 101429 | BBj0.11 | [M]lymphogranuloma, malignant |
| 43415 | ByuD000 | [X]other hodgkin's disease |
| 108719 | B628600 | Cutaneous follicle centre lymphoma |
| 106969 | B628500 | Diffuse follicle centre lymphoma |
| 104152 | B628.00 | Follicular lymphoma |
| 105889 | B628000 | Follicular lymphoma grade 1 |
| 105095 | B628100 | Follicular lymphoma grade 2 |
| 107166 | B628200 | Follicular lymphoma grade 3 |
| 105020 | B628300 | Follicular lymphoma grade 3a |
| 107973 | B628400 | Follicular lymphoma grade 3b |
| 17182 | B627C11 | Follicular lymphoma nos |
| 70842 | B627100 | Follicular non-hodg mixed sml cleavd & lge cell lymphoma |
| 49262 | B627200 | Follicular non-hodgkin's large cell lymphoma |
| 21549 | B627C00 | Follicular non-hodgkin's lymphoma |
| 28639 | B627000 | Follicular non-hodgkin's small cleaved cell lymphoma |
| 5179 | B620.00 | Nodular lymphoma (brill - symmers disease) |
| 65701 | B620z00 | Nodular lymphoma nos |
| 92068 | B620300 | Nodular lymphoma of intra-abdominal lymph nodes |
| 105203 | B620200 | Nodular lymphoma of intrathoracic lymph nodes |
| 111766 | B620400 | Nodular lymphoma of lymph nodes of axilla and upper limb |
| 45264 | B620100 | Nodular lymphoma of lymph nodes of head, face and neck |
| 94995 | B620500 | Nodular lymphoma of lymph nodes of inguinal region and leg |
| 58082 | B620800 | Nodular lymphoma of lymph nodes of multiple sites |
| 114025 | B620700 | Nodular lymphoma of spleen |
| 66327 | B620000 | Nodular lymphoma of unspecified site |
| 106063 | B628700 | Other types of follicular lymphoma |
| 31576 | B627B00 | Other types of follicular non-hodgkin's lymphoma |
| 64947 | BBk0.11 | [M]brill - symmers' disease |
| 27562 | BBk0.12 | [M]follicular lymphosarcoma nos |
| 49253 | BBk0.13 | [M]giant follicular lymphoma |
| 40513 | BBkz.00 | [M]lymphoma, nodular or follicular nos |
| 20437 | BBk..00 | [M]lymphomas, nodular or follicular |
| 39883 | BBk5.00 | [M]malig lymp, follicular centre cell, cleaved, follicular |
| 58953 | BBk8.00 | [M]malig lymp,follicular centre cell,noncleaved,follicular |
| 66603 | BBgK.00 | [M]malig lymphoma, follicular centre cell, non-cleaved nos |
| 106970 | BBk3.00 | [M]malig lymphoma, lymphocytic, well differentiated,nodular |
| 97852 | BBk7.00 | [M]malignant lymphoma, centroblastic type, follicular |
| 98961 | BBk2.00 | [M]malignant lymphoma, centroblastic-centrocytic, follicular |
| 41841 | BBgB.00 | [M]malignant lymphoma, follicular centre cell nos |
| 63699 | BBk0.00 | [M]malignant lymphoma, nodular nos |
| 67518 | ByuD100 | [X]other types of follicular non-hodgkin's lymphoma |
| 104325 | B640000 | B-cell acute lymphoblastic leukaemia |
| 21402 | B602.00 | Burkitt's lymphoma |
| 71304 | B602z00 | Burkitt's lymphoma nos |
| 97577 | B602300 | Burkitt's lymphoma of intra-abdominal lymph nodes |
| 100006 | B602200 | Burkitt's lymphoma of intrathoracic lymph nodes |
| 59115 | B602100 | Burkitt's lymphoma of lymph nodes of head, face and neck |
| 92380 | B602500 | Burkitt's lymphoma of lymph nodes of inguinal region and leg |
| 113020 | B602800 | Burkitt's lymphoma of lymph nodes of multiple sites |
| 102594 | B627E00 | Diffuse large b-cell lymphoma |
| 50695 | B627500 | Diffuse non-hodgkin mixed sml & lge cell (diffuse) lymphoma |
| 70509 | B627D00 | Diffuse non-hodgkin's centroblastic lymphoma |
| 53551 | B627600 | Diffuse non-hodgkin's immunoblastic (diffuse) lymphoma |
| 101114 | B627A00 | Diffuse non-hodgkin's large cell lymphoma |
| 17460 | B627700 | Diffuse non-hodgkin's lymphoblastic (diffuse) lymphoma |
| 65180 | B627800 | Diffuse non-hodgkin's lymphoma undifferentiated (diffuse) |
| 39798 | B627X00 | Diffuse non-hodgkin's lymphoma, unspecified |
| 50668 | B627300 | Diffuse non-hodgkin's small cell (diffuse) lymphoma |
| 108182 | B627400 | Diffuse non-hodgkin's small cleaved cell (diffuse) lymphoma |
| 111980 | A789611 | Hiv disease resulting in burkitt lymphoma |
| 44617 | A789600 | Hiv disease resulting in burkitt's lymphoma |
| 104412 | B62F200 | Lymphoblastic (diffuse) lymphoma |
| 103245 | B601700 | Lymphosarcoma of spleen |
| 104620 | B62F100 | Mantle cell lymphoma |
| 112845 | B640011 | Mature b-cell leukaemia burkitt-type |
| 105038 | B627G00 | Mediastinal (thymic) large b-cell lymphoma |
| 106867 | B62F.11 | Non-follicular lymphoma |
| 106884 | B62F.00 | Nonfollicular lymphoma |
| 1481 | B600.00 | Reticulosarcoma |
| 99240 | B600z00 | Reticulosarcoma nos |
| 70374 | B600300 | Reticulosarcoma of intra-abdominal lymph nodes |
| 71031 | B600100 | Reticulosarcoma of lymph nodes of head, face and neck |
| 95058 | B600700 | Reticulosarcoma of spleen |
| 60242 | B600000 | Reticulosarcoma of unspecified site |
| 104386 | B62F000 | Small cell b-cell lymphoma |
| 18383 | BBmH.00 | [M] large cell lymphoma |
| 113891 | BBq..00 | [M]burkitt's tumours |
| 34352 | BBgG.12 | [M]lymphoblastic lymphoma nos |
| 67203 | BBgG.11 | [M]lymphoblastic lymphosarcoma nos |
| 52591 | BBgG.13 | [M]lymphoblastoma nos |
| 61251 | BBgN.00 | [M]malign lymphoma,lymphocytic,intermediate differn, diffuse |
| 68964 | BBgA.00 | [M]malignant lymphoma, centroblastic-centrocytic, diffuse |
| 39906 | BBgE.00 | [M]malignant lymphoma, centrocytic |
| 23711 | BBg1000 | [M]malignant lymphoma, diffuse nos |
| 48253 | BBg8.00 | [M]malignant lymphoma, immunoblastic type |
| 63994 | BBgS.00 | [M]malignant lymphoma, large cell, cleaved, diffuse |
| 33869 | BBgR.00 | [M]malignant lymphoma, large cell, diffuse nos |
| 71619 | BBgT.00 | [M]malignant lymphoma, large cell, noncleaved, diffuse |
| 41754 | BBg7.00 | [M]malignant lymphoma, lymphoplasmacytoid type |
| 71652 | BBgP.00 | [M]malignant lymphoma, mixed small and large cell, diffuse |
| 51680 | BBgV.00 | [M]malignant lymphoma, small cell, noncleaved, diffuse |
| 31726 | BBgM.00 | [M]malignant lymphoma, small cleaved cell, diffuse |
| 46877 | BBgL.00 | [M]malignant lymphoma, small lymphocytic nos |
| 58015 | BBgQ.00 | [M]malignant lymphomatous polyposis |
| 72241 | BBgH.00 | [M]prolymphocytic lymphosarcoma |
| 72433 | BBh0.00 | [M]reticulosarcoma nos |
| 112729 | BBh1.00 | [M]reticulosarcoma, pleomorphic cell type |
| 106137 | BBh..00 | [M]reticulosarcomas |
| 49825 | BBh0.11 | [M]reticulum cell sarcoma nos |
| 64515 | ByuDC00 | [X]diffuse non-hodgkin's lymphoma, unspecified |
| 98596 | ByuD200 | [X]other types of diffuse non-hodgkin's lymphoma |
| 105955 | B62E200 | Anaplastic large cell lymphoma, alk-negative |
| 105559 | B62E100 | Anaplastic large cell lymphoma, alk-positive |
| 104862 | B62E300 | Cutaneous t-cell lymphoma |
| 57737 | B62x100 | Lymphoepithelioid lymphoma |
| 12006 | B621.00 | Mycosis fungoides |
| 38005 | B621z00 | Mycosis fungoides nos |
| 91674 | B621300 | Mycosis fungoides of intra-abdominal lymph nodes |
| 96379 | B621400 | Mycosis fungoides of lymph nodes of axilla and upper limb |
| 72714 | B621500 | Mycosis fungoides of lymph nodes of inguinal region and leg |
| 95012 | B621800 | Mycosis fungoides of lymph nodes of multiple sites |
| 95949 | B621000 | Mycosis fungoides of unspecified site |
| 44318 | B62xX00 | Oth and unspecif peripheral & cutaneous t-cell lymphomas |
| 104934 | B62Ew00 | Other mature t/nk-cell lymphoma |
| 12464 | B62x200 | Peripheral t-cell lymphoma |
| 35014 | B622.00 | Sezary's disease |
| 100532 | B622z00 | Sezary's disease nos |
| 90201 | B62x000 | T-zone lymphoma |
| 105085 | B62E.00 | T/nk-cell lymphoma |
| 16774 | BBmD.00 | [M] cutaneous lymphoma |
| 40766 | BBm5.00 | [M] peripheral t-cell lymphoma nos |
| 46967 | BBl..00 | [M]mycosis fungoides |
| 95464 | BBl0.00 | [M]mycosis fungoides |
| 99695 | BBlz.00 | [M]mycosis fungoides nos |
| 97756 | BBl1.00 | [M]sezary's disease |
| 109714 | ByuDD00 | [X]oth and unspecif peripheral & cutaneous t-cell lymphomas |
| 66367 | A789700 | Hiv dis resulting oth types of non-hodgkin's lymphoma |
| 111981 | A789711 | Hiv disease resulting in other types of non-hodgkin lymphoma |
| 60918 | 4M20.00 | Lymphoma stage i |
| 94935 | 4M21.00 | Lymphoma stage ii |
| 32240 | 4M22.00 | Lymphoma stage iii |
| 71672 | 4M23.00 | Lymphoma stage iv |
| 27416 | B601.00 | Lymphosarcoma |
| 41369 | B60..00 | Lymphosarcoma and reticulosarcoma |
| 63723 | B601z00 | Lymphosarcoma nos |
| 64670 | B601300 | Lymphosarcoma of intra-abdominal lymph nodes |
| 62380 | B601200 | Lymphosarcoma of intrathoracic lymph nodes |
| 71238 | B601100 | Lymphosarcoma of lymph nodes of head, face and neck |
| 100352 | B601500 | Lymphosarcoma of lymph nodes of inguinal region and leg |
| 104790 | B601800 | Lymphosarcoma of lymph nodes of multiple sites |
| 71625 | B601000 | Lymphosarcoma of unspecified site |
| 12335 | B62y.00 | Malignant lymphoma nos |
| 15027 | B62yz00 | Malignant lymphoma nos |
| 42579 | B62y300 | Malignant lymphoma nos of intra-abdominal lymph nodes |
| 71262 | B62y600 | Malignant lymphoma nos of intrapelvic lymph nodes |
| 72725 | B62y200 | Malignant lymphoma nos of intrathoracic lymph nodes |
| 63105 | B62y500 | Malignant lymphoma nos of lymph node inguinal region and leg |
| 34089 | B62y400 | Malignant lymphoma nos of lymph nodes of axilla and arm |
| 50696 | B62y100 | Malignant lymphoma nos of lymph nodes of head, face and neck |
| 15504 | B62y800 | Malignant lymphoma nos of lymph nodes of multiple sites |
| 60092 | B62y700 | Malignant lymphoma nos of spleen |
| 57427 | B62y000 | Malignant lymphoma nos of unspecified site |
| 17887 | B62x.00 | Malignant lymphoma otherwise specified |
| 62437 | B62x400 | Malignant reticulosis |
| 3604 | B627.00 | Non - hodgkin's lymphoma |
| 104391 | B627.11 | Non-hodgkin lymphoma |
| 111682 | B62Fy00 | Other non-follicular lymphoma |
| 99887 | B60y.00 | Other specified reticulosarcoma or lymphosarcoma |
| 113458 | B620.11 | Reticulosarcoma - follicular or nodular |
| 99951 | B60z.00 | Reticulosarcoma or lymphosarcoma nos |
| 31794 | B627W00 | Unspecified b-cell non-hodgkin's lymphoma |
| 68353 | BBmJ.00 | [M] angioendotheliomatosis |
| 31492 | BBm9.00 | [M] monocytoid b-cell lymphoma |
| 27965 | BBv2.00 | [M]angiocentrict-cell lymphoma |
| 21463 | BBgC.11 | [M]lymphocytic lymphoma nos |
| 60504 | BBgC.12 | [M]lymphocytic lymphosarcoma nos |
| 1483 | BBg1.11 | [M]lymphoma nos |
| 51895 | BBgz.00 | [M]lymphoma, diffuse or nos |
| 17178 | BBg..00 | [M]lymphomas, nos or diffuse |
| 99655 | BBg6.00 | [M]lymphosarcoma nos |
| 51852 | BBgD.00 | [M]malig lymphoma, lymphocytic, intermediate different nos |
| 36114 | BBg1.00 | [M]malignant lymphoma nos |
| 60275 | BBgJ.00 | [M]malignant lymphoma, centroblastic type nos |
| 69301 | BBg5.00 | [M]malignant lymphoma, convoluted cell type nos |
| 72196 | BBgG.00 | [M]malignant lymphoma, lymphocytic, poorly different nos |
| 69980 | BBgC.00 | [M]malignant lymphoma, lymphocytic, well differentiated nos |
| 16460 | BBg2.00 | [M]malignant lymphoma, non hodgkin's type |
| 46931 | BBg4.00 | [M]malignant lymphoma, stem cell type |
| 71117 | BBg3.00 | [M]malignant lymphoma, undifferentiated cell type nos |
| 70740 | BBm1.11 | [M]malignant reticulosis |
| 63973 | BBm0.00 | [M]microglioma |
| 31749 | BBv0.00 | [M]monocytoid b-cell lymphoma |
| 3371 | BBg2.11 | [M]non hodgkins lymphoma |
| 100544 | BBh2.00 | [M]reticulosarcoma, nodular |
| 112033 | AyuC611 | [X]hiv disease resulting in other non-hodgkin lymphoma |
| 69767 | AyuC600 | [X]hiv disease resulting in other non-hodgkin's lymphoma |
| 7940 | ByuDF11 | [X]non-hodgkin's lymphoma nos |
| 8649 | ByuDF00 | [X]non-hodgkin's lymphoma, unspecified type |
| 64336 | ByuD300 | [X]other specified types of non-hodgkin's lymphoma |
| 63375 | ByuDE00 | [X]unspecified b-cell non-hodgkin's lymphoma |
| 105636 | B62E900 | Angioimmunoblastic t-cell lymphoma |
| 105375 | B62E800 | Blastic nk-cell lymphoma |
| 105709 | B62E600 | Enteropathy-associated t-cell lymphoma |
| 109780 | B62E400 | Extranodal nk/t-cell lymphoma, nasal type |
| 107949 | B62E500 | Hepatosplenic t-cell lymphoma |
| 105925 | B62E700 | Subcutaneous panniculitic t-cell lymphoma |
| 101350 | C333100 | Alpha heavy chain disease |
| 105966 | B627F00 | Extranod marg zone b-cell lymphom mucosa-assoc lymphoid tiss |
| 99067 | C333200 | Gamma heavy chain disease |
| 108102 | C333300 | Heavy chain disease |
| 58962 | B62x500 | Malignant immunoproliferative small intestinal disease |
| 95715 | B627900 | Mucosa-associated lymphoma |
| 108235 | C333011 | Waldenstrom macroglobulinaemia |
| 10411 | C333000 | Waldenstrom's macroglobulinaemia |
| 26135 | BBm6.00 | [M] alpha heavy chain disease |
| 52593 | BBmE.00 | [M] gamma heavy chain disease |
| 9172 | BBmK.00 | [M]waldenstrom's macroglobulinaemia |
| 102688 | ByuD400 | [X]other malignant immunoproliferative diseases |
| 52946 | 4C53.00 | Bone marrow: myeloma cells |
| 43552 | B630.11 | Kahler's disease |
| 46042 | B630300 | Lambda light chain myeloma |
| 22158 | B630000 | Malignant plasma cell neoplasm, extramedullary plasmacytoma |
| 4944 | B630.00 | Multiple myeloma |
| 43312 | B936.11 | Myeloma - solitary |
| 15211 | B630.12 | Myelomatosis |
| 60433 | N330900 | Osteoporosis in multiple myelomatosis |
| 39187 | B631.00 | Plasma cell leukaemia |
| 21329 | B630200 | Plasmacytoma nos |
| 38321 | B936.12 | Plasmacytoma nos |
| 19028 | B630100 | Solitary myeloma |
| 104418 | B630400 | Solitary plasmacytoma |
| 102164 | BBn2.11 | [M]monostotic myeloma |
| 18744 | BBn0.11 | [M]multiple myeloma |
| 3672 | BBn0.12 | [M]myeloma nos |
| 53647 | BBn0.13 | [M]myelomatosis |
| 113633 | BBr3000 | [M]plasma cell leukaemia |
| 110349 | BBr3z00 | [M]plasma cell leukaemia nos |
| 64618 | BBr3.00 | [M]plasma cell leukaemias |
| 31671 | BBn0.00 | [M]plasma cell myeloma |
| 99702 | BBn3.00 | [M]plasma cell tumour, malignant |
| 39490 | BBn0.14 | [M]plasmacytic myeloma |
| 63864 | BBn2.00 | [M]plasmacytoma nos |
| 73135 | BBn2.12 | [M]solitary myeloma |
| 4251 | B640.00 | Acute lymphoid leukaemia |
| 37461 | B64y200 | Adult t-cell leukaemia |
| 104939 | B64y500 | Adult t-cell lymphoma/leukaemia (htlv-1-associated) |
| 104328 | B641000 | B-cell chronic lymphocytic leukaemia |
| 108656 | B64y300 | B-cell prolymphocytic leukaemia |
| 27790 | B641.11 | Chronic lymphatic leukaemia |
| 107017 | B641011 | Chronic lymphocytic leukaemia of b-cell type |
| 8625 | B641.00 | Chronic lymphoid leukaemia |
| 107052 | B641100 | Clinical stage a chronic lymphocytic leukaemia |
| 106924 | B641200 | Clinical stage b chronic lymphocytic leukaemia |
| 107163 | B641300 | Clinical stage c chronic lymphocytic leukaemia |
| 87335 | B624.12 | Hairy cell leukaemia |
| 65123 | B624300 | Leukaemic reticuloend of intra-abdominal lymph nodes |
| 5137 | B624.11 | Leukaemic reticuloendotheliosis |
| 27330 | B624.00 | Leukaemic reticuloendotheliosis |
| 73777 | B624z00 | Leukaemic reticuloendotheliosis nos |
| 65122 | B624000 | Leukaemic reticuloendotheliosis of unspecified sites |
| 4222 | B64..11 | Lymphatic leukaemia |
| 19372 | B64..00 | Lymphoid leukaemia |
| 38914 | B64z.00 | Lymphoid leukaemia nos |
| 49725 | B64y.00 | Other lymphoid leukaemia |
| 38331 | B64yz00 | Other lymphoid leukaemia nos |
| 31586 | B64y100 | Prolymphocytic leukaemia |
| 111627 | B64y411 | Prolymphocytic leukaemia of t-cell type |
| 72774 | B642.00 | Subacute lymphoid leukaemia |
| 107643 | B64y400 | T-cell prolymphocytic leukaemia |
| 37410 | BBr2100 | [M]acute lymphoid leukaemia |
| 29335 | BBr2700 | [M]adult t-cell leukaemia/lymphoma |
| 50928 | BBr2600 | [M]burkitt's cell leukaemia |
| 41500 | BBr2300 | [M]chronic lymphoid leukaemia |
| 5915 | BBrA400 | [M]hairy cell leukaemia |
| 20635 | BBr2011 | [M]lymphatic leukaemia |
| 12146 | BBr2000 | [M]lymphoid leukaemia nos |
| 48155 | BBr2.00 | [M]lymphoid leukaemias |
| 46048 | BBr2500 | [M]prolymphocytic leukaemia |
| 67029 | ByuD500 | [X]other lymphoid leukaemia |
| 104788 | B654.00 | Acute myeloblastic leukaemia |
| 4413 | B650.00 | Acute myeloid leukaemia |
| 61500 | B690.00 | Acute myelomonocytic leukaemia |
| 27664 | B65y100 | Acute promyelocytic leukaemia |
| 113527 | B65y000 | Aleukaemic myeloid leukaemia |
| 107236 | B651300 | Atypical chronic myeloid leukaemia, bcr/abl negative |
| 52327 | B653000 | Chloroma |
| 31701 | B651.11 | Chronic granulocytic leukaemia |
| 10726 | B651.00 | Chronic myeloid leukaemia |
| 27520 | B651z00 | Chronic myeloid leukaemia nos |
| 105957 | B651100 | Chronic myeloid leukaemia, bcr/abl positive |
| 22050 | B691.00 | Chronic myelomonocytic leukaemia |
| 39629 | B653100 | Granulocytic sarcoma |
| 113464 | B624100 | Leukaemic reticuloend of lymph nodes of head, face and neck |
| 7176 | B65..00 | Myeloid leukaemia |
| 33344 | B65z.00 | Myeloid leukaemia nos |
| 70724 | B653.00 | Myeloid sarcoma |
| 113098 | B653z00 | Myeloid sarcoma nos |
| 20440 | B69..00 | Myelomonocytic leukaemia |
| 112440 | B65y.00 | Other myeloid leukaemia |
| 66089 | B65yz00 | Other myeloid leukaemia nos |
| 63475 | B652.00 | Subacute myeloid leukaemia |
| 54585 | BBr6100 | [M]acute myeloid leukaemia |
| 46263 | BBr6700 | [M]acute myelomonocytic leukaemia |
| 57316 | BBr6600 | [M]acute promyelocytic leukaemia |
| 106197 | BBr7000 | [M]basophilic leukaemia |
| 93944 | BBrA311 | [M]chloroma |
| 52942 | BBr6300 | [M]chronic myeloid leukaemia |
| 48049 | BBr6800 | [M]chronic myelomonocytic leukaemia |
| 71377 | BBr8000 | [M]eosinophilic leukaemia |
| 107773 | BBr8z00 | [M]eosinophilic leukaemia nos |
| 37723 | BBr6011 | [M]granulocytic leukaemia nos |
| 98009 | BBrA312 | [M]granulocytic sarcoma |
| 71850 | BBr6000 | [M]myeloid leukaemia nos |
| 35697 | BBr6.00 | [M]myeloid leukaemias |
| 96893 | BBrA300 | [M]myeloid sarcoma |
| 62330 | BBr6z00 | [M]other myeloid leukaemia nos |
| 106483 | BBr6200 | [M]subacute myeloid leukaemia |
| 61693 | ByuD600 | [X]other myeloid leukaemia |
| 108424 | B663.00 | Acute monoblastic leukaemia |
| 19974 | B660.00 | Acute monocytic leukaemia |
| 27458 | B661.00 | Chronic monocytic leukaemia |
| 105069 | B693.00 | Juvenile myelomonocytic leukaemia |
| 35875 | B66..00 | Monocytic leukaemia |
| 93342 | B66z.00 | Monocytic leukaemia nos |
| 99015 | B66y.00 | Other monocytic leukaemia |
| 103645 | B66yz00 | Other monocytic leukaemia nos |
| 101606 | B662.00 | Subacute monocytic leukaemia |
| 104475 | B692.00 | Subacute myelomonocytic leukaemia |
| 113499 | BBr9300 | [M]chronic monocytic leukaemia |
| 108964 | BBr6900 | [M]juvenile myelomonocytic leukaemia |
| 73088 | BBr9000 | [M]monocytic leukaemia nos |
| 66694 | BBr6311 | [M]naegeli-type monocytic leukaemia |
| 113680 | BBr9012 | [M]schilling-type monocytic leukaemia |
| 89762 | ByuD700 | [X]other monocytic leukaemia |
| 42539 | B670.00 | Acute erythraemia and erythroleukaemia |
| 110838 | B676.00 | Acute erythroid leukaemia |
| 28276 | B675.00 | Acute myelofibrosis |
| 50858 | B674.00 | Acute panmyelosis |
| 37468 | B671.00 | Chronic erythraemia |
| 27340 | B670.11 | Di guglielmo's disease |
| 72197 | B67y000 | Lymphosarcoma cell leukaemia |
| 65721 | B673.00 | Mast cell leukaemia |
| 57671 | B672.00 | Megakaryocytic leukaemia |
| 67700 | B66..12 | Monoblastic leukaemia |
| 104273 | B677.00 | Myelodysplastic and myeloproliferative disease |
| 110065 | B6y1.12 | Osteomyelofibrosis |
| 37272 | B67..00 | Other specified leukaemia |
| 30632 | B67z.00 | Other specified leukaemia nos |
| 65777 | B672.11 | Thrombocytic leukaemia |
| 113637 | BBr4100 | [M]acute erythraemia |
| 49327 | BBrA500 | [M]acute megakaryoblastic leukaemia |
| 37487 | BBrA700 | [M]acute myelofibrosis |
| 102764 | BBrA600 | [M]acute panmyelosis |
| 101271 | BBs1.00 | [M]acute panmyelosis |
| 70935 | BBr4000 | [M]erythroleukaemia |
| 100927 | BBr4z00 | [M]erythroleukaemia nos |
| 46444 | BBr4.00 | [M]erythroleukaemias |
| 72222 | BBrA100 | [M]megakaryocytic leukaemia |
| 69299 | BBrA111 | [M]thrombocytic leukaemia |
| 89329 | ByuD800 | [X]other specified leukaemias |
| 4072 | B680.00 | Acute leukaemia nos |
| 16416 | B681.00 | Chronic leukaemia nos |
| 108715 | B66..11 | Histiocytic leukaemia |
| 4250 | B68z.00 | Leukaemia nos |
| 25191 | B68..00 | Leukaemia of unspecified cell type |
| 94174 | B67y.00 | Other and unspecified leukaemia |
| 99413 | B67yz00 | Other and unspecified leukaemia nos |
| 34692 | B68y.00 | Other leukaemia of unspecified cell type |
| 54793 | B682.00 | Subacute leukaemia nos |
| 6316 | BBr0100 | [M]acute leukaemia nos |
| 72310 | BBr0400 | [M]aleukaemic leukaemia nos |
| 22071 | BBr0111 | [M]blast cell leukaemia |
| 64963 | BBr0112 | [M]blastic leukaemia |
| 31750 | BBr0300 | [M]chronic leukaemia nos |
| 57713 | BBr8.00 | [M]eosinophilic leukaemias |
| 41734 | BBr0000 | [M]leukaemia nos |
| 42297 | BBrz.00 | [M]leukaemia nos |
| 59929 | BBr0z00 | [M]leukaemia unspecified, nos |
| 4637 | BBr..00 | [M]leukaemias |
| 40420 | BBr0.00 | [M]leukaemias unspecified |
| 108316 | BBrAz00 | [M]miscellaneous leukaemia nos |
| 73066 | BBrA.00 | [M]miscellaneous leukaemias |
| 24317 | BBr6012 | [M]myelosis nos |
| 63570 | BBr0113 | [M]stem cell leukaemia |
| 72179 | BBr0200 | [M]subacute leukaemia nos |
| 65165 | ByuD900 | [X]other leukaemia of unspecified cell type |
| 105083 | B62D.00 | Histiocytic sarcoma |
| 4870 | B625.11 | Histiocytosis x (acute, progressive) |
| 51708 | A789X00 | Hiv dis reslt/oth mal neopl/lymph,h'matopoetc+reltd tissu |
| 34926 | B625.00 | Letterer-siwe disease |
| 47204 | B625z00 | Letterer-siwe disease nos |
| 102158 | B625200 | Letterer-siwe disease of intrathoracic lymph nodes |
| 54083 | B625800 | Letterer-siwe disease of lymph nodes of multiple sites |
| 102715 | B625000 | Letterer-siwe disease of unspecified sites |
| 95792 | B62zz00 | Lymphoid and histiocytic malignancy nos |
| 44267 | B623.00 | Malignant histiocytosis |
| 58871 | B623z00 | Malignant histiocytosis nos |
| 65642 | B623300 | Malignant histiocytosis of intra-abdominal lymph nodes |
| 94415 | B623100 | Malignant histiocytosis of lymph nodes head, face and neck |
| 112570 | B623400 | Malignant histiocytosis of lymph nodes of axilla and arm |
| 110903 | B623800 | Malignant histiocytosis of lymph nodes of multiple sites |
| 69497 | B623000 | Malignant histiocytosis of unspecified site |
| 89657 | B626z00 | Malignant mast cell tumour nos |
| 15036 | B626.00 | Malignant mast cell tumours |
| 49301 | B6z..00 | Malignant neoplasm lymphatic or haematopoietic tissue nos |
| 30646 | B6y..00 | Malignant neoplasm lymphatic or haematopoietic tissue os |
| 37112 | B6...11 | Malignant neoplasm of histiocytic tissue |
| 12323 | B6...00 | Malignant neoplasm of lymphatic and haemopoietic tissue |
| 65434 | B62z.00 | Malignant neoplasms of lymphoid and histiocytic tissue nos |
| 51029 | PH32111 | Mast cell disease |
| 100615 | B626500 | Mast cell malignancy of lymph nodes inguinal region and leg |
| 113526 | B626100 | Mast cell malignancy of lymph nodes of head, face and neck |
| 31324 | B626800 | Mast cell malignancy of lymph nodes of multiple sites |
| 103900 | B626000 | Mast cell malignancy of unspecified site |
| 17521 | PH32112 | Mastocytosis |
| 110191 | B62B.00 | Multifocal and unisystemic langerhans-cell histiocytosis |
| 50569 | B93X.00 | Neo/uncertn+unknwn behav/lymph,h'matopetc+rel tiss,unspcf |
| 33333 | B62..00 | Other malignant neoplasm of lymphoid and histiocytic tissue |
| 105335 | B62A.00 | Sarcoma of dendritic cells |
| 95630 | B62x600 | True histiocytic lymphoma |
| 105762 | B62C.00 | Unifocal langerhans-cell histiocytosis |
| 103353 | B62z300 | Unspec malig neop lymphoid/histiocytic intra-abdominal nodes |
| 107638 | B62z400 | Unspec malig neop lymphoid/histiocytic lymph node axilla/arm |
| 64427 | B62z100 | Unspec malig neop lymphoid/histiocytic lymph node head/neck |
| 71609 | B62z500 | Unspec malig neop lymphoid/histiocytic nodes inguinal/leg |
| 109342 | B62z600 | Unspec malig neop lymphoid/histiocytic of intrapelvic nodes |
| 93384 | B62z200 | Unspec malig neop lymphoid/histiocytic of intrathoracic node |
| 101465 | B62z800 | Unspec malig neop lymphoid/histiocytic of multiple sites |
| 108037 | B62z000 | Unspec malig neop lymphoid/histiocytic of unspecified site |
| 113011 | BBm3.11 | [M]acute infancy reticulosis |
| 45768 | BBm3.12 | [M]acute progressive histiocytosis x |
| 108682 | BBgJ.11 | [M]germinoblastic sarcoma nos |
| 47330 | BBm2.00 | [M]histiocytic medullary reticulosis |
| 59593 | BBm3.00 | [M]letterer - siwe disease |
| 63239 | BBm1.00 | [M]malignant histiocytosis |
| 67339 | BBp2.00 | [M]malignant mastocytosis |
| 94239 | BBp1.00 | [M]mast cell sarcoma |
| 57544 | BBm4.00 | [M]true histiocytic lymphoma |
| 112034 | AyuC700 | [X]hiv dis reslt/oth mal neopl/lymph,h'matopoetc+reltd tissu |
| 72500 | ByuDB00 | [X]mal neoplasm/lymphoid,haematopoietic+related tissu,unspcf |
| 40740 | ByuD.00 | [X]malignant neoplasms of lymphoid, haematopoietic and rela |
| 105025 | ByuDA00 | [X]oth spcf mal neoplsm/lymphoid,haematopoietic+rltd tissue |
| 51352 | B592.00 | Malignant neoplasms of independent (primary) multiple sites |
| 63598 | ByuE.00 | [X]malignant neoplasms/independent (primary) multiple sites |
| 64897 | ByuE000 | [X]malignant neoplasms/independent(primary)multiple sites |
| 7830 | B56..11 | Lymph node metastases |
| 25366 | B561300 | Secondary and unspec malig neop ant mediastinal lymph nodes |
| 44627 | B560800 | Secondary and unspec malig neop anterior cervical ln |
| 50199 | B563.00 | Secondary and unspec malig neop axilla and upper limb ln |
| 73538 | B563z00 | Secondary and unspec malig neop axilla and upper limb ln nos |
| 37540 | B563000 | Secondary and unspec malig neop axillary lymph nodes |
| 62124 | B561800 | Secondary and unspec malig neop bronchopulmonary lymph nodes |
| 41691 | B562000 | Secondary and unspec malig neop coeliac lymph nodes |
| 18658 | B562300 | Secondary and unspec malig neop common iliac lymph nodes |
| 68611 | B560900 | Secondary and unspec malig neop deep cervical ln |
| 61289 | B564100 | Secondary and unspec malig neop deep inguinal lymph nodes |
| 92703 | B560400 | Secondary and unspec malig neop deep parotid lymph nodes |
| 95378 | B561200 | Secondary and unspec malig neop diaphragmatic lymph nodes |
| 69132 | B562400 | Secondary and unspec malig neop external iliac lymph nodes |
| 61677 | B562200 | Secondary and unspec malig neop inferior mesenteric ln |
| 69392 | B561700 | Secondary and unspec malig neop inferior tracheobronchial ln |
| 50904 | B563200 | Secondary and unspec malig neop infraclavicular lymph nodes |
| 63915 | B564.00 | Secondary and unspec malig neop inguinal and lower limb ln |
| 105953 | B561100 | Secondary and unspec malig neop intercostal lymph nodes |
| 84368 | B565000 | Secondary and unspec malig neop internal iliac lymph nodes |
| 37919 | B561000 | Secondary and unspec malig neop internal mammary lymph nodes |
| 44931 | B562z00 | Secondary and unspec malig neop intra-abdominal ln nos |
| 52736 | B562.00 | Secondary and unspec malig neop intra-abdominal lymph nodes |
| 72803 | B565z00 | Secondary and unspec malig neop intrapelvic ln nos |
| 6701 | B565.00 | Secondary and unspec malig neop intrapelvic lymph nodes |
| 93716 | B561z00 | Secondary and unspec malig neop intrathoracic ln nos |
| 64116 | B561.00 | Secondary and unspec malig neop intrathoracic lymph nodes |
| 49214 | B560.00 | Secondary and unspec malig neop lymph nodes head/face/neck |
| 20159 | B56y.00 | Secondary and unspec malig neop lymph nodes multiple sites |
| 15507 | B56z.00 | Secondary and unspec malig neop lymph nodes nos |
| 28059 | B560600 | Secondary and unspec malig neop of facial lymph nodes |
| 70747 | B564z00 | Secondary and unspec malig neop of inguinal and leg ln nos |
| 64918 | B560000 | Secondary and unspec malig neop of superficial parotid ln |
| 58692 | B561500 | Secondary and unspec malig neop paratracheal lymph nodes |
| 46409 | B563300 | Secondary and unspec malig neop pectoral lymph nodes |
| 55463 | B561400 | Secondary and unspec malig neop post mediastinal lymph nodes |
| 52190 | B561900 | Secondary and unspec malig neop pulmonary lymph nodes |
| 47366 | B565300 | Secondary and unspec malig neop sacral lymph nodes |
| 39433 | B560500 | Secondary and unspec malig neop submandibular lymph nodes |
| 38343 | B560700 | Secondary and unspec malig neop submental lymph nodes |
| 67797 | B561600 | Secondary and unspec malig neop superfic tracheobronchial ln |
| 33395 | B560200 | Secondary and unspec malig neop superficial cervical ln |
| 54278 | B564000 | Secondary and unspec malig neop superficial inguinal ln |
| 72713 | B562100 | Secondary and unspec malig neop superficial mesenteric ln |
| 98626 | B563100 | Secondary and unspec malig neop supratrochlear lymph nodes |
| 66775 | B560100 | Secondary and unspec malignant neoplasm mastoid lymph nodes |
| 65253 | B560300 | Secondary and unspec malignant neoplasm occipital lymph node |
| 9618 | B56..00 | Secondary and unspecified malignant neoplasm of lymph nodes |
| 67129 | B560z00 | Secondary unspec malig neop lymph nodes head/face/neck nos |
| 66163 | ByuC200 | [X]2ndry+unspcf malignant neoplasm lymph nodes/multi regions |
| 13575 | 4F32.00 | Ascitic fluid: malignant cells |
| 4403 | B577.11 | Liver metastases |
| 8154 | B576200 | Malignant ascites |
| 6471 | B57..11 | Metastases of respiratory and/or digestive systems |
| 24301 | B57..12 | Secondary carcinoma of respiratory and/or digestive systems |
| 36200 | B575z00 | Secondary malig neop of large intestine or rectum nos |
| 35053 | B57..00 | Secondary malig neop of respiratory and digestive systems |
| 66083 | B57z.00 | Secondary malig neop of respiratory or digestive system nos |
| 67396 | B576.00 | Secondary malig neop of retroperitoneum and peritoneum |
| 97672 | B576z00 | Secondary malig neop of retroperitoneum or peritoneum nos |
| 70026 | B574z00 | Secondary malig neop of small intestine or duodenum nos |
| 28727 | B575000 | Secondary malignant neoplasm of colon |
| 55946 | B574000 | Secondary malignant neoplasm of duodenum |
| 99511 | B574200 | Secondary malignant neoplasm of ileum |
| 110433 | B574100 | Secondary malignant neoplasm of jejunum |
| 44529 | B575.00 | Secondary malignant neoplasm of large intestine and rectum |
| 36147 | B153.00 | Secondary malignant neoplasm of liver |
| 15103 | B577.00 | Secondary malignant neoplasm of liver |
| 112718 | B577000 | Secondary malignant neoplasm of liver intrahepatic bile duct |
| 4137 | B570.00 | Secondary malignant neoplasm of lung |
| 51551 | B571.00 | Secondary malignant neoplasm of mediastinum |
| 56345 | B57y.00 | Secondary malignant neoplasm of other digestive organ |
| 62584 | B573.00 | Secondary malignant neoplasm of other respiratory organs |
| 27391 | B576100 | Secondary malignant neoplasm of peritoneum |
| 16213 | B572.00 | Secondary malignant neoplasm of pleura |
| 62909 | B575100 | Secondary malignant neoplasm of rectum |
| 35364 | B576000 | Secondary malignant neoplasm of retroperitoneum |
| 64680 | B574.00 | Secondary malignant neoplasm of small intestine and duodenum |
| 17098 | BB83.00 | [M]pseudomyxoma peritonei |
| 57481 | ByuC300 | [X]secondary malignant neoplasm/oth+unspc respiratory organs |
| 88022 | ByuC400 | [X]secondary malignant neoplasm/oth+unspcfd digestive organs |
| 5199 | B583200 | Cerebral metastasis |
| 13569 | B590.00 | Disseminated malignancy nos |
| 7593 | H51y700 | Malignant pleural effusion |
| 8600 | 1D18.00 | Pain from metastases |
| 18676 | B585000 | Pathological fracture due to metastatic bone disease |
| 49341 | 4D56.00 | Pleural fluid: malignant cells |
| 101662 | B565200 | Secondary and unspec malig neop circumflex iliac ln |
| 97832 | B58y211 | Secondary cancer of the cervix |
| 65490 | B58y411 | Secondary cancer of the vulva |
| 27651 | B58..11 | Secondary carcinoma of other specified sites |
| 36401 | B587.00 | Secondary malignant neoplasm of adrenal gland |
| 22146 | B581100 | Secondary malignant neoplasm of bladder |
| 7654 | B585.00 | Secondary malignant neoplasm of bone and bone marrow |
| 5198 | B583000 | Secondary malignant neoplasm of brain |
| 33843 | B583.00 | Secondary malignant neoplasm of brain and spinal cord |
| 59375 | B583z00 | Secondary malignant neoplasm of brain or spinal cord nos |
| 16760 | B58y000 | Secondary malignant neoplasm of breast |
| 73616 | B58y200 | Secondary malignant neoplasm of cervix uteri |
| 104480 | B58y800 | Secondary malignant neoplasm of epididymis and vas deferens |
| 1952 | B580.00 | Secondary malignant neoplasm of kidney |
| 54120 | B584.00 | Secondary malignant neoplasm of other part of nervous system |
| 22524 | B58yz00 | Secondary malignant neoplasm of other specified site nos |
| 16500 | B58z.00 | Secondary malignant neoplasm of other specified site nos |
| 18616 | B58y.00 | Secondary malignant neoplasm of other specified sites |
| 5842 | B58..00 | Secondary malignant neoplasm of other specified sites |
| 62828 | B581z00 | Secondary malignant neoplasm of other urinary organ nos |
| 73213 | B581.00 | Secondary malignant neoplasm of other urinary organs |
| 44615 | B586.00 | Secondary malignant neoplasm of ovary |
| 49145 | B58y700 | Secondary malignant neoplasm of penis |
| 21590 | B58y500 | Secondary malignant neoplasm of prostate |
| 19945 | B582.00 | Secondary malignant neoplasm of skin |
| 55096 | B582z00 | Secondary malignant neoplasm of skin nos |
| 9505 | B582600 | Secondary malignant neoplasm of skin of breast |
| 100296 | B582100 | Secondary malignant neoplasm of skin of face |
| 43930 | B582000 | Secondary malignant neoplasm of skin of head |
| 48828 | B582500 | Secondary malignant neoplasm of skin of hip and leg |
| 35999 | B582200 | Secondary malignant neoplasm of skin of neck |
| 63896 | B582400 | Secondary malignant neoplasm of skin of shoulder and arm |
| 41144 | B582300 | Secondary malignant neoplasm of skin of trunk |
| 38918 | B583100 | Secondary malignant neoplasm of spinal cord |
| 34145 | B58y600 | Secondary malignant neoplasm of testis |
| 45824 | B58y900 | Secondary malignant neoplasm of tongue |
| 54679 | B594.00 | Secondary malignant neoplasm of unknown site |
| 60134 | B581000 | Secondary malignant neoplasm of ureter |
| 53528 | B581200 | Secondary malignant neoplasm of urethra |
| 55090 | B58y100 | Secondary malignant neoplasm of uterus |
| 70736 | B58y300 | Secondary malignant neoplasm of vagina |
| 60335 | B58y400 | Secondary malignant neoplasm of vulva |
| 5086 | D410.00 | Secondary polycythaemia |
| 15301 | D410z00 | Secondary polycythaemia nos |
| 48991 | 5136 | X-ray metastasis control |
| 5455 | BB53.00 | [M]adenocarcinoma, metastatic, nos |
| 3152 | BB13.00 | [M]carcinoma, metastatic, nos |
| 53694 | BB85111 | [M]krukenberg tumour |
| 54874 | BB85100 | [M]metastatic signet ring cell carcinoma |
| 3197 | BB03.00 | [M]neoplasm, metastatic |
| 34891 | BBF2.00 | [M]sarcomatosis nos |
| 9366 | BB13.11 | [M]secondary carcinoma |
| 6985 | BB03.11 | [M]secondary neoplasm |
| 24293 | BB2B.00 | [M]squamous cell carcinoma, metastatic nos |
| 97091 | ByuC500 | [X]2ndry malignant neoplasm/bladder+oth+unsp urinary organs |
| 68332 | ByuC600 | [X]2ndry malignant neoplasm/oth+unspec parts/nervous system |
| 54253 | ByuC700 | [X]secondary malignant neoplasm of other specified sites |
| 9965 | 8CM0.00 | Cancer care plan |
| 10334 | 8CP0.00 | Cancer care plan discussed with patient |
| 52401 | 8CP1.00 | Cancer care plan discussed with significant other |
| 19310 | 8BC3.00 | Cancer care plan given |
| 10652 | 8BAV.00 | Cancer care review |
| 54918 | Z4B3.00 | Cancer counselling |
| 54313 | 8O83.00 | Cancer emotional and psychosocial support and advice |
| 47018 | 677K.00 | Cancer home care pack given |
| 102339 | 9EV3.00 | Cancer multidisciplinary team report |
| 10332 | 9NX0.00 | Cancer primary healthcare multidisciplinary team |
| 26259 | 8CR8.00 | Cancer shared care medication card |
| 70300 | 9NX1.00 | Cancer supportive care worker |
| 2755 | B....11 | Cancers |
| 107734 | 7L1d200 | Deliver simple parenteral chemother neoplas first attendance |
| 110390 | 8Hgg.00 | Discharge from secondary care cancer service |
| 110366 | 8CS4000 | Establishing focus of care with specialist cancer team |
| 50371 | 9e00.00 | Gp out of hours service notified of cancer care plan |
| 102686 | 9NNc.00 | Has cancer key worker |
| 110537 | ZRVy.11 | Mac - mental adjustment to cancer scale |
| 70765 | ZRVy.00 | Mental adjustment to cancer scale |
| 27236 | 43mG.00 | Paraneoplastic antibody level |
| 90118 | 9OkC.00 | Patient on regional cancer register |
| 109656 | 8BAV000 | Prostate cancer care review |
| 10333 | 8HH8.00 | Referred to cancer primary healthcare multidisciplinary team |
| 23224 | 4M...00 | Tumour staging |
| 58167 | 9Nh1.00 | Under care cancer primary healthcare multidisciplinary team |
| 33508 | BB0z.00 | [M]unspecified tumour cell nos |
| 93228 | Fyu1300 | [X]paraneoplastic neuromyopathy and neuropathy |
| 46824 | 8H2G.00 | Admit radiotherapy emergency |
| 51959 | 8BAI.00 | Ambulatory chemotherapy |
| 110894 | SL07.00 | Antineoplastic antibiotic poisoning |
| 99865 | 9R59.00 | Awaiting chemotherapy |
| 36981 | 5155 | Awaiting radiotherapy |
| 62951 | 5A15.00 | Bone tumour/metast.irradiat. |
| 5019 | 8BAD000 | Cancer chemotherapy |
| 101693 | 8CRC.00 | Cancer chemotherapy management plan |
| 108081 | 7Q0J000 | Cancer hormonal treatment drugs band 1 |
| 783 | 8BAD.00 | Chemotherapy |
| 18079 | 8HB7.00 | Chemotherapy follow-up |
| 26149 | 8CV1.00 | Chemotherapy started |
| 107418 | 5A17.00 | Combined internal radiotherapy |
| 93602 | 7G0E200 | Combined photochemotherapy and uva light therapy to skin |
| 100865 | 7G0E300 | Combined photochemotherapy and uvb light therapy to skin |
| 28809 | 8BAM.00 | Combined post-operative chemotherapy and radiotherapy |
| 22490 | 8BAL.00 | Combined pre-operative chemotherapy and radiotherapy |
| 72978 | 597..00 | Combined radiotherapy |
| 64143 | 597Z.00 | Combined radiotherapy nos |
| 28071 | 7L10200 | Continuous infusion of chemotherapy |
| 18832 | 8BAa.00 | Date chemotherapy completed |
| 46028 | 8BAY.00 | Date chemotherapy stopped |
| 113376 | 7L1d100 | Deliv complex parenteral chemotherapy neopl first attendance |
| 105323 | 7M0c000 | Delivery fraction complex radiotherapy megavoltage machine |
| 108202 | 7M0c200 | Delivery fraction radiotherapy superficial orthovoltage mach |
| 113797 | 7M0c100 | Delivery fraction simple radiotherapy megavoltage machine |
| 91694 | 7L1Z300 | Delivery of a fraction of external beam radiotherapy nec |
| 103525 | 7L1Z200 | Delivery of a fraction of interstitial radiotherapy |
| 95009 | 7L1Z100 | Delivery of a fraction of intracavitary radiotherapy |
| 64997 | 7L1d.00 | Delivery of chemotherapy for neoplasm |
| 95424 | 7L1dz00 | Delivery of chemotherapy for neoplasm nos |
| 90743 | 7L1e.00 | Delivery of oral chemotherapy for neoplasm |
| 94431 | 7L1ez00 | Delivery of oral chemotherapy for neoplasm nos |
| 113708 | 7L1d300 | Delivery subsequent element cycle chemotherapy for neoplasm |
| 52006 | ZLE3100 | Discharge from radiotherapy service |
| 105336 | 7L1d400 | Electrochemotherapy |
| 51787 | 5975 | Ext.beam + chemotherapy |
| 69387 | 5974 | Ext.beam-surgery+chemotherapy |
| 91918 | 7272700 | External beam radiotherapy to lesion of retina |
| 1482 | 59...00 | External radiotherapy |
| 22472 | 59Z..00 | External radiotherapy nos |
| 72348 | Q007800 | Fetus/neonate affected-plac./breast transfer chemotherapy |
| 28061 | 5A16.11 | I131 radiotherapy |
| 37123 | 7L18200 | Intramuscular chemotherapy |
| 40490 | 7046200 | Intrathecal chemotherapy |
| 15386 | 7L16100 | Intravenous chemotherapy |
| 6153 | 5A16.12 | Iodine 131 radiotherapy |
| 28712 | 8J01.00 | Iodine seed radiotherapy |
| 104099 | 7M0c300 | Megavoltage treatment for complex radiotherapy |
| 100901 | 7M0c400 | Megavoltage treatment for simple radiotherapy |
| 104142 | 8BAl.00 | Neoadjuvant chemotherapy |
| 45087 | 8H3L.00 | Non-urgent radiotherapy admisn |
| 14887 | 8BA5.00 | Oral chemotherapy |
| 86329 | 7L1Z400 | Oral delivery of radiotherapy for thyroid ablation |
| 108561 | 7Q0J.00 | Other chemotherapy drugs |
| 42671 | 5A8..00 | Other radiotherapy misc. |
| 54919 | 5A8Z.00 | Other radiotherapy nos |
| 96310 | 7L1dy00 | Other specified delivery of chemotherapy for neoplasm |
| 111213 | 7Q0J200 | Other specified other chemotherapy drugs |
| 95126 | 7L1Zy00 | Other specified radiotherapy delivery |
| 70445 | 7M0cy00 | Other specified radiotherapy procedures |
| 95693 | 7L1Xy00 | Other specified radiotherapy volume definition |
| 105610 | 7M0ly00 | Other specified support for preparation for radiotherapy |
| 42854 | Z6E2.00 | Photochemotherapy |
| 88596 | 7272400 | Plaque radiotherapy to lesion of retina |
| 18675 | 8BAK.00 | Post-operartive chemotherapy |
| 44247 | C137200 | Post-radiotherapy hypopituitarism |
| 19467 | 8BAJ.00 | Pre-operative chemotherapy |
| 108140 | 7L1hz00 | Preparation for external beam radiotherapy nos |
| 74898 | Z9J1112 | Preparing chemotherapy |
| 89452 | 7L1b.00 | Procurement drugs for chemotherapy for neoplasm in bands 1-5 |
| 16935 | 515..00 | Progress of radiotherapy |
| 5333 | 65N..00 | Prophylactic chemotherapy |
| 34155 | 65NZ.00 | Prophylactic chemotherapy nos |
| 31527 | 514..00 | Purpose of radiotherapy |
| 73172 | 5A7Z.00 | Radio-chemotherapy nos |
| 103523 | TB12.00 | Radiological procedures/radiotherapy + complication,no blame |
| 88762 | 5135 | Radiological tumour control |
| 70290 | 5A7..00 | Radiomimetic chemotherapy |
| 108138 | 7L2..00 | Radiotherapy |
| 10776 | 5A...11 | Radiotherapy - internal |
| 29679 | 5146 | Radiotherapy - post-op.control |
| 43261 | 5144 | Radiotherapy - pre-op. Control |
| 55828 | 5145 | Radiotherapy -intra-op.control |
| 55261 | 5152 | Radiotherapy changed |
| 9706 | 5154 | Radiotherapy completed |
| 68423 | 7L1Z.00 | Radiotherapy delivery |
| 91891 | 7L1Zz00 | Radiotherapy delivery nos |
| 1009 | 8HB6.00 | Radiotherapy follow-up |
| 35597 | 5147 | Radiotherapy for analgesia |
| 55836 | 5148 | Radiotherapy for inflammation |
| 320 | 7M37100 | Radiotherapy nec |
| 93669 | 7L1Y.00 | Radiotherapy preparation |
| 107069 | 7L1Yz00 | Radiotherapy preparation nos |
| 31804 | TB12100 | Radiotherapy procedure with complication, without blame |
| 59684 | 7M0c.00 | Radiotherapy procedures |
| 91102 | 7M0cz00 | Radiotherapy procedures nos |
| 45099 | 515Z.00 | Radiotherapy progress nos |
| 29301 | 514Z.00 | Radiotherapy purpose - nos |
| 53453 | 8HJG.00 | Radiotherapy self-referral |
| 38466 | 5151 | Radiotherapy started |
| 44831 | 5153 | Radiotherapy stopped |
| 58036 | 7220100 | Radiotherapy to lacrimal gland |
| 68344 | 7244400 | Radiotherapy to lesion of cornea |
| 60674 | 7052200 | Radiotherapy to lesion of peripheral nerve |
| 20336 | 7272200 | Radiotherapy to lesion of retina |
| 41044 | 8J...00 | Radiotherapy treatment groups |
| 29285 | 5149 | Radiotherapy-tumour palliation |
| 10932 | 8H67.00 | Referred for radiotherapy |
| 10346 | 9N0D.00 | Seen in radiotherapy clinic |
| 30942 | 9N1yC00 | Seen in radiotherapy clinic |
| 101391 | 7809300 | Selective internal radiotherapy microspheres lesion of liver |
| 18715 | 5AB..00 | Stereotactic radiotherapy |
| 30264 | 7L19300 | Subcutaneous chemotherapy |
| 98902 | 7M0c500 | Superficial or orthovoltage treatment for radiotherapy |
| 103872 | 7M0l.00 | Support for preparation for radiotherapy |
| 67248 | 5A12.00 | Thyroid tumour/metast irradiat |
| 97154 | 5137 | X-ray radiotherapy control |
| 20443 | ZV67200 | [V]chemotherapy follow-up |
| 31489 | ZV58800 | [V]chemotherapy session for neoplasm |
| 35609 | ZV66200 | [V]convalescence after chemotherapy |
| 16662 | ZV66100 | [V]convalescence after radiotherapy |
| 60682 | ZV6B100 | [V]follow-up exam after chemotherapy for other conditions |
| 30547 | ZV67700 | [V]follow-up exam after radiotherapy for malignant neoplasm |
| 30425 | ZV6B000 | [V]follow-up exam after radiotherapy for other conditions |
| 25479 | ZV67800 | [V]follow-up examin after chemotherapy for malign neoplasm |
| 53477 | ZV67811 | [V]follow-up examination after chemotherapy for leukaemia |
| 20381 | ZV58100 | [V]maintenance chemotherapy |
| 45500 | ZV07300 | [V]other prophylactic chemotherapy |
| 18904 | ZV67100 | [V]radiotherapy follow-up |
| 5527 | ZV58000 | [V]radiotherapy session |
| 95851 | U603311 | [X] adverse reaction to antineoplastic antibiotics |
| 98625 | U603100 | [X]antineoplast antimetabs caus adverse eff in therap use |
| 52984 | ZVu1B00 | [X]need for other prophylactic chemotherapy |
| 71837 | U603300 | [X]other antineoplast drugs caus adverse eff in therap use |
| 51781 | ZVu3L00 | [X]other chemotherapy |

*Note: ICD-10 codes C18,19 ad C20 were also used as an exclusion criteria from the control group

## End stage kidney disease (medcode, Read)

| medcode | readcode | readterm |
| --- | --- | --- |
| 49028 | 14S2.00 | H/O: kidney recipient |
| 2997 | 7B00.00 | Transplantation of kidney |
| 55151 | 7B00000 | Autotransplant of kidney |
| 11745 | 7B00100 | Transplantation of kidney from live donor |
| 66705 | 7B00111 | Allotransplantation of kidney from live donor |
| 24361 | 7B00200 | Transplantation of kidney from cadaver |
| 98364 | 7B00211 | Allotransplantation of kidney from cadaver |
| 105328 | 7B00212 | Cadaveric renal transplant |
| 89924 | 7B00300 | Allotransplantation of kidney from cadaver, heart-beating |
| 96133 | 7B00400 | Allotransplantation kidney from cadaver, heart non-beating |
| 109455 | 7B00500 | Allotransplantation of kidney from cadaver NEC |
| 105787 | 7B00600 | Xenograft renal transplant |
| 70874 | 7B00y00 | Other specified transplantation of kidney |
| 5504 | 7B00z00 | Transplantation of kidney NOS |
| 51039 | 7B01200 | Bilateral nephrectomy |
| 48121 | 7B01500 | Transplant nephrectomy |
| 72004 | 7B01511 | Excision of rejected transplanted kidney |
| 26862 | 7B06300 | Exploration of renal transplant |
| 93366 | 7B0F.00 | Interventions associated with transplantation of kidney |
| 90952 | 7B0F100 | Pre-transplantation of kidney work-up, recipient |
| 103429 | 7B0F300 | Post-transplantation of kidney examination, recipient |
| 104050 | 7B0Fy00 | OS interventions associated with transplantation of kidney |
| 104049 | 7B0Fz00 | Interventions associated with transplantation of kidney NOS |
| 17253 | 8L50.00 | Renal transplant planned |
| 100693 | Kyu1C00 | [X]Renal tubulo-interstitial disorders/transplant rejection |
| 70712 | SP08011 | Det.ren.func.after ren.transpl |
| 11553 | SP08300 | Kidney transplant failure and rejection |
| 104905 | SP08D00 | Acute-on-chronic rejection of renal transplant |
| 104960 | SP08E00 | Acute rejection of renal transplant - grade I |
| 107000 | SP08F00 | Acute rejection of renal transplant - grade II |
| 104630 | SP08G00 | Acute rejection of renal transplant - grade III |
| 104201 | SP08H00 | Acute rejection of renal transplant |
| 106620 | SP08J00 | Chronic rejection of renal transplant |
| 105724 | SP08N00 | Unexplained episode of renal transplant dysfunction |
| 106301 | SP08P00 | Stenosis of vein of transplanted kidney |
| 105811 | SP08R00 | Renal transplant rejection |
| 107752 | SP08T00 | Urological complication of renal transplant |
| 108437 | SP08V00 | Very mild acute rejection of renal transplant |
| 106866 | SP08W00 | Vascular complication of renal transplant |
| 54990 | TB00100 | Kidney transplant with complication, without blame |
| 18774 | TB00111 | Renal transplant with complication, without blame |
| 5911 | ZV42000 | [V]Kidney transplanted |
| 24151 | 7A60.00 | Arteriovenous shunt |
| 63063 | 7A60000 | Insertion of arteriovenous prosthesis |
| 3205 | 7A60100 | Creation of arteriovenous fistula NEC |
| 28269 | 7A60111 | Creation of radial-cephalic fistula |
| 25521 | 7A60112 | Creation of brachial-cephalic fistula |
| 63190 | 7A60200 | Attention to arteriovenous shunt |
| 31478 | 7A60300 | Removal of infected arteriovenous shunt |
| 96131 | 7A60400 | Banding of arteriovenous fistula |
| 63305 | 7A60500 | Thrombectomy of arteriovenous fistula |
| 60302 | 7A60600 | Creation of graft fistula for dialysis |
| 65398 | 7A60y00 | Other specified arteriovenous shunt |
| 58618 | 7A60z00 | Arteriovenous shunt NOS |
| 18779 | 7A61100 | Repair of acquired arteriovenous fistula |
| 100235 | 7A61111 | Ligation of acquired arteriovenous fistula |
| 9765 | 7A61400 | Ligation of acquired arteriovenous fistula |
| 96347 | 7A61900 | Ligation of arteriovenous dialysis fistula |
| 107719 | 7A61A00 | Ligation of arteriovenous dialysis graft |
| 31549 | 7L1A.00 | Compensation for renal failure |
| 11773 | 7L1A.11 | Dialysis for renal failure |
| 20073 | 7L1A000 | Renal dialysis |
| 101756 | 7L1A011 | Thomas intravascular shunt for dialysis |
| 2994 | 7L1A100 | Peritoneal dialysis |
| 2996 | 7L1A200 | Haemodialysis NEC |
| 71124 | 7L1A300 | Haemofiltration |
| 88597 | 7L1A400 | Automated peritoneal dialysis |
| 30756 | 7L1A500 | Continuous ambulatory peritoneal dialysis |
| 64828 | 7L1A600 | Peritoneal dialysis NEC |
| 48022 | 7L1Ay00 | Other specified compensation for renal failure |
| 64636 | 7L1Az00 | Compensation for renal failure NOS |
| 56760 | 7L1B.00 | Placement ambulatory apparatus compensation renal failure |
| 36442 | 7L1B.11 | Placement ambulatory dialysis apparatus - compens renal fail |
| 8037 | 7L1B000 | Insertion of ambulatory peritoneal dialysis catheter |
| 23773 | 7L1B100 | Removal of ambulatory peritoneal dialysis catheter |
| 104586 | 7L1B200 | Flushing of peritoneal dialysis catheter |
| 59194 | 7L1By00 | Placement ambulatory apparatus- compensate renal failure OS |
| 83513 | 7L1C.00 | Placement other apparatus for compensation for renal failure |
| 30709 | 7L1C000 | Insertion of temporary peritoneal dialysis catheter |
| 107901 | 7L1Cy00 | Placement other apparatus- compensate for renal failure OS |
| 65089 | 7L1Cz00 | Placement other apparatus- compensate for renal failure NOS |
| 102210 | 7L1f000 | Extracorporeal albumin haemodialysis |
| 105760 | G72C.00 | Ruptured aneurysm of dialysis vascular access |
| 107188 | G72D.00 | Aneurysm of dialysis arteriovenous fistula |
| 110095 | G72D000 | Aneurysm of superficialised artery of dialysis AV fistula |
| 107220 | G72D100 | Aneurysm of needle site of dialysis arteriovenous fistula |
| 105742 | G72D200 | Aneurysm of anastomotic site of dialysis AV fistula |
| 2995 | G760.00 | Acquired arteriovenous fistula |
| 107746 | Gy1..00 | Stenosis of dialysis vascular access |
| 108699 | Gy10.00 | Stenosis of dialysis arteriovenous graft |
| 109809 | Gy2..00 | Thrombosis of dialysis vascular access |
| 106720 | Gy21.00 | Thrombosis of dialysis arteriovenous fistula |
| 108116 | Gy3..00 | Occlusion of dialysis vascular access |
| 109135 | Gy30.00 | Occlusion of dialysis arteriovenous graft |
| 107082 | Gy31.00 | Occlusion of dialysis arteriovenous fistula |
| 110051 | Gy4..00 | Infection of dialysis vascular access |
| 108213 | Gy40.00 | Infection of dialysis arteriovenous graft |
| 107260 | Gy41.00 | Infection of dialysis arteriovenous fistula |
| 108759 | Gy5..00 | Haemorrhage of dialysis vascular access |
| 106975 | Gy51.00 | Haemorrhage of dialysis arteriovenous fistula |
| 108423 | Gy60.00 | Rupture of dialysis arteriovenous graft |
| 107900 | SP0E.00 | Disorders associated with peritoneal dialysis |
| 111103 | SP0E100 | Thrombus in peritoneal dialysis catheter |
| 108785 | SP0F.00 | Haemodialysis first use syndrome |
| 105436 | SP0G.00 | Anaphylactoid reaction due to haemodialysis |
| 109884 | SP0H.00 | Disorder associated with dialysis |
| 96184 | TA02000 | Accid cut puncture perf h'ge - kidney dialysis |
| 69427 | TA02z00 | Accid cut puncture perf h'ge - perfusion NOS |
| 35921 | TA22.00 | Failure of sterile precautions during perfusion |
| 69266 | TA22000 | Failure of sterile precautions during kidney dialysis |
| 111637 | TA42000 | Mechanical failure of apparatus during kidney dialysis |
| 28158 | TB11.00 | Kidney dialysis with complication without blame |
| 66714 | TB11.11 | Renal dialysis with complication without blame |
| 54844 | U612200 | [X]Failure sterile precautions dur kidney dialys/other perf |
| 110072 | Z919200 | Washing back through haemodialysis lines |
| 22252 | ZV45100 | [V]Renal dialysis status |
| 60743 | ZV56.00 | [V]Aftercare involving intermittent dialysis |
| 46145 | ZV56011 | [V]Aftercare involving renal dialysis NOS |
| 69760 | ZVu3G00 | [X]Other dialysis |

## End stage kidney disease (ICD-10)

| ICD code | description |
| --- | --- |
| I77.0 | arteriovenous fistula, acquired |
| N16.5 | renal tubulo-interstitial disorders in transplant rejection |
| N18.5 | chronic kidney disease, stage 5 |
| T82.4 | mechanical complication of vascular dialysis catheter |
| T86.1 | kidney transplant failure and rejection |
| Y60.2 | during kidney dialysis or other perfusion |
| Y61.2 | during kidney dialysis or other perfusion |
| Y62.2 | during kidney dialysis or other perfusion |
| Y84.1 | kidney dialysis |
| Z49 | care involving dialysis |
| Z49.0 | preparatory care for dialysis |
| Z49.1 | extracorporeal dialysis |
| Z49.2 | other dialysis |
| Z94.0 | kidney transplant status |
| Z99.2 | dependence on renal dialysis |

# Outcome

## AKI (ICD-10)

| icdcode | description |
| --- | --- |
| N14 | Drug- and heavy-metal-induced tubulo-interstitial and tubular conditions |
| N14.1 | Nephropathy induced by other drugs, medicaments and biological substances |
| N14.2 | Nephropathy induced by unspecified drug, medicament or biological substance |
| N17 | Acute renal failure |
| N17.0 | Acute renal failure with tubular necrosis |
| N17.1 | Acute renal failure with acute cortical necrosis |
| N17.2 | Acute renal failure with medullary necrosis |
| N17.8 | Other acute renal failure |
| N17.9 | Acute renal failure, unspecified |
| N19 | Unspecified kidney failure |
| N99.0 | Postprocedural renal failure |
| R34 | Anuria and oliguria |
| R94.4 | Abnormal results of kidney function studies |

# Baseline Variables

## Smoking (medcode, Read)

| medcode | readterm | smokstatus |
| --- | --- | --- |
| 33 | Never smoked tobacco | non-smoker |
| 60 | Current non-smoker | non-smoker |
| 90 | Ex smoker | ex-smoker |
| 93 | Cigarette smoker | current smoker |
| 776 | Stopped smoking | ex-smoker |
| 1822 | Very heavy smoker - 40+cigs/d | current smoker |
| 1823 | Smoker | current smoker |
| 1878 | Moderate smoker - 10-19 cigs/d | current smoker |
| 2111 | Health ed. - smoking | current smoker |
| 3568 | Heavy smoker - 20-39 cigs/day | current smoker |
| 6359 | Nicotine withdrawal | current or ex-smoker |
| 7622 | Smoking cessation advice | current or ex-smoker |
| 9045 | Advice on smoking | current smoker |
| 9833 | Nicotine replacement therapy | current or ex-smoker |
| 10184 | Pregnancy smoking advice | current smoker |
| 10211 | Smoking cessation milestones | ex-smoker |
| 10558 | Current smoker | current smoker |
| 10742 | Referral to stop-smoking clinic | current or ex-smoker |
| 10898 | Smoking free weeks | ex-smoker |
| 11356 | Seen by smoking cessation advisor | current or ex-smoker |
| 11527 | DNA - Did not attend smoking cessation clinic | current smoker |
| 11713 | Pack years | current or ex-smoker |
| 11788 | Non-smoker | non-smoker |
| 12240 | Trying to give up smoking | current or ex-smoker |
| 12878 | Date ceased smoking | ex-smoker |
| 12941 | Occasional smoker | current smoker |
| 12942 | Smoker - amount smoked | current smoker |
| 12943 | Cigar smoker | current smoker |
| 12944 | Light smoker - 1-9 cigs/day | current smoker |
| 12945 | Rolls own cigarettes | current smoker |
| 12946 | Ex-smoker - amount unknown | ex-smoker |
| 12947 | Pipe smoker | current smoker |
| 12951 | Smoking restarted | current smoker |
| 12952 | Smoking started | current smoker |
| 12953 | Attends stop smoking monitor. | current or ex-smoker |
| 12954 | [V]Tobacco use | current smoker |
| 12955 | Ex-moderate smoker (10-19/day) | ex-smoker |
| 12956 | Ex-heavy smoker (20-39/day) | ex-smoker |
| 12957 | Ex-light smoker (1-9/day) | ex-smoker |
| 12958 | Trivial smoker - < 1 cig/day | current smoker |
| 12959 | Ex-very heavy smoker (40+/day) | ex-smoker |
| 12960 | Tobacco consumption NOS | current smoker |
| 12961 | Ex-trivial smoker (<1/day) | ex-smoker |
| 12963 | Cigar consumption | current smoker |
| 12964 | Keeps trying to stop smoking | current smoker |
| 12965 | Cigarette consumption | current smoker |
| 12966 | Smoking reduced | current smoker |
| 12967 | Pipe tobacco consumption | current smoker |
| 13351 | Passive smoker | non-smoker |
| 16717 | Smokers' cough | current smoker |
| 18573 | Referral to smoking cessation advisor | current or ex-smoker |
| 18926 | Lifestyle advice regarding smoking | current smoker |
| 19485 | Stop smoking monitor.chck done | current or ex-smoker |
| 19488 | Ex cigar smoker | ex-smoker |
| 21637 | Stop smoking monitor admin.NOS | current or ex-smoker |
| 24529 | Nicotine replacement therapy refused | current smoker |
| 25106 | Nicotine replacement therapy provided free | current or ex-smoker |
| 26470 | Ex pipe smoker | ex-smoker |
| 28834 | Anti-smoking monitoring admin. | current or ex-smoker |
| 30423 | Thinking about stopping smoking | current smoker |
| 30762 | Not interested in stopping smoking | current smoker |
| 31114 | Ready to stop smoking | current smoker |
| 32083 | Stop smoking clinic admin. | current or ex-smoker |
| 32572 | Over the counter nicotine replacement therapy | current or ex-smoker |
| 32687 | Tobacco dependence | current smoker |
| 34126 | Negotiated date for cessation of smoking | current smoker |
| 35055 | [V]Tobacco abuse counselling | current smoker |
| 38112 | Smoking cessation programme start date | current smoker |
| 40417 | Stop smoking monitor default | current or ex-smoker |
| 40418 | Refuses stop smoking monitor | current smoker |
| 41042 | Smoking cessation advice provided by community pharmacist | current or ex-smoker |
| 41979 | Smoking restarted | current smoker |
| 42288 | Pack years | current or ex-smoker |
| 42722 | Stop smoking monitor 1st lettr | current or ex-smoker |
| 43433 | Toxic effect of tobacco and nicotine | current smoker |
| 46300 | Cigarette pack-years | current or ex-smoker |
| 46321 | Reason for restarting smoking | current smoker |
| 47273 | Motives for smoking scale | current smoker |
| 49418 | RFS - Reasons for smoking scale | current smoker |
| 52503 | No smokers in the household | non-smoker |
| 53101 | Stop smoking monitor verb.inv. | current or ex-smoker |
| 56144 | [X]Mental and behav dis due to use of tobacco: harmful use | current smoker |
| 57639 | Bupropion refused | current smoker |
| 58597 | Stop smoking monitor phone inv | current or ex-smoker |
| 59866 | Reasons for smoking scale | current smoker |
| 60720 | Stop smoking monitor 2nd lettr | current or ex-smoker |
| 61905 | [X]Mental and behavioural disorder due to use of tobacco | current smoker |
| 62686 | Minutes from waking to first tobacco consumption | current smoker |
| 63016 | [X]Bupropion causing adverse effects in therapeutic use | current or ex-smoker |
| 63299 | FTND - Fagerstrom test for nicotine dependence | current smoker |
| 63666 | Fagerstrom test for nicotine dependence | current smoker |
| 63717 | Bupropion contraindicated | current or ex-smoker |
| 63901 | Stop smoking monitoring delete | current or ex-smoker |
| 66387 | Stop smoking monitor 3rd lettr | current or ex-smoker |
| 66409 | Nicotine replacement therapy contraindicated | current or ex-smoker |
| 67178 | Nicotine replacement therapy provided by community pharmacis | current or ex-smoker |
| 68658 | Tobacco dependence NOS | current smoker |
| 70746 | Tobacco dependence, continuous | current smoker |
| 72700 | [V]Personal history of tobacco abuse | current or ex-smoker |
| 72706 | Tobacco dependence in remission | ex-smoker |
| 74907 | Smoking cessation therapy | current or ex-smoker |
| 81440 | Nicotine replacement therapy using nicotine patches | current or ex-smoker |
| 85247 | Nicotine replacement therapy using nicotine inhalator | current or ex-smoker |
| 85975 | Nicotine replacement therapy using nicotine gum | current or ex-smoker |
| 89464 | Nicotine replacement therapy using nicotine lozenges | current or ex-smoker |
| 90522 | Smoking cessation therapy NOS | current or ex-smoker |
| 91513 | Occasions for smoking scale | current smoker |
| 91708 | Other specified smoking cessation therapy | current or ex-smoker |
| 94958 | Smoking cessation drug therapy | current or ex-smoker |
| 95610 | Tobacco dependence, unspecified | current smoker |
| 96992 | Smoking cessation - enhanced services administration | current or ex-smoker |
| 97210 | Ex-cigarette smoker | ex-smoker |
| 97643 | Fagerstrom test for nicotine dependence | current smoker |
| 97973 | Maternal tobacco abuse | current smoker |
| 98137 | Brief intervention for smoking cessation | current or ex-smoker |
| 98154 | Referral to NHS stop smoking service | current or ex-smoker |
| 98177 | Non-smoker annual review - enhanced services administration | non-smoker |
| 98245 | Stop smoking face to face follow-up | current or ex-smoker |
| 98347 | Current smoker annual review - enhanced services admin | current smoker |
| 98447 | Ex-smoker annual review - enhanced services administration | ex-smoker |
| 98493 | Smoking cessatn monitor template complet - enhanc serv admin | current or ex-smoker |
| 99838 | Recently stopped smoking | ex-smoker |
| 100099 | Smoking cessation advice declined | current or ex-smoker |
| 100495 | Ex roll-up cigarette smoker | ex-smoker |
| 100963 | Ex-smoker annual review | ex-smoker |
| 101210 | Consent given for smoking cessation data sharing | current or ex-smoker |
| 101325 | Declin cons follow-up evaluation after smoking cess interven | current or ex-smoker |
| 101338 | Failed attempt to stop smoking | current smoker |
| 101385 | Consent given for follow-up by smoking cessation team | current or ex-smoker |
| 101634 | Consent given follow-up after smoking cessation intervention | current or ex-smoker |
| 101764 | Practice based smoking cessation programme start date | current or ex-smoker |
| 101851 | Declined consent for follow-up by smoking cessation team | current or ex-smoker |
| 101854 | Declined consent for smoking cessation data sharing | current or ex-smoker |
| 101878 | Non-smoker annual review | non-smoker |
| 102361 | Referral for smoking cessation service offered | current or ex-smoker |
| 102951 | Lost to smoking cessation follow-up | current or ex-smoker |
| 103507 | Stop smoking service opportunity signposted | current or ex-smoker |
| 104086 | Stop smoking invitation first SMS text message | current or ex-smoker |
| 104185 | Smoking cessation drug therapy declined | current or ex-smoker |
| 104230 | Smoking cessation programme declined | current or ex-smoker |
| 104310 | Current smoker annual review | current smoker |
| 105572 | Stop smoking invitation short message service text message | current or ex-smoker |
| 105710 | Smoking cessation 12 week follow-up | current or ex-smoker |
| 106359 | Referral to smoking cessation service | current or ex-smoker |
| 106384 | Stop smoking invitation second SMS text message | current or ex-smoker |
| 106385 | Stop smoking invitation third SMS text message | current or ex-smoker |
| 106391 | Referral to smoking cessation service declined | current or ex-smoker |
| 107792 | [X]Mental and behav dis due to use tobacco: dependence syndr | current smoker |
| 108503 | Ex user of electronic cigarette | current or ex-smoker |
| 108966 | Smoking cessation ESA monitoring template completed | current or ex-smoker |
| 109716 | Issue of nicotine replacement therapy voucher | current or ex-smoker |
| 110692 | Varenicline smoking cessation therapy offered | current or ex-smoker |
| 111853 | [X]Mental & behav dis due to use tobacco: acute intoxication | current smoker |
| 112529 | [X]Mental & behav dis due to use tobacco: psychotic disorder | current smoker |

## Alcohol use (medcode, Read)

| **medcode** | **readterm** | **alcstatus** | **alclevel** |
| --- | --- | --- | --- |
| 12975 | Trivial drinker - <1u/day | curr | L |
| 385 | Drinks rarely | curr | L |
| 749 | Drinks occasionally | curr | L |
| 12972 | Light drinker - 1-2u/day | curr | L |
| 322 | Moderate drinker - 3-6u/day | curr | M |
| 1618 | Heavy drinker - 7-9u/day | curr | H |
| 12977 | Very heavy drinker - >9u/day | curr | H |
| 12976 | Suspect alcohol abuse - denied | curr | H |
| 12971 | Spirit drinker | curr |  |
| 2689 | Beer drinker | curr |  |
| 12968 | Drinks beer and spirits | curr |  |
| 12969 | Drinks wine | curr |  |
| 956 | Social drinker | curr | L |
| 12982 | Alcohol intake above recommended sensible limits | curr | H |
| 26472 | Alcohol intake within recommended sensible limits | curr | L |
| 12980 | Light drinker | curr | L |
| 12985 | Moderate drinker | curr | M |
| 8999 | Heavy drinker | curr | H |
| 12984 | Very heavy drinker | curr | H |
| 19401 | Binge drinker | curr | H |
| 19494 | Hazardous alcohol use | curr | H |
| 30695 | Harmful alcohol use | curr | H |
| 94670 | Alcohol misuse | curr | H |
| 101718 | Drinks in morning to get rid of hangover | curr |  |
| 102665 | Increasing risk drinking | curr |  |
| 99877 | Feels should cut down drinking | curr | H |
| 102448 | Higher risk drinking | curr |  |
| 103230 | Lower risk drinking | curr |  |
| 18156 | Alcoholics anonymous | curr | H |
| 84218 | Disqualified from driving due to excess alcohol | curr | H |
| 38061 | Alcohol induced hallucinations | curr | H |
| 44783 | Pain in lymph nodes after alcohol consumption | curr |  |
| 24735 | O/E - breath - alcohol smell | curr | H |
| 10161 | O/E - alcoholic breath | curr | H |
| 11491 | Health ed. - alcohol | curr |  |
| 18711 | Lifestyle advice regarding alcohol | curr | H |
| 56410 | Delivery of rehabilitation for alcohol addiction | curr | H |
| 2083 | Alcohol detoxification | curr | H |
| 110624 | Alcohol relapse prevention | curr | H |
| 110494 | Alcohol harm reduction programme | curr | H |
| 7692 | Patient advised about alcohol | curr | H |
| 102564 | Advised to abstain from alcohol consumption | curr |  |
| 97309 | Advised to contact primary care alcohol worker | curr | H |
| 37264 | Alcohol leaflet given | curr | H |
| 29691 | Aversion therapy - alcoholism | curr | H |
| 21650 | Admitted to alcohol detoxification centre | curr | H |
| 9849 | Referral to community alcohol team | curr | H |
| 12554 | Referral to community drug and alcohol team | curr | H |
| 94553 | Referral to specialist alcohol treatment service | curr | H |
| 96993 | Referral to alcohol brief intervention service | curr | H |
| 97261 | Brief intervention for excessive alcohol consumptn declined | curr | H |
| 97680 | Declined referral to specialist alcohol treatment service | curr | H |
| 102247 | Extended interven for excessive alcohol consumption declined | curr | H |
| 103459 | Referral to community alcohol team declined | curr | H |
| 109241 | Alcohol Use Disorders Identification Test declined | curr | H |
| 109105 | Refer to MH services deferred until alcohol misuse resolved | curr | H |
| 108644 | In-house alcohol detoxification | curr | H |
| 9489 | Under care of community alcohol team | curr | H |
| 110382 | Withdrawn from alcohol detoxification programme | curr |  |
| 109800 | Hospital attendance related to personal alcohol consumption | curr | H |
| 11740 | Alcohol misuse - enhanced services administration | curr | H |
| 35330 | Alcohol consumption counselling | curr | H |
| 63529 | Alcohol misuse - enhanced service completed | curr | H |
| 47123 | Alcohol counselling by other agencies | curr | H |
| 96053 | Brief intervention for excessive alcohol consumptn completed | curr | H |
| 96054 | Extended intervention for excessive alcohol consumptn complt | curr | H |
| 65754 | Alcohol-induced pseudo-Cushing's syndrome | curr | H |
| 16237 | Alcoholic psychoses | curr | H |
| 16225 | Alcohol withdrawal delirium | curr | H |
| 22277 | DTs - delirium tremens | curr | H |
| 1476 | Delirium tremens | curr | H |
| 20762 | Alcohol amnestic syndrome | curr | H |
| 4500 | Korsakov's alcoholic psychosis | curr | H |
| 11106 | Korsakov's alcoholic psychosis with peripheral neuritis | curr | H |
| 18636 | Wernicke-Korsakov syndrome | curr | H |
| 41920 | Alcohol amnestic syndrome NOS | curr | H |
| 54505 | Other alcoholic dementia | curr | H |
| 27342 | Alcoholic dementia NOS | curr | H |
| 37946 | Chronic alcoholic brain syndrome | curr | H |
| 25110 | Alcohol withdrawal hallucinosis | curr | H |
| 57939 | Pathological alcohol intoxication | curr | H |
| 20407 | Drunkenness - pathological | curr | H |
| 30404 | Alcoholic paranoia | curr | H |
| 33670 | Other alcoholic psychosis | curr | H |
| 2082 | Alcohol withdrawal syndrome | curr | H |
| 68111 | Other alcoholic psychosis NOS | curr | H |
| 67651 | Alcoholic psychosis NOS | curr | H |
| 2084 | Alcohol dependence syndrome | curr | H |
| 2081 | Alcoholism | curr | H |
| 1399 | Alcohol problem drinking | curr | H |
| 5740 | Acute alcoholic intoxication in alcoholism | curr | H |
| 57714 | Alcohol dependence with acute alcoholic intoxication | curr | H |
| 40530 | Acute alcoholic intoxication, unspecified, in alcoholism | curr | H |
| 56947 | Continuous acute alcoholic intoxication in alcoholism | curr | H |
| 21624 | Episodic acute alcoholic intoxication in alcoholism | curr | H |
| 59574 | Acute alcoholic intoxication in remission, in alcoholism | curr | H |
| 36296 | Acute alcoholic intoxication in alcoholism NOS | curr | H |
| 31443 | Chronic alcoholism | curr | H |
| 43193 | Unspecified chronic alcoholism | curr | H |
| 24064 | Continuous chronic alcoholism | curr | H |
| 26106 | Episodic chronic alcoholism | curr | H |
| 33635 | Chronic alcoholism NOS | curr | H |
| 6169 | Alcohol dependence syndrome NOS | curr | H |
| 7746 | Nondependent alcohol abuse | curr |  |
| 27518 | Hangover (alcohol) | curr |  |
| 3782 | Intoxication - alcohol | curr |  |
| 669 | Nondependent alcohol abuse, unspecified | curr | H |
| 23610 | Nondependent alcohol abuse, continuous | curr | H |
| 12974 | Nondependent alcohol abuse, episodic | curr | H |
| 31569 | Nondependent alcohol abuse in remission | curr | H |
| 28150 | Nondependent alcohol abuse NOS | curr | H |
| 5611 | [X]Mental and behavioural disorders due to use of alcohol | curr | H |
| 44299 | [X]Mental & behav dis due to use alcohol: acute intoxication | curr | H |
| 9508 | [X]Acute alcoholic drunkenness | curr | H |
| 21879 | [X]Mental and behav dis due to use of alcohol: harmful use | curr | H |
| 39327 | [X]Mental and behav dis due to use alcohol: dependence syndr | curr | H |
| 28780 | [X]Alcohol addiction | curr | H |
| 5758 | [X]Chronic alcoholism | curr | H |
| 20514 | [X]Mental and behav dis due to use alcohol: withdrawal state | curr | H |
| 17259 | [X]Delirium tremens, alcohol induced | curr | H |
| 12353 | [X]Mental & behav dis due to use alcohol: psychotic disorder | curr | H |
| 6467 | [X]Alcoholic hallucinosis | curr | H |
| 65932 | [X]Alcoholic jealousy | curr | H |
| 30162 | [X]Alcoholic paranoia | curr | H |
| 17607 | [X]Alcoholic psychosis NOS | curr | H |
| 39799 | [X]Mental and behav dis due to use alcohol: amnesic syndrome | curr | H |
| 11670 | [X]Korsakov's psychosis, alcohol induced | curr | H |
| 26323 | [X]Alcoholic dementia NOS | curr | H |
| 37691 | [X]Chronic alcoholic brain syndrome | curr | H |
| 32927 | [X]Alcohol withdrawal-induced seizure | curr | H |
| 45169 | [X]Men & behav dis due to use alcohol: oth men & behav dis | curr | H |
| 64389 | [X]Ment & behav dis due use alcohol: unsp ment & behav dis | curr | H |
| 47555 | Cerebral degeneration due to alcoholism | curr | H |
| 36748 | Alcoholic encephalopathy | curr | H |
| 33839 | Cerebellar ataxia due to alcoholism | curr | H |
| 30604 | Alcohol-induced epilepsy | curr | H |
| 2925 | Alcoholic polyneuropathy | curr | H |
| 31742 | Alcoholic myopathy | curr | H |
| 4915 | Alcoholic cardiomyopathy | curr | H |
| 8363 | Oesophageal varices in alcoholic cirrhosis of the liver | curr | H |
| 4506 | Alcoholic gastritis | curr | H |
| 10691 | Alcoholic fatty liver | curr | H |
| 3216 | Acute alcoholic hepatitis | curr | H |
| 4743 | Alcoholic cirrhosis of liver | curr | H |
| 21713 | Alcoholic fibrosis and sclerosis of liver | curr | H |
| 7885 | Alcoholic liver damage unspecified | curr | H |
| 17330 | Alcoholic hepatic failure | curr | H |
| 7943 | Alcoholic hepatitis | curr | H |
| 7602 | Chronic alcoholic hepatitis | curr | H |
| 42843 | Other non-alcoholic chronic liver disease | curr | H |
| 104611 | Alcohol-induced acute pancreatitis | curr | H |
| 24984 | Alcohol-induced chronic pancreatitis | curr | H |
| 66019 | Suspect fetal damage from maternal alcohol | curr | H |
| 27670 | Maternal care for (suspected) damage to fetus from alcohol | curr | H |
| 23945 | Fetal alcohol syndrome | curr | H |
| 66699 | Fetus and newborn affected by maternal use of alcohol | curr | H |
| 97916 | Fetal alcohol syndrome | curr | H |
| 9169 | [D]Alcohol blood level excessive | curr | H |
| 19217 | Alcohol causing toxic effect | curr | H |
| 110911 | Accidental poisoning by denatured alcohol | curr | H |
| 23978 | [X]Evid of alcohol involv determind by level of intoxication | curr |  |
| 41983 | Alcohol detoxification | curr | H |
| 46677 | Alcohol withdrawal regime | curr | H |
| 61383 | Planned reduction of alcohol consumption | curr | H |
| 95181 | Alcohol reduction programme | curr | H |
| 110671 | Controlled drinking regime | curr | M |
| 64409 | Self-monitoring of alcohol intake | curr | H |
| 30460 | Alcoholism counselling | curr | H |
| 111683 | Removal of alcohol | curr |  |
| 95650 | Advice to change drink intake | curr | H |
| 97163 | Advice to change alcoholic drink intake | curr | H |
| 54209 | Advice to change alcohol intake | curr | H |
| 104109 | Drinks only | curr |  |
| 11140 | Advice on alcohol consumption | curr | H |
| 22707 | Drinking problem scale | curr | H |
| 46848 | DPS - Drinking problem scale | curr | H |
| 7123 | [V]Personal history of alcoholism | curr | H |
| 16587 | [V]Problems related to lifestyle alcohol use | curr | H |
| 7545 | [V] Alcohol use | curr |  |
| 8388 | [V]Alcohol rehabilitation | curr | H |
| 8030 | [V]Alcohol abuse counselling and surveillance | curr | H |

## Ethnicity (medcode, Read)

| medcode | readcode | readterm | | eth16 | eth5 |
| --- | --- | --- | --- | --- | --- |
| 12351 | 9i0..00 | british or mixed british - ethnic category 2001 census | | 1. British | 0. White |
| 110432 | 9t20.00 | white: scottish - scotland ethnic category 2011 census | | 1. British | 0. White |
| 12436 | 9i21.00 | scottish - ethnic category 2001 census | | 1. British | 0. White |
| 42294 | 9i24.00 | northern irish - ethnic category 2001 census | | 1. British | 0. White |
| 110694 | 9t21.00 | white: other british - scotland ethnic category 2011 census | | 1. British | 0. White |
| 110420 | 9t00.00 | white:eng/welsh/scot/ni/brit - england and wales 2011 census | | 1. British | 0. White |
| 12191 | 134B.00 | race: caucasian | | 1. British | 0. White |
| 22467 | 9S1..00 | white | | 1. British | 0. White |
| 12446 | 9S10.00 | white british | | 1. British | 0. White |
| 28887 | 9i23.00 | cornish - ethnic category 2001 census | | 1. British | 0. White |
| 26467 | 9S13.00 | white scottish | | 1. British | 0. White |
| 98111 | 9i00.00 | white british - ethnic category 2001 census | | 1. British | 0. White |
| 112899 | 9t10.00 | white - northern ireland ethnic category 2011 census | | 1. British | 0. White |
| 26310 | 9S14.00 | other white british ethnic group | | 1. British | 0. White |
| 12352 | 9i20.00 | english - ethnic category 2001 census | | 1. British | 0. White |
| 12681 | 9i22.00 | welsh - ethnic category 2001 census | | 1. British | 0. White |
| 40102 | 9i25.00 | ulster scots - ethnic category 2001 census | | 1. British | 0. White |
| 98213 | 9i10.00 | white irish - ethnic category 2001 census | | 2. Irish | 0. White |
| 110556 | 9t01.00 | white: irish - england and wales ethnic category 2011 census | | 2. Irish | 0. White |
| 12532 | 9i1..00 | irish - ethnic category 2001 census | | 2. Irish | 0. White |
| 110687 | 9t22.00 | white: irish - scotland ethnic category 2011 census | | 2. Irish | 0. White |
| 24837 | 9S11.00 | white irish | | 2. Irish | 0. White |
| 24270 | 9SA9.00 | irish (nmo) | | 2. Irish | 0. White |
| 12746 | 9i29.00 | turkish - ethnic category 2001 census | | 3. Other White | 0. White |
| 12433 | 9i2G.00 | baltic estonian/latvian/lithuanian - ethn categ 2001 census | | 3. Other White | 0. White |
| 32126 | 9SAB.11 | turkish (nmo) | | 3. Other White | 0. White |
| 110407 | 9t03.00 | white: other white backgrd- eng+wales ethnic cat 2011 census | | 3. Other White | 0. White |
| 12421 | 9i2..00 | other white background - ethnic category 2001 census | | 3. Other White | 0. White |
| 110695 | 9t25.00 | white: other white ethnic grp- scotland ethnic cat 2011 cens | | 3. Other White | 0. White |
| 28936 | 9i2P.00 | other republics former yugoslavia - ethnic categ 2001 census | | 3. Other White | 0. White |
| 28900 | 9i2S.00 | other mixed white - ethnic category 2001 census | | 3. Other White | 0. White |
| 12412 | 9i2B.00 | italian - ethnic category 2001 census | | 3. Other White | 0. White |
| 42290 | 9i2E.00 | gypsy/romany - ethnic category 2001 census | | 3. Other White | 0. White |
| 12633 | 9SAC.00 | other european (nmo) | | 3. Other White | 0. White |
| 55113 | 9i2D.00 | traveller - ethnic category 2001 census | | 3. Other White | 0. White |
| 12444 | 9S12.00 | other white ethnic group | | 3. Other White | 0. White |
| 111386 | 9t02.00 | white: gypsy/irish traveller - eng+wales eth cat 2011 census | | 3. Other White | 0. White |
| 113253 | 9t23.00 | white: gypsy/irish traveller - scotland ethnic cat 2011 cens | | 3. Other White | 0. White |
| 47601 | 9SI..00 | irish traveller | | 3. Other White | 0. White |
| 45955 | 9SAA.11 | greek (nmo) | | 3. Other White | 0. White |
| 26341 | 9i2J.00 | kosovan - ethnic category 2001 census | | 3. Other White | 0. White |
| 32066 | 9SAB.00 | turkish/turkish cypriot (nmo) | | 3. Other White | 0. White |
| 32778 | 9i26.00 | cypriot (part not stated) - ethnic category 2001 census | | 3. Other White | 0. White |
| 25422 | 9i2K.00 | albanian - ethnic category 2001 census | | 3. Other White | 0. White |
| 28973 | 9i2H.00 | commonwealth (russian) indep states - ethn categ 2001 census | | 3. Other White | 0. White |
| 46956 | 9i2L.00 | bosnian - ethnic category 2001 census | | 3. Other White | 0. White |
| 45947 | 9SAA.00 | greek/greek cypriot (nmo) | | 3. Other White | 0. White |
| 110465 | 9t24.00 | white: polish - scotland ethnic category 2011 census | | 3. Other White | 0. White |
| 55223 | 9i2C.00 | irish traveller - ethnic category 2001 census | | 3. Other White | 0. White |
| 32069 | 9SAB.12 | turkish cypriot (nmo) | | 3. Other White | 0. White |
| 12467 | 9i2F.00 | polish - ethnic category 2001 census | | 3. Other White | 0. White |
| 26391 | 9i2Q.00 | mixed irish and other white - ethnic category 2001 census | | 3. Other White | 0. White |
| 12355 | 9i27.00 | greek - ethnic category 2001 census | | 3. Other White | 0. White |
| 47949 | 9SAA.12 | greek cypriot (nmo) | | 3. Other White | 0. White |
| 47074 | 9i2N.00 | serbian - ethnic category 2001 census | | 3. Other White | 0. White |
| 12769 | 9i28.00 | greek cypriot - ethnic category 2001 census | | 3. Other White | 0. White |
| 12591 | 9i2T.00 | other white or white unspecified ethnic category 2001 census | | 3. Other White | 0. White |
| 32413 | 9i2A.00 | turkish cypriot - ethnic category 2001 census | | 3. Other White | 0. White |
| 28866 | 9i2M.00 | croatian - ethnic category 2001 census | | 3. Other White | 0. White |
| 12402 | 9i2R.00 | oth white european/european unsp/mixed european 2001 census | | 3. Other White | 0. White |
| 110661 | 9t12.00 | mixed: white and black caribbean - ni ethnic cat 2011 census | | 4. White and Black Caribbean | 4. Mixed |
| 32425 | 9SB5.00 | black caribbean and white | | 4. White and Black Caribbean | 4. Mixed |
| 110445 | 9t04.00 | mixed: white+black caribbean - eng+wales eth cat 2011 census | | 4. White and Black Caribbean | 4. Mixed |
| 12742 | 9i3..00 | white and black caribbean - ethnic category 2001 census | | 4. White and Black Caribbean | 4. Mixed |
| 12437 | 9i4..00 | white and black african - ethnic category 2001 census | | 5. White and Black African | 4. Mixed |
| 110421 | 9t05.00 | mixed: white+black african - eng+wales eth cat 2011 census | | 5. White and Black African | 4. Mixed |
| 45167 | 134L.00 | race: afro-caucasian | | 5. White and Black African | 4. Mixed |
| 32443 | 9SB6.00 | black african and white | | 5. White and Black African | 4. Mixed |
| 110651 | 9t13.00 | mixed: white and black african - ni ethnic cat 2011 census | | 5. White and Black African | 4. Mixed |
| 110471 | 9t14.00 | mixed: white and asian - ni ethnic category 2011 census | | 6. White and Asian | 4. Mixed |
| 12638 | 9i5..00 | white and asian - ethnic category 2001 census | | 6. White and Asian | 4. Mixed |
| 32401 | 9SB2.00 | other ethnic, asian/white orig | | 6. White and Asian | 4. Mixed |
| 110652 | 9t06.00 | mixed: white+asian - eng+wales ethnic category 2011 census | | 6. White and Asian | 4. Mixed |
| 12706 | 9i63.00 | chinese and white - ethnic category 2001 census | | 7. Other Mixed | 4. Mixed |
| 12696 | 9SB..00 | other ethnic, mixed origin | | 7. Other Mixed | 4. Mixed |
| 12873 | 9i6..00 | other mixed background - ethnic category 2001 census | | 7. Other Mixed | 4. Mixed |
| 35459 | 9SB3.00 | other ethnic, mixed white orig | | 7. Other Mixed | 4. Mixed |
| 110536 | 9t15.00 | mixed: other mixed/multiple ethnic backgrd - ni 2011 census | | 7. Other Mixed | 4. Mixed |
| 40110 | 9i62.00 | black and white - ethnic category 2001 census | | 7. Other Mixed | 4. Mixed |
| 32420 | 9SB4.00 | other ethnic, other mixed orig | | 7. Other Mixed | 4. Mixed |
| 25623 | 9S51.00 | other black - black/white orig | | 7. Other Mixed | 4. Mixed |
| 110696 | 9t26.00 | mixed/multiple ethnic grps: any- scot ethnic cat 2011 census | | 7. Other Mixed | 4. Mixed |
| 47005 | 9i64.00 | asian and chinese - ethnic category 2001 census | | 7. Other Mixed | 4. Mixed |
| 32165 | 9S52.00 | other black - black/asian orig | | 7. Other Mixed | 4. Mixed |
| 32408 | 9i65.00 | other mixed or mixed unspecified ethnic category 2001 census | | 7. Other Mixed | 4. Mixed |
| 47401 | 9SB1.00 | other ethnic, black/white orig | | 7. Other Mixed | 4. Mixed |
| 12795 | 9i60.00 | black and asian - ethnic category 2001 census | | 7. Other Mixed | 4. Mixed |
| 110654 | 9t07.00 | mixed: other mixed/multiple backgrd - eng+wales 2011 census | | 7. Other Mixed | 4. Mixed |
| 49940 | 9i61.00 | black and chinese - ethnic category 2001 census | | 7. Other Mixed | 4. Mixed |
| 12482 | 9S6..00 | indian | | 8. Indian | 1. South Asian |
| 45144 | 1347 | indian origin | | 8. Indian | 1. South Asian |
| 25920 | 9T1D.00 | indian | | 8. Indian | 1. South Asian |
| 110477 | 9t08.00 | asian/asian brit: indian - eng+wales ethnic cat 2011 census | | 8. Indian | 1. South Asian |
| 12414 | 9i7..00 | indian or british indian - ethnic category 2001 census | | 8. Indian | 1. South Asian |
| 111368 | 9t28.00 | asian: indian, indian scot/indian brit- scotland 2011 census | | 8. Indian | 1. South Asian |
| 110422 | 9t16.00 | asian or asian british: indian - ni ethnic cat 2011 census | | 8. Indian | 1. South Asian |
| 110460 | 9t27.00 | asian: pakistani/pakistani scot/pakistani brit- scot 2011 | | 9. Pakistani | 1. South Asian |
| 110464 | 9t09.00 | asian/asian british:pakistani- eng+wales eth cat 2011 census | | 9. Pakistani | 1. South Asian |
| 12460 | 9i8..00 | pakistani or british pakistani - ethnic category 2001 census | | 9. Pakistani | 1. South Asian |
| 110538 | 9t17.00 | asian/asian british: pakistani - ni ethnic cat 2011 census | | 9. Pakistani | 1. South Asian |
| 24690 | 9S7..00 | pakistani | | 9. Pakistani | 1. South Asian |
| 26062 | 134M.00 | race: pakistani | | 9. Pakistani | 1. South Asian |
| 26348 | 134I.00 | race: bangladeshi | | 10. Bangladeshi | 1. South Asian |
| 28888 | 9i9..00 | bangladeshi or british bangladeshi - ethn categ 2001 census | | 10. Bangladeshi | 1. South Asian |
| 110590 | 9t0A.00 | asian/asian brit: bangladeshi- eng+wales eth cat 2011 census | | 10. Bangladeshi | 1. South Asian |
| 24740 | 9S8..00 | bangladeshi | | 10. Bangladeshi | 1. South Asian |
| 112225 | 9t29.00 | bangladeshi, bangladeshi scot or bangladeshi brit- scot 2011 | | 10. Bangladeshi | 1. South Asian |
| 110720 | 9t18.00 | asian/asian british: bangladeshi - ni ethnic cat 2011 census | | 10. Bangladeshi | 1. South Asian |
| 64133 | 9iA2.00 | kashmiri - ethnic category 2001 census | | 11. Other Asian | 1. South Asian |
| 38097 | 9SA6.00 | e afric asian/indo-carib (nmo) | | 11. Other Asian | 1. South Asian |
| 39696 | 9SA7.00 | indian sub-continent (nmo) | | 11. Other Asian | 1. South Asian |
| 110855 | 9t2B.00 | asian: other asian group - scotland ethnic cat 2011 census | | 11. Other Asian | 1. South Asian |
| 32095 | 134K.00 | 11. Other Asian | 1. South Asian |  |  |
| 46056 | 9iA9.00 | mixed asian - ethnic category 2001 census | | 11. Other Asian | 1. South Asian |
| 12653 | 9iA8.00 | british asian - ethnic category 2001 census | | 11. Other Asian | 1. South Asian |
| 32132 | 2263.11 | o/e - asian origin | | 11. Other Asian | 1. South Asian |
| 99316 | 9SA6.12 | indo-caribbean (nmo) | | 11. Other Asian | 1. South Asian |
| 47077 | 9iA3.00 | east african asian - ethnic category 2001 census | | 11. Other Asian | 1. South Asian |
| 12887 | 9iA6.00 | sinhalese - ethnic category 2001 census | | 11. Other Asian | 1. South Asian |
| 28935 | 9iAA.00 | other asian or asian unspecified ethnic category 2001 census | | 11. Other Asian | 1. South Asian |
| 12760 | 9iA5.00 | tamil - ethnic category 2001 census | | 11. Other Asian | 1. South Asian |
| 25801 | 1343 | asian origin | | 11. Other Asian | 1. South Asian |
| 12668 | 9SH..00 | other asian ethnic group | | 11. Other Asian | 1. South Asian |
| 12513 | 9iA..00 | other asian background - ethnic category 2001 census | | 11. Other Asian | 1. South Asian |
| 12608 | 9iA4.00 | sri lankan - ethnic category 2001 census | | 11. Other Asian | 1. South Asian |
| 26379 | 9SA8.00 | other asian (nmo) | | 11. Other Asian | 1. South Asian |
| 45131 | 134A.00 | west indian origin | | 11. Other Asian | 1. South Asian |
| 26392 | 9iA1.00 | punjabi - ethnic category 2001 census | | 11. Other Asian | 1. South Asian |
| 110425 | 9t1A.00 | asian/asian british: other asian - ni ethnic cat 2011 census | | 11. Other Asian | 1. South Asian |
| 32396 | 9T1E.00 | other asian | | 11. Other Asian | 1. South Asian |
| 111743 | 9t0C.00 | asian/asian brit: other asian- eng+wales eth cat 2011 census | | 11. Other Asian | 1. South Asian |
| 46818 | 9SA6.11 | east african asian (nmo) | | 11. Other Asian | 1. South Asian |
| 32399 | 9iA7.00 | caribbean asian - ethnic category 2001 census | | 11. Other Asian | 1. South Asian |
| 110779 | 9t1C.00 | black/afri/carib/black brit: caribbean- ni eth cat 2011 cens | | 12. Caribbean | 2. Black |
| 12632 | 9S2..00 | black caribbean | | 12. Caribbean | 2. Black |
| 113671 | 9t2E.00 | carib/black: caribbean/carib scot/carib brit- scotland 2011 | | 12. Caribbean | 2. Black |
| 12432 | 9iB..00 | caribbean - ethnic category 2001 census | | 12. Caribbean | 2. Black |
| 110436 | 9t0E.00 | black/african/caribbn/black brit: caribbean - eng+wales 2011 | | 12. Caribbean | 2. Black |
| 110655 | 9t2D.00 | african: any other african - scotland ethnic cat 2011 census | | 13. African | 2. Black |
| 12350 | 9iC..00 | african - ethnic category 2001 census | | 13. African | 2. Black |
| 47969 | 9SA5.00 | other african countries (nmo) | | 13. African | 2. Black |
| 111059 | 9t2C.00 | african: african/african scot/african brit - scotland 2011 | | 13. African | 2. Black |
| 110630 | 9t1B.00 | black/afri/carib/black brit: african- ni eth cat 2011 census | | 13. African | 2. Black |
| 45125 | 1342 | african origin | | 13. African | 2. Black |
| 35412 | 9S44.00 | black - other african country | | 13. African | 2. Black |
| 110437 | 9t0D.00 | black/african/carib/black brit: african- eng+wales 2011 cens | | 13. African | 2. Black |
| 12778 | 9S3..00 | black african | | 13. African | 2. Black |
| 32100 | 9S42.13 | black guyana | | 14. Other Black | 2. Black |
| 47965 | 9S45.00 | black e afric asia/indo-caribb | | 14. Other Black | 2. Black |
| 57752 | 9S43.12 | black arab | | 14. Other Black | 2. Black |
| 25676 | 9S5..00 | black - other, mixed | | 14. Other Black | 2. Black |
| 57753 | 9S45.11 | black east african asian | | 14. Other Black | 2. Black |
| 24339 | 9S4..00 | black, other, non-mixed origin | | 14. Other Black | 2. Black |
| 12443 | 9iD0.00 | somali - ethnic category 2001 census | | 14. Other Black | 2. Black |
| 46812 | 9S43.11 | black north african | | 14. Other Black | 2. Black |
| 12452 | 9S41.00 | black british | | 14. Other Black | 2. Black |
| 111880 | 9t1D.00 | black/afri/carib/black brit: other - ni eth cat 2011 census | | 14. Other Black | 2. Black |
| 57763 | 9S45.12 | black indo-caribbean | | 14. Other Black | 2. Black |
| 40096 | 9iD3.00 | mixed black - ethnic category 2001 census | | 14. Other Black | 2. Black |
| 54593 | 9SA3.00 | caribbean i./w.i./guyana (nmo) | | 14. Other Black | 2. Black |
| 112649 | 9t2F.00 | carib/black: black/black scot/black brit- scotland 2011 cens | | 14. Other Black | 2. Black |
| 50286 | 9S43.13 | black iranian | | 14. Other Black | 2. Black |
| 47950 | 9S42.11 | black caribbean | | 14. Other Black | 2. Black |
| 35350 | 9S47.00 | black - other asian | | 14. Other Black | 2. Black |
| 93144 | 9SA3.13 | guyana (nmo) | | 14. Other Black | 2. Black |
| 32136 | 9SG..00 | other black ethnic group | | 14. Other Black | 2. Black |
| 26312 | 9S48.00 | black black - other | | 14. Other Black | 2. Black |
| 32389 | 9iD..00 | other black background - ethnic category 2001 census | | 14. Other Black | 2. Black |
| 46047 | 9iD4.00 | other black or black unspecified ethnic category 2001 census | | 14. Other Black | 2. Black |
| 41329 | 9S43.00 | black n african/arab/iranian | | 14. Other Black | 2. Black |
| 40097 | 9iD2.00 | black british - ethnic category 2001 census | | 14. Other Black | 2. Black |
| 112216 | 9t2G.00 | carib/black: any other black/caribbean grp - scotland 2011 | | 14. Other Black | 2. Black |
| 25894 | 134H.00 | race: afro-caribbean | | 14. Other Black | 2. Black |
| 57435 | 9S42.00 | black caribbean/w.i./guyana | | 14. Other Black | 2. Black |
| 110540 | 9t0F.00 | black/afr/carib/black brit: other black- eng+wales 2011 cens | | 14. Other Black | 2. Black |
| 57075 | 9SA3.12 | west indian (nmo) | | 14. Other Black | 2. Black |
| 47997 | 9S42.12 | black west indian | | 14. Other Black | 2. Black |
| 57094 | 9SA3.11 | caribbean island (nmo) | | 14. Other Black | 2. Black |
| 48005 | 9S46.00 | black indian sub-continent | | 14. Other Black | 2. Black |
| 32886 | 9iD1.00 | nigerian - ethnic category 2001 census | | 14. Other Black | 2. Black |
| 24272 | 9S9..00 | chinese | | 15. Chinese | 3. Other |
| 111064 | 9t2A.00 | asian: chinese - scotland ethnic category 2011 census | | 15. Chinese | 3. Other |
| 110922 | 9t0B.00 | asian/asian brit: chinese - eng+wales ethnic cat 2011 census | | 15. Chinese | 3. Other |
| 112363 | 9t19.00 | asian/asian british: chinese - ni ethnic cat 2011 census | | 15. Chinese | 3. Other |
| 12468 | 9iE..00 | chinese - ethnic category 2001 census | | 15. Chinese | 3. Other |
| 47285 | 9SA4.11 | north african arab (nmo) | | 16. Other ethnic group | 3. Other |
| 26246 | 9iFG.00 | latin american - ethnic category 2001 census | | 16. Other ethnic group | 3. Other |
| 24962 | 9SA4.00 | n african arab/iranian (nmo) | | 16. Other ethnic group | 3. Other |
| 12473 | 9iF1.00 | japanese - ethnic category 2001 census | | 16. Other ethnic group | 3. Other |
| 45964 | 9iFE.00 | kurdish - ethnic category 2001 census | | 16. Other ethnic group | 3. Other |
| 47091 | 9iF7.00 | muslim - ethnic category 2001 census | | 16. Other ethnic group | 3. Other |
| 110646 | 9t1F.00 | other ethnic group: any other grp- ni ethnic cat 2011 census | | 16. Other ethnic group | 3. Other |
| 110780 | 9t1E.00 | other ethnic group: arab - ni ethnic category 2011 census | | 16. Other ethnic group | 3. Other |
| 32382 | 9iFJ.00 | mauritian/seychellois/maldivian/st helena eth cat 2001census | | 16. Other ethnic group | 3. Other |
| 110742 | 9t0H.00 | other ethnic: any other grp - eng+wales eth cat 2011 census | | 16. Other ethnic group | 3. Other |
| 96789 | 9T1Y.00 | other new zealand ethnic group | | 16. Other ethnic group | 3. Other |
| 111806 | 9t2J.00 | other ethnic grp: any other ethnic grp- scotland 2011 census | | 16. Other ethnic group | 3. Other |
| 25411 | 9SC..00 | vietnamese | | 16. Other ethnic group | 3. Other |
| 41214 | 9SAD.00 | other ethnic nec (nmo) | | 16. Other ethnic group | 3. Other |
| 32110 | 9SA1.00 | brit. ethnic minor. spec.(nmo) | | 16. Other ethnic group | 3. Other |
| 46059 | 9iF9.00 | arab - ethnic category 2001 census | | 16. Other ethnic group | 3. Other |
| 12757 | 9SJ..00 | other ethnic group | | 16. Other ethnic group | 3. Other |
| 46649 | 9T1B.00 | south east asian | | 16. Other ethnic group | 3. Other |
| 46063 | 9iF6.00 | jewish - ethnic category 2001 census | | 16. Other ethnic group | 3. Other |
| 12719 | 9iF0.00 | vietnamese - ethnic category 2001 census | | 16. Other ethnic group | 3. Other |
| 110555 | 9t0G.00 | other ethnic group: arab - eng+wales ethnic cat 2011 census | | 16. Other ethnic group | 3. Other |
| 56127 | 9iF5.00 | hindu - ethnic category 2001 census | | 16. Other ethnic group | 3. Other |
| 25937 | 9iFD.00 | iranian - ethnic category 2001 census | | 16. Other ethnic group | 3. Other |
| 30280 | 9SA..00 | other ethnic non-mixed (nmo) | | 16. Other ethnic group | 3. Other |
| 12730 | 9iF3.00 | malaysian - ethnic category 2001 census | | 16. Other ethnic group | 3. Other |
| 46752 | 9T1A.00 | other pacific ethnic group | | 16. Other ethnic group | 3. Other |
| 49658 | 9iF8.00 | sikh - ethnic category 2001 census | | 16. Other ethnic group | 3. Other |
| 12434 | 9iF..00 | other - ethnic category 2001 census | | 16. Other ethnic group | 3. Other |
| 63872 | 9iF4.00 | buddhist - ethnic category 2001 census | | 16. Other ethnic group | 3. Other |
| 12420 | 9iF2.00 | filipino - ethnic category 2001 census | | 16. Other ethnic group | 3. Other |
| 28909 | 9iFB.00 | mid east (excl israeli, iranian & arab) - eth cat 2001 cens | | 16. Other ethnic group | 3. Other |
| 25451 | 9iFF.00 | moroccan - ethnic category 2001 census | | 16. Other ethnic group | 3. Other |
| 26455 | 9iFK.00 | any other group - ethnic category 2001 census | | 16. Other ethnic group | 3. Other |
| 71425 | 9T1Z.00 | new zealand ethnic group nos | | 16. Other ethnic group | 3. Other |
| 12756 | 9iFH.00 | south and central american - ethnic category 2001 census | | 16. Other ethnic group | 3. Other |
| 112245 | 9t2H.00 | other ethnic grp: arab/arab scot/arab british- scotland 2011 | | 16. Other ethnic group | 3. Other |
| 57764 | 9SA2.00 | brit. ethnic minor. unsp (nmo) | | 16. Other ethnic group | 3. Other |
| 46964 | 9iFC.00 | israeli - ethnic category 2001 census | | 16. Other ethnic group | 3. Other |
| 25082 | 9SA4.12 | iranian (nmo) | | 16. Other ethnic group | 3. Other |
| 47028 | 9iFA.00 | north african - ethnic category 2001 census | | 16. Other ethnic group | 3. Other |
| 112302 | 9t1..00 | ethnic category - 2011 census northern ireland | | 17. Not Stated | 5. Not Stated |
| 12429 | 9SD..00 | ethnic group not given - patient refused | | 17. Not Stated | 5. Not Stated |
| 45199 | 9SZ..00 | ethnic groups (census) nos | | 17. Not Stated | 5. Not Stated |
| 10196 | 9S...00 | ethnic groups (census) | | 17. Not Stated | 5. Not Stated |
| 12435 | 9i...00 | ethnic category - 2001 census | | 17. Not Stated | 5. Not Stated |
| 60284 | 226Z.00 | o/e - ethnic group nos | | 17. Not Stated | 5. Not Stated |
| 110417 | 9t0..00 | ethnic category - 2011 census england and wales | | 17. Not Stated | 5. Not Stated |
| 45008 | 9T1..00 | new zealand ethnic groups | | 17. Not Stated | 5. Not Stated |
| 12459 | 9iG..00 | ethnic category not stated - 2001 census | | 17. Not Stated | 5. Not Stated |
| 110472 | 9t...00 | ethnic category - 2011 census | | 17. Not Stated | 5. Not Stated |
| 12332 | 226..11 | o/e - ethnic origin | | 17. Not Stated | 5. Not Stated |
| 24340 | 9SE..00 | ethnic group not recorded | | 17. Not Stated | 5. Not Stated |
| 23955 | 9T...00 | ethnicity and other related nationality data | | 17. Not Stated | 5. Not Stated |
| 25969 | 226..00 | o/e - ethnic group | | 17. Not Stated | 5. Not Stated |
| 110962 | 9t2..00 | ethnic category - 2011 census scotland | | 17. Not Stated | 5. Not Stated |

## Diabetes mellitus (medcode, Read)

| medcode | readcode | readterm |
| --- | --- | --- |
| 6813 | 1434 | h/o: diabetes mellitus |
| 7045 | 14F4.00 | h/o: admission in last year for diabetes foot problem |
| 17236 | 14P3.00 | h/o: insulin therapy |
| 28622 | 2126300 | diabetes resolved |
| 18766 | 212H.00 | diabetes resolved |
| 54846 | 9OL9.00 | diabetes monitoring deleted |
| 85660 | 66An.00 | diabetes type 1 review |
| 102704 | 66At000 | type i diabetic dietary review |
| 104453 | 66At011 | type 1 diabetic dietary review |
| 24490 | C100000 | diabetes mellitus, juvenile type, no mention of complication |
| 1038 | C100011 | insulin dependent diabetes mellitus |
| 53200 | C101000 | diabetes mellitus, juvenile type, with ketoacidosis |
| 40023 | C102000 | diabetes mellitus, juvenile type, with hyperosmolar coma |
| 42567 | C103000 | diabetes mellitus, juvenile type, with ketoacidotic coma |
| 93922 | C104000 | diabetes mellitus, juvenile type, with renal manifestation |
| 69748 | C105000 | diabetes mellitus, juvenile type, + ophthalmic manifestation |
| 67853 | C106000 | diabetes mellitus, juvenile, + neurological manifestation |
| 70448 | C107000 | diabetes mellitus, juvenile +peripheral circulatory disorder |
| 69124 | C107300 | iddm with peripheral circulatory disorder |
| 1647 | C108.00 | insulin dependent diabetes mellitus |
| 18505 | C108.11 | iddm-insulin dependent diabetes mellitus |
| 17858 | C108.12 | type 1 diabetes mellitus |
| 24423 | C108.13 | type i diabetes mellitus |
| 46963 | C108000 | insulin-dependent diabetes mellitus with renal complications |
| 61344 | C108011 | type i diabetes mellitus with renal complications |
| 21983 | C108012 | type 1 diabetes mellitus with renal complications |
| 49276 | C108100 | insulin-dependent diabetes mellitus with ophthalmic comps |
| 102740 | C108112 | type 1 diabetes mellitus with ophthalmic complications |
| 52283 | C108200 | insulin-dependent diabetes mellitus with neurological comps |
| 49146 | C108211 | type i diabetes mellitus with neurological complications |
| 61829 | C108212 | type 1 diabetes mellitus with neurological complications |
| 52104 | C108300 | insulin dependent diabetes mellitus with multiple complicatn |
| 108007 | C108311 | type i diabetes mellitus with multiple complications |
| 26855 | C108400 | unstable insulin dependent diabetes mellitus |
| 60107 | C108411 | unstable type i diabetes mellitus |
| 97474 | C108412 | unstable type 1 diabetes mellitus |
| 44443 | C108500 | insulin dependent diabetes mellitus with ulcer |
| 51957 | C108511 | type i diabetes mellitus with ulcer |
| 68390 | C108512 | type 1 diabetes mellitus with ulcer |
| 60499 | C108600 | insulin dependent diabetes mellitus with gangrene |
| 6509 | C108700 | insulin dependent diabetes mellitus with retinopathy |
| 38161 | C108711 | type i diabetes mellitus with retinopathy |
| 41049 | C108712 | type 1 diabetes mellitus with retinopathy |
| 6791 | C108800 | insulin dependent diabetes mellitus - poor control |
| 46850 | C108811 | type i diabetes mellitus - poor control |
| 45914 | C108812 | type 1 diabetes mellitus - poor control |
| 31310 | C108900 | insulin dependent diabetes maturity onset |
| 63017 | C108911 | type i diabetes mellitus maturity onset |
| 97446 | C108912 | type 1 diabetes mellitus maturity onset |
| 56448 | C108A00 | insulin-dependent diabetes without complication |
| 95992 | C108A11 | type i diabetes mellitus without complication |
| 111106 | C108A12 | type 1 diabetes mellitus without complication |
| 24694 | C108B00 | insulin dependent diabetes mellitus with mononeuropathy |
| 99231 | C108B11 | type i diabetes mellitus with mononeuropathy |
| 41716 | C108C00 | insulin dependent diabetes mellitus with polyneuropathy |
| 113975 | C108C11 | type i diabetes mellitus with polyneuropathy |
| 57621 | C108D00 | insulin dependent diabetes mellitus with nephropathy |
| 66872 | C108D11 | type i diabetes mellitus with nephropathy |
| 113197 | C108D12 | type 1 diabetes mellitus with nephropathy |
| 44440 | C108E00 | insulin dependent diabetes mellitus with hypoglycaemic coma |
| 42729 | C108E11 | type i diabetes mellitus with hypoglycaemic coma |
| 70766 | C108E12 | type 1 diabetes mellitus with hypoglycaemic coma |
| 44260 | C108F00 | insulin dependent diabetes mellitus with diabetic cataract |
| 17545 | C108F11 | type i diabetes mellitus with diabetic cataract |
| 110400 | C108F12 | type 1 diabetes mellitus with diabetic cataract |
| 64446 | C108G00 | insulin dependent diab mell with peripheral angiopathy |
| 65616 | C108H00 | insulin dependent diabetes mellitus with arthropathy |
| 62352 | C108H11 | type i diabetes mellitus with arthropathy |
| 39809 | C108J00 | insulin dependent diab mell with neuropathic arthropathy |
| 60208 | C108J11 | type i diabetes mellitus with neuropathic arthropathy |
| 18230 | C108J12 | type 1 diabetes mellitus with neuropathic arthropathy |
| 98392 | C10C.12 | maturity onset diabetes in youth type 1 |
| 1549 | C10E.00 | type 1 diabetes mellitus |
| 12455 | C10E.11 | type i diabetes mellitus |
| 51261 | C10E.12 | insulin dependent diabetes mellitus |
| 47582 | C10E000 | type 1 diabetes mellitus with renal complications |
| 109837 | C10E011 | type i diabetes mellitus with renal complications |
| 102946 | C10E012 | insulin-dependent diabetes mellitus with renal complications |
| 47649 | C10E100 | type 1 diabetes mellitus with ophthalmic complications |
| 99311 | C10E111 | type i diabetes mellitus with ophthalmic complications |
| 98071 | C10E112 | insulin-dependent diabetes mellitus with ophthalmic comps |
| 42831 | C10E200 | type 1 diabetes mellitus with neurological complications |
| 101735 | C10E212 | insulin-dependent diabetes mellitus with neurological comps |
| 47650 | C10E300 | type 1 diabetes mellitus with multiple complications |
| 91942 | C10E311 | type i diabetes mellitus with multiple complications |
| 45276 | C10E312 | insulin dependent diabetes mellitus with multiple complicat |
| 43921 | C10E400 | unstable type 1 diabetes mellitus |
| 49949 | C10E411 | unstable type i diabetes mellitus |
| 54600 | C10E412 | unstable insulin dependent diabetes mellitus |
| 18683 | C10E500 | type 1 diabetes mellitus with ulcer |
| 93878 | C10E511 | type i diabetes mellitus with ulcer |
| 98704 | C10E512 | insulin dependent diabetes mellitus with ulcer |
| 69993 | C10E600 | type 1 diabetes mellitus with gangrene |
| 102112 | C10E611 | type i diabetes mellitus with gangrene |
| 109051 | C10E612 | insulin dependent diabetes mellitus with gangrene |
| 18387 | C10E700 | type 1 diabetes mellitus with retinopathy |
| 95343 | C10E711 | type i diabetes mellitus with retinopathy |
| 93875 | C10E712 | insulin dependent diabetes mellitus with retinopathy |
| 35288 | C10E800 | type 1 diabetes mellitus - poor control |
| 105337 | C10E811 | type i diabetes mellitus - poor control |
| 72702 | C10E812 | insulin dependent diabetes mellitus - poor control |
| 40682 | C10E900 | type 1 diabetes mellitus maturity onset |
| 96235 | C10E911 | type i diabetes mellitus maturity onset |
| 97849 | C10E912 | insulin dependent diabetes maturity onset |
| 69676 | C10EA00 | type 1 diabetes mellitus without complication |
| 62613 | C10EA11 | type i diabetes mellitus without complication |
| 99719 | C10EA12 | insulin-dependent diabetes without complication |
| 68105 | C10EB00 | type 1 diabetes mellitus with mononeuropathy |
| 46301 | C10EC00 | type 1 diabetes mellitus with polyneuropathy |
| 91943 | C10EC11 | type i diabetes mellitus with polyneuropathy |
| 101311 | C10EC12 | insulin dependent diabetes mellitus with polyneuropathy |
| 10418 | C10ED00 | type 1 diabetes mellitus with nephropathy |
| 113863 | C10ED11 | type i diabetes mellitus with nephropathy |
| 102163 | C10ED12 | insulin dependent diabetes mellitus with nephropathy |
| 39070 | C10EE00 | type 1 diabetes mellitus with hypoglycaemic coma |
| 99716 | C10EE12 | insulin dependent diabetes mellitus with hypoglycaemic coma |
| 49554 | C10EF00 | type 1 diabetes mellitus with diabetic cataract |
| 100770 | C10EF12 | insulin dependent diabetes mellitus with diabetic cataract |
| 93468 | C10EG00 | type 1 diabetes mellitus with peripheral angiopathy |
| 18642 | C10EH00 | type 1 diabetes mellitus with arthropathy |
| 54008 | C10EJ00 | type 1 diabetes mellitus with neuropathic arthropathy |
| 30323 | C10EK00 | type 1 diabetes mellitus with persistent proteinuria |
| 30294 | C10EL00 | type 1 diabetes mellitus with persistent microalbuminuria |
| 102620 | C10EL11 | type i diabetes mellitus with persistent microalbuminuria |
| 10692 | C10EM00 | type 1 diabetes mellitus with ketoacidosis |
| 62209 | C10EM11 | type i diabetes mellitus with ketoacidosis |
| 40837 | C10EN00 | type 1 diabetes mellitus with ketoacidotic coma |
| 66145 | C10EN11 | type i diabetes mellitus with ketoacidotic coma |
| 22871 | C10EP00 | type 1 diabetes mellitus with exudative maculopathy |
| 97894 | C10EP11 | type i diabetes mellitus with exudative maculopathy |
| 55239 | C10EQ00 | type 1 diabetes mellitus with gastroparesis |
| 108724 | C10EQ11 | type i diabetes mellitus with gastroparesis |
| 108360 | C10P000 | type i diabetes mellitus in remission |
| 109628 | C10P011 | type 1 diabetes mellitus in remission |
| 110997 | C10y000 | diabetes mellitus, juvenile, + other specified manifestation |
| 68792 | C10z000 | diabetes mellitus, juvenile type, + unspecified complication |
| 50960 | L180500 | pre-existing diabetes mellitus, insulin-dependent |
| 38076 | M21yC00 | insulin lipohypertrophy |
| 43493 | M21yC11 | insulin site lipohypertrophy |
| 69043 | ZC2C900 | dietary advice for type i diabetes |
| 109878 | ZC2C911 | diet advice for insulin-dependent diabetes |
| 32359 | ZRbH.00 | perceived control of insulin-dependent diabetes |
| 83532 | 66Ao.00 | diabetes type 2 review |
| 101801 | 66At100 | type ii diabetic dietary review |
| 102611 | 66At111 | type 2 diabetic dietary review |
| 14803 | C100100 | diabetes mellitus, adult onset, no mention of complication |
| 14889 | C100111 | maturity onset diabetes |
| 506 | C100112 | non-insulin dependent diabetes mellitus |
| 54856 | C101100 | diabetes mellitus, adult onset, with ketoacidosis |
| 43139 | C102100 | diabetes mellitus, adult onset, with hyperosmolar coma |
| 68843 | C103100 | diabetes mellitus, adult onset, with ketoacidotic coma |
| 35105 | C104100 | diabetes mellitus, adult onset, with renal manifestation |
| 41389 | C105100 | diabetes mellitus, adult onset, + ophthalmic manifestation |
| 39317 | C106100 | diabetes mellitus, adult onset, + neurological manifestation |
| 63357 | C107100 | diabetes mellitus, adult, + peripheral circulatory disorder |
| 33807 | C107200 | diabetes mellitus, adult with gangrene |
| 56803 | C107400 | niddm with peripheral circulatory disorder |
| 4513 | C109.00 | non-insulin dependent diabetes mellitus |
| 5884 | C109.11 | niddm - non-insulin dependent diabetes mellitus |
| 17859 | C109.12 | type 2 diabetes mellitus |
| 18219 | C109.13 | type ii diabetes mellitus |
| 52303 | C109000 | non-insulin-dependent diabetes mellitus with renal comps |
| 50225 | C109011 | type ii diabetes mellitus with renal complications |
| 18209 | C109012 | type 2 diabetes mellitus with renal complications |
| 50429 | C109100 | non-insulin-dependent diabetes mellitus with ophthalm comps |
| 59725 | C109111 | type ii diabetes mellitus with ophthalmic complications |
| 70316 | C109112 | type 2 diabetes mellitus with ophthalmic complications |
| 55842 | C109200 | non-insulin-dependent diabetes mellitus with neuro comps |
| 67905 | C109211 | type ii diabetes mellitus with neurological complications |
| 45919 | C109212 | type 2 diabetes mellitus with neurological complications |
| 62146 | C109300 | non-insulin-dependent diabetes mellitus with multiple comps |
| 113495 | C109311 | type ii diabetes mellitus with multiple complications |
| 108005 | C109312 | type 2 diabetes mellitus with multiple complications |
| 34912 | C109400 | non-insulin dependent diabetes mellitus with ulcer |
| 55075 | C109411 | type ii diabetes mellitus with ulcer |
| 65704 | C109412 | type 2 diabetes mellitus with ulcer |
| 40401 | C109500 | non-insulin dependent diabetes mellitus with gangrene |
| 62107 | C109511 | type ii diabetes mellitus with gangrene |
| 46150 | C109512 | type 2 diabetes mellitus with gangrene |
| 17262 | C109600 | non-insulin-dependent diabetes mellitus with retinopathy |
| 58604 | C109611 | type ii diabetes mellitus with retinopathy |
| 42762 | C109612 | type 2 diabetes mellitus with retinopathy |
| 8403 | C109700 | non-insulin dependent diabetes mellitus - poor control |
| 24458 | C109711 | type ii diabetes mellitus - poor control |
| 45913 | C109712 | type 2 diabetes mellitus - poor control |
| 29979 | C109900 | non-insulin-dependent diabetes mellitus without complication |
| 109103 | C109911 | type ii diabetes mellitus without complication |
| 105784 | C109912 | type 2 diabetes mellitus without complication |
| 72320 | C109A00 | non-insulin dependent diabetes mellitus with mononeuropathy |
| 50813 | C109A11 | type ii diabetes mellitus with mononeuropathy |
| 45467 | C109B00 | non-insulin dependent diabetes mellitus with polyneuropathy |
| 47409 | C109B11 | type ii diabetes mellitus with polyneuropathy |
| 109865 | C109B12 | type 2 diabetes mellitus with polyneuropathy |
| 59365 | C109C00 | non-insulin dependent diabetes mellitus with nephropathy |
| 64571 | C109C11 | type ii diabetes mellitus with nephropathy |
| 24836 | C109C12 | type 2 diabetes mellitus with nephropathy |
| 43785 | C109D00 | non-insulin dependent diabetes mellitus with hypoglyca coma |
| 56268 | C109D11 | type ii diabetes mellitus with hypoglycaemic coma |
| 61071 | C109D12 | type 2 diabetes mellitus with hypoglycaemic coma |
| 69278 | C109E00 | non-insulin depend diabetes mellitus with diabetic cataract |
| 48192 | C109E11 | type ii diabetes mellitus with diabetic cataract |
| 44779 | C109E12 | type 2 diabetes mellitus with diabetic cataract |
| 54212 | C109F00 | non-insulin-dependent d m with peripheral angiopath |
| 54899 | C109F11 | type ii diabetes mellitus with peripheral angiopathy |
| 60699 | C109F12 | type 2 diabetes mellitus with peripheral angiopathy |
| 24693 | C109G00 | non-insulin dependent diabetes mellitus with arthropathy |
| 18143 | C109G11 | type ii diabetes mellitus with arthropathy |
| 49869 | C109G12 | type 2 diabetes mellitus with arthropathy |
| 40962 | C109H00 | non-insulin dependent d m with neuropathic arthropathy |
| 47816 | C109H11 | type ii diabetes mellitus with neuropathic arthropathy |
| 66965 | C109H12 | type 2 diabetes mellitus with neuropathic arthropathy |
| 18278 | C109J00 | insulin treated type 2 diabetes mellitus |
| 37648 | C109J11 | insulin treated non-insulin dependent diabetes mellitus |
| 18264 | C109J12 | insulin treated type ii diabetes mellitus |
| 36633 | C109K00 | hyperosmolar non-ketotic state in type 2 diabetes mellitus |
| 46624 | C10C.11 | maturity onset diabetes in youth |
| 36695 | C10D.00 | diabetes mellitus autosomal dominant type 2 |
| 59991 | C10D.11 | maturity onset diabetes in youth type 2 |
| 95636 | C10ER00 | latent autoimmune diabetes mellitus in adult |
| 758 | C10F.00 | type 2 diabetes mellitus |
| 22884 | C10F.11 | type ii diabetes mellitus |
| 18777 | C10F000 | type 2 diabetes mellitus with renal complications |
| 57278 | C10F011 | type ii diabetes mellitus with renal complications |
| 47321 | C10F100 | type 2 diabetes mellitus with ophthalmic complications |
| 100964 | C10F111 | type ii diabetes mellitus with ophthalmic complications |
| 34268 | C10F200 | type 2 diabetes mellitus with neurological complications |
| 98616 | C10F211 | type ii diabetes mellitus with neurological complications |
| 65267 | C10F300 | type 2 diabetes mellitus with multiple complications |
| 43227 | C10F311 | type ii diabetes mellitus with multiple complications |
| 49074 | C10F400 | type 2 diabetes mellitus with ulcer |
| 91646 | C10F411 | type ii diabetes mellitus with ulcer |
| 12736 | C10F500 | type 2 diabetes mellitus with gangrene |
| 104323 | C10F511 | type ii diabetes mellitus with gangrene |
| 18496 | C10F600 | type 2 diabetes mellitus with retinopathy |
| 49655 | C10F611 | type ii diabetes mellitus with retinopathy |
| 25627 | C10F700 | type 2 diabetes mellitus - poor control |
| 47315 | C10F711 | type ii diabetes mellitus - poor control |
| 47954 | C10F900 | type 2 diabetes mellitus without complication |
| 53392 | C10F911 | type ii diabetes mellitus without complication |
| 62674 | C10FA00 | type 2 diabetes mellitus with mononeuropathy |
| 95351 | C10FA11 | type ii diabetes mellitus with mononeuropathy |
| 18425 | C10FB00 | type 2 diabetes mellitus with polyneuropathy |
| 50527 | C10FB11 | type ii diabetes mellitus with polyneuropathy |
| 12640 | C10FC00 | type 2 diabetes mellitus with nephropathy |
| 102201 | C10FC11 | type ii diabetes mellitus with nephropathy |
| 46917 | C10FD00 | type 2 diabetes mellitus with hypoglycaemic coma |
| 98723 | C10FD11 | type ii diabetes mellitus with hypoglycaemic coma |
| 44982 | C10FE00 | type 2 diabetes mellitus with diabetic cataract |
| 93727 | C10FE11 | type ii diabetes mellitus with diabetic cataract |
| 37806 | C10FF00 | type 2 diabetes mellitus with peripheral angiopathy |
| 104639 | C10FF11 | type ii diabetes mellitus with peripheral angiopathy |
| 59253 | C10FG00 | type 2 diabetes mellitus with arthropathy |
| 103902 | C10FG11 | type ii diabetes mellitus with arthropathy |
| 35385 | C10FH00 | type 2 diabetes mellitus with neuropathic arthropathy |
| 109197 | C10FH11 | type ii diabetes mellitus with neuropathic arthropathy |
| 1407 | C10FJ00 | insulin treated type 2 diabetes mellitus |
| 64668 | C10FJ11 | insulin treated type ii diabetes mellitus |
| 34450 | C10FK00 | hyperosmolar non-ketotic state in type 2 diabetes mellitus |
| 107701 | C10FK11 | hyperosmolar non-ketotic state in type ii diabetes mellitus |
| 26054 | C10FL00 | type 2 diabetes mellitus with persistent proteinuria |
| 60796 | C10FL11 | type ii diabetes mellitus with persistent proteinuria |
| 18390 | C10FM00 | type 2 diabetes mellitus with persistent microalbuminuria |
| 85991 | C10FM11 | type ii diabetes mellitus with persistent microalbuminuria |
| 32627 | C10FN00 | type 2 diabetes mellitus with ketoacidosis |
| 106528 | C10FN11 | type ii diabetes mellitus with ketoacidosis |
| 51756 | C10FP00 | type 2 diabetes mellitus with ketoacidotic coma |
| 106061 | C10FP11 | type ii diabetes mellitus with ketoacidotic coma |
| 25591 | C10FQ00 | type 2 diabetes mellitus with exudative maculopathy |
| 111798 | C10FQ11 | type ii diabetes mellitus with exudative maculopathy |
| 63690 | C10FR00 | type 2 diabetes mellitus with gastroparesis |
| 113609 | C10FR11 | type ii diabetes mellitus with gastroparesis |
| 107824 | C10P100 | type ii diabetes mellitus in remission |
| 110611 | C10P111 | type 2 diabetes mellitus in remission |
| 63371 | C10y100 | diabetes mellitus, adult, + other specified manifestation |
| 63762 | C10z100 | diabetes mellitus, adult onset, + unspecified complication |
| 50609 | L180600 | pre-existing diabetes mellitus, non-insulin-dependent |
| 25041 | ZC2CA00 | dietary advice for type ii diabetes |
| 21689 | 13AB.00 | diabetic lipid lowering diet |
| 13078 | 13AC.00 | diabetic weight reducing diet |
| 13074 | 13B1.00 | diabetic diet |
| 110393 | 13B1000 | diabetic carbohydrate counting diet |
| 105481 | 14O8000 | high risk of diabetes mellitus |
| 103935 | 1IA..00 | no evidence of diabetic nephropathy |
| 109760 | 1M8..00 | diabetic peripheral neuropathic pain |
| 22967 | 2BBF.00 | retinal abnormality - diabetes related |
| 9835 | 2BBL.00 | o/e - diabetic maculopathy present both eyes |
| 47144 | 2BBM.00 | o/e - diabetic maculopathy absent both eyes |
| 11433 | 2BBP.00 | o/e - right eye background diabetic retinopathy |
| 11129 | 2BBQ.00 | o/e - left eye background diabetic retinopathy |
| 13099 | 2BBR.00 | o/e - right eye preproliferative diabetic retinopathy |
| 13103 | 2BBS.00 | o/e - left eye preproliferative diabetic retinopathy |
| 13097 | 2BBT.00 | o/e - right eye proliferative diabetic retinopathy |
| 13101 | 2BBV.00 | o/e - left eye proliferative diabetic retinopathy |
| 13102 | 2BBW.00 | o/e - right eye diabetic maculopathy |
| 13108 | 2BBX.00 | o/e - left eye diabetic maculopathy |
| 47328 | 2BBk.00 | o/e - right eye stable treated prolif diabetic retinopathy |
| 52041 | 2BBl.00 | o/e - left eye stable treated prolif diabetic retinopathy |
| 52630 | 2BBo.00 | o/e - sight threatening diabetic retinopathy |
| 101881 | 2BBr.00 | impaired vision due to diabetic retinopathy |
| 27921 | 2G51000 | foot abnormality - diabetes related |
| 17095 | 2G5A.00 | o/e - right diabetic foot at risk |
| 26664 | 2G5B.00 | o/e - left diabetic foot at risk |
| 18056 | 2G5C.00 | foot abnormality - diabetes related |
| 26666 | 2G5E.00 | o/e - right diabetic foot at low risk |
| 31157 | 2G5F.00 | o/e - right diabetic foot at moderate risk |
| 31171 | 2G5G.00 | o/e - right diabetic foot at high risk |
| 35316 | 2G5H.00 | o/e - right diabetic foot - ulcerated |
| 26667 | 2G5I.00 | o/e - left diabetic foot at low risk |
| 31156 | 2G5J.00 | o/e - left diabetic foot at moderate risk |
| 31172 | 2G5K.00 | o/e - left diabetic foot at high risk |
| 35116 | 2G5L.00 | o/e - left diabetic foot - ulcerated |
| 62384 | 2G5V.00 | o/e - right chronic diabetic foot ulcer |
| 49640 | 2G5W.00 | o/e - left chronic diabetic foot ulcer |
| 105740 | 2G5d.00 | o/e - left diabetic foot at increased risk |
| 105741 | 2G5e.00 | o/e - right diabetic foot at increased risk |
| 99822 | 38DK.00 | finnish diabetes risk score |
| 106622 | 38Gj.00 | qdiabetes risk calculator |
| 107554 | 38Gv.00 | diabetes uk diabetes risk score |
| 113594 | 38QW.00 | diabetes distress scale 2 item |
| 108993 | 661M400 | diabetes self-management plan agreed |
| 107423 | 661N400 | diabetes self-management plan review |
| 7563 | 66A3.00 | diabetic on diet only |
| 1684 | 66A4.00 | diabetic on oral treatment |
| 8842 | 66A5.00 | diabetic on insulin |
| 13069 | 66A8.00 | has seen dietician - diabetes |
| 38078 | 66A9.00 | understands diet - diabetes |
| 20696 | 66AA.11 | injection sites - diabetic |
| 13196 | 66AD.00 | fundoscopy - diabetic check |
| 53238 | 66AG.00 | diabetic drug side effects |
| 16490 | 66AH.00 | diabetic treatment changed |
| 107508 | 66AH200 | conversion to insulin by diabetes specialist nurse |
| 13071 | 66AI.00 | diabetic - good control |
| 2378 | 66AJ.00 | diabetic - poor control |
| 9013 | 66AJ.11 | unstable diabetes |
| 2478 | 66AJ100 | brittle diabetes |
| 22023 | 66AJz00 | diabetic - poor control nos |
| 43951 | 66AK.00 | diabetic - cooperative patient |
| 17869 | 66AL.00 | diabetic-uncooperative patient |
| 29041 | 66AN.00 | date diabetic treatment start |
| 55123 | 66AO.00 | date diabetic treatment stopp. |
| 12506 | 66AP.00 | diabetes: practice programme |
| 12675 | 66AQ.00 | diabetes: shared care programme |
| 100533 | 66AQ000 | unsuitable for diabetes year of care programme |
| 101190 | 66AQ100 | declined consent for diabetes year of care programme |
| 8836 | 66AR.00 | diabetes management plan given |
| 6125 | 66AS.00 | diabetic annual review |
| 107464 | 66AS000 | diabetes year of care annual review |
| 18167 | 66AT.00 | annual diabetic blood test |
| 12307 | 66AU.00 | diabetes care by hospital only |
| 28769 | 66AV.00 | diabetic on insulin and oral treatment |
| 50175 | 66AW.00 | diabetic foot risk assessment |
| 46577 | 66AX.00 | diabetes: shared care in pregnancy - diabetol and obstet |
| 26604 | 66AY.00 | diabetic diet - good compliance |
| 25636 | 66Aa.00 | diabetic diet - poor compliance |
| 22823 | 66Ab.00 | diabetic foot examination |
| 10977 | 66Ac.00 | diabetic peripheral neuropathy screening |
| 90301 | 66Ag.00 | insulin needles changed daily |
| 66274 | 66Ah.00 | insulin needles changed for each injection |
| 28873 | 66Ai.00 | diabetic 6 month review |
| 69152 | 66Aj.00 | insulin needles changed less than once a day |
| 83485 | 66Am.00 | insulin dose changed |
| 96010 | 66Ap.00 | insulin treatment initiated |
| 95994 | 66Aq.00 | diabetic foot screen |
| 100791 | 66Ar.00 | insulin treatment stopped |
| 101728 | 66As.00 | diabetic on subcutaneous treatment |
| 101177 | 66At.00 | diabetic dietary review |
| 102434 | 66Au.00 | diabetic erectile dysfunction review |
| 102490 | 66Av.00 | diabetic assessment of erectile dysfunction |
| 102549 | 66Aw.00 | insulin dose |
| 105484 | 66Az.00 | high risk of diabetes mellitus annual review |
| 109643 | 66o1.00 | enquiry about diabetic erectile dysfunction declined |
| 110344 | 66o2.00 | diabetic on non-insulin injectable medication |
| 113661 | 66o3.00 | has diabetes identity card |
| 110379 | 66o5.00 | diabetic on oral treatment and glucagon-like peptide 1 |
| 111483 | 66o6.00 | diabetic on insulin and glucagon-like peptide 1 |
| 49884 | 6761 | diabetic pre-pregnancy counselling |
| 107739 | 679L211 | advice about diabetes and driving |
| 102767 | 67IJ100 | pre-conception advice for diabetes mellitus |
| 18311 | 68A7.00 | diabetic retinopathy screening |
| 19739 | 68A9.00 | diabetic retinopathy screening offered |
| 61021 | 68AB.00 | diabetic digital retinopathy screening offered |
| 11599 | 7276 | pan retinal photocoagulation for diabetes |
| 36798 | 7L10000 | continuous subcutaneous infusion of insulin |
| 17817 | 7L19800 | subcutaneous injection of insulin |
| 61670 | 889A.00 | diab mellit insulin-glucose infus acute myocardial infarct |
| 24363 | 8A13.00 | diabetic stabilisation |
| 11471 | 8B3l.00 | diabetes medication review |
| 12213 | 8BL2.00 | patient on maximal tolerated therapy for diabetes |
| 8414 | 8CA4100 | pt advised re diabetic diet |
| 12483 | 8CAQ.00 | advice about blood glucose control |
| 28856 | 8CP2.00 | transition of diabetes care options discussed |
| 7059 | 8H2J.00 | admit diabetic emergency |
| 35321 | 8H3O.00 | non-urgent diabetic admission |
| 11677 | 8H7r.00 | refer to diabetic foot screener |
| 11018 | 8HBG.00 | diabetic retinopathy 12 month review |
| 18662 | 8HBH.00 | diabetic retinopathy 6 month review |
| 47370 | 8HLE.00 | diabetology d.v. done |
| 64142 | 8Hl1.00 | referral for diabetic retinopathy screening |
| 104287 | 8Hlc.00 | referral to community diabetes service |
| 18824 | 8I3W.00 | diabetic foot examination declined |
| 12262 | 8I3X.00 | diabetic retinopathy screening refused |
| 58159 | 8I3k.00 | insulin therapy declined |
| 58639 | 8I57.00 | patient held diabetic record declined |
| 101456 | 8IAs.00 | diabetic dietary review declined |
| 105937 | 8IEQ.00 | referral to community diabetes specialist nurse declined |
| 106953 | 8IEa.00 | referral to dafne diabetes structured educn prog declined |
| 106679 | 8OA3.00 | provision of written information about diabetes and driving |
| 110087 | 8OAH.00 | provision of written information about diabetes and high bp |
| 113762 | 8OAH.11 | provision of written information about diabetes & hypertensn |
| 112237 | 8OAJ.00 | provision written information abt diabetes & high hba1c levl |
| 109744 | 8OAK.00 | provsn written information about diabetes & high cholesterol |
| 52237 | 9360 | patient held diabetic record issued |
| 108634 | 9NJy.00 | in-house diabetic foot screening |
| 111347 | 9Na5100 | joint consultn pracse nurse & comm diabetes specialist nurse |
| 113796 | 9Na5200 | joint consultation gp & community diabetes specialist nurse |
| 102768 | 9NiZ.00 | did not attend diabetes foot screening |
| 35383 | 9OLD.00 | diabetic patient unsuitable for digital retinal photography |
| 106738 | 9Oy0000 | diabetic foot screening invitation |
| 106723 | 9Oy0200 | diabetic foot screening invitation first letter |
| 106722 | 9Oy0300 | diabetic foot screening invitation second letter |
| 107793 | 9Oy0400 | diabetic foot screening invitation third letter |
| 103798 | 9b92000 | diabetic medicine |
| 106269 | 9m0..00 | diabetic retinopathy screening administrative status |
| 106332 | 9m00.00 | eligible for diabetic retinopathy screening |
| 106441 | 9m01.00 | ineligible for diabetic retinopathy screening |
| 109521 | 9m02.00 | eligibility temporarily inactive for diabetic retinop screen |
| 109520 | 9m03.00 | eligibility permanently inactive for diabetic retinop screen |
| 106327 | 9m04.00 | excluded from diabetic retinopathy screening |
| 106350 | 9m05.00 | excluded from diabetic retinopathy screening as moved away |
| 106352 | 9m06.00 | excluded from diabetic retinopathy screening as deceased |
| 106329 | 9m08.00 | excluded from diabetic retinopathy screening as blind |
| 106351 | 9m09.00 | excluded from diabetic retinop screen as no longer diabetic |
| 106218 | 9m0A.00 | declined diabetic retinopathy screening |
| 106778 | 9m0C.00 | excluded frm diabetic retinopathy screen as terminal illness |
| 107597 | 9m0D.00 | excluded from diabetic retinopthy screen as learn disability |
| 106445 | 9m0E.00 | excluded from diabetic retinopathy screen physical disorder |
| 711 | C10..00 | diabetes mellitus |
| 38986 | C100.00 | diabetes mellitus with no mention of complication |
| 50972 | C100z00 | diabetes mellitus nos with no mention of complication |
| 1682 | C101.00 | diabetes mellitus with ketoacidosis |
| 38617 | C101y00 | other specified diabetes mellitus with ketoacidosis |
| 42505 | C101z00 | diabetes mellitus nos with ketoacidosis |
| 21482 | C102.00 | diabetes mellitus with hyperosmolar coma |
| 72345 | C102z00 | diabetes mellitus nos with hyperosmolar coma |
| 15690 | C103.00 | diabetes mellitus with ketoacidotic coma |
| 59288 | C103y00 | other specified diabetes mellitus with coma |
| 65062 | C103z00 | diabetes mellitus nos with ketoacidotic coma |
| 16502 | C104.00 | diabetes mellitus with renal manifestation |
| 2475 | C104.11 | diabetic nephropathy |
| 13279 | C104y00 | other specified diabetes mellitus with renal complications |
| 35107 | C104z00 | diabetes mellitus with nephropathy nos |
| 33254 | C105.00 | diabetes mellitus with ophthalmic manifestation |
| 47377 | C105y00 | other specified diabetes mellitus with ophthalmic complicatn |
| 34283 | C105z00 | diabetes mellitus nos with ophthalmic manifestation |
| 16230 | C106.00 | diabetes mellitus with neurological manifestation |
| 59903 | C106.11 | diabetic amyotrophy |
| 7795 | C106.12 | diabetes mellitus with neuropathy |
| 16491 | C106.13 | diabetes mellitus with polyneuropathy |
| 61523 | C106y00 | other specified diabetes mellitus with neurological comps |
| 22573 | C106z00 | diabetes mellitus nos with neurological manifestation |
| 35399 | C107.00 | diabetes mellitus with peripheral circulatory disorder |
| 32403 | C107.11 | diabetes mellitus with gangrene |
| 32556 | C107.12 | diabetes with gangrene |
| 112402 | C107y00 | other specified diabetes mellitus with periph circ comps |
| 65025 | C107z00 | diabetes mellitus nos with peripheral circulatory disorder |
| 46290 | C108y00 | other specified diabetes mellitus with multiple comps |
| 64449 | C108z00 | unspecified diabetes mellitus with multiple complications |
| 52236 | C10A.00 | malnutrition-related diabetes mellitus |
| 66675 | C10A000 | malnutrition-related diabetes mellitus with coma |
| 33969 | C10A100 | malnutrition-related diabetes mellitus with ketoacidosis |
| 100347 | C10A500 | malnutritn-relat diabetes melitus wth periph circul complctn |
| 43453 | C10C.00 | diabetes mellitus autosomal dominant |
| 43857 | C10M.00 | lipoatrophic diabetes mellitus |
| 107603 | C10P.00 | diabetes mellitus in remission |
| 113115 | C10Q.00 | maturity onset diabetes of the young type 5 |
| 33343 | C10y.00 | diabetes mellitus with other specified manifestation |
| 10098 | C10yy00 | other specified diabetes mellitus with other spec comps |
| 70821 | C10yz00 | diabetes mellitus nos with other specified manifestation |
| 45491 | C10z.00 | diabetes mellitus with unspecified complication |
| 64283 | C10zy00 | other specified diabetes mellitus with unspecified comps |
| 64357 | C10zz00 | diabetes mellitus nos with unspecified complication |
| 11848 | C314.11 | renal diabetes |
| 23479 | C350011 | bronzed diabetes |
| 52212 | Cyu2.00 | [x]diabetes mellitus |
| 41686 | Cyu2000 | [x]other specified diabetes mellitus |
| 100292 | Cyu2300 | [x]unspecified diabetes mellitus with renal complications |
| 17067 | F171100 | autonomic neuropathy due to diabetes |
| 44033 | F345000 | diabetic mononeuritis multiplex |
| 17247 | F35z000 | diabetic mononeuritis nos |
| 31790 | F372.00 | polyneuropathy in diabetes |
| 5002 | F372.11 | diabetic polyneuropathy |
| 2342 | F372.12 | diabetic neuropathy |
| 48078 | F372000 | acute painful diabetic neuropathy |
| 35785 | F372100 | chronic painful diabetic neuropathy |
| 24571 | F372200 | asymptomatic diabetic neuropathy |
| 39420 | F381300 | myasthenic syndrome due to diabetic amyotrophy |
| 2340 | F381311 | diabetic amyotrophy |
| 37315 | F3y0.00 | diabetic mononeuropathy |
| 1323 | F420.00 | diabetic retinopathy |
| 7069 | F420000 | background diabetic retinopathy |
| 3286 | F420100 | proliferative diabetic retinopathy |
| 2986 | F420200 | preproliferative diabetic retinopathy |
| 10099 | F420300 | advanced diabetic maculopathy |
| 3837 | F420400 | diabetic maculopathy |
| 47584 | F420500 | advanced diabetic retinal disease |
| 10755 | F420600 | non proliferative diabetic retinopathy |
| 30477 | F420700 | high risk proliferative diabetic retinopathy |
| 65463 | F420800 | high risk non proliferative diabetic retinopathy |
| 11626 | F420z00 | diabetic retinopathy nos |
| 17313 | F440700 | diabetic iritis |
| 10659 | F464000 | diabetic cataract |
| 34152 | G73y000 | diabetic peripheral angiopathy |
| 2471 | K01x100 | nephrotic syndrome in diabetes mellitus |
| 105302 | K08yA00 | proteinuric diabetic nephropathy |
| 106360 | K27y700 | erectile dysfunction due to diabetes mellitus |
| 99628 | Kyu0300 | [x]glomerular disorders in diabetes mellitus |
| 109133 | L180700 | pre-existing malnutrition-related diabetes mellitus |
| 55431 | L180X00 | pre-existing diabetes mellitus, unspecified |
| 112365 | Lyu2900 | [x]pre-existing diabetes mellitus, unspecified |
| 7328 | M037200 | cellulitis in diabetic foot |
| 24327 | M271000 | ischaemic ulcer diabetic foot |
| 11663 | M271100 | neuropathic diabetic ulcer - foot |
| 9881 | M271200 | mixed diabetic ulcer - foot |
| 18142 | N030000 | diabetic cheiroarthropathy |
| 57333 | N030011 | diabetic cheiropathy |
| 27891 | N030100 | diabetic charcot arthropathy |
| 21472 | Q441.00 | neonatal diabetes mellitus |
| 53634 | R054200 | [d]gangrene of toe in diabetic |
| 31053 | R054300 | [d]widespread diabetic foot gangrene |
| 68928 | TJ23.00 | adverse reaction to insulins and antidiabetic agents |
| 61210 | TJ23z00 | adverse reaction to insulins and antidiabetic agents nos |
| 65684 | U602311 | [x] adverse reaction to insulins and antidiabetic agents |
| 100033 | U60231E | [x] adverse reaction to insulins and antidiabetic agents nos |
| 10642 | ZC2C800 | dietary advice for diabetes mellitus |
| 16881 | ZV65312 | [v]dietary counselling in diabetes mellitus |

## Cardiovascular disease (medcode, Read)

| medcode | readcode | readterm | cvd_desc |
| --- | --- | --- | --- |
| 59534 | 14NB.00 | H/O: Peripheral vascular disease procedure | pvd |
| 18499 | 662U.00 | Peripheral vascular disease monitoring | pvd |
| 106224 | 9m1..00 | Peripheral vascular disease monitoring invitation | pvd |
| 106260 | 9m10.00 | Peripheral vascular disease monitoring first letter | pvd |
| 106660 | 9m11.00 | Peripheral vascular disease monitoring second letter | pvd |
| 106855 | 9m12.00 | Peripheral vascular disease monitoring third letter | pvd |
| 109034 | 1M11000 | Ischaemic foot pain at rest | pvd |
| 109217 | 1M11100 | Ischaemic foot pain when walking | pvd |
| 9561 | 2G63.00 | Ischaemic toe | pvd |
| 100475 | 8HlP.00 | Referred for peripheral artery disease assessment | pvd |
| 104394 | 9hS0.00 | Excepted frm peripheral arterial dis qual ind: pt unsuitable | pvd |
| 104270 | 9hS1.00 | Except frm peripheral arter dis qual indicat: inform dissent | pvd |
| 72632 | A3A0F00 | Gas gangrene-foot | pvd |
| 35399 | C107.00 | Diabetes mellitus with peripheral circulatory disorder | pvd |
| 70448 | C107000 | Diabetes mellitus, juvenile +peripheral circulatory disorder | pvd |
| 63357 | C107100 | Diabetes mellitus, adult, + peripheral circulatory disorder | pvd |
| 69124 | C107300 | IDDM with peripheral circulatory disorder | pvd |
| 56803 | C107400 | NIDDM with peripheral circulatory disorder | pvd |
| 65025 | C107z00 | Diabetes mellitus NOS with peripheral circulatory disorder | pvd |
| 64446 | C108G00 | Insulin dependent diab mell with peripheral angiopathy | pvd |
| 54212 | C109F00 | Non-insulin-dependent d m with peripheral angiopath | pvd |
| 54899 | C109F11 | Type II diabetes mellitus with peripheral angiopathy | pvd |
| 60699 | C109F12 | Type 2 diabetes mellitus with peripheral angiopathy | pvd |
| 93468 | C10EG00 | Type 1 diabetes mellitus with peripheral angiopathy | pvd |
| 37806 | C10FF00 | Type 2 diabetes mellitus with peripheral angiopathy | pvd |
| 104639 | C10FF11 | Type II diabetes mellitus with peripheral angiopathy | pvd |
| 19155 | G700.11 | Aorto-iliac disease | pvd |
| 14797 | G702.00 | Extremity artery atheroma | pvd |
| 16260 | G702z00 | Extremity artery atheroma NOS | pvd |
| 5943 | G73..00 | Other peripheral vascular disease | pvd |
| 5702 | G73..11 | Peripheral ischaemic vascular disease | pvd |
| 1826 | G73..12 | Ischaemia of legs | pvd |
| 6827 | G73..13 | Peripheral ischaemia | pvd |
| 34638 | G731.00 | Thromboangiitis obliterans | pvd |
| 23497 | G731000 | Buerger's disease | pvd |
| 67401 | G731z00 | Thromboangiitis obliterans NOS | pvd |
| 9204 | G732.00 | Peripheral gangrene | pvd |
| 5414 | G732000 | Gangrene of toe | pvd |
| 12735 | G732100 | Gangrene of foot | pvd |
| 98174 | G733.00 | Ischaemic foot | pvd |
| 105317 | G734.00 | Peripheral arterial disease | pvd |
| 38907 | G73y.00 | Other specified peripheral vascular disease | pvd |
| 34152 | G73y000 | Diabetic peripheral angiopathy | pvd |
| 23871 | G73y100 | Peripheral angiopathic disease EC NOS | pvd |
| 4317 | G73y200 | Acrocyanosis | pvd |
| 22834 | G73y400 | Acroparaesthesia - Schultze's type | pvd |
| 25954 | G73y500 | Acroparaesthesia - Nothnagel's type | pvd |
| 68698 | G73y511 | Nothnagel's vasomotor acroparaesthesia | pvd |
| 3715 | G73y600 | Acroparaesthesia - unspecified | pvd |
| 15272 | G73y700 | Erythrocyanosis | pvd |
| 10500 | G73y800 | Erythromelalgia | pvd |
| 4325 | G73yz00 | Other specified peripheral vascular disease NOS | pvd |
| 3530 | G73z.00 | Peripheral vascular disease NOS | pvd |
| 1517 | G73z000 | Intermittent claudication | pvd |
| 6853 | G73z011 | Claudication | pvd |
| 101866 | G73z012 | Vascular claudication | pvd |
| 2760 | G73zz00 | Peripheral vascular disease NOS | pvd |
| 5650 | G740.12 | Aortoiliac obstruction | pvd |
| 15302 | G742z00 | Peripheral arterial embolism and thrombosis NOS | pvd |
| 73961 | Gyu7400 | [X]Other specified peripheral vascular diseases | pvd |
| 6308 | M271.12 | Ischaemic leg ulcer | pvd |
| 24327 | M271000 | Ischaemic ulcer diabetic foot | pvd |
| 11624 | M271300 | Arterial leg ulcer | pvd |
| 8801 | M271400 | Mixed venous and arterial leg ulcer | pvd |
| 105624 | Pyu2B00 | [X]Oth specified cong malform of peripheral vascular system | pvd |
| 104012 | Q31y400 | Perinatal acrocyanosis | pvd |
| 53634 | R054200 | [D]Gangrene of toe in diabetic | pvd |
| 31053 | R054300 | [D]Widespread diabetic foot gangrene | pvd |
| 14796 | R055000 | [D]Failure of peripheral circulation | pvd |
| 30484 | R055011 | [D]Peripheral circulatory failure | pvd |
| 15133 | 7911300 | replacement of aortic valve nec | vhd |
| 32211 | G121.00 | rheumatic aortic insufficiency | vhd |
| 7963 | G121.12 | aortic regurgitation - rheumatic | vhd |
| 63960 | G122.00 | rheumatic aortic stenosis with insufficiency | vhd |
| 47887 | G541011 | aortic insufficiency, non-rheumatic | vhd |
| 10187 | G541012 | aortic regurgitation, non-rheumatic | vhd |
| 999 | G541100 | aortic stenosis, non-rheumatic | vhd |
| 58810 | G541211 | aortic insufficiency alone, cause unspecified | vhd |
| 1005 | G541212 | aortic regurgitation alone, cause unspecified | vhd |
| 10964 | G541400 | aortic valve stenosis with insufficiency | vhd |
| 9591 | G541500 | aortic stenosis | vhd |
| 6886 | P63..00 | congenital aortic valve stenosis | vhd |
| 8636 | P64..00 | congenital aortic valve insufficiency | vhd |
| 58734 | P640.00 | congenital aortic valve insufficiency, unspecified | vhd |
| 6843 | P64z.00 | congenital aortic valve insufficiency nos | vhd |
| 51879 | G111.00 | rheumatic mitral insufficiency | vhd |
| 22837 | G111.12 | mitral regurgitation - rheumatic | vhd |
| 44488 | G112.00 | mitral stenosis with insufficiency | vhd |
| 44328 | G112.13 | mitral stenosis with regurgitation | vhd |
| 28662 | G113.00 | nonrheumatic mitral valve stenosis | vhd |
| 49355 | G131.00 | mitral stenosis and aortic insufficiency | vhd |
| 17596 | G131.14 | mitral stenosis and aortic regurgitation | vhd |
| 33262 | G132.00 | mitral insufficiency and aortic stenosis | vhd |
| 33907 | G132.13 | mitral regurgitation and aortic stenosis | vhd |
| 94872 | G133.11 | mitral and aortic insufficiency | vhd |
| 11878 | G133.12 | mitral and aortic regurgitation | vhd |
| 40949 | G540.12 | mitral valve insufficiency | vhd |
| 9450 | G540.14 | mitral valve regurgitation | vhd |
| 561 | G540.16 | mitral regurgitation | vhd |
| 61651 | P66..00 | congenital mitral insufficiency | vhd |
| 54088 | G141100 | rheumatic pulmonary insufficiency | vhd |
| 105626 | G141200 | rheumatic pulmonary stenosis and insufficiency | vhd |
| 15640 | G543011 | pulmonary insufficiency, non-rheumatic | vhd |
| 15496 | G543012 | pulmonary regurgitation, non-rheumatic | vhd |
| 38299 | G543213 | pulmonary insufficiency, cause unspecified | vhd |
| 6077 | G543215 | pulmonary regurgitation, cause unspecified | vhd |
| 34932 | G543400 | pulmonary valve stenosis with insufficiency | vhd |
| 65000 | H585.00 | trauma and post-operative pulmonary insufficiency | vhd |
| 96754 | H585200 | pulmonary insufficiency following trauma | vhd |
| 60266 | G140100 | rheumatic tricuspid insufficiency | vhd |
| 21980 | G140111 | tricuspid regurgitation - rheumatic | vhd |
| 93114 | G140200 | rheumatic tricuspid stenosis and insufficiency | vhd |
| 93113 | G14021X | rheumatic tricuspid stenosis and regurgitation | vhd |
| 42128 | G140400 | tricuspid insufficiency, cause unspecified | vhd |
| 9286 | G140413 | tricuspid regurgitation, cause unspecified | vhd |
| 72306 | G140500 | tricuspid stenosis and insufficiency, cause unspecified | vhd |
| 49551 | G140514 | tricuspid stenosis and regurgitation, cause unspecified | vhd |
| 97738 | G542011 | tricuspid insufficiency, non-rheumatic | vhd |
| 35372 | G542012 | tricuspid regurgitation, non-rheumatic | vhd |
| 52271 | G542200 | nonrheumatic tricuspid valve stenosis with insufficiency | vhd |
| 5743 | G54z500 | valvular heart disease | vhd |
| 96444 | AB40300 | histoplasma capsulatum with pericarditis | pericarditis |
| 94380 | AB41300 | histoplasma duboisii with pericarditis | pericarditis |
| 35119 | G501.00 | post infarction pericarditis | pericarditis |
| 50720 | G531.00 | adhesive pericarditis | pericarditis |
| 96101 | G531z00 | adhesive pericarditis nos | pericarditis |
| 20157 | G532.00 | constrictive pericarditis | pericarditis |
| 65807 | G532z00 | constrictive pericarditis nos | pericarditis |
| 18293 | G533.00 | pericardial effusion - noninflammatory | pericarditis |
| 105192 | G536.00 | pericardial effusion | pericarditis |
| 112442 | Gyu5200 | [x]pericarditis in bacterial diseases classified elsewhere | pericarditis |
| 108258 | Gyu5400 | [x]pericarditis in other diseases classified elsewhere | pericarditis |
| 11920 | N000400 | systemic lupus erythematosus with pericarditis | pericarditis |
| 16803 | A364100 | meningococcal pericarditis | pericarditis |
| 9113 | A742100 | coxsackie pericarditis | pericarditis |
| 65897 | A93y000 | syphilitic pericarditis | pericarditis |
| 96449 | A98y200 | gonococcal pericarditis | pericarditis |
| 58769 | G500000 | acute pericarditis - coxsackie | pericarditis |
| 61379 | G500100 | acute pericarditis - meningococcal | pericarditis |
| 112041 | G500200 | acute pericarditis - syphilitic | pericarditis |
| 57126 | G500300 | acute pericarditis - tuberculous | pericarditis |
| 16996 | G500311 | tb - acute pericarditis | pericarditis |
| 103850 | G500500 | acute pericarditis - gonococcal | pericarditis |
| 8411 | G50z111 | viral pericarditis nos | pericarditis |
| 36496 | G50z200 | acute pericarditis - pneumococcal | pericarditis |
| 104081 | G50z300 | acute pericarditis - staphylococcal | pericarditis |
| 59677 | G50z400 | acute pericarditis - streptococcal | pericarditis |
| 24636 | G010.00 | acute rheumatic pericarditis | pericarditis |
| 3399 | G50..00 | acute pericarditis | pericarditis |
| 45311 | G50..11 | pericardial effusion - acute | pericarditis |
| 29551 | G500.00 | acute pericarditis in diseases ec | pericarditis |
| 40956 | G500400 | acute pericarditis - uraemic | pericarditis |
| 15089 | G500z00 | acute pericarditis in diseases ec nos | pericarditis |
| 14646 | G50z.00 | other and unspecified acute pericarditis | pericarditis |
| 27606 | G50z000 | acute pericarditis - unspecified | pericarditis |
| 59102 | G50z100 | acute idiopathic pericarditis | pericarditis |
| 64481 | G50z500 | acute purulent pericarditis unspecified | pericarditis |
| 36755 | G50zz00 | acute pericarditis nos | pericarditis |
| 2520 | G534.00 | pericardial effusion - acute | pericarditis |
| 100907 | Gyu5000 | [x]other forms of acute pericarditis | pericarditis |
| 44376 | G10..00 | chronic rheumatic pericarditis | pericarditis |
| 72628 | G102.00 | chronic rheumatic myopericarditis | pericarditis |
| 107662 | G536000 | chronic pericardial effusion | pericarditis |
| 18877 | G53yz11 | chronic pericarditis | pericarditis |
| 7320 | G343.00 | ischaemic cardiomyopathy | cardiomyopathy |
| 57306 | G554.00 | other primary cardiomyopathies | cardiomyopathy |
| 5141 | G554000 | congestive cardiomyopathy | cardiomyopathy |
| 68766 | G554011 | congestive obstructive cardiomyopathy | cardiomyopathy |
| 7535 | G554400 | primary dilated cardiomyopathy | cardiomyopathy |
| 40834 | G554z00 | other primary cardiomyopathy nos | cardiomyopathy |
| 9402 | G55y.11 | secondary dilated cardiomyopathy | cardiomyopathy |
| 8010 | G551.00 | hypertrophic obstructive cardiomyopathy | cardiomyopathy |
| 3499 | G554300 | hypertrophic non-obstructive cardiomyopathy | cardiomyopathy |
| 70648 | Gyu5M00 | [x]other hypertrophic cardiomyopathy | cardiomyopathy |
| 92266 | Gyu5N00 | [x]other restrictive cardiomyopathy | cardiomyopathy |
| 97780 | G559.00 | arrhythmogenic right ventricular cardiomyopathy | cardiomyopathy |
| 44272 | F391B00 | cardiomyopathy in duchenne muscular dystrophy | cardiomyopathy |
| 3204 | G55..00 | cardiomyopathy | cardiomyopathy |
| 68685 | G552.00 | obscure african cardiomyopathy | cardiomyopathy |
| 41488 | G554100 | constrictive cardiomyopathy | cardiomyopathy |
| 101015 | G554500 | takotsubo cardiomyopathy | cardiomyopathy |
| 104658 | G554511 | stress cardiomyopathy | cardiomyopathy |
| 100966 | G557.00 | nutritional and metabolic cardiomyopathies | cardiomyopathy |
| 64673 | G557z00 | nutritional and metabolic cardiomyopathy nos | cardiomyopathy |
| 55850 | G558.00 | cardiomyopathy in disease ec | cardiomyopathy |
| 70855 | G558000 | cardiomyopathy in friedreich's ataxia | cardiomyopathy |
| 27683 | G558100 | cardiomyopathy in myotonic dystrophy | cardiomyopathy |
| 64837 | G558200 | dystrophic cardiomyopathy | cardiomyopathy |
| 105651 | G558400 | amyloid cardiomyopathy | cardiomyopathy |
| 98020 | G558z00 | cardiomyopathy in diseases ec, nos | cardiomyopathy |
| 42043 | G55y.00 | secondary cardiomyopathy nos | cardiomyopathy |
| 58938 | G55y000 | cardiomyopathy due to drugs and other external agents | cardiomyopathy |
| 22993 | G55z.00 | cardiomyopathy nos | cardiomyopathy |
| 97617 | Gyu5P00 | [x]other cardiomyopathies | cardiomyopathy |
| 98634 | Gyu5R00 | [x]cardiomyopathy in metabolic diseases ce | cardiomyopathy |
| 40095 | L186500 | cardiomyopathy in the puerperium | cardiomyopathy |
| 6434 | 1736 | paroxysmal nocturnal dyspnoea | hf |
| 102627 | 183B.00 | worsening pulmonary oedema | hf |
| 9913 | 1O1..00 | heart failure confirmed | hf |
| 111428 | 2JZ..00 | on optimal heart failure therapy | hf |
| 46672 | 388D.00 | new york heart assoc classification heart failure symptoms | hf |
| 11284 | 585f.00 | echocardiogram shows left ventricular systolic dysfunction | hf |
| 11351 | 585g.00 | echocardiogram shows left ventricular diastolic dysfunction | hf |
| 106198 | 661M500 | heart failure self-management plan agreed | hf |
| 18853 | 662f.00 | new york heart association classification - class i | hf |
| 13189 | 662g.00 | new york heart association classification - class ii | hf |
| 19066 | 662h.00 | new york heart association classification - class iii | hf |
| 51214 | 662i.00 | new york heart association classification - class iv | hf |
| 24503 | 8B29.00 | cardiac failure therapy | hf |
| 32945 | 8CL3.00 | heart failure care plan discussed with patient | hf |
| 32898 | 8H2S.00 | admit heart failure emergency | hf |
| 8464 | G400.00 | acute cor pulmonale | hf |
| 5695 | G41z.11 | chronic cor pulmonale | hf |
| 2062 | G58..00 | heart failure | hf |
| 1223 | G58..11 | cardiac failure | hf |
| 398 | G580.00 | congestive heart failure | hf |
| 2906 | G580.11 | congestive cardiac failure | hf |
| 10079 | G580.12 | right heart failure | hf |
| 10154 | G580.13 | right ventricular failure | hf |
| 9524 | G580.14 | biventricular failure | hf |
| 23707 | G580000 | acute congestive heart failure | hf |
| 32671 | G580100 | chronic congestive heart failure | hf |
| 27884 | G580200 | decompensated cardiac failure | hf |
| 11424 | G580300 | compensated cardiac failure | hf |
| 884 | G581.00 | left ventricular failure | hf |
| 5942 | G581.13 | impaired left ventricular function | hf |
| 5255 | G581000 | acute left ventricular failure | hf |
| 27964 | G582.00 | acute heart failure | hf |
| 101138 | G583.00 | heart failure with normal ejection fraction | hf |
| 101137 | G583.11 | hfnef - heart failure with normal ejection fraction | hf |
| 106897 | G583.12 | heart failure with preserved ejection fraction | hf |
| 104275 | G584.00 | right ventricular failure | hf |
| 4024 | G58z.00 | heart failure nos | hf |
| 17278 | G58z.12 | cardiac failure nos | hf |
| 8966 | G5yy900 | left ventricular systolic dysfunction | hf |
| 12550 | G5yyA00 | left ventricular diastolic dysfunction | hf |
| 104876 | G5yyB00 | right ventricular diastolic dysfunction | hf |
| 107397 | G5yyD00 | left ventricular cardiac dysfunction | hf |
| 108180 | G5yyE00 | right ventricular systolic dysfunction | hf |
| 112120 | L09y200 | cardiac failure following abortive pregnancy | hf |
| 20324 | R2y1000 | [d]cardiorespiratory failure | hf |
| 26242 | ZRad.00 | new york heart assoc classification heart failure symptoms | hf |
| 94870 | G580400 | congestive heart failure due to valvular disease | hf |
| 50157 | G210.00 | malignant hypertensive heart disease | hf |
| 95334 | G210000 | malignant hypertensive heart disease without ccf | hf |
| 72668 | G210100 | malignant hypertensive heart disease with ccf | hf |
| 103046 | G210z00 | malignant hypertensive heart disease nos | hf |
| 52127 | G211100 | benign hypertensive heart disease with ccf | hf |
| 62718 | G21z100 | hypertensive heart disease nos with ccf | hf |
| 67232 | G230.00 | malignant hypertensive heart and renal disease | hf |
| 21837 | G232.00 | hypertensive heart&renal dis wth (congestive) heart failure | hf |
| 57987 | G234.00 | hyperten heart&renal dis+both(congestv)heart and renal fail | hf |
| 22262 | G1yz100 | rheumatic left ventricular failure | hf |
| 20822 | Q48y100 | congenital cardiac failure | hf |
| 15058 | 14A6.00 | h/o: heart failure | hf |
| 46912 | 14AM.00 | h/o: heart failure in last year | hf |
| 100784 | 2126400 | heart failure resolved | hf |
| 12366 | 662T.00 | congestive heart failure monitoring | hf |
| 30779 | 662W.00 | heart failure annual review | hf |
| 83502 | 662p.00 | heart failure 6 month review | hf |
| 103732 | 8CMK.00 | has heart failure management plan | hf |
| 17851 | 8HBE.00 | heart failure follow-up | hf |
| 18793 | 9On..00 | left ventricular dysfunction monitoring administration | hf |
| 60710 | 9On0.00 | left ventricular dysfunction monitoring first letter | hf |
| 60721 | 9On1.00 | left ventricular dysfunction monitoring second letter | hf |
| 72341 | 9On2.00 | left ventricular dysfunction monitoring third letter | hf |
| 92305 | 9On3.00 | left ventricular dysfunction monitoring verbal invite | hf |
| 96484 | 9On4.00 | left ventricular dysfunction monitoring telephone invite | hf |
| 32911 | 9Or..00 | heart failure monitoring administration | hf |
| 19380 | 9Or0.00 | heart failure review completed | hf |
| 90193 | 9Or1.00 | heart failure monitoring telephone invite | hf |
| 90192 | 9Or2.00 | heart failure monitoring verbal invite | hf |
| 72965 | 9Or3.00 | heart failure monitoring first letter | hf |
| 72386 | 9Or4.00 | heart failure monitoring second letter | hf |
| 89650 | 9Or5.00 | heart failure monitoring third letter | hf |
| 34687 | 7A09311 | trendelenburg pulmonary embolectomy | vte |
| 1266 | G401.00 | pulmonary embolism | vte |
| 9701 | G401.12 | pulmonary embolus | vte |
| 18121 | G401000 | post operative pulmonary embolus | vte |
| 96209 | G401100 | recurrent pulmonary embolism | vte |
| 67006 | L096400 | pulmonary embolism following abortive pregnancy | vte |
| 7174 | L43..00 | obstetric pulmonary embolism | vte |
| 68438 | L43..11 | obstetric pulmonary embolus | vte |
| 31313 | L430.00 | obstetric air pulmonary embolism | vte |
| 44192 | L431.00 | amniotic fluid pulmonary embolism | vte |
| 73624 | L431100 | amniotic fluid pulmonary embolism - delivered | vte |
| 49269 | L432.00 | obstetric blood-clot pulmonary embolism | vte |
| 113599 | L432000 | obstetric blood-clot pulmonary embolism unspecified | vte |
| 112578 | L432300 | obstetric blood-clot pulmonary embolism + a/n complication | vte |
| 112710 | L432z00 | obstetric blood-clot pulmonary embolism nos | vte |
| 109337 | L43yz00 | other obstetric pulmonary embolism nos | vte |
| 73569 | L43z.00 | obstetric pulmonary embolism nos | vte |
| 101944 | L43z000 | obstetric pulmonary embolism nos, unspecified | vte |
| 97367 | L43z100 | obstetric pulmonary embolism nos - delivered | vte |
| 98639 | L43zz00 | obstetric pulmonary embolism nos | vte |
| 824 | G801.11 | deep vein thrombosis | vte |
| 3392 | G801.13 | dvt - deep vein thrombosis | vte |
| 106482 | G801900 | thrombophlebitis of the dorsalis pedis vein | vte |
| 22038 | G801D00 | deep vein thrombosis of lower limb | vte |
| 104342 | G801G00 | recurrent deep vein thrombosis | vte |
| 110542 | G801H00 | unprovoked deep vein thrombosis | vte |
| 110449 | G801J00 | provoked deep vein thrombosis | vte |
| 38099 | G80y.11 | phlebitis and/or thrombophlebitis of iliac vein | vte |
| 58166 | G80y400 | thrombophlebitis of the common iliac vein | vte |
| 94496 | G80y500 | thrombophlebitis of the internal iliac vein | vte |
| 58023 | G80y600 | thrombophlebitis of the external iliac vein | vte |
| 91282 | G80y700 | thrombophlebitis of the iliac vein unspecified | vte |
| 70467 | G80y800 | phlebitis and thrombophlebitis of the iliac vein nos | vte |
| 8769 | G81..00 | portal vein thrombosis | vte |
| 57100 | G820.11 | hepatic vein thrombosis | vte |
| 43555 | G822.00 | embolism and thrombosis of the vena cava | vte |
| 97808 | G822000 | thrombosis of inferior vena cava | vte |
| 113022 | Gy20.00 | thrombosis of dialysis arteriovenous graft | vte |
| 54205 | J420200 | thrombus of the superior mesenteric veins | vte |
| 1224 | SP12200 | post operative deep vein thrombosis | vte |
| 3576 | G801.00 | deep vein phlebitis and thrombophlebitis of the leg | vte |
| 25478 | G801.12 | deep vein thrombosis, leg | vte |
| 15382 | G801600 | thrombophlebitis of the femoral vein | vte |
| 55661 | G801700 | thrombophlebitis of the popliteal vein | vte |
| 95077 | G801800 | thrombophlebitis of the anterior tibial vein | vte |
| 61760 | G801A00 | thrombophlebitis of the posterior tibial vein | vte |
| 32002 | G801B00 | deep vein thrombophlebitis of the leg unspecified | vte |
| 42506 | G801C00 | deep vein thrombosis of leg related to air travel | vte |
| 48920 | G801E00 | deep vein thrombosis of leg related to intravenous drug use | vte |
| 98526 | G801F00 | deep vein thrombosis of peroneal vein | vte |
| 27284 | G801z00 | deep vein phlebitis and thrombophlebitis of the leg nos | vte |
| 49715 | F05..00 | phlebitis and thrombophlebitis of intracranial sinuses | vte |
| 31390 | F051.00 | thrombosis of central nervous system venous sinuses | vte |
| 61366 | F051z00 | thrombosis of central nervous system venous sinus nos | vte |
| 41180 | F053.00 | thrombophlebitis of central nervous system venous sinuses | vte |
| 55883 | F053000 | thrombophlebitis of cavernous sinus | vte |
| 70455 | F053100 | thrombophlebitis of superior longitudinal venous sinus | vte |
| 113445 | F053z00 | thrombophlebitis of central nervous system venous sinus nos | vte |
| 65855 | F05z.00 | phlebitis or thrombophlebitis of cns venous sinus nos | vte |
| 37947 | G676.00 | nonpyogenic venous sinus thrombosis | vte |
| 5614 | 14A8100 | h/o: deep vein thrombosis | vte |
| 10280 | 14AC.00 | h/o: pulmonary embolus | vte |
| 19562 | ZV12800 | [v] personal history deep vein thrombosis | vte |
| 17847 | ZV12811 | [v] personal history dvt- deep vein thrombosis | vte |
| 16976 | ZV12900 | [v] personal history of pulmonary embolism | vte |
| 3757 | 3272 | ecg: atrial fibrillation | arrhythmia |
| 6771 | 3273 | ecg: atrial flutter | arrhythmia |
| 84152 | 793M100 | perc transluminal ablation of atrial wall for atrial flutter | arrhythmia |
| 86416 | 793M300 | perc translum ablat conduct sys heart for atrial flutter nec | arrhythmia |
| 107936 | 8OAD.00 | provision of written information about atrial fibrillation | arrhythmia |
| 2212 | G573.00 | atrial fibrillation and flutter | arrhythmia |
| 1664 | G573000 | atrial fibrillation | arrhythmia |
| 1757 | G573100 | atrial flutter | arrhythmia |
| 1268 | G573200 | paroxysmal atrial fibrillation | arrhythmia |
| 35127 | G573300 | non-rheumatic atrial fibrillation | arrhythmia |
| 96277 | G573400 | permanent atrial fibrillation | arrhythmia |
| 96076 | G573500 | persistent atrial fibrillation | arrhythmia |
| 107472 | G573600 | paroxysmal atrial flutter | arrhythmia |
| 23437 | G573z00 | atrial fibrillation and flutter nos | arrhythmia |
| 18746 | 662S.00 | atrial fibrillation monitoring | arrhythmia |
| 9479 | 7936A00 | implant intravenous pacemaker for atrial fibrillation | arrhythmia |
| 105554 | 8CMW200 | atrial fibrillation care pathway | arrhythmia |
| 90187 | 9Os0.00 | atrial fibrillation monitoring first letter | arrhythmia |
| 90188 | 9Os1.00 | atrial fibrillation monitoring second letter | arrhythmia |
| 90189 | 9Os2.00 | atrial fibrillation monitoring third letter | arrhythmia |
| 90190 | 9Os3.00 | atrial fibrillation monitoring verbal invite | arrhythmia |
| 90191 | 9Os4.00 | atrial fibrillation monitoring telephone invite | arrhythmia |
| 28994 | 212R.00 | atrial fibrillation resolved | arrhythmia |
| 26716 | 2426 | o/e - pulse rate tachycardia | arrhythmia |
| 7128 | 2426.11 | o/e - tachycardia | arrhythmia |
| 4924 | 3282 | ecg: ventricular tachycardia | arrhythmia |
| 4940 | G570.00 | paroxysmal supraventricular tachycardia | arrhythmia |
| 1297 | G570000 | paroxysmal atrial tachycardia | arrhythmia |
| 23647 | G570100 | paroxysmal atrioventricular tachycardia | arrhythmia |
| 51845 | G570200 | paroxysmal junctional tachycardia | arrhythmia |
| 29491 | G570300 | paroxysmal nodal tachycardia | arrhythmia |
| 35124 | G570z00 | paroxysmal supraventricular tachycardia nos | arrhythmia |
| 3418 | G571.00 | paroxysmal ventricular tachycardia | arrhythmia |
| 7794 | G571.11 | ventricular tachycardia | arrhythmia |
| 25266 | G572.00 | paroxysmal tachycardia unspecified | arrhythmia |
| 60047 | G572000 | essential paroxysmal tachycardia | arrhythmia |
| 1381 | G572z00 | paroxysmal tachycardia nos | arrhythmia |
| 7005 | G57y700 | sinus tachycardia | arrhythmia |
| 1536 | G57y900 | supraventricular tachycardia nos | arrhythmia |
| 1501 | R050.00 | [d]tachycardia, unspecified | arrhythmia |
| 93387 | R050.12 | [d]postural orthostatic tachycardia syndrome (pots) | arrhythmia |
| 13608 | 2422 | o/e - pulse rate - bradycardia | arrhythmia |
| 1352 | 2422.11 | o/e - bradycardia | arrhythmia |
| 3849 | G57y000 | persistent sinus bradycardia | arrhythmia |
| 18268 | G57y100 | severe sinus bradycardia | arrhythmia |
| 8061 | R059.00 | [d]sinus bradycardia | arrhythmia |
| 3115 | R05W.00 | [d] bradycardia, unspecified | arrhythmia |
| 52423 | Ryu0600 | [x]bradycardia, unspecified | arrhythmia |
| 326 | 181..00 | palpitations | arrhythmia |
| 2975 | 1812 | palpitations | arrhythmia |
| 15616 | 181Z.00 | palpitations nos | arrhythmia |
| 17597 | 327..00 | ecg: supraventricular arrhythmia | arrhythmia |
| 19707 | 328..00 | ecg: ventricular arrhythmia | arrhythmia |
| 29371 | 328Z.00 | ecg: ventricular arrhythmia nos | arrhythmia |
| 4044 | G57..00 | cardiac dysrhythmias | arrhythmia |
| 6503 | G57..11 | cardiac arrhythmias | arrhythmia |
| 426 | G577.00 | sinus arrhythmia | arrhythmia |
| 7827 | G57y.00 | other cardiac dysrhythmias | arrhythmia |
| 31690 | G57yA00 | re-entry ventricular arrhythmia | arrhythmia |
| 31133 | G57yz00 | other cardiac dysrhythmia nos | arrhythmia |
| 1535 | G57z.00 | cardiac dysrhythmia nos | arrhythmia |
| 53893 | Gyu5a00 | [x]other specified cardiac arrhythmias | arrhythmia |
| 4789 | R051.00 | [d]palpitations | arrhythmia |
| 15998 | R051z00 | [d]palpitations nos | arrhythmia |
| 108171 | 8CMWH00 | palpitations care pathway | arrhythmia |
| 45773 | 6A9..00 | atrial fibrillation annual review | arrhythmia |
| 6345 | 14AN.00 | h/o: atrial fibrillation | arrhythmia |
| 30712 | 14AP.00 | history of ventricular tachycardia | arrhythmia |
| 12375 | 14AQ.00 | history of supraventricular tachycardia | arrhythmia |
| 93460 | 14AR.00 | history of atrial flutter | arrhythmia |
| 57495 | G63..11 | infarction - precerebral | stroke |
| 23671 | G63y000 | cerebral infarct due to thrombosis of precerebral arteries | stroke |
| 24446 | G63y100 | cerebral infarction due to embolism of precerebral arteries | stroke |
| 5363 | G64..11 | cva - cerebral artery occlusion | stroke |
| 569 | G64..12 | infarction - cerebral | stroke |
| 6155 | G64..13 | stroke due to cerebral arterial occlusion | stroke |
| 36717 | G640000 | cerebral infarction due to thrombosis of cerebral arteries | stroke |
| 27975 | G641000 | cerebral infarction due to embolism of cerebral arteries | stroke |
| 3149 | G64z.00 | cerebral infarction nos | stroke |
| 15252 | G64z.11 | brainstem infarction nos | stroke |
| 5602 | G64z.12 | cerebellar infarction | stroke |
| 25615 | G64z000 | brainstem infarction | stroke |
| 9985 | G64z200 | left sided cerebral infarction | stroke |
| 10504 | G64z300 | right sided cerebral infarction | stroke |
| 26424 | G64z400 | infarction of basal ganglia | stroke |
| 39344 | G676000 | cereb infarct due cerebral venous thrombosis, nonpyogenic | stroke |
| 40758 | G6W..00 | cereb infarct due unsp occlus/stenos precerebr arteries | stroke |
| 33543 | G6X..00 | cerebrl infarctn due/unspcf occlusn or sten/cerebrl artrs | stroke |
| 91627 | Gyu6300 | [x]cerebrl infarctn due/unspcf occlusn or sten/cerebrl artrs | stroke |
| 53745 | Gyu6400 | [x]other cerebral infarction | stroke |
| 94482 | Gyu6G00 | [x]cereb infarct due unsp occlus/stenos precerebr arteries | stroke |
| 31805 | G62..00 | other and unspecified intracranial haemorrhage | stroke |
| 20284 | G62z.00 | intracranial haemorrhage nos | stroke |
| 101824 | C154211 | adrenocortical haemorrhage | stroke |
| 5051 | G61..00 | intracerebral haemorrhage | stroke |
| 6960 | G61..11 | cva - cerebrovascular accid due to intracerebral haemorrhage | stroke |
| 18604 | G61..12 | stroke due to intracerebral haemorrhage | stroke |
| 31595 | G610.00 | cortical haemorrhage | stroke |
| 40338 | G611.00 | internal capsule haemorrhage | stroke |
| 46316 | G612.00 | basal nucleus haemorrhage | stroke |
| 13564 | G613.00 | cerebellar haemorrhage | stroke |
| 7912 | G614.00 | pontine haemorrhage | stroke |
| 30045 | G616.00 | external capsule haemorrhage | stroke |
| 30202 | G617.00 | intracerebral haemorrhage, intraventricular | stroke |
| 57315 | G618.00 | intracerebral haemorrhage, multiple localized | stroke |
| 107440 | G619.00 | lobar cerebral haemorrhage | stroke |
| 31060 | G61X.00 | intracerebral haemorrhage in hemisphere, unspecified | stroke |
| 28314 | G61X000 | left sided intracerebral haemorrhage, unspecified | stroke |
| 19201 | G61X100 | right sided intracerebral haemorrhage, unspecified | stroke |
| 3535 | G61z.00 | intracerebral haemorrhage nos | stroke |
| 53810 | Gyu6200 | [x]other intracerebral haemorrhage | stroke |
| 96630 | Gyu6F00 | [x]intracerebral haemorrhage in hemisphere, unspecified | stroke |
| 56007 | G601.00 | subarachnoid haemorrhage from carotid siphon and bifurcation | stroke |
| 19412 | G602.00 | subarachnoid haemorrhage from middle cerebral artery | stroke |
| 17326 | G60X.00 | subarachnoid haemorrh from intracranial artery, unspecif | stroke |
| 108668 | Gyu6000 | [x]subarachnoid haemorrhage from other intracranial arteries | stroke |
| 108630 | Gyu6E00 | [x]subarachnoid haemorrh from intracranial artery, unspecif | stroke |
| 108569 | 2Ba2200 | scpe class predom patt c.3 infarct of middle cerebral artery | stroke |
| 95347 | Fyu5700 | [x]other vascular syndroms/brain in cerebrovasculr diseases | stroke |
| 1469 | G66..00 | stroke and cerebrovascular accident unspecified | stroke |
| 1298 | G66..11 | cva unspecified | stroke |
| 6253 | G66..12 | stroke unspecified | stroke |
| 6116 | G66..13 | cva - cerebrovascular accident unspecified | stroke |
| 8443 | G663.00 | brain stem stroke syndrome | stroke |
| 17322 | G664.00 | cerebellar stroke syndrome | stroke |
| 33499 | G665.00 | pure motor lacunar syndrome | stroke |
| 7780 | G667.00 | left sided cva | stroke |
| 12833 | G668.00 | right sided cva | stroke |
| 47607 | L440.11 | cva - cerebrovascular accident in the puerperium | stroke |
| 56279 | L440.12 | stroke in the puerperium | stroke |
| 107195 | 661M700 | stroke self-management plan agreed | stroke |
| 109743 | 661N700 | stroke self-management plan review | stroke |
| 10792 | 662M.00 | stroke monitoring | stroke |
| 56458 | 8HHM.00 | ref to multidisciplinary stroke function improvement service | stroke |
| 105520 | 8Hd6.00 | admission to stroke unit | stroke |
| 31218 | 9Om..00 | stroke/transient ischaemic attack monitoring administration | stroke |
| 28753 | 9Om0.00 | stroke/transient ischaemic attack monitoring first letter | stroke |
| 34245 | 9Om1.00 | stroke/transient ischaemic attack monitoring second letter | stroke |
| 34375 | 9Om2.00 | stroke/transient ischaemic attack monitoring third letter | stroke |
| 51465 | 9Om3.00 | stroke/transient ischaemic attack monitoring verbal invitati | stroke |
| 89913 | 9Om4.00 | stroke/transient ischaemic attack monitoring telephone invte | stroke |
| 4917 | 7017000 | evacuation of subdural haematoma | stroke |
| 4273 | G621.00 | subdural haemorrhage - nontraumatic | stroke |
| 17734 | G622.00 | subdural haematoma - nontraumatic | stroke |
| 18912 | G623.00 | subdural haemorrhage nos | stroke |
| 4107 | 7032000 | evacuation of extradural haematoma | stroke |
| 36178 | G620.00 | extradural haemorrhage - nontraumatic | stroke |
| 31941 | A94y600 | rupture of syphilitic cerebral aneurysm | stroke |
| 47642 | G64z100 | wallenberg syndrome | stroke |
| 105100 | 662M100 | stroke 6 month review | stroke |
| 18686 | 662e.00 | stroke/cva annual review | stroke |
| 107886 | 662e.11 | stroke annual review | stroke |
| 28914 | 662o.00 | haemorrhagic stroke monitoring | stroke |
| 34135 | 14A7.00 | h/o: cva/stroke | stroke |
| 6305 | 14A7.11 | h/o: cva | stroke |
| 5871 | 14A7.12 | h/o: stroke | stroke |
| 66873 | 14AK.00 | h/o: stroke in last year | stroke |
| 100639 | 1M4..00 | central post-stroke pain | stroke |
| 109679 | 5C13.00 | old cerebral infarction on imaging | stroke |
| 104505 | 662M200 | stroke initial post discharge review | stroke |
| 55351 | 7P24200 | delivery of rehabilitation for stroke | stroke |
| 5185 | G64z111 | lateral medullary syndrome | stroke |
| 48149 | G681.00 | sequelae of intracerebral haemorrhage | stroke |
| 43451 | G682.00 | sequelae of other nontraumatic intracranial haemorrhage | stroke |
| 39403 | G683.00 | sequelae of cerebral infarction | stroke |
| 6228 | G68X.00 | sequelae of stroke,not specfd as h'morrhage or infarction | stroke |
| 42248 | ZLEP.00 | discharge from stroke serv | stroke |
| 19348 | ZV12511 | [v]personal history of stroke | stroke |
| 7138 | ZV12512 | [v]personal history of cerebrovascular accident (cva) | stroke |
| 5925 | 2241 | o/e - collapse -cardiac arrest | sca |
| 97560 | 7932111 | cardiac massage - open | sca |
| 10160 | 7937500 | implantation of internal cardiac defibrillator | sca |
| 96801 | 7L1H600 | advanced cardiopulmonary resuscitation | sca |
| 41717 | 853..00 | cardiac massage - external | sca |
| 72762 | 8531 | closed cardiac massage alone | sca |
| 60367 | 8532 | closed cardiac massage+ventil. | sca |
| 28236 | 8532.11 | cardiopulmonary resuscitation | sca |
| 72785 | 853Z.00 | external cardiac massage nos | sca |
| 2099 | G575.00 | cardiac arrest | sca |
| 25407 | G575.11 | cardio-respiratory arrest | sca |
| 33402 | G575.12 | asystole | sca |
| 33899 | G575000 | cardiac arrest with successful resuscitation | sca |
| 51140 | G575200 | electromechanical dissociation with successful resuscitation | sca |
| 7630 | G575300 | electromechanical dissociation | sca |
| 49882 | G575z00 | cardiac arrest, unspecified | sca |
| 72435 | SP11000 | cardiac arrest as a complication of care | sca |
| 34325 | 7937600 | removal of internal cardiac defibrillator | sca |
| 12743 | 790H300 | revascularisation of wall of heart | revascularisation |
| 9498 | 7911 | plastic repair of aortic valve | revascularisation |
| 1756 | 7911.12 | replacement of aortic valve | revascularisation |
| 737 | 792..11 | coronary artery bypass graft operations | revascularisation |
| 18249 | 7920 | saphenous vein graft replacement of coronary artery | revascularisation |
| 8312 | 7920.11 | saphenous vein graft bypass of coronary artery | revascularisation |
| 8679 | 7920000 | saphenous vein graft replacement of one coronary artery | revascularisation |
| 7634 | 7920100 | saphenous vein graft replacement of two coronary arteries | revascularisation |
| 7442 | 7920200 | saphenous vein graft replacement of three coronary arteries | revascularisation |
| 11610 | 7920300 | saphenous vein graft replacement of four+ coronary arteries | revascularisation |
| 7137 | 7920y00 | saphenous vein graft replacement of coronary artery os | revascularisation |
| 51515 | 7920z00 | saphenous vein graft replacement coronary artery nos | revascularisation |
| 9414 | 7921 | other autograft replacement of coronary artery | revascularisation |
| 7134 | 7921.11 | other autograft bypass of coronary artery | revascularisation |
| 44561 | 7921000 | autograft replacement of one coronary artery nec | revascularisation |
| 19413 | 7921100 | autograft replacement of two coronary arteries nec | revascularisation |
| 10209 | 7921200 | autograft replacement of three coronary arteries nec | revascularisation |
| 42708 | 7921300 | autograft replacement of four of more coronary arteries nec | revascularisation |
| 61310 | 7921y00 | other autograft replacement of coronary artery os | revascularisation |
| 7609 | 7921z00 | other autograft replacement of coronary artery nos | revascularisation |
| 31556 | 7922 | allograft replacement of coronary artery | revascularisation |
| 32651 | 7922.11 | allograft bypass of coronary artery | revascularisation |
| 70111 | 7922000 | allograft replacement of one coronary artery | revascularisation |
| 57241 | 7922100 | allograft replacement of two coronary arteries | revascularisation |
| 45886 | 7922200 | allograft replacement of three coronary arteries | revascularisation |
| 45370 | 7922300 | allograft replacement of four or more coronary arteries | revascularisation |
| 59423 | 7922y00 | other specified allograft replacement of coronary artery | revascularisation |
| 48767 | 7922z00 | allograft replacement of coronary artery nos | revascularisation |
| 36011 | 7923.11 | prosthetic bypass of coronary artery | revascularisation |
| 28837 | 7925.11 | creation of bypass from mammary artery to coronary artery | revascularisation |
| 42462 | 7928200 | percut translum balloon angioplasty bypass graft coronary a | revascularisation |
| 34963 | 792D.00 | other bypass of coronary artery | revascularisation |
| 3159 | 792Dy00 | other specified other bypass of coronary artery | revascularisation |
| 33471 | 792Dz00 | other bypass of coronary artery nos | revascularisation |
| 105184 | 792E.00 | percutaneous coronary intervention | revascularisation |
| 107406 | 7.92E+02 | emergency percutaneous coronary intervention | revascularisation |
| 66914 | 7A10.00 | extraanatomic bypass of aorta | revascularisation |
| 23352 | 7A10100 | bypass aorta by anastomosis axillary to femoral artery nec | revascularisation |
| 102684 | 7A10400 | bypass aorta anastomosis axillary artery bi femoral arteries | revascularisation |
| 67839 | 7A10y00 | other specified extraanatomic bypass of aorta | revascularisation |
| 100712 | 7A10z00 | extraanatomic bypass of aorta nos | revascularisation |
| 36952 | 7A12.00 | other bypass of bifurcation of aorta | revascularisation |
| 48755 | 7A12000 | emerg bypass bifurc aorta by anast aorta to femoral artery | revascularisation |
| 2761 | 7A12100 | bypass bifurc aorta by anastom aorta to femoral artery nec | revascularisation |
| 15532 | 7A12300 | bypass bifurcation aorta by anastom aorta to iliac artery | revascularisation |
| 55825 | 7A12y00 | other specified other bypass of bifurcation of aorta | revascularisation |
| 64798 | 7A12z00 | other bypass of bifurcation of aorta nos | revascularisation |
| 31822 | 7A15.00 | other emergency bypass of segment of aorta | revascularisation |
| 66810 | 7A15000 | emerg bypass ascending aorta by anastom aorta to aorta nec | revascularisation |
| 112581 | 7A15100 | emerg bypass thoracic aorta by anatomosis aorta to aorta nec | revascularisation |
| 98082 | 7A15300 | emerg bypass infrarenal aorta by anastom aorta to aorta nec | revascularisation |
| 102031 | 7A15400 | emerg bypass abdominal aorta by anastom aorta to aorta nec | revascularisation |
| 101835 | 7A15y00 | other specified other emergency bypass of segment of aorta | revascularisation |
| 104815 | 7A15z00 | other emergency bypass of segment of aorta nos | revascularisation |
| 37639 | 7A16.00 | other bypass of segment of aorta | revascularisation |
| 53050 | 7A16000 | bypass of ascending aorta by anastomosis aorta to aorta nec | revascularisation |
| 65926 | 7A16100 | bypass of thoracic aorta by anastomosis aorta to aorta nec | revascularisation |
| 61978 | 7A16300 | bypass of infrarenal aorta by anastomosis aorta to aorta nec | revascularisation |
| 5597 | 7A16400 | bypass of abdominal aorta by anastomosis aorta to aorta nec | revascularisation |
| 59518 | 7A16y00 | other specified other bypass of segment of aorta | revascularisation |
| 42751 | 7A16z00 | other bypass of segment of aorta nos | revascularisation |
| 43449 | 7A20100 | intracranial bypass to carotid artery | revascularisation |
| 20811 | 7A20200 | bypass to carotid artery nec | revascularisation |
| 91775 | 7A20700 | intracranial bypass from carotid artery nec | revascularisation |
| 29183 | 7A26000 | bypass of subclavian artery nec | revascularisation |
| 58191 | 7A26100 | bypass of axillary artery nec | revascularisation |
| 37546 | 7A26700 | bypass of brachial artery nec | revascularisation |
| 49727 | 7A30100 | bypass of renal artery | revascularisation |
| 88652 | 7A33000 | bypass of coeliac artery nec | revascularisation |
| 52331 | 7A33100 | bypass of superior mesenteric artery nec | revascularisation |
| 101588 | 7A33200 | bypass of inferior mesenteric artery nec | revascularisation |
| 94643 | 7A33H00 | bypass of visceral branch of abdominal aorta nec | revascularisation |
| 21927 | 7A41.00 | other bypass of iliac artery | revascularisation |
| 101910 | 7A41.11 | other bypass of iliac artery by anastomosis | revascularisation |
| 28616 | 7A41100 | bypass iliac artery by iliac/femoral artery anastomosis nec | revascularisation |
| 72448 | 7A41200 | emerg bypass iliac artery by femoral/femoral art anast nec | revascularisation |
| 36443 | 7A41300 | bypass iliac artery by femoral/femoral art anastomosis nec | revascularisation |
| 68141 | 7A41400 | emerg bypass comm iliac art by aorta/com iliac art anast nec | revascularisation |
| 66917 | 7A41600 | emerg bypass leg artery by aorta/com fem art anastomosis nec | revascularisation |
| 32492 | 7A41900 | bypass common iliac artery by aorta/com iliac art anast nec | revascularisation |
| 55554 | 7A41B00 | bypass leg artery by aorta/com femoral art anastomosis nec | revascularisation |
| 66804 | 7A41C00 | bypass leg artery by aorta/deep femoral art anastomosis nec | revascularisation |
| 100036 | 7A41D00 | bypass iliac artery by iliac/iliac artery anastomosis nec | revascularisation |
| 112861 | 7A41E00 | emergency bypass of iliac artery by unspecified anastomosis | revascularisation |
| 52357 | 7A41y00 | other specified other bypass of iliac artery | revascularisation |
| 38921 | 7A41z00 | other bypass of iliac artery nos | revascularisation |
| 9099 | 7A47.00 | other emergency bypass of femoral artery or popliteal artery | revascularisation |
| 100113 | 7A47.12 | other emergency bypass of common femoral artery | revascularisation |
| 63238 | 7A47.13 | other emergency bypass of deep femoral artery | revascularisation |
| 39776 | 7A47.14 | other emergency bypass of popliteal artery | revascularisation |
| 97606 | 7A47.15 | other emergency bypass of superficial femoral artery | revascularisation |
| 11766 | 7A47.16 | other emergency bypass of femoral artery | revascularisation |
| 52342 | 7A47200 | emerg bypass femoral art by fem/pop a anast c vein graft nec | revascularisation |
| 60693 | 7A47300 | emerg bypass pop art by pop/pop art anast c vein graft nec | revascularisation |
| 96255 | 7A47600 | emerg bypass femoral art by fem/tib a anast c vein graft nec | revascularisation |
| 66879 | 7A47700 | emerg bypass pop art by pop/tib art anast c vein graft nec | revascularisation |
| 112511 | 7A47A00 | emerg bypass fem art by fem/peron a anast c vein graft nec | revascularisation |
| 62775 | 7A47B00 | emerg bypass pop art by pop/peron art anast c vein graft nec | revascularisation |
| 48939 | 7A47C00 | emerg bypass femoral artery by fem/fem art anastomosis nec | revascularisation |
| 70922 | 7A47D00 | emerg bypass popliteal artery by pop/fem art anastomosis nec | revascularisation |
| 65692 | 7A47y00 | other emergency bypass of femoral or popliteal artery os | revascularisation |
| 68320 | 7A47z00 | other emergency bypass of femoral or popliteal artery nos | revascularisation |
| 24692 | 7A48.00 | other bypass of femoral artery or popliteal artery | revascularisation |
| 61974 | 7A48.11 | other bypass of femoral or popliteal artery by anastomosis | revascularisation |
| 37787 | 7A48.12 | other bypass of common femoral artery | revascularisation |
| 113851 | 7A48.13 | other bypass of deep femoral artery | revascularisation |
| 18060 | 7A48.14 | other bypass of femoral artery | revascularisation |
| 12331 | 7A48.15 | other bypass of popliteal artery | revascularisation |
| 40732 | 7A48.16 | other bypass of superficial femoral artery | revascularisation |
| 27580 | 7A48000 | bypass femoral artery by fem/pop art anast c prosthesis nec | revascularisation |
| 64555 | 7A48100 | bypass popliteal artery by pop/pop a anast c prosthesis nec | revascularisation |
| 28030 | 7A48200 | bypass femoral artery by fem/pop art anast c vein graft nec | revascularisation |
| 24097 | 7A48300 | bypass popliteal artery by pop/pop a anast c vein graft nec | revascularisation |
| 39877 | 7A48400 | bypass femoral artery by fem/tib art anast c prosthesis nec | revascularisation |
| 60465 | 7A48500 | bypass popliteal artery by pop/tib a anast c prosthesis nec | revascularisation |
| 41823 | 7A48600 | bypass femoral artery by fem/tib art anast c vein graft nec | revascularisation |
| 48700 | 7A48700 | bypass popliteal artery by pop/tib a anast c vein graft nec | revascularisation |
| 67982 | 7A48800 | bypass femoral artery by fem/peron a anast c prosthesis nec | revascularisation |
| 107158 | 7A48900 | bypass popliteal artery by pop/peron art anast c prosth nec | revascularisation |
| 53675 | 7A48A00 | bypass femoral artery by fem/peron a anast c vein graft nec | revascularisation |
| 68412 | 7A48B00 | bypass popliteal art by pop/peron art anast c vein graft nec | revascularisation |
| 45428 | 7A48C00 | bypass femoral artery by femoral/femoral art anastomosis nec | revascularisation |
| 42115 | 7A48D00 | bypass popliteal artery by pop/fem artery anastomosis nec | revascularisation |
| 42640 | 7A48y00 | other bypass of femoral artery or popliteal artery os | revascularisation |
| 2066 | 7A48z00 | other bypass of femoral artery or popliteal artery nos | revascularisation |
| 31889 | 7A64.00 | other bypass operations on vein | revascularisation |
| 62583 | 7A64y00 | other specified bypass operation on vein | revascularisation |
| 28748 | 7A64z00 | bypass operation on vein nos | revascularisation |
| 18393 | 7A66000 | crossover graft of saphenous vein | revascularisation |
| 40971 | 7A66011 | palma crossover graft of saphenous vein | revascularisation |
| 3827 | 7M36000 | cardiopulmonary bypass | revascularisation |
| 100574 | 7M36400 | modified ultrafiltration adjunct cardiopulmonary bypass | revascularisation |
| 12734 | SP07600 | coronary artery bypass graft occlusion | revascularisation |
| 12229 | G30X000 | acute st segment elevation myocardial infarction | stemi |
| 10562 | G307100 | acute non-st segment elevation myocardial infarction | nstemi |
| 7783 | 323..00 | ECG: myocardial infarction | mi_nos |
| 55401 | 3235 | ECG: subendocardial infarct | mi_nos |
| 59032 | 323Z.00 | ECG: myocardial infarct NOS | mi_nos |
| 61670 | 889A.00 | Diab mellit insulin-glucose infus acute myocardial infarct | mi_nos |
| 112296 | 9bH1.00 | EMIS - LV | mi_nos |
| 113637 | BBr4100 | [M]Acute erythraemia | mi_nos |
| 241 | G30..00 | Acute myocardial infarction | mi_nos |
| 13566 | G30..11 | Attack - heart | mi_nos |
| 30421 | G30..13 | Cardiac rupture following myocardial infarction (MI) | mi_nos |
| 1204 | G30..14 | Heart attack | mi_nos |
| 1677 | G30..15 | MI - acute myocardial infarction | mi_nos |
| 17689 | G30..17 | Silent myocardial infarction | mi_nos |
| 12139 | G300.00 | Acute anterolateral infarction | mi_nos |
| 5387 | G301.00 | Other specified anterior myocardial infarction | mi_nos |
| 40429 | G301000 | Acute anteroapical infarction | mi_nos |
| 17872 | G301100 | Acute anteroseptal infarction | mi_nos |
| 14897 | G301z00 | Anterior myocardial infarction NOS | mi_nos |
| 8935 | G302.00 | Acute inferolateral infarction | mi_nos |
| 29643 | G303.00 | Acute inferoposterior infarction | mi_nos |
| 23892 | G304.00 | Posterior myocardial infarction NOS | mi_nos |
| 14898 | G305.00 | Lateral myocardial infarction NOS | mi_nos |
| 63467 | G306.00 | True posterior myocardial infarction | mi_nos |
| 3704 | G307.00 | Acute subendocardial infarction | mi_nos |
| 9507 | G307000 | Acute non-Q wave infarction | mi_nos |
| 1678 | G308.00 | Inferior myocardial infarction NOS | mi_nos |
| 30330 | G309.00 | Acute Q-wave infarct | mi_nos |
| 32854 | G30B.00 | Acute posterolateral myocardial infarction | mi_nos |
| 29758 | G30X.00 | Acute transmural myocardial infarction of unspecif site | mi_nos |
| 34803 | G30y.00 | Other acute myocardial infarction | mi_nos |
| 28736 | G30y000 | Acute atrial infarction | mi_nos |
| 62626 | G30y100 | Acute papillary muscle infarction | mi_nos |
| 41221 | G30y200 | Acute septal infarction | mi_nos |
| 46017 | G30yz00 | Other acute myocardial infarction NOS | mi_nos |
| 14658 | G30z.00 | Acute myocardial infarction NOS | mi_nos |
| 18842 | G35..00 | Subsequent myocardial infarction | mi_nos |
| 45809 | G350.00 | Subsequent myocardial infarction of anterior wall | mi_nos |
| 38609 | G351.00 | Subsequent myocardial infarction of inferior wall | mi_nos |
| 72562 | G353.00 | Subsequent myocardial infarction of other sites | mi_nos |
| 46166 | G35X.00 | Subsequent myocardial infarction of unspecified site | mi_nos |
| 32272 | G38..00 | Postoperative myocardial infarction | mi_nos |
| 46112 | G380.00 | Postoperative transmural myocardial infarction anterior wall | mi_nos |
| 46276 | G381.00 | Postoperative transmural myocardial infarction inferior wall | mi_nos |
| 106812 | G383.00 | Postoperative transmural myocardial infarction unspec site | mi_nos |
| 41835 | G384.00 | Postoperative subendocardial myocardial infarction | mi_nos |
| 68748 | G38z.00 | Postoperative myocardial infarction, unspecified | mi_nos |
| 96838 | Gyu3400 | [X]Acute transmural myocardial infarction of unspecif site | mi_nos |
| 109035 | Gyu3500 | [X]Subsequent myocardial infarction of other sites | mi_nos |
| 99991 | Gyu3600 | [X]Subsequent myocardial infarction of unspecified site | mi_nos |
| 2491 | G30..12 | Coronary thrombosis | mi_nos |
| 13571 | G30..16 | Thrombosis - coronary | mi_nos |
| 17133 | G30A.00 | Mural thrombosis | mi_nos |
| 68357 | G31y100 | Microinfarction of heart | mi_nos |
| 35674 | 14A3.00 | H/O: myocardial infarct <60 | mi_nos |
| 40399 | 14A4.00 | H/O: myocardial infarct >60 | mi_nos |
| 50372 | 14AH.00 | H/O: Myocardial infarction in last year | mi_nos |
| 100139 | 14AT.00 | History of myocardial infarction | mi_nos |
| 39904 | 3232 | ECG: old myocardial infarction | mi_nos |
| 23579 | G310.00 | Postmyocardial infarction syndrome | mi_nos |
| 15661 | G310.11 | Dressler's syndrome | mi_nos |
| 4017 | G32..00 | Old myocardial infarction | mi_nos |
| 16408 | G32..11 | Healed myocardial infarction | mi_nos |
| 17464 | G32..12 | Personal history of myocardial infarction | mi_nos |
| 9555 | G33z500 | Post infarct angina | mi_nos |
| 36423 | G36..00 | Certain current complication follow acute myocardial infarct | mi_nos |
| 24126 | G360.00 | Haemopericardium/current comp folow acut myocard infarct | mi_nos |
| 23708 | G361.00 | Atrial septal defect/curr comp folow acut myocardal infarct | mi_nos |
| 37657 | G362.00 | Ventric septal defect/curr comp fol acut myocardal infarctn | mi_nos |
| 59189 | G363.00 | Ruptur cardiac wall w'out haemopericard/cur comp fol ac MI | mi_nos |
| 59940 | G364.00 | Ruptur chordae tendinae/curr comp fol acute myocard infarct | mi_nos |
| 69474 | G365.00 | Rupture papillary muscle/curr comp fol acute myocard infarct | mi_nos |
| 29553 | G366.00 | Thrombosis atrium,auric append&vent/curr comp foll acute MI | mi_nos |
| 113358 | Gyu3100 | [X]Other current complicatns following acute myocard infarct | mi_nos |
| 1430 | G33..00 | angina pectoris | angina |
| 20095 | G330.00 | angina decubitus | angina |
| 18125 | G330000 | nocturnal angina | angina |
| 29902 | G330z00 | angina decubitus nos | angina |
| 25842 | G33z.00 | angina pectoris nos | angina |
| 54535 | G33z100 | stenocardia | angina |
| 7696 | G33z200 | syncope anginosa | angina |
| 1414 | G33z300 | angina on effort | angina |
| 26863 | G33z600 | new onset angina | angina |
| 12804 | G33z700 | stable angina | angina |
| 28554 | G33zz00 | angina pectoris nos | angina |
| 24540 | G34y000 | chronic coronary insufficiency | angina |
| 39546 | Gyu3000 | [x]other forms of angina pectoris | angina |
| 103655 | 187..00 | frequency of angina | angina |
| 107967 | 661M000 | angina self-management plan agreed | angina |
| 109391 | 661N000 | angina self-management plan review | angina |
| 108504 | 662K400 | angina self management plan commenced | angina |
| 108056 | 8IEY.00 | referral to angina plan self-management programme declined | angina |
| 107574 | 8T04.00 | referral to angina plan self-management programme | angina |
| 4656 | G311.11 | crescendo angina | angina |
| 1431 | G311.13 | unstable angina | angina |
| 19655 | G311.14 | angina at rest | angina |
| 7347 | G311100 | unstable angina | angina |
| 17307 | G311200 | angina at rest | angina |
| 9276 | G31y000 | acute coronary insufficiency | angina |
| 66388 | G33z000 | status anginosus | angina |
| 34328 | G311300 | refractory angina | angina |
| 8568 | G37..00 | cardiac syndrome x | angina |
| 12986 | G331.00 | prinzmetal's angina | angina |
| 11048 | G331.11 | variant angina pectoris | angina |
| 36854 | G332.00 | coronary artery spasm | angina |
| 13185 | 662K.00 | angina control | angina |
| 19542 | 662K000 | angina control - good | angina |
| 15373 | 662K100 | angina control - poor | angina |
| 14782 | 662K200 | angina control - improving | angina |
| 29300 | 662K300 | angina control - worsening | angina |
| 108506 | 662K500 | angina self management plan completed | angina |
| 15349 | 662Kz00 | angina control nos | angina |
| 18118 | G311400 | worsening angina | angina |
| 6336 | 14A5.00 | h/o: angina pectoris | angina |
| 57062 | 14AJ.00 | h/o: angina in last year | angina |
| 1344 | G340.12 | coronary artery disease | coronary |
| 3999 | G340000 | single coronary vessel disease | coronary |
| 5254 | G340100 | double coronary vessel disease | coronary |
| 105479 | G39..00 | coronary microvascular disease | coronary |

## Hypertension (medcode, Read)

| medcode | readcode | readterm |
| --- | --- | --- |
| 204 | G2...00 | hypertensive disease |
| 245 | G410.00 | primary pulmonary hypertension |
| 351 | G20..11 | high blood pressure |
| 799 | G20..00 | essential hypertension |
| 1894 | G201.00 | benign essential hypertension |
| 2666 | 14A2.00 | h/o: hypertension |
| 3425 | 662O.00 | on treatment for hypertension |
| 3712 | G20z.11 | hypertension nos |
| 3979 | G672.00 | hypertensive encephalopathy |
| 4372 | G202.00 | systolic hypertension |
| 4668 | G22..00 | hypertensive renal disease |
| 6702 | F421300 | hypertensive retinopathy |
| 7057 | G2z..00 | hypertensive disease nos |
| 7329 | G24..00 | secondary hypertension |
| 8732 | G2...11 | bp - hypertensive disease |
| 10818 | G20z.00 | essential hypertension nos |
| 11056 | 8BL0.00 | patient on maximal tolerated antihypertensive therapy |
| 12680 | 8CR4.00 | hypertension clinical management plan |
| 12948 | 662H.00 | hypertension treatm.stopped |
| 13188 | 662G.00 | hypertensive treatm.changed |
| 15106 | G22z.00 | hypertensive renal disease nos |
| 15377 | G200.00 | malignant essential hypertension |
| 16059 | G24z.00 | secondary hypertension nos |
| 16565 | 6627 | good hypertension control |
| 18057 | 8B26.00 | antihypertensive therapy |
| 18482 | 662c.00 | hypertension six month review |
| 18590 | 662b.00 | moderate hypertension control |
| 18765 | G2y..00 | other specified hypertensive disease |
| 19070 | 662d.00 | hypertension annual review |
| 20497 | TJC7z00 | adverse reaction to antihypertensives nos |
| 21660 | TJC7.00 | adverse reaction to other antihypertensives |
| 21826 | 662F.00 | hypertension treatm. started |
| 22333 | 8I3N.00 | hypertension treatment refused |
| 25371 | G241000 | secondary benign renovascular hypertension |
| 26347 | G8y3.00 | chronic peripheral venous hypertension |
| 27511 | 6628 | poor hypertension control |
| 29310 | G22z.11 | renal hypertension |
| 30770 | U60C511 | [x] adverse reaction to other antihypertensives |
| 30776 | 6629 | hypertension:follow-up default |
| 31341 | G24z100 | hypertension secondary to drug |
| 31387 | G24z000 | secondary renovascular hypertension nos |
| 31755 | G240.00 | secondary malignant hypertension |
| 31816 | G672.11 | hypertensive crisis |
| 34065 | G41y000 | secondary pulmonary hypertension |
| 34744 | G244.00 | hypertension secondary to endocrine disorders |
| 37086 | F404200 | blind hypertensive eye |
| 39649 | G220.00 | malignant hypertensive renal disease |
| 42229 | G24zz00 | secondary hypertension nos |
| 43664 | L127.00 | pre-eclampsia or eclampsia with pre-existing hypertension |
| 43935 | G221.00 | benign hypertensive renal disease |
| 44350 | U60C51A | [x] adverse reaction to antihypertensives nos |
| 44549 | L128.00 | pre-exist hypertension compl preg childbirth and puerperium |
| 51635 | G241z00 | secondary benign hypertension nos |
| 52621 | L128200 | pre-exist 2ndry hypertens comp preg childbth and puerperium |
| 55603 | 7Q01000 | primary pulmonary hypertension drugs band 1 |
| 57288 | G241.00 | secondary benign hypertension |
| 59383 | G240000 | secondary malignant renovascular hypertension |
| 62432 | L122z00 | other pre-existing hypertension in preg/childb/puerp nos |
| 63164 | U60C500 | [x]oth antihyperten drug caus advers eff in therap use, nec |
| 63260 | SLC6z00 | hypertensive agent poisoning nos |
| 63946 | 7Q01100 | primary pulmonary hypertension drugs band 2 |
| 65081 | 7Q01200 | primary pulmonary hypertension drugs band 3 |
| 66567 | L122.00 | other pre-existing hypertension in preg/childbirth/puerp |
| 69753 | Gyu2.00 | [x]hypertensive diseases |
| 72030 | L122100 | other pre-existing hypertension in preg/childb/puerp - deliv |
| 72226 | SLC6.00 | other hypertensive agent poisoning |
| 73293 | G240z00 | secondary malignant hypertension nos |
| 73586 | L122000 | other pre-existing hypertension in preg/childb/puerp unspec |
| 83473 | G203.00 | diastolic hypertension |
| 85944 | 7Q01.00 | high cost hypertension drugs |
| 90875 | 7Q01300 | primary pulmonary hypertension drugs band 4 |
| 93055 | L127z00 | pre-eclampsia or eclampsia + pre-existing hypertension nos |
| 95359 | 662r.00 | trial withdrawal of antihypertensive therapy |
| 96743 | L122300 | other pre-exist hypertension in preg/childb/puerp-not deliv |
| 97533 | Gyu2100 | [x]hypertension secondary to other renal disorders |
| 99259 | 662q.00 | trial reduction of antihypertensive therapy |
| 101649 | 7Q01y00 | other specified high cost hypertension drugs |
| 102406 | 662P000 | hypertension 9 month review |
| 102444 | G41y100 | thromboembolic pulmonary hypertension |
| 102458 | Gyu2000 | [x]other secondary hypertension |
| 105274 | G28..00 | stage 2 hypertension (nice - nat ins for hth clin excl 2011) |
| 105316 | G25..11 | stage 1 hypertension |
| 105371 | G25..00 | stage 1 hypertension (nice - nat ins for hth clin excl 2011) |
| 105480 | G27..00 | hypertension resistant to drug therapy |
| 105487 | G26..11 | severe hypertension |
| 105989 | G26..00 | severe hypertension (nat inst for health clinical ex 2011) |
| 106279 | 8IA5.00 | trial withdrawal of antihypertensive therapy declined |
| 107704 | G20..12 | primary hypertension |
| 108136 | G250.00 | stage 1 hyperten (nice 2011) without evidnce end organ damge |
| 109611 | 661M600 | hypertension self-management plan agreed |
| 109797 | G251.00 | stage 1 hyperten (nice 2011) with evidnce end organ damge |
| 110631 | 661N600 | hypertension self-management plan review |
| 112611 | SyuFT00 | [x]poisoning by other antihypertensive drugs, nec |

## Hypertension (ICD-10)

| icd | description |
| --- | --- |
| i10 | essential (primary) hypertension |
| i11 | hypertensive heart disease |
| i11.0 | hypertensive heart disease with (congestive) heart failure |
| i11.9 | hypertensive heart disease without (congestive) heart failure |
| i12 | hypertensive renal disease |
| i12.0 | hypertensive renal disease with renal failure |
| i12.9 | hypertensive renal disease without renal failure |
| i13 | hypertensive heart and renal disease |
| i13.0 | hypertensive heart and renal disease with (congestive) heart failure |
| i13.1 | hypertensive heart and renal disease with renal failure |
| i13.2 | hypertensive heart and renal disease with both (congestive) heart failure and renal failure |
| i13.9 | hypertensive heart and renal disease, unspecified |
| i15 | secondary hypertension |
| i15.0 | renovascular hypertension |
| i15.1 | hypertension secondary to other renal disorders |
| i15.2 | hypertension secondary to endocrine disorders |
| i15.8 | other secondary hypertension |
| i15.9 | secondary hypertension, unspecified |
| i67.4 | hypertensive encephalopathy |

## Autoimmune disease (medcode, Read)

| medcode | readterm |
| --- | --- |
| 162 | psoriasis unspecified |
| 172 | psoriasis nos |
| 476 | psoriatic arthropathy |
| 593 | Crohn's disease |
| 704 | Ulcerative colitis |
| 1515 | coeliac disease |
| 1784 | Ulcerative colitis and/or proctitis |
| 1796 | Inflammatory bowel disease |
| 2945 | psoriasis plantaris |
| 3193 | guttate psoriasis |
| 3437 | h/o: psoriasis |
| 3509 | gluten enteropathy |
| 3733 | psoriasis and similar disorders |
| 3859 | Pulmonary sarcoidosis |
| 3865 | Sarcoidosis |
| 4125 | Lupus erythematosus |
| 4231 | pustular psoriasis |
| 5133 | Idiopathic proctocolitis |
| 5638 | primary biliary cirrhosis |
| 5749 | H/O: ulcerative colitis |
| 6538 | Crohn's colitis |
| 6650 | Ulcerative proctocolitis |
| 7522 | Lupus erythematosus NOS |
| 7871 | Systemic lupus erythematosus |
| 8014 | psoriasis palmaris |
| 8347 | Ulcerative proctitis |
| 9359 | Crohn's disease of the small bowel NOS |
| 11119 | CDAI - Crohn's disease activity index |
| 11286 | Regional enteritis - Crohn's disease |
| 11337 | Crohn's disease activity index |
| 11761 | scalp psoriasis |
| 11920 | Systemic lupus erythematosus with pericarditis |
| 12500 | psoriatic arthropathy nos |
| 12575 | Juvenile arthritis in Crohn's disease |
| 15207 | Idiopathic proctocolitis NOS |
| 15773 | Regional ileocolitis |
| 16365 | sprue - nontropical |
| 17094 | erythrodermic psoriasis |
| 17641 | Arthropathy in ulcerative colitis |
| 18755 | psoriasis discoidea |
| 20007 | Disseminated lupus erythematosus |
| 20222 | psoriasis universalis |
| 20480 | Arthropathy in Crohn's disease |
| 20688 | Crohn's disease of the large bowel NOS |
| 21104 | psoriasis annularis |
| 21503 | arthritis mutilans |
| 21633 | psoriasis geographica |
| 22205 | Lupus nephritis |
| 22501 | other psoriasis |
| 22516 | Exacerbation of ulcerative colitis |
| 24136 | psoriasis punctata |
| 24550 | Other idiopathic proctocolitis |
| 24858 | Ulcerative rectosigmoiditis |
| 25390 | Subacute cutaneous lupus erythematosus |
| 26368 | psoriasis spondylitica |
| 26405 | Hepatic granulomas in sarcoidosis |
| 27769 | Sarcoidosis of skin |
| 28456 | juvenile arthritis in psoriasis |
| 28476 | Crohn's disease of the terminal ileum |
| 28952 | Lupus pernio |
| 29519 | Systemic lupus erythematosus with organ or sys involv |
| 29616 | Orofacial Crohn's disease |
| 30210 | psoriasis vulgaris |
| 30272 | psoriasis circinata |
| 30433 | Ulcerative (chronic) enterocolitis |
| 30975 | psoriasis and similar disorders nos |
| 31564 | Lung disease with systemic lupus erythematosus |
| 32149 | distal interphalangeal psoriatic arthropathy |
| 33449 | Lupus erythematosus chronicus |
| 33456 | Ulcerative proctocolitis NOS |
| 33980 | Sarcoidosis of lung |
| 34437 | Sarcoid myocarditis |
| 36942 | Drug-induced systemic lupus erythematosus |
| 39278 | Crohn's disease of the ileum NOS |
| 40613 | Sarcoid arthropathy |
| 40751 | Polyneuropathy in sarcoidosis |
| 40797 | Lupus erythematosus migrans |
| 41149 | other psoriasis and similar disorders |
| 42008 | psoriasis diffusa |
| 42719 | Systemic lupus erythematosus NOS |
| 42822 | Ulcerative (chronic) ileocolitis |
| 43090 | Other idiopathic proctocolitis NOS |
| 44095 | Polyneuropathy in disseminated lupus erythematosus |
| 44310 | coeliac disease nos |
| 44426 | Regional enteritis of the large bowel |
| 44984 | Lupus erythematosus tumidus |
| 45726 | Systemic lupus erythematosus disease activity index |
| 46148 | Lupus erythematosus profundus |
| 47037 | Sarcoid heart disease |
| 47672 | Nephrotic syndrome in systemic lupus erythematosus |
| 47718 | Myositis in sarcoidosis |
| 48257 | psoriasis inveterata |
| 48732 | Ulcerative ileocolitis |
| 49075 | Sarcoidosis of lymph nodes |
| 49454 | Meningitis due to sarcoidosis |
| 51576 | Regional enteritis of the small bowel |
| 51578 | Granulomatous enteritis |
| 51798 | Systemic lupus activity measure |
| 52449 | Regional enteritis NOS |
| 52519 | Myopathy due to sarcoidosis |
| 53743 | [X]Other ulcerative colitis |
| 55612 | Multiple cranial nerve palsies in sarcoidosis |
| 57966 | gee - herter disease |
| 58706 | [X]Other forms of systemic lupus erythematosus |
| 58841 | Sarcoidosis of lung with sarcoidosis of lymph nodes |
| 59107 | [x]other psoriatic arthropathies |
| 59994 | Crohn's disease NOS |
| 60169 | psoriasis ostracea |
| 62397 | congenital coeliac disease |
| 62628 | Regional enteritis of the colon |
| 63036 | Regional enteritis of the jejunum |
| 63195 | acquired coeliac disease |
| 63955 | Lupus erythematosus unguium mutilans |
| 64773 | Regional enteritis of the rectum |
| 65391 | Lupus erythematosus nodularis |
| 65839 | psoriasis gyrata |
| 66238 | Crohn's disease of the ileum unspecified |
| 66711 | [x]other psoriasis |
| 68680 | coeliac rickets |
| 69959 | [X]Other Crohn's disease |
| 71083 | Juvenile arthritis in ulcerative colitis |
| 71945 | Regional enteritis of the duodenum |
| 72595 | Sarcoidosis of inferior turbinates |
| 73284 | [X]Sarcoidosis of other and combined sites |
| 93511 | chronic large plaque psoriasis |
| 94837 | Family history of sarcoidosis |
| 96880 | psoriatic arthritis |
| 99435 | Neonatal lupus erythematosus |
| 101433 | Cerebral lupus |
| 105229 | palmoplantar pustular psoriasis |
| 107494 | flexural psoriasis |

## Rheumatoid arthritis (medcode, Read)

| medcode | readterm |
| --- | --- |
| 844 | Rheumatoid arthritis |
| 4186 | Juvenile rheumatoid arthritis - Still's disease |
| 5723 | Rheumatoid nodule |
| 6916 | Seronegative rheumatoid arthritis |
| 8350 | Flare of rheumatoid arthritis |
| 9707 | Seropositive errosive rheumatoid arthritis |
| 9954 | Rheumatoid lung |
| 12019 | Seropositive rheumatoid arthritis, unspecified |
| 18155 | Rheumatoid bursitis |
| 21358 | Rheumatoid arthritis of shoulder |
| 21533 | Pauciarticular juvenile rheumatoid arthritis |
| 23552 | Felty's syndrome |
| 23834 | Adult Still's Disease |
| 27557 | Juvenile rheumatoid arthritis NOS |
| 27603 | Rheumatoid arthritis and other inflammatory polyarthropathy |
| 28853 | Fibrosing alveolitis associated with rheumatoid arthritis |
| 30548 | Rheumatoid vasculitis |
| 31054 | Rheumatoid arthritis - multiple joint |
| 31209 | Myopathy due to rheumatoid arthritis |
| 31360 | Juvenile rheumatoid arthritis |
| 31724 | Rheumatoid lung |
| 32001 | Adult-onset Still's disease |
| 33264 | O/E-hands-rheumatoid spindling |
| 36276 | Monarticular juvenile rheumatoid arthritis |
| 37431 | Rheumatoid arthropathy + visceral/systemic involvement NOS |
| 41941 | Rheumatoid arthritis of PIP joint of finger |
| 42299 | Rheumatoid arthritis of MCP joint |
| 43816 | Rheumatoid carditis |
| 44203 | Other rheumatoid arthritis of spine |
| 44743 | Rheumatoid arthritis of cervical spine |
| 46436 | Rheumatoid lung disease |
| 47831 | Acute polyarticular juvenile rheumatoid arthritis |
| 48832 | Rheumatoid arthritis of wrist |
| 49067 | Rheumatoid arthritis of hip |
| 49227 | Other rheumatoid arthropathy + visceral/systemic involvement |
| 49787 | Rheumatoid myocarditis |
| 50644 | Juvenile rheumatoid arthropathy unspecified |
| 50863 | Rheumatoid arthritis of knee |
| 51238 | Rheumatoid arthritis of 1st MTP joint |
| 51239 | Rheumatoid arthritis of ankle |
| 53621 | Rheumatoid nodule |
| 56202 | [X]Seropositive rheumatoid arthritis, unspecified |
| 56838 | Caplan's syndrome |
| 59738 | Rheumatoid arthritis of elbow |
| 62401 | Polyneuropathy in rheumatoid arthritis |
| 63198 | Rheumatoid arthritis of DIP joint of finger |
| 63365 | Rheumatoid arthritis of distal radio-ulnar joint |
| 70221 | [X]Other specified rheumatoid arthritis |
| 70658 | Rheumatoid arthritis of talonavicular joint |
| 71784 | Rheumatoid arthritis of other tarsal joint |
| 73619 | Rheumatoid arthritis of subtalar joint |
| 93715 | [X]Other seropositive rheumatoid arthritis |
| 99414 | Rheumatoid arthritis of lesser MTP joint |
| 100776 | Rheumatoid arthritis of sacro-iliac joint |
| 100914 | Rheumatoid arthritis of acromioclavicular joint |
| 102088 | Delivery of rehabilitation for rheumatoid arthritis |

## Chronic Kidney disease (medcode, Read)

| medcode | readcode | readterm | stage_1_2 | ckd_3_5 | dialysis | transplant |
| --- | --- | --- | --- | --- | --- | --- |
| 109750 | 2126E00 | Chronic kidney disease resolved | 1 |  |  |  |
| 29013 | 1Z10.00 | Chronic kidney disease stage 1 | 1 |  |  |  |
| 12586 | 1Z11.00 | Chronic kidney disease stage 2 | 1 |  |  |  |
| 94789 | 1Z17.00 | Chronic kidney disease stage 1 with proteinuria | 1 |  |  |  |
| 97980 | 1Z17.11 | CKD stage 1 with proteinuria | 1 |  |  |  |
| 95572 | 1Z18.00 | Chronic kidney disease stage 1 without proteinuria | 1 |  |  |  |
| 111022 | 1Z18.11 | CKD stage 1 without proteinuria | 1 |  |  |  |
| 95146 | 1Z19.00 | Chronic kidney disease stage 2 with proteinuria | 1 |  |  |  |
| 97979 | 1Z19.11 | CKD stage 2 with proteinuria | 1 |  |  |  |
| 95121 | 1Z1A.00 | Chronic kidney disease stage 2 without proteinuria | 1 |  |  |  |
| 97978 | 1Z1A.11 | CKD stage 2 without proteinuria | 1 |  |  |  |
| 94793 | 1Z1B.00 | Chronic kidney disease stage 3 with proteinuria | 1 |  |  |  |
| 110033 | 1Z1M.00 | CKD with GFR category G1 & albuminuria category A1 | 1 |  |  |  |
| 110003 | 1Z1N.00 | CKD with GFR category G1 & albuminuria category A2 | 1 |  |  |  |
| 110484 | 1Z1P.00 | CKD with GFR category G1 & albuminuria category A3 | 1 |  |  |  |
| 110269 | 1Z1Q.00 | CKD with GFR category G2 & albuminuria category A1 | 1 |  |  |  |
| 110108 | 1Z1R.00 | CKD with GFR category G2 & albuminuria category A2 | 1 |  |  |  |
| 110251 | 1Z1S.00 | CKD with GFR category G2 & albuminuria category A3 | 1 |  |  |  |
| 105392 | K051.00 | Chronic kidney disease stage 1 | 1 |  |  |  |
| 105383 | K052.00 | Chronic kidney disease stage 2 | 1 |  |  |  |
| 12566 | 1Z12.00 | Chronic kidney disease stage 3 |  | 1 |  |  |
| 94965 | 1Z15.00 | Chronic kidney disease stage 3A |  | 1 |  |  |
| 95179 | 1Z16.00 | Chronic kidney disease stage 3B |  | 1 |  |  |
| 95145 | 1Z1B.11 | CKD stage 3 with proteinuria |  | 1 |  |  |
| 95123 | 1Z1C.00 | Chronic kidney disease stage 3 without proteinuria |  | 1 |  |  |
| 95188 | 1Z1C.11 | CKD stage 3 without proteinuria |  | 1 |  |  |
| 95408 | 1Z1D.00 | Chronic kidney disease stage 3A with proteinuria |  | 1 |  |  |
| 95571 | 1Z1D.11 | CKD stage 3A with proteinuria |  | 1 |  |  |
| 95175 | 1Z1E.00 | Chronic kidney disease stage 3A without proteinuria |  | 1 |  |  |
| 95176 | 1Z1E.11 | CKD stage 3A without proteinuria |  | 1 |  |  |
| 95178 | 1Z1F.00 | Chronic kidney disease stage 3B with proteinuria |  | 1 |  |  |
| 95180 | 1Z1F.11 | CKD stage 3B with proteinuria |  | 1 |  |  |
| 95177 | 1Z1G.00 | Chronic kidney disease stage 3B without proteinuria |  | 1 |  |  |
| 100633 | 1Z1G.11 | CKD stage 3B without proteinuria |  | 1 |  |  |
| 109804 | 1Z1T.00 | CKD with GFR category G3a & albuminuria category A1 |  | 1 |  |  |
| 109805 | 1Z1V.00 | CKD with GFR category G3a & albuminuria category A2 |  | 1 |  |  |
| 109905 | 1Z1W.00 | CKD with GFR category G3a & albuminuria category A3 |  | 1 |  |  |
| 109963 | 1Z1X.00 | CKD with GFR category G3b & albuminuria category A1 |  | 1 |  |  |
| 109657 | 1Z1Y.00 | CKD with GFR category G3b & albuminuria category A2 |  | 1 |  |  |
| 109990 | 1Z1Z.00 | CKD with GFR category G3b & albuminuria category A3 |  | 1 |  |  |
| 104619 | K053.00 | Chronic kidney disease stage 3 |  | 1 |  |  |
| 12479 | 1Z13.00 | Chronic kidney disease stage 4 |  | 1 |  |  |
| 12585 | 1Z14.00 | Chronic kidney disease stage 5 |  | 1 |  |  |
| 109980 | 1Z1a.00 | CKD with GFR category G4 & albuminuria category A1 |  | 1 |  |  |
| 109904 | 1Z1b.00 | CKD with GFR category G4 & albuminuria category A2 |  | 1 |  |  |
| 110626 | 1Z1c.00 | CKD with GFR category G4 & albuminuria category A3 |  | 1 |  |  |
| 110133 | 1Z1d.00 | CKD with GFR category G5 & albuminuria category A1 |  | 1 |  |  |
| 109981 | 1Z1e.00 | CKD with GFR category G5 & albuminuria category A2 |  | 1 |  |  |
| 110467 | 1Z1f.00 | CKD with GFR category G5 & albuminuria category A3 |  | 1 |  |  |
| 95122 | 1Z1H.00 | Chronic kidney disease stage 4 with proteinuria |  | 1 |  |  |
| 99312 | 1Z1H.11 | CKD stage 4 with proteinuria |  | 1 |  |  |
| 95406 | 1Z1J.00 | Chronic kidney disease stage 4 without proteinuria |  | 1 |  |  |
| 97587 | 1Z1J.11 | CKD stage 4 without proteinuria |  | 1 |  |  |
| 95508 | 1Z1K.00 | Chronic kidney disease stage 5 with proteinuria |  | 1 |  |  |
| 99160 | 1Z1K.11 | CKD stage 5 with proteinuria |  | 1 |  |  |
| 95405 | 1Z1L.00 | Chronic kidney disease stage 5 without proteinuria |  | 1 |  |  |
| 97683 | 1Z1L.11 | CKD stage 5 without proteinuria |  | 1 |  |  |
| 16929 | D215.00 | Anaemia secondary to renal failure |  | 1 |  |  |
| 25394 | D215000 | Anaemia secondary to chronic renal failure |  | 1 |  |  |
| 32423 | G222.00 | Hypertensive renal disease with renal failure |  | 1 |  |  |
| 28684 | G233.00 | Hypertensive heart and renal disease with renal failure |  | 1 |  |  |
| 57987 | G234.00 | Hyperten heart&renal dis+both(congestv)heart and renal fail |  | 1 |  |  |
| 26860 | G752000 | Goodpasture's syndrome |  | 1 |  |  |
| 109840 | G752100 | Goodpasture's disease |  | 1 |  |  |
| 2999 | K01..00 | Nephrotic syndrome |  | 1 |  |  |
| 9840 | K010.00 | Nephrotic syndrome with proliferative glomerulonephritis |  | 1 |  |  |
| 1803 | K011.00 | Nephrotic syndrome with membranous glomerulonephritis |  | 1 |  |  |
| 99644 | K012.00 | Nephrotic syndrome+membranoproliferative glomerulonephritis |  | 1 |  |  |
| 29634 | K013.00 | Nephrotic syndrome with minimal change glomerulonephritis |  | 1 |  |  |
| 40349 | K013.11 | Lipoid nephrosis |  | 1 |  |  |
| 57926 | K013.12 | Steroid sensitive nephrotic syndrome |  | 1 |  |  |
| 23913 | K014.00 | Nephrotic syndrome minor glomerular abnormality |  | 1 |  |  |
| 22852 | K015.00 | Nephrotic syndrome focal and segmental glomerular lesions |  | 1 |  |  |
| 19316 | K016.00 | Nephrotic syndrome diffuse membranous glomerulonephritis |  | 1 |  |  |
| 21947 | K017.00 | Nephrotic syn difus mesangial prolifertiv glomerulonephritis |  | 1 |  |  |
| 50472 | K018.00 | Nephrotic syn difus endocapilary proliftv glomerulonephritis |  | 1 |  |  |
| 21989 | K019.00 | Nephrotic syn diffuse mesangiocapillary glomerulonephritis |  | 1 |  |  |
| 56987 | K01A.00 | Nephrotic syndrome dense deposit disease |  | 1 |  |  |
| 17365 | K01B.00 | Nephrotic syndrome diffuse crescentic glomerulonephritis |  | 1 |  |  |
| 63786 | K01w.00 | Congenital nephrotic syndrome |  | 1 |  |  |
| 72303 | K01w000 | Finnish nephrosis syndrome |  | 1 |  |  |
| 108591 | K01w100 | Drash syndrome |  | 1 |  |  |
| 112548 | K01w111 | Nephrotic syndrome with pseudohermaphroditism |  | 1 |  |  |
| 108922 | K01w112 | Wilms' tumour + nephrotic syndrome + pseudohermaphroditism |  | 1 |  |  |
| 110749 | K01w200 | Congenital nephrotic syndrome with focal glomerulosclerosis |  | 1 |  |  |
| 111370 | K01wz00 | Congenital nephrotic syndrome NOS |  | 1 |  |  |
| 108816 | K01x.00 | Nephrotic syndrome in diseases EC |  | 1 |  |  |
| 47922 | K01x000 | Nephrotic syndrome in amyloidosis |  | 1 |  |  |
| 2471 | K01x100 | Nephrotic syndrome in diabetes mellitus |  | 1 |  |  |
| 99201 | K01x200 | Nephrotic syndrome in malaria |  | 1 |  |  |
| 58750 | K01x300 | Nephrotic syndrome in polyarteritis nodosa |  | 1 |  |  |
| 47672 | K01x400 | Nephrotic syndrome in systemic lupus erythematosus |  | 1 |  |  |
| 94373 | K01y.00 | Nephrotic syndrome with other pathological kidney lesions |  | 1 |  |  |
| 27427 | K01z.00 | Nephrotic syndrome NOS |  | 1 |  |  |
| 65064 | K023.00 | Chronic rapidly progressive glomerulonephritis |  | 1 |  |  |
| 58164 | K033.00 | Rapidly progressive nephritis unspecified |  | 1 |  |  |
| 512 | K05..00 | Chronic renal failure |  | 1 |  |  |
| 53852 | K05..12 | End stage renal failure |  | 1 |  |  |
| 6712 | K050.00 | End stage renal failure |  | 1 |  |  |
| 104963 | K054.00 | Chronic kidney disease stage 4 |  | 1 |  |  |
| 105151 | K055.00 | Chronic kidney disease stage 5 |  | 1 |  |  |
| 61814 | K0A0700 | Acute nephrotic syndrm diffuse crescentic glomerulonephritis |  | 1 |  |  |
| 71174 | K0A1.00 | Rapidly progressive nephritic syndrome |  | 1 |  |  |
| 97734 | K0A1100 | Rapid progres nephritic syn focal+segmental glomerulr lesion |  | 1 |  |  |
| 41285 | K0A1200 | Rapid progres neph syn diffuse membranous glomerulonephritis |  | 1 |  |  |
| 58060 | K0A1300 | Rpd prog neph syn df mesangial prolifratv glomerulonephritis |  | 1 |  |  |
| 109945 | K0A1400 | Rapid progres neph syn df endocapilary prolifv glomnephritis |  | 1 |  |  |
| 50200 | K0A1600 | Rapid progressive nephritic syndrome dense deposit disease |  | 1 |  |  |
| 62320 | K0A1700 | Rapid progres nephritic syn df crescentic glomerulonephritis |  | 1 |  |  |
| 105859 | K0A8.00 | Rapidly progressive glomerulonephritis |  | 1 |  |  |
| 48057 | K0B5.00 | Renal tubulo-interstitial disordrs in transplant rejectn |  | 1 |  |  |
| 8330 | K0D..00 | End-stage renal disease |  | 1 |  |  |
| 100205 | K0E..00 | Acute-on-chronic renal failure |  | 1 |  |  |
| 53940 | Kyu2100 | [X]Other chronic renal failure |  | 1 |  |  |
| 49028 | 14S2.00 | H/O: kidney recipient |  |  |  | 1 |
| 2997 | 7B00.00 | Transplantation of kidney |  |  |  | 1 |
| 55151 | 7B00000 | Autotransplant of kidney |  |  |  | 1 |
| 11745 | 7B00100 | Transplantation of kidney from live donor |  |  |  | 1 |
| 66705 | 7B00111 | Allotransplantation of kidney from live donor |  |  |  | 1 |
| 24361 | 7B00200 | Transplantation of kidney from cadaver |  |  |  | 1 |
| 98364 | 7B00211 | Allotransplantation of kidney from cadaver |  |  |  | 1 |
| 105328 | 7B00212 | Cadaveric renal transplant |  |  |  | 1 |
| 89924 | 7B00300 | Allotransplantation of kidney from cadaver, heart-beating |  |  |  | 1 |
| 96133 | 7B00400 | Allotransplantation kidney from cadaver, heart non-beating |  |  |  | 1 |
| 109455 | 7B00500 | Allotransplantation of kidney from cadaver NEC |  |  |  | 1 |
| 105787 | 7B00600 | Xenograft renal transplant |  |  |  | 1 |
| 70874 | 7B00y00 | Other specified transplantation of kidney |  |  |  | 1 |
| 5504 | 7B00z00 | Transplantation of kidney NOS |  |  |  | 1 |
| 51039 | 7B01200 | Bilateral nephrectomy |  |  |  | 1 |
| 48121 | 7B01500 | Transplant nephrectomy |  |  |  | 1 |
| 72004 | 7B01511 | Excision of rejected transplanted kidney |  |  |  | 1 |
| 26862 | 7B06300 | Exploration of renal transplant |  |  |  | 1 |
| 93366 | 7B0F.00 | Interventions associated with transplantation of kidney |  |  |  | 1 |
| 90952 | 7B0F100 | Pre-transplantation of kidney work-up, recipient |  |  |  | 1 |
| 103429 | 7B0F300 | Post-transplantation of kidney examination, recipient |  |  |  | 1 |
| 104050 | 7B0Fy00 | OS interventions associated with transplantation of kidney |  |  |  | 1 |
| 104049 | 7B0Fz00 | Interventions associated with transplantation of kidney NOS |  |  |  | 1 |
| 17253 | 8L50.00 | Renal transplant planned |  |  |  | 1 |
| 100693 | Kyu1C00 | [X]Renal tubulo-interstitial disorders/transplant rejection |  |  |  | 1 |
| 70712 | SP08011 | Det.ren.func.after ren.transpl |  |  |  | 1 |
| 11553 | SP08300 | Kidney transplant failure and rejection |  |  |  | 1 |
| 104905 | SP08D00 | Acute-on-chronic rejection of renal transplant |  |  |  | 1 |
| 104960 | SP08E00 | Acute rejection of renal transplant - grade I |  |  |  | 1 |
| 107000 | SP08F00 | Acute rejection of renal transplant - grade II |  |  |  | 1 |
| 104630 | SP08G00 | Acute rejection of renal transplant - grade III |  |  |  | 1 |
| 104201 | SP08H00 | Acute rejection of renal transplant |  |  |  | 1 |
| 106620 | SP08J00 | Chronic rejection of renal transplant |  |  |  | 1 |
| 105724 | SP08N00 | Unexplained episode of renal transplant dysfunction |  |  |  | 1 |
| 106301 | SP08P00 | Stenosis of vein of transplanted kidney |  |  |  | 1 |
| 105811 | SP08R00 | Renal transplant rejection |  |  |  | 1 |
| 107752 | SP08T00 | Urological complication of renal transplant |  |  |  | 1 |
| 108437 | SP08V00 | Very mild acute rejection of renal transplant |  |  |  | 1 |
| 106866 | SP08W00 | Vascular complication of renal transplant |  |  |  | 1 |
| 54990 | TB00100 | Kidney transplant with complication, without blame |  |  |  | 1 |
| 18774 | TB00111 | Renal transplant with complication, without blame |  |  |  | 1 |
| 5911 | ZV42000 | [V]Kidney transplanted |  |  |  | 1 |
| 24151 | 7A60.00 | Arteriovenous shunt |  |  | 1 |  |
| 63063 | 7A60000 | Insertion of arteriovenous prosthesis |  |  | 1 |  |
| 3205 | 7A60100 | Creation of arteriovenous fistula NEC |  |  | 1 |  |
| 28269 | 7A60111 | Creation of radial-cephalic fistula |  |  | 1 |  |
| 25521 | 7A60112 | Creation of brachial-cephalic fistula |  |  | 1 |  |
| 63190 | 7A60200 | Attention to arteriovenous shunt |  |  | 1 |  |
| 31478 | 7A60300 | Removal of infected arteriovenous shunt |  |  | 1 |  |
| 96131 | 7A60400 | Banding of arteriovenous fistula |  |  | 1 |  |
| 63305 | 7A60500 | Thrombectomy of arteriovenous fistula |  |  | 1 |  |
| 60302 | 7A60600 | Creation of graft fistula for dialysis |  |  | 1 |  |
| 65398 | 7A60y00 | Other specified arteriovenous shunt |  |  | 1 |  |
| 58618 | 7A60z00 | Arteriovenous shunt NOS |  |  | 1 |  |
| 18779 | 7A61100 | Repair of acquired arteriovenous fistula |  |  | 1 |  |
| 100235 | 7A61111 | Ligation of acquired arteriovenous fistula |  |  | 1 |  |
| 9765 | 7A61400 | Ligation of acquired arteriovenous fistula |  |  | 1 |  |
| 96347 | 7A61900 | Ligation of arteriovenous dialysis fistula |  |  | 1 |  |
| 107719 | 7A61A00 | Ligation of arteriovenous dialysis graft |  |  | 1 |  |
| 31549 | 7L1A.00 | Compensation for renal failure |  |  | 1 |  |
| 11773 | 7L1A.11 | Dialysis for renal failure |  |  | 1 |  |
| 20073 | 7L1A000 | Renal dialysis |  |  | 1 |  |
| 101756 | 7L1A011 | Thomas intravascular shunt for dialysis |  |  | 1 |  |
| 2994 | 7L1A100 | Peritoneal dialysis |  |  | 1 |  |
| 2996 | 7L1A200 | Haemodialysis NEC |  |  | 1 |  |
| 71124 | 7L1A300 | Haemofiltration |  |  | 1 |  |
| 88597 | 7L1A400 | Automated peritoneal dialysis |  |  | 1 |  |
| 30756 | 7L1A500 | Continuous ambulatory peritoneal dialysis |  |  | 1 |  |
| 64828 | 7L1A600 | Peritoneal dialysis NEC |  |  | 1 |  |
| 48022 | 7L1Ay00 | Other specified compensation for renal failure |  |  | 1 |  |
| 64636 | 7L1Az00 | Compensation for renal failure NOS |  |  | 1 |  |
| 56760 | 7L1B.00 | Placement ambulatory apparatus compensation renal failure |  |  | 1 |  |
| 36442 | 7L1B.11 | Placement ambulatory dialysis apparatus - compens renal fail |  |  | 1 |  |
| 8037 | 7L1B000 | Insertion of ambulatory peritoneal dialysis catheter |  |  | 1 |  |
| 23773 | 7L1B100 | Removal of ambulatory peritoneal dialysis catheter |  |  | 1 |  |
| 104586 | 7L1B200 | Flushing of peritoneal dialysis catheter |  |  | 1 |  |
| 59194 | 7L1By00 | Placement ambulatory apparatus- compensate renal failure OS |  |  | 1 |  |
| 83513 | 7L1C.00 | Placement other apparatus for compensation for renal failure |  |  | 1 |  |
| 30709 | 7L1C000 | Insertion of temporary peritoneal dialysis catheter |  |  | 1 |  |
| 107901 | 7L1Cy00 | Placement other apparatus- compensate for renal failure OS |  |  | 1 |  |
| 65089 | 7L1Cz00 | Placement other apparatus- compensate for renal failure NOS |  |  | 1 |  |
| 102210 | 7L1f000 | Extracorporeal albumin haemodialysis |  |  | 1 |  |
| 105760 | G72C.00 | Ruptured aneurysm of dialysis vascular access |  |  | 1 |  |
| 107188 | G72D.00 | Aneurysm of dialysis arteriovenous fistula |  |  | 1 |  |
| 110095 | G72D000 | Aneurysm of superficialised artery of dialysis AV fistula |  |  | 1 |  |
| 107220 | G72D100 | Aneurysm of needle site of dialysis arteriovenous fistula |  |  | 1 |  |
| 105742 | G72D200 | Aneurysm of anastomotic site of dialysis AV fistula |  |  | 1 |  |
| 2995 | G760.00 | Acquired arteriovenous fistula |  |  | 1 |  |
| 107746 | Gy1..00 | Stenosis of dialysis vascular access |  |  | 1 |  |
| 108699 | Gy10.00 | Stenosis of dialysis arteriovenous graft |  |  | 1 |  |
| 109809 | Gy2..00 | Thrombosis of dialysis vascular access |  |  | 1 |  |
| 106720 | Gy21.00 | Thrombosis of dialysis arteriovenous fistula |  |  | 1 |  |
| 108116 | Gy3..00 | Occlusion of dialysis vascular access |  |  | 1 |  |
| 109135 | Gy30.00 | Occlusion of dialysis arteriovenous graft |  |  | 1 |  |
| 107082 | Gy31.00 | Occlusion of dialysis arteriovenous fistula |  |  | 1 |  |
| 110051 | Gy4..00 | Infection of dialysis vascular access |  |  | 1 |  |
| 108213 | Gy40.00 | Infection of dialysis arteriovenous graft |  |  | 1 |  |
| 107260 | Gy41.00 | Infection of dialysis arteriovenous fistula |  |  | 1 |  |
| 108759 | Gy5..00 | Haemorrhage of dialysis vascular access |  |  | 1 |  |
| 106975 | Gy51.00 | Haemorrhage of dialysis arteriovenous fistula |  |  | 1 |  |
| 108423 | Gy60.00 | Rupture of dialysis arteriovenous graft |  |  | 1 |  |
| 107900 | SP0E.00 | Disorders associated with peritoneal dialysis |  |  | 1 |  |
| 111103 | SP0E100 | Thrombus in peritoneal dialysis catheter |  |  | 1 |  |
| 108785 | SP0F.00 | Haemodialysis first use syndrome |  |  | 1 |  |
| 105436 | SP0G.00 | Anaphylactoid reaction due to haemodialysis |  |  | 1 |  |
| 109884 | SP0H.00 | Disorder associated with dialysis |  |  | 1 |  |
| 96184 | TA02000 | Accid cut puncture perf h'ge - kidney dialysis |  |  | 1 |  |
| 69427 | TA02z00 | Accid cut puncture perf h'ge - perfusion NOS |  |  | 1 |  |
| 35921 | TA22.00 | Failure of sterile precautions during perfusion |  |  | 1 |  |
| 69266 | TA22000 | Failure of sterile precautions during kidney dialysis |  |  | 1 |  |
| 111637 | TA42000 | Mechanical failure of apparatus during kidney dialysis |  |  | 1 |  |
| 28158 | TB11.00 | Kidney dialysis with complication without blame |  |  | 1 |  |
| 66714 | TB11.11 | Renal dialysis with complication without blame |  |  | 1 |  |
| 54844 | U612200 | [X]Failure sterile precautions dur kidney dialys/other perf |  |  | 1 |  |
| 110072 | Z919200 | Washing back through haemodialysis lines |  |  | 1 |  |
| 22252 | ZV45100 | [V]Renal dialysis status |  |  | 1 |  |
| 60743 | ZV56.00 | [V]Aftercare involving intermittent dialysis |  |  | 1 |  |
| 46145 | ZV56011 | [V]Aftercare involving renal dialysis NOS |  |  | 1 |  |
| 69760 | ZVu3G00 | [X]Other dialysis |  |  | 1 |  |

# Serum creatinine

## Ever had a serum creatinine test (medcode)

| medcode | readterm |
| --- | --- |
| 5 | serum creatinine |
| 522 | test - laboratory |
| 2160 | laboratory procedure nos |
| 2379 | seen in diabetic clinic |
| 2998 | renal function tests |
| 3927 | serum creatinine raised |
| 3974 | laboratory procedure performed |
| 3980 | renal function tests abnormal |
| 4265 | renal function tests normal |
| 5458 | renal function monitoring |
| 6842 | impaired renal function |
| 8413 | investigation-laboratory |
| 8919 | impaired renal function disorder |
| 10768 | [d]renal function test abnormal |
| 11365 | referral for laboratory tests |
| 11867 | laboratory procedures |
| 13736 | plasma creatinine level |
| 13812 | blood urea/renal function |
| 21172 | laboratory test requested |
| 21687 | gout due to impairment of renal function |
| 21777 | refer to pathology laboratory |
| 23208 | laboratory procedures -general |
| 24460 | laboratory test due |
| 25763 | renal function tests borderline |
| 25980 | impaired renal function disorder nos |
| 26001 | deteriorating renal function |
| 26903 | serum creatinine normal |
| 26943 | blood urea/renal function nos |
| 27095 | creatinine level |
| 30689 | laboratory test nos due |
| 31277 | serum creatinine abnormal |
| 33251 | [v]laboratory examination |
| 35545 | serum creatinine low |
| 37236 | renal function test nos |
| 39840 | other impaired renal function disorder |
| 41013 | renal function impairment with growth failure |
| 42345 | serum creatinine nos |
| 44890 | [d]unexplained laboratory result |
| 45096 | corrected serum creatinine level |
| 49876 | laboratory result |
| 50804 | other impaired renal function disorder nos |
| 56293 | differential renal function |
| 62062 | corrected plasma creatinine level |
| 91616 | laboratory administration procedures |
| 94847 | review of patient laboratory test report |
| 97404 | laboratory request |
| 101976 | recovery of renal function |
| 106389 | renal function monitoring invitation |
| 106653 | reason for repeat laboratory test |
| 107647 | renal function monitoring invitation first letter |
| 107978 | specimen received in laboratory |
